# Palladium-Catalyzed Addition of Aryl Halides to *N*-Sulfinylamines for the Synthesis of Sulfinamides

**DOI:** 10.1021/jacs.4c06726

**Published:** 2024-07-12

**Authors:** Ming-Kai Wei, Daniel F. Moseley, Robin M. Bär, Yeshua Sempere, Michael C. Willis

**Affiliations:** † Department of Chemistry, University of Oxford, Mansfield Road, Oxford OX1 3TA, United Kingdom; ‡ Research & Development, Crop Science, Bayer AG, Alfred-Nobel-Str. 50, Monheim am Rhein 40789, Germany

## Abstract

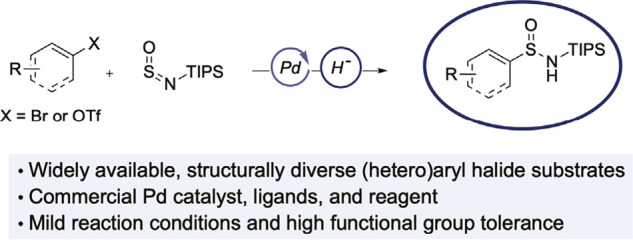

Sulfinamides are versatile, synthetically useful intermediates, and final motifs. Traditional methods to synthesize sulfinamides generally require substrates with preinstalled sulfur centers. However, these precursors have limited commercial availability, and the associated synthetic routes often require harsh reaction conditions and highly reactive reagents, thus severely limiting their application. Herein, we report the synthesis of sulfinamides from aryl and alkenyl (pseudo)halides and *N*-sulfinylamines, enabled by palladium catalysis. The reactions use mild conditions and are achieved without the use of highly reactive preformed organometallic reagents, resulting in transformations of broad generality and high functional group tolerance. In particular, substrates featuring protic and electrophilic functional groups can be used successfully. The modification of complex aryl cores and natural product derivatives demonstrates the utility of this method.

Sulfinamides are valuable, flexible building blocks in both organic synthesis and medicinal- and agro-chemistry. For example, enantiopure sulfinamides have extensive applications as ligands in transition metal and organo-catalysis,^1^ and as chiral auxiliaries for the synthesis of enantioenriched amines (Scheme 1a).^2^ Sulfinamides have been used as amide bioisosteres^3^ and have found applications in treatments for hepatitis C^4^ and leukemia.^5^ Importantly, sulfinamides can be easily transformed into alternative high-value sulfur functional groups such as sulfonamides, sulfonimidamides, and sulfonimidoyl fluorides.^6^ Sulfonamides, in particular, are prized sulfur functional groups in medicinal chemistry,^7^ and there are over 150 FDA-approved sulfonamide containing drugs (Scheme 1b).^8^ Sulfonimidamides, the mono aza-analogues of sulfonamides, are yet to appear in a marketed pharmaceutical, but feature extensively in the recent medicinal and agrochemistry patent literature;^9^Scheme 1b shows an example that is an inhibitor of the NLRP3 inflammasome,^10^ as well as a sulfonimidamide that displays herbicidal activity.^9c^ Sulfonimidoyl fluorides are important electrophilic motifs in chemical biology due to their reactivity by SuFEx pathways.^11^

**Scheme 1:**
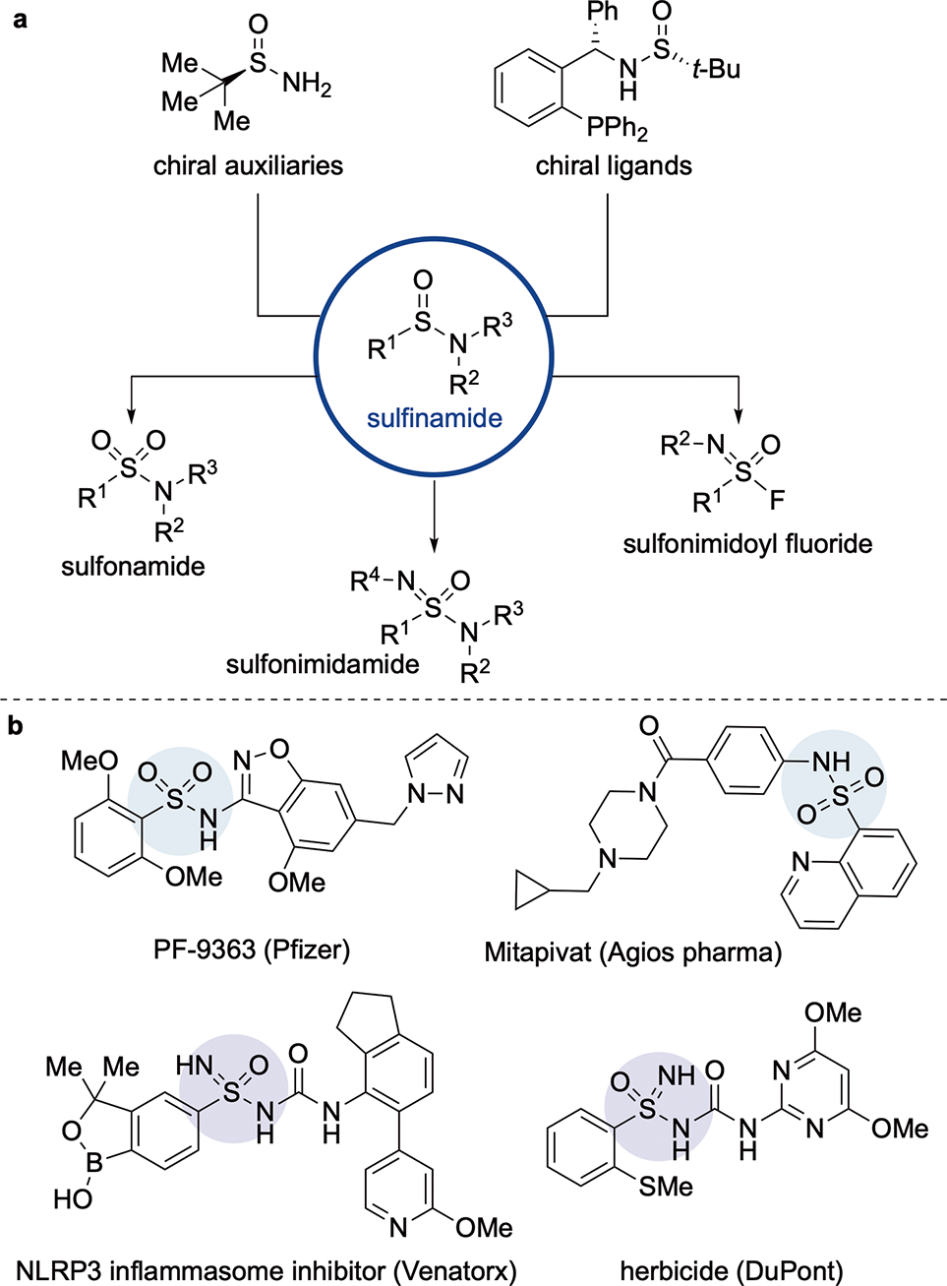
a) Examples of Chiral Sulfinamides and Conversion to Diverse S(VI)-Functional Groups; b) Examples of Bioactive Sulfonamides and Sulfonimidamides

The majority of existing methods for the synthesis of sulfinamides require substrates with preinstalled sulfur centers; common precursors include sulfonyl chlorides,^12^ thiols and disulfides,^13^ as well as sulfinyl chlorides and esters.^14^ Methods using these substrates show commendable scope and efficiency; however, the precursors all have limited commercial availability and often display compromised stability, and the synthetic methods generally utilize highly reactive reagents and harsh reaction conditions, thus limiting functional group tolerance. This final point is particularly important in medicinal and agrochemistry applications, where the synthesis of functionalized building blocks is crucial. Approaches to sulfinamides which rely on the use of nonsulfur containing carbon substrates are attractive alternatives, potentially offering solutions to many of the earlier shortcomings.^15^

An early example of this approach involves the addition of preformed organometallic reagents into *N*-sulfinylamines, and although efficient, the limited functional group tolerance remains (Scheme 2a).^15a^ Other substrates that have been used include aryl diazonium salts, aryl potassium trifluoroborate salts, and aryl boroxines (Scheme 2a).^6b,16^ The functional group tolerance of these approaches is improved; however, none of these substrates are ideal, with the majority being challenging to handle and all having only limited commercial availability. In addition, these groups are not amenable to multistep synthetic sequences, making their use in the late-stages of complex molecule synthesis challenging. Using aryl and heteroaryl halides as substrates would address many of these issues; these are substrates with unrivaled availability and structural diversity. In addition, although reactive under specific, often catalytic, reaction conditions, these substrates are stable to a diverse range of reagents and are therefore useful in complex molecule synthesis (Scheme 2b). Using aryl halides as substrates presents a different set of challenges: (i) unlike the redox-neutral catalytic pathways exploited in the prior methods, these transformations will require a metal redox shuttle in which a reductant is required to close the catalytic cycle, however, sulfinylamines are known to be susceptible to reduction;^17^ (ii) although reductive couplings between aryl/alkenyl (pseudo) halides and SO_2_ (surrogates) have been developed, poisoning of the lower oxidation state metal complexes by SO_2_ is often noted, and the electronically similar R-NSO reagents will likely have related issues.^18^ To our best knowledge, there are no reports of *N*-sulfinylamines in combination with metal catalysts undergoing a redox event,^18b,19^ nor of the direct addition of aryl halides into sulfinylamines. Despite these challenges, the advantages realized from the successful union of aryl and heteroaryl halides with sulfinylamines using metal catalysis are significant and are the inspiration for this study.

**Scheme 2:**
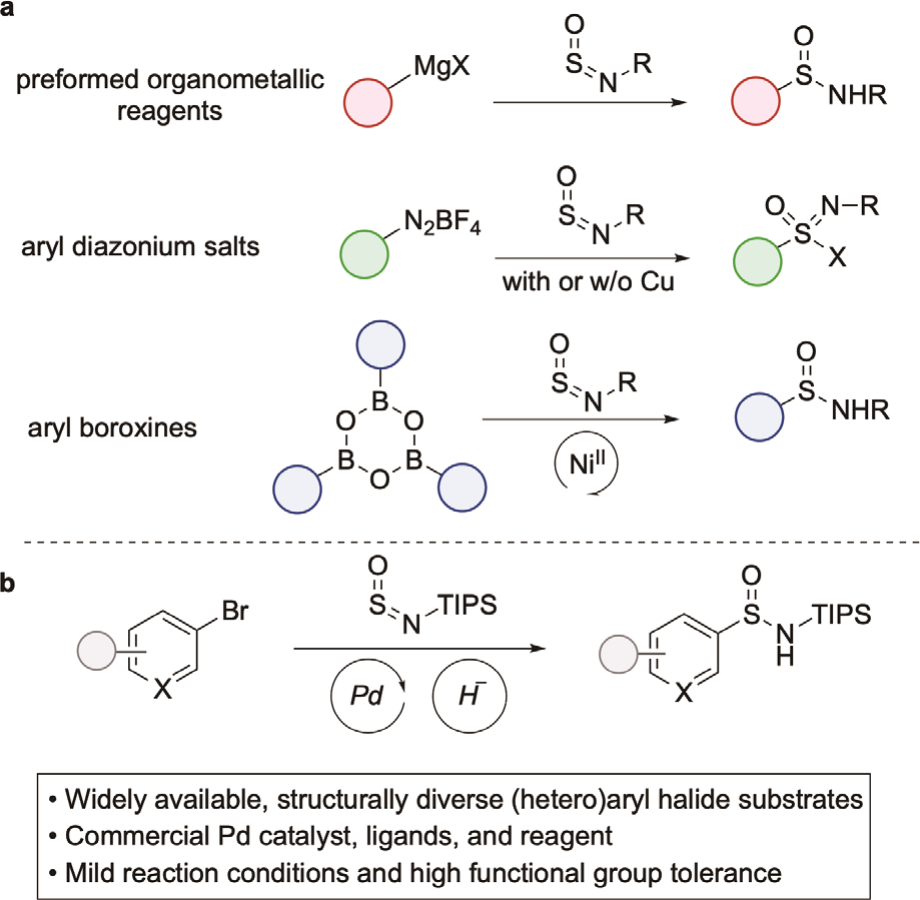
a) Sulfinamide Syntheses Using Non-sulfur Containing Aryl Substrates; b) *This work*, the Palladium-Catalysed Synthesis of Sufinamides from Aryl Halides

We initially examined the combination of *p*-fluoro bromobenzene with various sulfinylamines, leading to sulfinamides **1** (Table 1). The optimized reaction conditions involved using 10 mol % SPhos Pd G3 as the catalyst, HCO_2_Cs as the reductant, and *N*-triisopropylsilyl sulfinylamine (TIPS-NSO) in 1,4-dioxane at 75 °C for 18 h, and delivered the sulfinamide **1a** in 85% isolated yield (Table 1, entry 1, see the Supporting Information for full details). Using the alternative *N-*sulfinylamine reagent Tr-NSO (entry 2) resulted in a reduced yield, and alternative reductants and ligands were also less successful (e.g., entry 3). Using a separate palladium salt and ligand (and not the precomplexed G3 system), although effective, was less efficient than using the preformed catalyst (entry 5). Control experiments confirmed the necessity of both the Pd complex and reductant (entries 6 and 7).

**Table 1:**
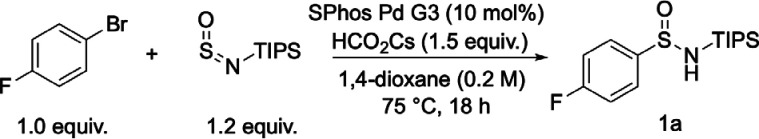
Optimization of the Synthesis of Sulfinamide **1a**^a^

entry	variations from standard	yield^a^
1	none	86% (85%)
2	Tr-NSO instead of TIPS-NSO	20%
3	CataCXium A Pd G3 instead of SPhos Pd G3	61%
4	5 mol % SPhos Pd G3, 18 h	65%
5	10 mol % Pd(OAc)_2_ + 15 mol % SPhos	75%
6	No SPhos Pd G3	0
7	No HCO_2_Cs	<5%

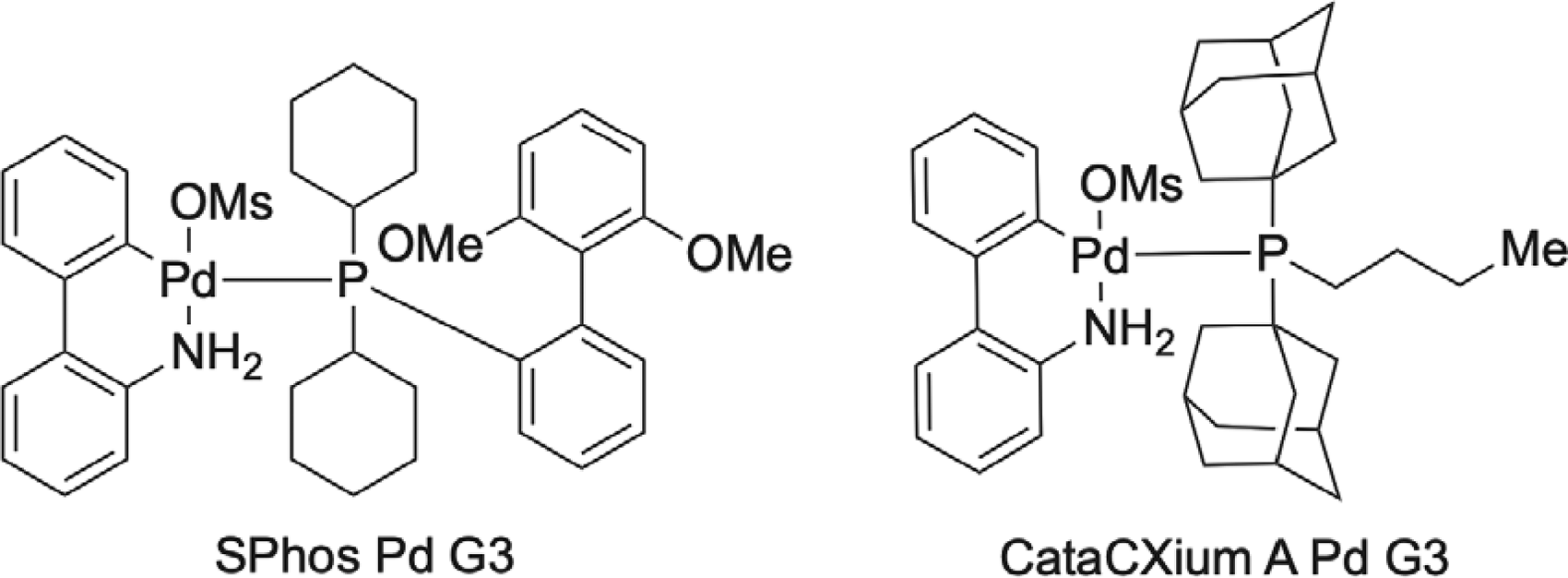

a Yields determined by quantitative ^19^F NMR spectroscopy of the crude reaction mixture using 1,4-difluorobenzene as the internal standard. Isolated yields are in parentheses.

With the optimized conditions in hand, the scope of the process with respect to aryl bromides was investigated (Table 2). Aromatics with electron-withdrawing groups at the *para*-position were well tolerated (**1a**–**1e**), including functional groups such as nitro (**1c**), nitrile (**1d**), and ketone (**1e**). These are all functional groups that would be challenging to use in approaches that rely on preformed organometallic reagents. *Meta*-substituted aryl bromides also performed well (**1f**, **1g**). A naphthalene group (**1h**), and a disubstituted benzene featuring a NH-carbamate (**1i**) were efficient substrates. However, significantly lower yields were obtained with a substrate featuring an electron-donating methoxy group positioned at the *para*-position (**1j**). Switching the catalyst to a combination of Pd(OAc)_2_ and di(1-adamantyl)benzyl phosphine, and reducing the loading of HCO_2_Cs, allowed the yield of 4-bromoanisole product **1j** to increase to 61% (see the Supporting Information for details). Using these modified conditions allowed additional electron-rich and electron-neutral aryl bromides to be successfully used, including *p*-tolyl (**1k**), *p*-succinimide (**1l**), dioxole (**1p**), and bicyclic ketone (**1q**). Several *ortho*-substituted aryl halides were competent substrates (**1m**, **1o**, and **1q**). Heteroaromatic substrates were then investigated: Pyridines (**1r**–**1u**), pyrimidine (**1v**), quinoline (**1w**), indole (**1x**), and thiophene (**1y**) substrates were well tolerated in the reaction. Additionally, several complex aryl sulfinamides, such as Celecoxib precursor (**1z**), piperidine-substituted pyridine (**1aa**), and chemical probe mimic (**1ab**), could all be synthesized.^20^ The *meta*-ester example (**1f**) was scaled to a 1 mmol reaction, and delivered 0.28 g of the sulfinamide in 78% yield using the standard reaction conditions; using 5 mol % of catalyst on the same scale provided sulfinamide **1f** in 69% yield. Aryl chlorides substrates were unreactive using the optimized conditions.

**Table 2:**
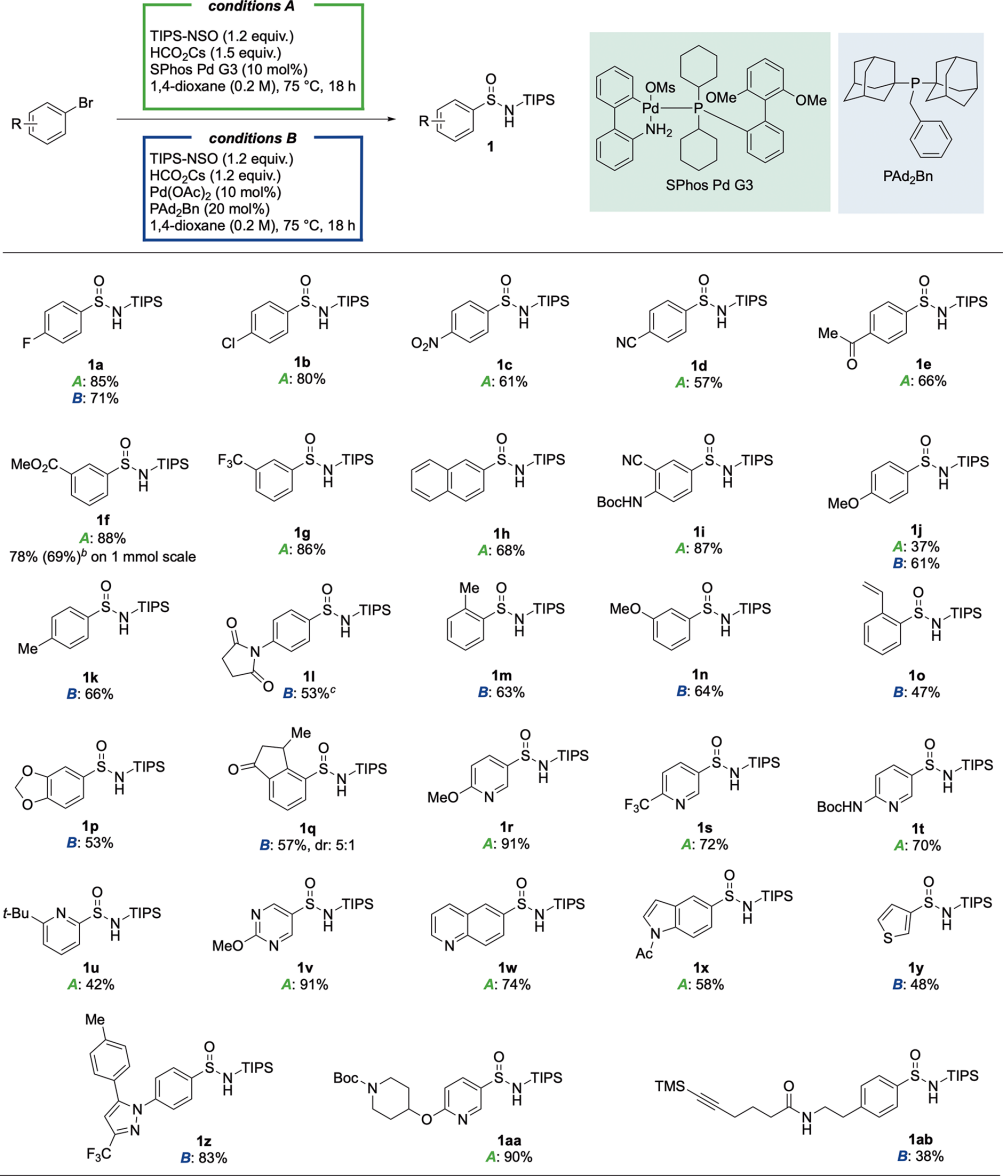
Scope of Sulfinamide Synthesis Using Aryl and Heteroaryl Bromides^a^

a Reaction conditions ***A***: aryl bromide (0.20 mmol, 1.0 equiv), TIPS-NSO (1.2 equiv), HCO_2_Cs (1.5 equiv), SPhos Pd G3 (10 mol %), 1,4-dioxane (0.2 M), 75 °C, 18 h; Reaction conditions ***B***: aryl bromide (0.20 mmol, 1.0 equiv), TIPS-NSO (1.2 equiv), HCO_2_Cs (1.2 equiv), Pd(OAc)_2_ (10 mol %), PAd_2_Bn (20 mol %), 1,4-dioxane (0.2 M), 75 °C, 18 h.

b Using 5 mol % catalyst.

c 85 °C

Having established the transformation of aryl bromides into sulfinamides, we then extended the method to include cyclic alkenyl (pseudo)halides as substrates (Table 3). Alkenyl sulfonamides are of interest in medicinal chemistry,^21^ and using these types of substrates could also contribute to the efforts to increase *sp*^3^-rich molecules in drug discovery.^22^ Using modified reaction conditions (see the Supporting Information for details), we found that the use of alkenyl triflate **2a**, in combination with HCO_2_K as the reductant, provided the corresponding alkenyl sulfinamide in a good yield (71%, **3a**). Following this lead, the scope of alkenyl triflates was investigated, with variation of ring size and substitution pattern being explored. Differentially *N*-substituted 4-piperidone derived precursors, including *N*-Boc (**3b**) and *N*-benzyl (**3c**) derivatives, delivered the products in good yields. Alkenyl triflates based on carbocyclic frameworks, including cyclopentene (**3d**) and cyclohexene (**3e**-**3g**) were well tolerated in the reaction. Tetralone-derived alkenyl triflate (**3h**) reacted smoothly, and bicyclic (**3i**, **3j**) and steroid-derived (**3k**) alkenyl triflates were also successful substrates. Importantly, a gram-scale synthesis of **3a**, using a reduced catalyst loading (5 mol %) was equally efficient (88%).

**Table 3:**
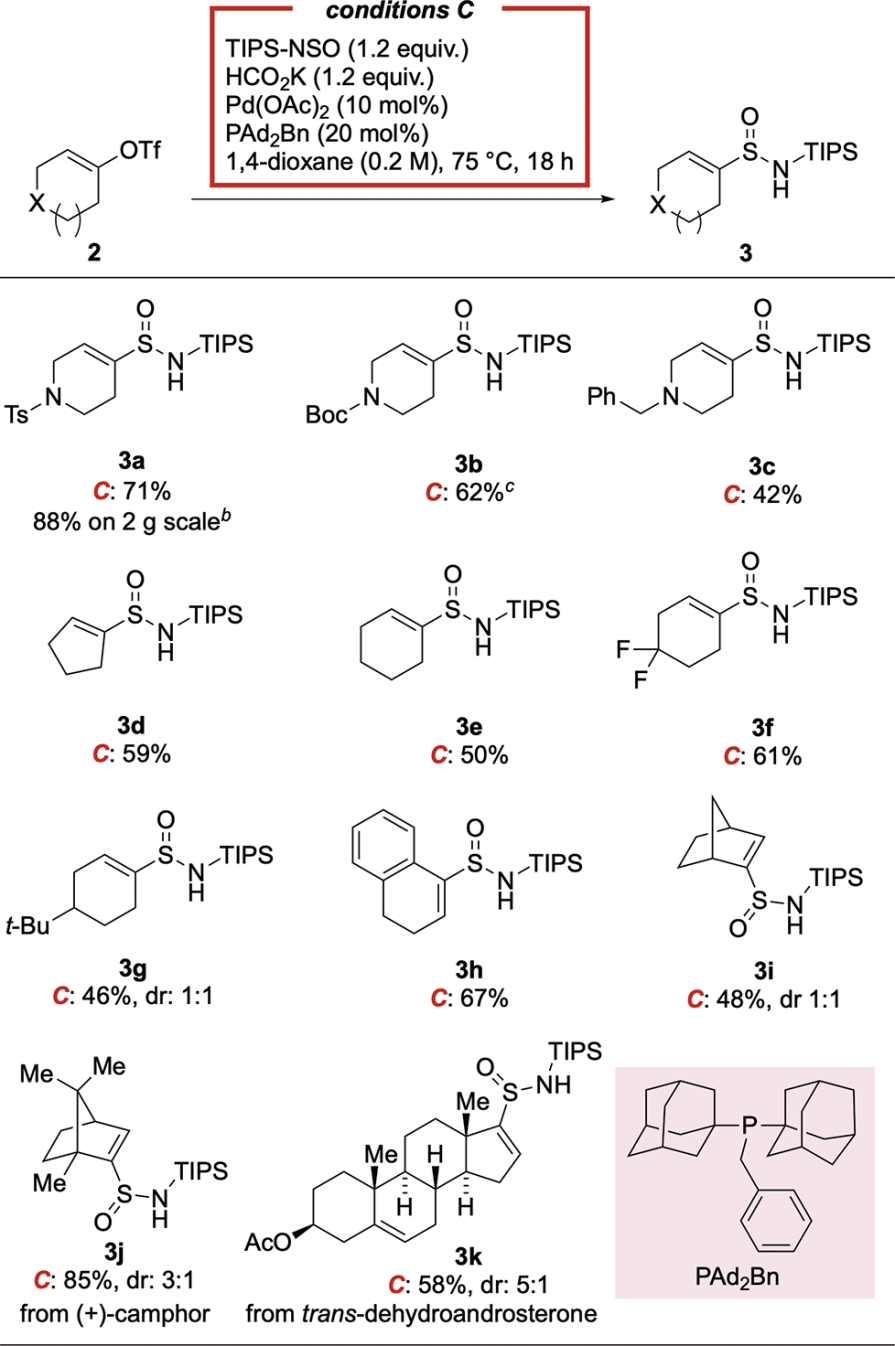
Scope of Sulfinamide Synthesis Using Alkenyl Triflate Substrates^a^

a Reaction conditions ***C***: alkenyl triflate 2 (0.20 mmol, 1.0 equiv), TIPS-NSO (1.2 equiv), HCO_2_K (1.2 equiv), Pd(OAc)_2_ (10 mol %), PAd_2_Bn (20 mol %), 1,4-dioxane (0.2 M), 75 °C, 18 h.

b At 5 mmol scale using 5 mol % Pd(OAc)_2_, 10 mol % PAd_2_Bn.

c 1.0 equiv HCO_2_K.

With success in preparing a broad range of functional aryl and alkenyl sulfinamides, we then chose aryl methyl ester **1f** and cyclic alkene **3a** to explore derivatization strategies (Scheme 3). The silyl group of aryl sulfinamide **1f** could be easily removed by treatment with TBAF, resulting in primary sulfinamide **4a** in excellent yield (99%). Treatment of sulfinamide **4a** with PhI(OAc)_2_, morpholine, and triethylamine led efficiently to sulfonimidamide **4b**.^15f^ Ammonia- and aniline-derived sulfonimidamides (**4c** and **4d**) were prepared using a chlorination/amination/deprotection sequence, in high yields (69% and 72%, respectively). Using sulfinamide **1f** in a deprotonation/oxidative fluorination process provided sulfonimidoyl fluoride **4e** in 90% yield. Primary sulfonamide **4f** was available using an oxidation-deprotection protocol (95%, two steps). Importantly, in all of these transformations of the sulfur-core, the integratory of the spectating methyl ester was uneffected. Alkenyl sulfinamide **3a** was also amenable to manipulation; primary sulfinamide **5a** was isolated from N-TIPS derivative **3a** in 85% yield, and could be smoothly converted into sulfonimidamide **5b** using an oxidative amination. Sulfonimidoyl fluoride **5c** was similarly available in high yield by using NFSI as the oxidant following deprotonation with NaH.

**Scheme 3:**
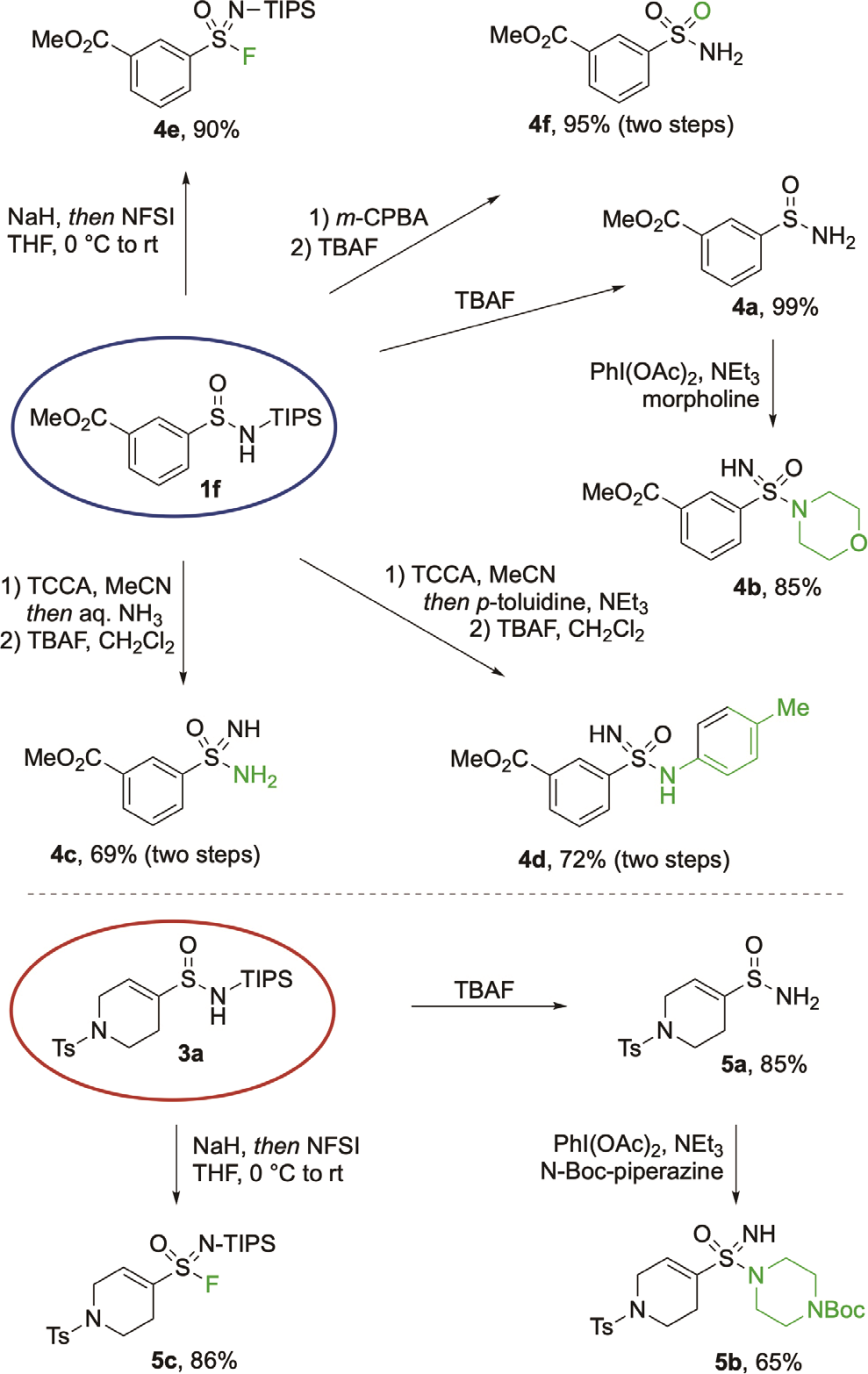
Derivatisation of Sulfinamides **1f** and **3a** into Diverse Sulfur Functional Groups

We have shown for the first time that aryl and heteroaryl halides are viable substrates for the catalytic synthesis of sulfinamides. This modular synthesis employs commercial catalyst components and a commercial sulfinylamine reagent and is achieved under mild conditions. The reaction can be performed at gram scale with a reduced catalyst loading. The sulfinamide products could be readily converted into high-value sulfur(VI) groups including sulfonamides, sulfonimidamides, and sulfonimidoyl fluorides. The wide substrate scope, good functional group tolerance, and the broad availability of aryl halides suggest that this transformation will find wide application in discovery chemistry.

## Supporting Information Available

Experimental procedures, spectral characterization (NMR spectra for all compounds), additional reaction optimization data, and a table of unsuccessful substrates (PDF)
