# Supplementary material for: Palladium-Catalyzed Addition of Aryl Halides to N-Sulfinylamines for the Synthesis of Sulfinamides
Source: J Am Chem Soc. 2024 Jul 12;146(29):19690–5. doi: 10.1021/jacs.4c06726 (PMC11273345; doi:10.1021/jacs.4c06726)
Supplement: Supplementary file 1 — ja4c06726_si_001.pdf [file ja4c06726_si_001.pdf]

Palladium-catalysed addition of aryl halides to *N*-sulfinylamines for the synthesis of sulfinamides

Ming-Kai Wei, [¶,a] Daniel F. Moseley, [¶,a] Robin M. Bär,[b] Yeshua Sempere,[b] Michael C. Willis[a*]

*michael.willis@chem.ox.ac.uk

[a]Department of chemistry, University of Oxford, Chemistry Research Laboratory, Mansfield Road, Oxford, OX1 3TA, UK

[b]Research & Development, Crop Science, Bayer AG, Alfred-Nobel-Str. 50, Monheim am Rhein, Germany

[¶]These authors contributed equally to this work

Supporting Information

Table of Contents

1. General information 3

1.1 General Considerations 3

1.2 Preparation of starting materials 5

2. Optimisation of the reaction conditions: 6

Table S1: *N*-Sulfinylamine screening 6

Table S2: Reductant screening 6

Table S3: Addition of phase transfer catalyst 7

Table S4: Temperature screening 7

Table S5: Solvent screening 8

Table S6: Catalyst screening 9

Table S7: Varying the loading of TIPS-NSO and HCO_2Cs 10

Table S8: Reaction concentration 10

Table S9: Reaction time 11

Table S10: Selected optimisation conditions for electron-rich aryl bromide 11

Table S11: Ligand screen for alkenyl triflate 12

Table S12: Reductant screen for alkenyl triflates 12

Table S13: Problematic substrates: 13

3. Experiment Procedures: 14

3.1 General procedures 14

3.2 Substrates scope 17

3.3 Derivatizations 55

4. References 67

5. NMR spectra 68

1. General information

1.1 General Considerations

Unless otherwise stated, all reactions were conducted under an atmosphere of nitrogen with anhydrous solvents using standard Schlenk techniques. Glassware was dried in an oven (> 100 °C) and allowed to cool to room temperature under a positive pressure of nitrogen before use. Cooling of reaction mixtures to 0 °C was achieved using an ice-water bath.

Unless otherwise stated, all chemicals were purchased from commercial sources (Sigma-Aldrich, Fluorochem, Fisher Scientific, Alfa-Aesar or Apollo Scientific) and were used without further purification. SPhos Pd G3 (CAS No.: 1445085-82-4) was purchased from Sigma-Aldrich and used as received. Cesium formate was purchased from Sigma-Aldrich or Thermo Scientific and used as received. Anhydrous solvents were purified by filtration through dried alumina columns using the University of Oxford internal solvent drying system (Innovative Technology Inc. PS-400-7) and sparged with nitrogen before use. 1,4-dioxane was obtained from the solvent drying system and stored under nitrogen with activated 4 Å molecular sieves. The solvent was degassed with nitrogen for 30 min before use. All inert gases were sourced from the University of Oxford internal supplies and dried through $CaCl_2$ drying columns. 'Petrol ether' refers to the fraction of petroleum ether which boils in the range 40-60 °C. 'Brine' refers to a saturated aqueous solution of sodium chloride.

Thin-layer chromatography (TLC) was performed on Merck silica gel 60 F_{254} pre-coated aluminium backed TLC sheets with a visualisation under a UV lamp (λ_{max} = 254 nm) and/or by staining with $KMnO_4$ solution. Flash column chromatography was performed using Merk silica gel 60 (230-400 mesh) with the solvent system indicated in parenthesis.

^{1}H NMR spectra were recorded on a Bruker AVIII HD 400 (400 MHz), AVII 500 (500 MHz), AVII HD 500 (500 HMz), NEO 600 (600 MHz) spectrometer using the residual solvent as an internal standard. ^{13}C NMR spectra (proton decoupled) were recorded on a Bruker AVIII HD 400 (101 MHz), AVII 500 (126 MHz), AVIII HD 500 (126 MHz) or NEO 600 (151 MHz) spectrometer at 101 MHz. ^{19}F NMR spectra were recorded on a Bruker AVIII400 spectrometer at 377 MHz. All reported ^{1}H and ^{13}C chemical shifts (δH, δC) are referenced to the residual signal of deuterated solvents ($CDCl_3$: δ_H = 7.26 ppm, δ_C = 77.16 ppm; ^{19}F chemical shifts (δF) are referenced externally to $CFCl_3$ (δ_F = 0.0 ppm). Chemical shifts (δ) are reported in parts per million (ppm) to the nearest 0.01 ppm for 1H NMR, and 0.1 ppm for ^{13}C and ^{19}F NMR. Coupling constants (*J*) are reported in Hertz (Hz) and rounded to the nearest 0.5 Hz.

Multiplicities are reported as followings: s (singlet), d (doublet), t (triplet), q (quartet), m (multiplet), br. (broad signal), app. (apparent).

Infrared spectra were recorded on a Bruker Tensor 27 Fourier Transform spectrometer with an internal range 600-4000 cm^{-1} and all absorption maximum (ν_{max}) are given in wavenumbers (cm^{-1}).

Melting points were recorded in degrees Celsius (°C) using a STUART scientific hotstage-microscope apparatus SMP1 or a Reichert melting point apparatus and are reported uncorrected.

Low resolution mass spectra (LRMS) were recorded on a Waters LCT Premier mass spectrometer. High resolution mass spectra (HRMS) were obtained on an ACQUITY I-Class PLUS UPLC system (Waters, Milford, MA, USA) coupled to an ACQUITY RDa mass spectrometer (Waters, Milford, MA, USA) equipped with an ESI probe, in positive ion mode with a TOF mass analyzer. Values quoted are a ratio of mass to charge in Daltons to four decimal places. The mass found was compared to the mass calculated from the monoisotopic molecular formula, and all results were found to be within a 5 ppm error of the calculated values. Samples for mass spectra were prepared in 1 mg/mL solution in MeCN or MeOH (LRMS, HRMS-ESI).

1.2 Preparation of starting materials

The *N*-sulfinylamines were prepared according to the literatures. [1-5]

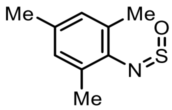

Mesityl-NSO, Ref. 1

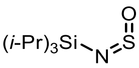

TIPS-NSO, Ref. 2,3

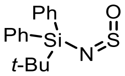

TBDPS-NSO, Ref. 2,3

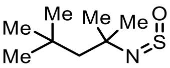

t-Oct-NSO, Ref. 4

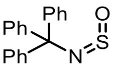

Tr-NSO, Ref. 5

The alkenyl triflates were prepared according to the literatures. [6-9]

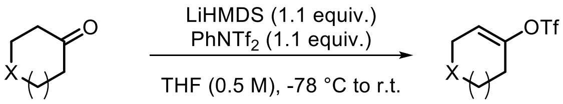

(2)

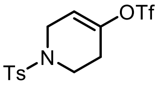

2a, Ref. 6

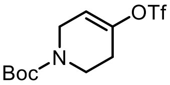

2b, Ref. 6

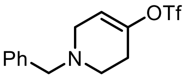

2c, Ref. 6

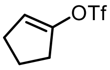

2d, Ref. 7

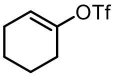

2e, Ref. 7

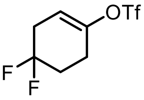

2f, Ref. 6

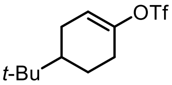

2g, Ref. 6

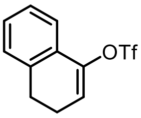

2h, Ref. 6

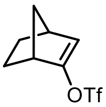

2i, Ref. 8

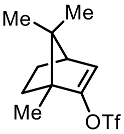

2j, Ref. 8

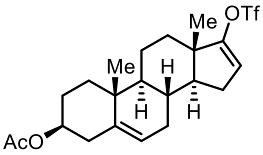

2k, Ref. 9

2. Optimisation of the reaction conditions:

Table S1: *N*-Sulfinylamine screening

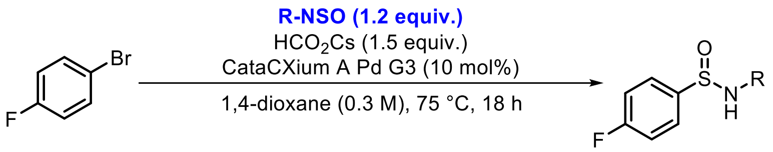

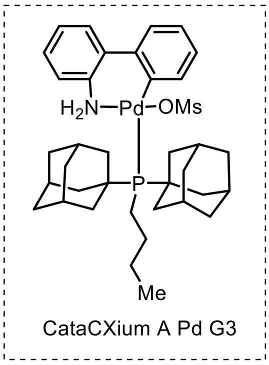

Entry	R-NSO	Yield (%)[a]
1	Trityl	15
2	*t*-Octyl	6
3	Mesityl	12
4	TBDPS	27
5	TIPS	61

[a] Yields determined by quantitative ^{19}F NMR spectroscopy of the crude reaction mixture using 1,4-difluorobenzene as the internal standard.

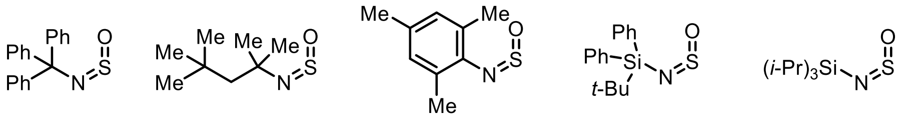

Tr-NSO   *t*-Oct-NSO   Mesityl-NSO   TBDPS-NSO   TIPS-NSO

Table S2: Reductant screening

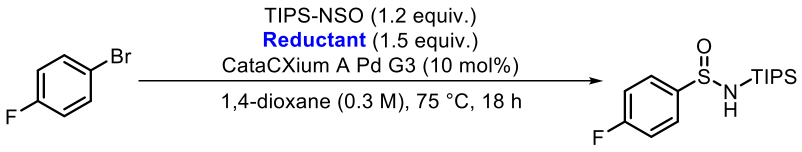

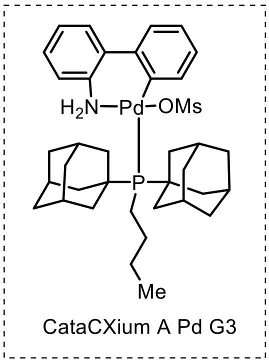

Entry	Reductant	Yield (%)[a]
1	Zn	0[b]
2	Mn	0[b]
3	HCO_2Na	21
4	HCO_2K	45
5	HCO_2Cs	61
6	HCO_2NH_4	trace
7	$Me(OMe)_2SiH$	0
8	$Me(OEt)_2SiH$	0

[a] Yields determined by quantitative ^{19}F NMR spectroscopy of the crude reaction mixture using 1,4-difluorobenzene as the internal standard.
[b] With TrNSO

Table S3: Addition of phase transfer catalyst

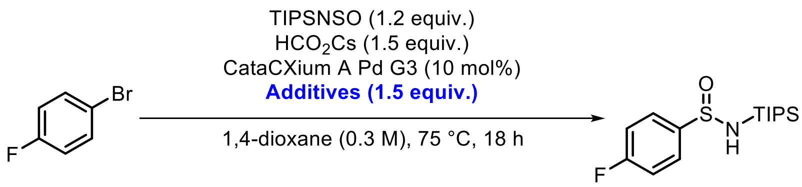

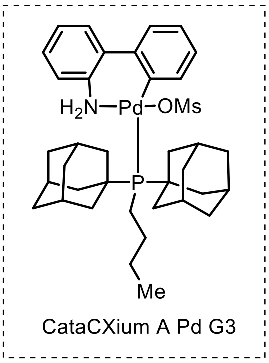

Entry	Additives	Yield (%)[a]
1	TBAB	0

[a] Yields determined by quantitative ^{19}F NMR spectroscopy of the crude reaction mixture using 1,4-difluorobenzene as the internal standard.

Table S4: Temperature screening

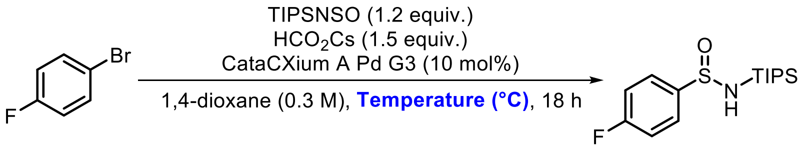

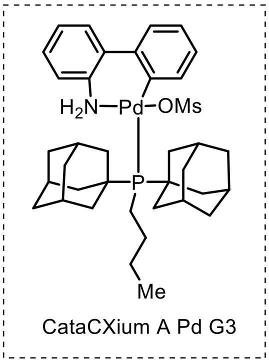

Entry	Temperature (°C)	Yield (%)[a]
1	65	2
2	75	61
3	85	42
4	95	32

[a] Yields determined by quantitative ^{19}F NMR spectroscopy of the crude reaction mixture using 1,4-difluorobenzene as the internal standard.

Table S5: Solvent screening

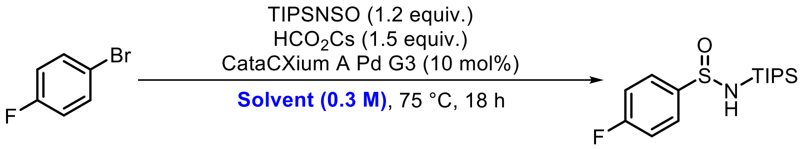

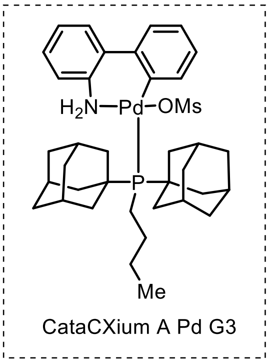

Entry	Solvent	Yield (%)[a]
1	1,4-dioxane	61
2	THF	23
3	CPME	23
4	MTBE	21
5	DMF	0
6	DMSO	0
7	MeCN	0
8	Toluene	0
9	DCE	32

[a] Yields determined by quantitative ^{19}F NMR spectroscopy of the crude reaction mixture using 1,4-difluorobenzene as the internal standard.

Table S6: Catalyst screening

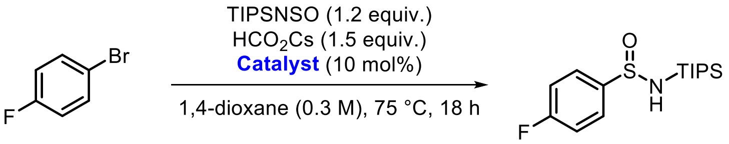

Entry	Catalyst	Yield (%)[a]
1	CataCXium A Pd G3	61
2	SPhos Pd G3	84
3	RuPhos Pd G3	70
4	XPhos Pd G3	77
5	APhos Pd G3	33
6	BrettPhos Pd G3	9
7	(t-Bu)$_3$P Pd G4	17
8	DPEPhosPdCl$_2$	0
9	Bis((o-Tol)$_3$P)PdCl$_2$	12

[a] Yields determined by quantitative ^{19}F NMR spectroscopy of the crude reaction mixture using 1,4-difluorobenzene as the internal standard.

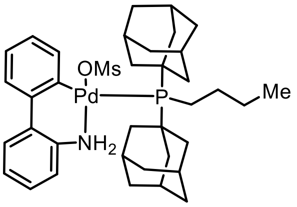

CataCXium A Pd G3

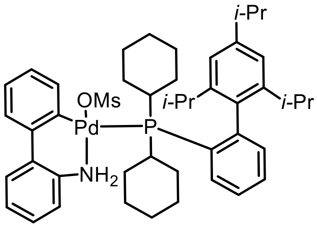

XPhos Pd G3

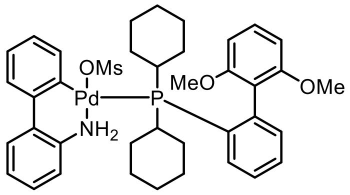

SPhos Pd G3

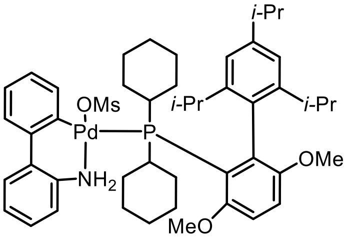

BrettPhos Pd G3

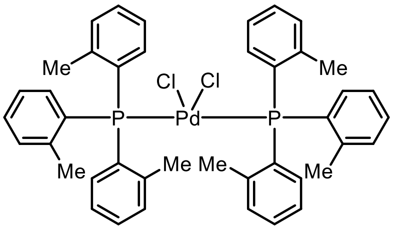

Bis((o-Tol)$_3$P)PdCl$_2$

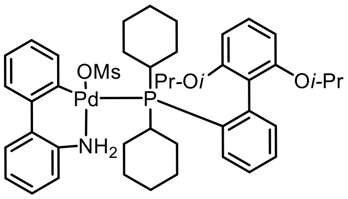

RuPhos Pd G3

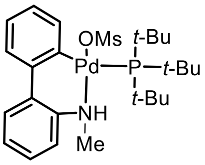

(t-Bu)$_3$P Pd G4

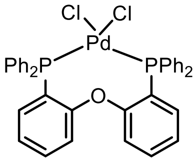

DPEPhosPdCl$_2$

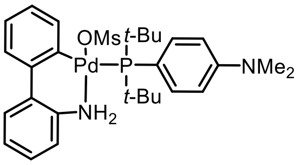

APhos Pd G3

Table S7: Varying the loading of TIPS-NSO and HCO_2Cs

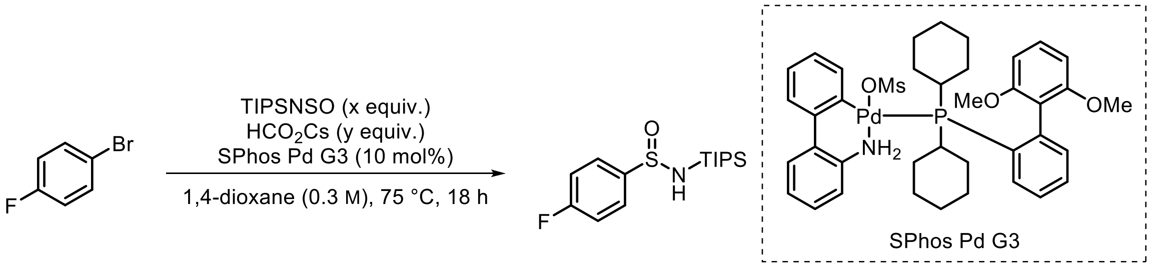

Entry	TIPS-NSO loading (equiv.)	HCO_2Cs loading (equiv.)	Yield (%)[a]
1	1.2	1.5	84
2	1.2	2.0	71
3	1.2	3.0	10
4	1.5	1.5	52
5	1.5	2.0	8
6	1.5	3.0	2

[a] Yields determined by quantitative ^{19}F NMR spectroscopy of the crude reaction mixture using 1,4-difluorobenzene as the internal standard.

Table S8: Reaction concentration

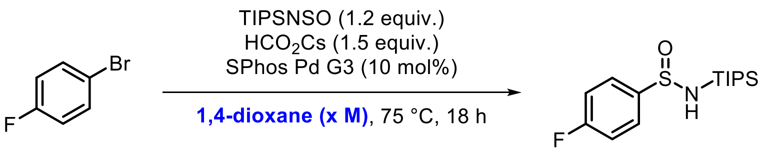

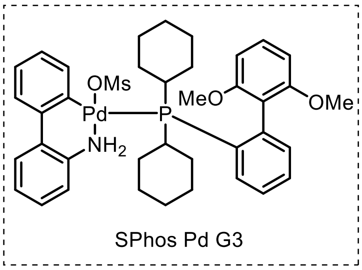

Entry	Aryl bromide concentration (M)	Yield (%)[a]
1	0.1	75
2	0.2	86
3	0.3	84
4	0.4	81

[a] Yields determined by quantitative ^{19}F NMR spectroscopy of the crude reaction mixture using 1,4-difluorobenzene as the internal standard.

Table S9: Reaction time

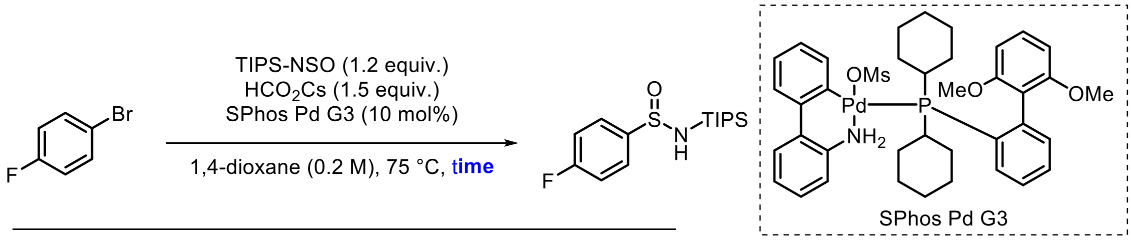

Entry	time	Yield (%)[a]
1	1 h	28
2	4 h	70
3	7 h	75
4	18 h	86

[a] Yields determined by quantitative ^{19}F NMR spectroscopy of the crude reaction mixture using 1,4-difluorobenzene as the internal standard.

Table S10: Selected optimisation conditions for electron-rich aryl bromide

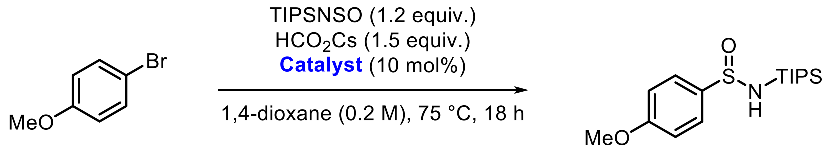

Entry	Catalyst	Yield (%)[a]
1	SPhos Pd G3	37
2	CataCXium A Pd G3	52
3	10 mol% $Pd(OAc)_2$, 20 mol% CataCXium A	54
4	10 mol% $Pd(OAc)_2$, 20 mol% CataCXium A	39[b]
5	10 mol% $Pd(OAc)_2$, 20 mol% PAd_2Bn	56
6	10 mol% $Pd(OAc)_2$, 20 mol% PAd_2Bn	61[b]

[a] Yields determined by quantitative ^{1}H NMR spectroscopy of the crude reaction mixture using dibromomethane as the internal standard.
[b] 1.2 equiv HCO_2Cs

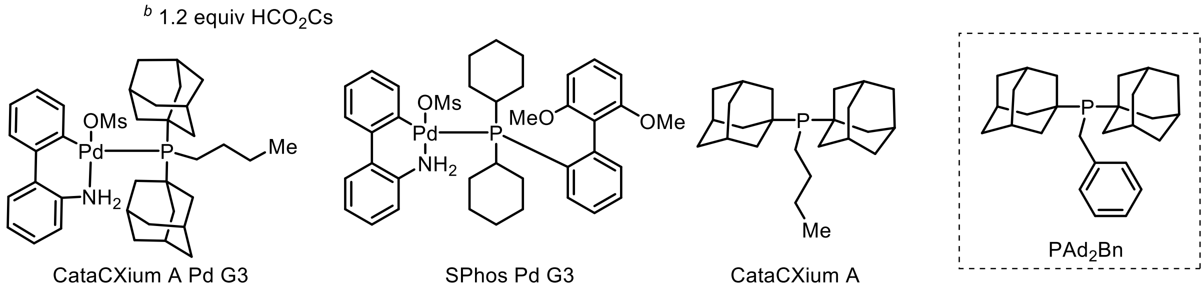

Table S11: Ligand screen for alkenyl triflate

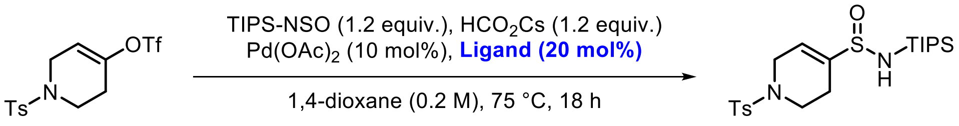

Entry	Ligand	Yield (%)[a]
1	PAd_2Bn	52% (44%[b])
2	CataCXium A	44%
3	APhos	10%
4	10 mol% SPhos Pd G3 (without $Pd(OAc)_2$)	38%

[a] Yields determined by quantitative ^{1}H NMR spectroscopy of the crude reaction mixture using dibromomethane as the internal standard.
[b] Alkenyl Bromide was used

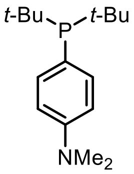

APhos

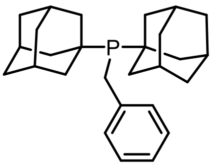

PAd_2Bn

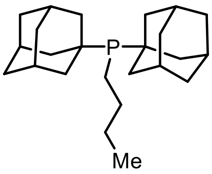

CataCXium A

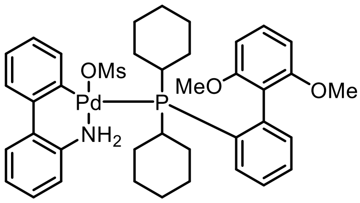

SPhos Pd G3

Table S12: Reductant screen for alkenyl triflates

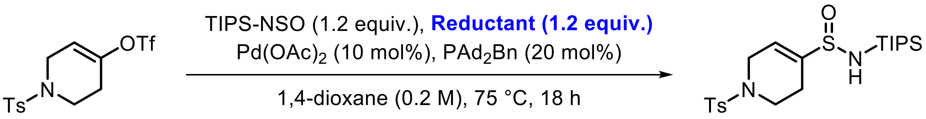

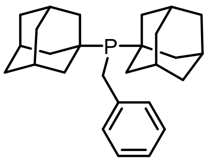

PAd_2Bn

Entry	Reductant	Yield (%)[a]
1	HCO_2Cs	52%
2	HCO_2Na	30%
3	HCO_2K	74%
4	$(HCO_2)_2Ca$ (0.6 equiv.)	0%

[a] Yields determined by quantitative ^{1}H NMR spectroscopy of the crude reaction mixture using dibromomethane as the internal standard.

Table S13: Problematic substrates:

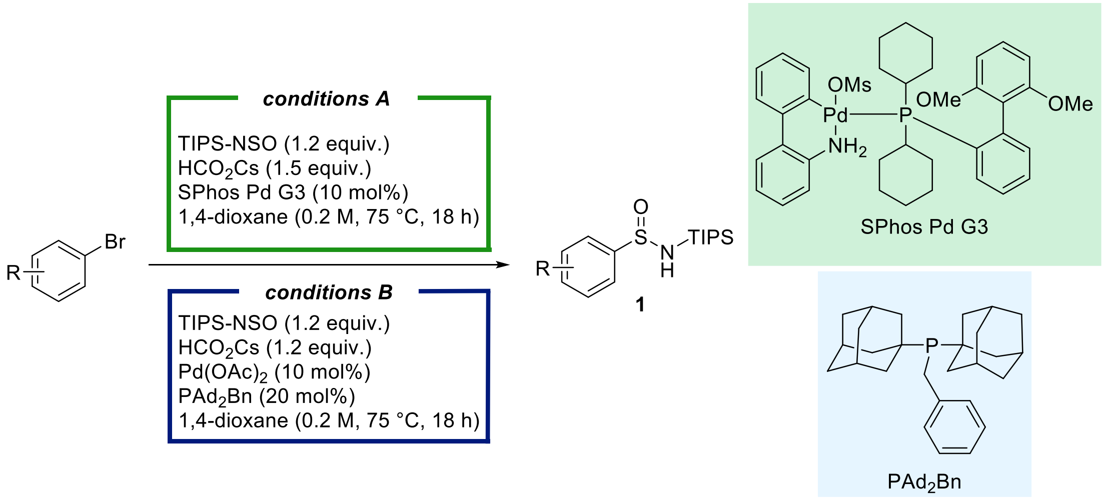

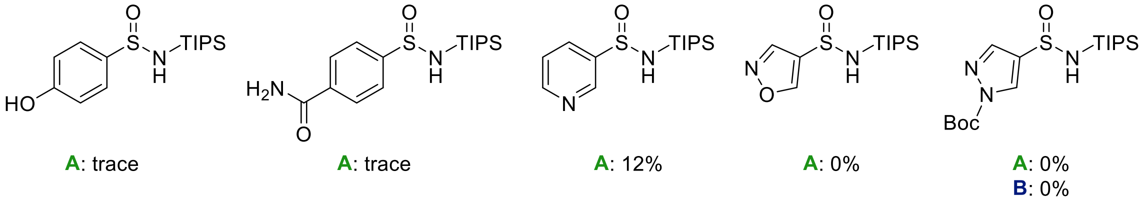

3. Experiment Procedures:

3.1 General procedures

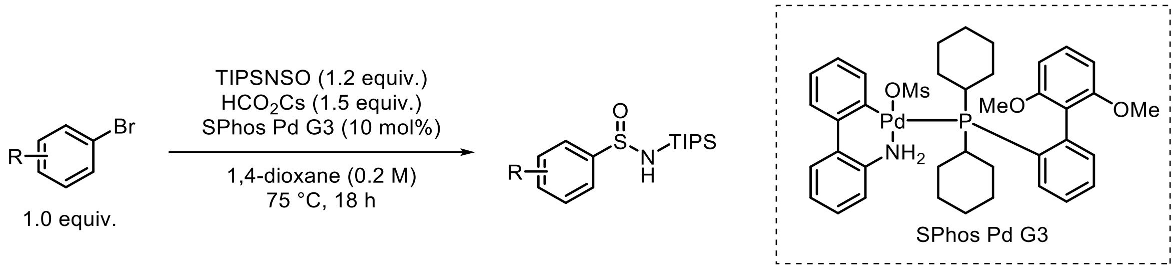

General Procedure A: An oven-dried microwave vial containing a magnetic stirring bar was charged with SPhos Pd G3 (10 mol%), aryl bromide (if it is a solid) (1.0 equiv.) and HCO_2Cs (1.5 equiv.). The vial was then sealed with a microwave cap and subjected to three evacuation/argon refill cycles. Aryl bromide (1.0 equiv.) was added (if liquid), followed by TIPS-NSO (1.2 equiv.) and anhydrous 1,4-dioxane (0.2 M). The vial was then suspended in a 75 °C oil bath with vigorous stirring (500-800 rpm) for 18 h. The reaction vial was cooled to room temperature, the contents diluted with EtOAc (5 mL for 0.2 mmol scale), and filtered through a pad of silica (1.5 to 2 cm for 0.2 mmol) washing with EtOAc (40 mL for 0.2 mmol). The resultant crude solution was concentrated *in vacuo* and purified by column chromatography to afford the desired product.

Notes (for all the general procedures):

1. The formate salt (HCO_2Cs) salt is hydroscopic, but the reaction was always performed outside the glovebox and the yield was reproducible. We recommend weighing the formate salt, making sure it does not stick on the sides of the reaction tube. The tube should be sealed as soon as the formate salt is added. We also recommend storing the formate salt under N_2 after use.
2. Cesium formate can be dried by heating at 150 ˚C under high-vacuum overnight. Finely grinding the formate helps with the reproducibility of the reaction.
3. Because the reaction is heterogenous, stirring is very important to reaction.

4. An oil bath was used for heating the reaction.
5. The quality of TIPS-NSO is also important, and any impurity such as TIPS-NH_2 or TIPSOH will lower the reaction yield.

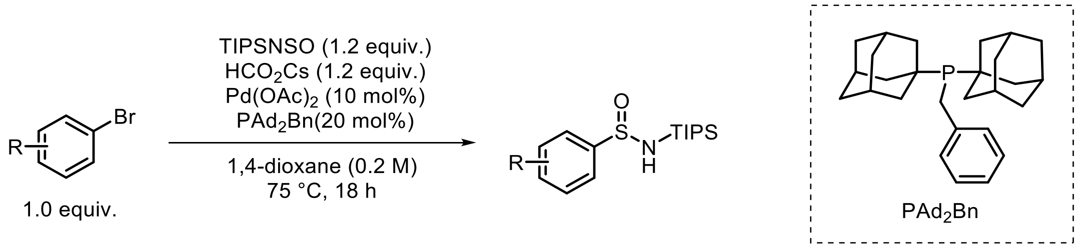

General Procedure B: An oven-dried microwave vial containing a magnetic stirring bar was charged with $Pd(OAc)_2$ (10 mol%), di(1-adamantyl)benzyl phosphine (20 mol%), aryl bromide (if it is a solid) (1.0 equiv.) and HCO_2Cs (1.2 equiv.). The vial was then sealed with a microwave cap and subjected to three evacuation/argon refill cycles. Aryl bromide (1.0 equiv.) was added (if liquid), followed by TIPS-NSO (1.2 equiv.) and anhydrous 1,4-dioxane (0.2 M). The vial was then suspended in a 75 °C oil bath with vigorous stirring (500-800 rpm) for 18 h. The reaction vial was cooled to room temperature, the contents diluted with EtOAc (5 mL for 0.2 mmol scale), and filtered through a pad of silica (1.5 to 2 cm for 0.2 mmol scale) washing with EtOAc (40 mL for 0.2 mmol scale). The resultant crude solution was concentrated *in vacuo* and purified by column chromatography to afford the desired product.

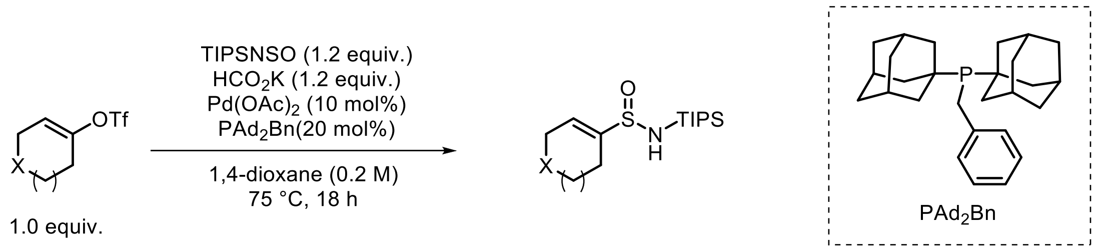

General Procedure C: An oven-dried microwave vial containing a magnetic stirring bar was charged with $Pd(OAc)_2$ (10 mol%), di(1-adamantyl)benzyl phosphine (20 mol%), alkenyl triflate (if it is a solid) (1.0 equiv.) and HCO_2K (1.2 equiv.). The vial was then sealed with a microwave cap and subjected to three evacuation/argon refill cycles. Alkenyl triflate (1.0 equiv.) was added (if liquid), followed by TIPS-NSO (1.2 equiv.) and anhydrous 1,4-dioxane (0.2 M). The vial was then suspended in a 75 °C oil bath with vigorous stirring (500-800 rpm) for 18 h. The reaction vial was cooled to room temperature, the contents diluted with EtOAc (5 mL for 0.2 mmol scale), and filtered through a pad of silica (1.5 to 2 cm for 0.2 mmol scale) washing with EtOAc (40 mL for 0.2 mmol scale). The resultant crude solution was concentrated *in vacuo* and purified by column chromatography to afford the desired product.

3.2 Substrates scope

4-Fluoro-*N*-(triisopropylsilyl)benzenesulfinamide (1a)

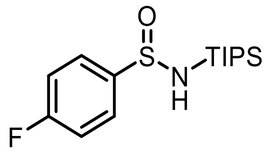

Synthesised according to **general procedure A**, using SPhos Pd G3 (15.6 mg, 0.02 mmol, 10 mol%), HCO_2Cs (53.4 mg, 0.30 mmol, 1.50 equiv.), 1-bromo-4-fluorobenzene (35.0 mg, 0.20 mmol, 1.00 equiv.), TIPS-NSO (52.7 mg, 0.24 mmol, 1.20 equiv.) and 1,4-dioxane (1.0 mL). Purification of the crude residue using column chromatography (10:1 to 5:1 petrol ether: ethyl acetate) yielded title compound **1a** (53.6 mg, 85%) as an off-white solid.

^{1}H NMR (500 MHz, $CDCl_3$) 7.72 (dd, J = 8.6, 5.3 Hz, 2H), 7.17 (app t, J = 8.6 Hz, 2H), 3.67 (bs, 1H), 1.35 – 1.19 (m, 3H), 1.17 – 1.11 (m, 18H);

^{13}C NMR (126 MHz, $CDCl_3$) 164.3 (d, J = 250.9 Hz), 145.9 (d, J = 3.0 Hz), 127.4 (d, J = 8.8 Hz), 116.2 (d, J = 22.3 Hz), 18.1, 18.0, 11.9. (Note: For dimethyl carbons in tri-*iso*propylsilyl group, $NSi(CH(CH_3)_2)_3$, 2 peaks were found instead of 1 due to the loss of symmetry caused by chiral sulfur atom.)

^{19}F NMR (377 MHz, $CDCl_3$) δ -109.82 (s).

M.P.: 66–68 °C (Et_2O);

IR (ATR) (ν_{max} cm $^{-1}$) =3115, 2946, 2869, 1589, 1491, 1466, 1230, 1085, 1061, 883, 741, 679.

HRMS (ESI) calcd. for $C_{15}H_{27}FNOSSi^+$ $[M+H]^+$: 316.1561; found: 316.1547.

4-Chloro-*N*-(triisopropylsilyl)benzenesulfinamide (1b)

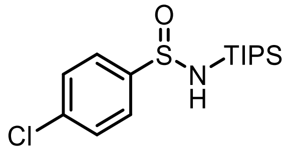

Synthesised according to **general procedure A**, using SPhos Pd G3 (15.6 mg, 0.02 mmol, 10 mol%), HCO_2Cs (53.4 mg, 0.30 mmol, 1.50 equiv.), 1-bromo-4-chlorobenzene (38.3 mg, 0.20 mmol, 1.00 equiv.), TIPS-NSO (52.7 mg, 0.24 mmol, 1.20 equiv.) and 1,4-dioxane (1.0 mL). Purification of the crude residue using column chromatography (10:1 to 5:1 petrol ether: ethyl acetate) yielded title compound **1b** (53.2 mg, 80%) as an off-white solid.

^{1}H NMR (400 MHz, $CDCl_3$) δ 7.66 (d, *J* = 8.6 Hz, 2H), 7.45 (d, *J* = 8.6 Hz, 2H), 3.70 (s, 1H), 1.30 – 1.20 (m, 3H), 1.13 (dd, *J* = 7.3, 5.6 Hz, 18H).

^{13}C NMR (101 MHz, $CDCl_3$) δ 148.7, 137.1, 129.3, 126.6, 18.1, 17.9, 11.9. (Note: For dimethyl carbons in tri-*iso*propylsilyl group, $NSi(CH(CH_3)_2)_3$, 2 peaks were found instead of 1 due to the loss of symmetry caused by chiral sulfur atom.)

IR (ATR) (ν_{max} cm $^{-1}$) = 3062, 1471, 1092, 880, 830, 675.

M.P. :80-81 ˚C (CH_2Cl_2)

HRMS (ESI) calcd. for $C_{15}H_{27}ClNOSSi^+$ $[M+H]^+$:332.1266; found: 332.1263.

4-Nitro-*N*-(triisopropylsilyl)benzenesulfinamide (1c)

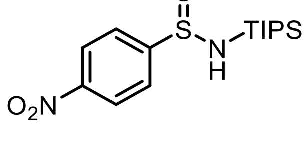

Synthesised according to **general procedure A**, using SPhos Pd G3 (15.6 mg, 0.02 mmol, 10 mol%), HCO_2Cs (53.4 mg, 0.30 mmol, 1.50 equiv.), 1-bromo-4-nitrobenzene (40.4 mg, 0.20

mmol, 1.00 equiv.), TIPS-NSO (52.7 mg, 0.24 mmol, 1.20 equiv.) and 1,4-dioxane (1.0 mL). Purification of the crude residue using column chromatography (10:1 to 5:1 petrol ether: ethyl acetate) yielded title compound **1c** (41.7 mg, 61%) as an off-white solid.

^{1}H NMR (400 MHz, $CDCl_3$) δ 8.33 (d, *J* = 8.7 Hz, 2H), 7.91 (d, *J* = 8.7 Hz, 2H), 3.79 (s, 1H), 1.33 – 1.23 (m, 3H), 1.15 (dd, *J* = 7.3, 4.7 Hz, 18H).

^{13}C NMR (101 MHz, $CDCl_3$) δ 156.7, 149.4, 126.4, 124.3, 18.0, 17.9, 11.9. (Note: For dimethyl carbons in tri-*iso*propylsilyl group, NSi(CH(CH_3)$_2$)$_3$, 2 peaks were found instead of 1 due to the loss of symmetry caused by chiral sulfur atom.)

IR (ATR) (ν_{max} cm $^{-1}$) = 1595, 1520, 1469, 1345, 1250, 1079, 1047, 878, 853.

M.P. : 120-122 ˚C (CH_2Cl_2)

HRMS (ESI) calcd. for $C_{15}H_{26}N_2O_3SSi^+$ $[M+H]^+$: 343.1506; found: 343.1507.

4-Cyano-*N*-(triisopropylsilyl)benzenesulfinamide (1d)

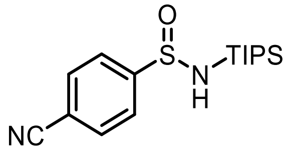

Synthesised according to **general procedure A**, using SPhos Pd G3 (15.6 mg, 0.02 mmol, 10 mol%), HCO_2Cs (53.4 mg, 0.30 mmol, 1.50 equiv.), 4-bromobenzonitrile (36.4 mg, 0.20 mmol, 1.00 equiv.), TIPS-NSO (52.7 mg, 0.24 mmol, 1.20 equiv.) and 1,4-dioxane (1.0 mL). Purification of the crude residue using column chromatography (10:1 to 5:1 to 4:1 petrol ether: ethyl acetate) yielded title compound **1d** (36.8 mg, 57%) as an off-white solid.

^{1}H NMR (400 MHz, $CDCl_3$) δ 7.87 – 7.83 (m, 2H), 7.80 – 7.75 (m, 2H), 3.77 (s, 1H), 1.31 – 1.21 (m, 3H), 1.13 (dd, *J* = 7.3, 5.3 Hz, 18H).

^{13}C NMR (101 MHz, $CDCl_3$) δ 155.0, 132.8, 126.0, 118.0, 114.6, 18.0, 17.9, 11.9. (Note: For dimethyl carbons in tri-*iso*propylsilyl group, $NSi(CH(CH_3)_2)_3$, 2 peaks were found instead of 1 due to the loss of symmetry caused by chiral sulfur atom.)

IR (ATR) (ν_{max} cm $^{-1}$) = 3267, 1464, 1391, 1083, 869, 763.

M.P. : 100-102 ˚C (CH_2Cl_2)

HRMS (ESI) calcd. for $C_{16}H_{27}N_2OSSi^+$ $[M+H]^+$: 323.1608; found: 323.1601.

4-Acetyl-*N*-(triisopropylsilyl)benzenesulfinamide (1e)

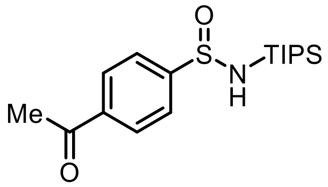

Synthesised according to **general procedure A**, using SPhos Pd G3 (15.6 mg, 0.02 mmol, 10 mol%), HCO_2Cs (53.4 mg, 0.30 mmol, 1.50 equiv.), 1-(4-bromophenyl)ethan-1-one (39.8 mg, 0.20 mmol, 1.00 equiv.), TIPS-NSO (52.7 mg, 0.24 mmol, 1.20 equiv.) and 1,4-dioxane (1.0 mL). Purification of the crude residue using column chromatography (5:1 to 1:1 petrol ether: ethyl acetate) yielded title compound **1e** (44.5 mg, 66%) as an off-white solid.

^{1}H NMR (400 MHz, $CDCl_3$) 8.06 (d, J = 8.0 Hz, 2H), 7.83 (d, J = 8.1 Hz, 2H), 3.74 (bs, 1H), 2.63 (s, 3H), 1.27 (ddt, J = 13.4, 8.6, 6.5 Hz, 3H), 1.14 (dd, J = 7.3, 5.0 Hz, 18H);

^{13}C NMR (101 MHz, $CDCl_3$) 197.4, 154.8, 138.9, 129.0, 125.5, 27.0, 18.1, 17.9, 11.9; (Note: For dimethyl carbons in tri-*iso*propylsilyl group, $NSi(CH(CH_3)_2)_3$, 2 peaks were found instead of 1 due to the loss of symmetry caused by chiral sulfur atom.)

M.P. 117–119 °C (Et_2O);

IR (ATR) (ν_{max} cm $^{-1}$) = 3183, 2962, 2943, 2867, 1683, 1270, 1090, 1067, 903, 885;

HRMS (ESI) calcd. for $C_{17}H_{30}NO_2SSi^+$ $[M+H]^+$: 340.1761; found: 340.1751.

Methyl 3-(((triisopropylsilyl)amino)sulfinyl)benzoate (1f)

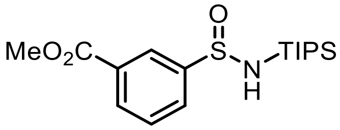

Synthesised according to **general procedure A**, using SPhos Pd G3 (15.6 mg, 0.02 mmol, 10 mol%), HCO_2Cs (53.4 mg, 0.30 mmol, 1.50 equiv.), methyl 3-bromobenzoate (43.0 mg, 0.20 mmol, 1.00 equiv.), TIPS-NSO (52.7 mg, 0.24 mmol, 1.20 equiv.) and 1,4-dioxane (1.0 mL). Purification of the crude residue using column chromatography (10:1 to 5:1 to 4:1 petrol ether: ethyl acetate) yielded title compound **1f** (62.5 mg, 88%) as an off-white solid.

Also prepared on a 1mmol scale: An oven-dried microwave vial containing a magnetic stirring bar was charged with SPhos Pd G3 (78.0 mg, 0.10 mmol, 10 mol%), methyl 3-bromobenzoate (215.1 mg, 1.00 mmol, 1.00 equiv.) and HCO_2Cs (266.9 mg, 1.50 mmol, 1.50 equiv.). The vial was then sealed with a microwave cap and subjected to three evacuation/argon refill cycles. TIPS-NSO (263.3 mg, 1.20 mmol, 1.20 equiv.) and anhydrous 1,4-dioxane (5.0 mL, 0.2 M) was then added. The resulting mixture was then put into a 75 °C oil bath with vigorous stirring (500-800 rpm) for 18 h. The resulting mixture was cooled to room temperature, diluted with EtOAc (10 mL), and filtered through a pad of silica (3 cm) washing with EtOAc (60 mL). The resultant crude solution was concentrated in *vacuo* and purified by column chromatography (10:1 to 3:1 petrol ether: ethyl acetate) to afford the desired product **1f** as an off white solid (278.0 mg, 78%).

1mmol scale with reduced Pd loading (5 mol%): An oven-dried microwave vial containing a magnetic stirring bar was charged with SPhos Pd G3 (39.0 mg, 0.05 mmol, 5 mol%), methyl 3-bromobenzoate (215.1 mg, 1.00 mmol, 1.00 equiv.) and HCO_2Cs (266.9 mg, 1.50 mmol,

1.50 equiv.). The vial was then sealed with a microwave cap and subjected to three evacuation/argon refill cycles. TIPS-NSO (263.3 mg, 1.20 mmol, 1.20 equiv.) and anhydrous 1,4-dioxane (5.0 mL, 0.2 M) was then added. The resulting mixture was then put into a 75 °C oil bath with vigorous stirring (500-800 rpm) for 18 h. The resulting mixture was cooled to room temperature, diluted with EtOAc (10 mL), and filtered through a pad of silica (3 cm) washing with EtOAc (60 mL). The resultant crude solution was concentrated in *vacuo* and purified by column chromatography (10:1 to 3:1 petrol ether: ethyl acetate) to afford the desired product **1f** as an off white solid (246.7 mg, 69%).

^{1}H NMR (400 MHz, $CDCl_3$) δ 8.38 (t, *J* = 1.7 Hz, 1H), 8.11 (dt, *J* = 7.7, 1.4 Hz, 1H), 7.92 (ddd, *J* = 7.8, 1.9, 1.2 Hz, 1H), 7.56 (t, *J* = 7.8 Hz, 1H), 3.91 (s, 3H), 3.76 (s, 1H), 1.32 – 1.21 (m, 3H), 1.14 (dd, *J* = 7.3, 5.7 Hz, 18H).

^{13}C NMR (101 MHz, $CDCl_3$) δ 166.1, 150.9, 131.8, 131.2, 129.5, 129.2, 126.4, 52.5, 18.0, 17.9, 11.9. (Note: For dimethyl carbons in tri-*iso*propylsilyl group, $NSi(CH(CH_3)_2)_3$, 2 peaks were found instead of 1 due to the loss of symmetry caused by chiral sulfur atom.)

M.P. : 70-72 ˚C (CH_2Cl_2)

HRMS (ESI) calcd. for $C_{17}H_{30}NO_3SSi^+$ $[M+H]^+$: 356.1710; found: 356.1709.

IR (ATR) (ν_{max} cm $^{-1}$) = 3064, 1722, 1464, 1436, 1296, 1264, 1124, 1060, 899, 880.

3-(Trifluoromethyl)-*N*-(triisopropylsilyl)benzenesulfinamide (1g)

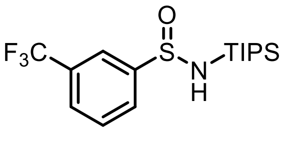

Synthesised according to **general procedure A**, using SPhos Pd G3 (15.6 mg, 0.02 mmol, 10 mol%), HCO_2Cs (53. 4 mg, 0.30 mmol, 1.50 equiv.), 1-bromo-3-(trifluoromethyl)benzene

(27.9 μL, 0.20 mmol, 1.00 equiv.), TIPS-NSO (52.7 mg, 0.24 mmol, 1.20 equiv.) and 1,4-dioxane (1.0 mL). Purification of the crude residue using column chromatography (10:1 to 5:1 to 4:1 petrol ether: ethyl acetate) yielded title compound **1g** (63.0 mg, 86%) as a white solid.

^{1}H NMR (400 MHz, $CDCl_3$) δ 8.02 (tt, *J* = 1.8, 0.8 Hz, 1H), 7.92 (dt, *J* = 7.8, 1.5 Hz, 1H), 7.72 (ddt, *J* = 7.7, 1.8, 1.0 Hz, 1H), 7.63 (t, *J* = 7.8 Hz, 1H), 3.79 (s, 1H), 1.27 (ddt, *J* = 13.7, 8.7, 6.5 Hz, 3H), 1.15 (dd, *J* = 7.3, 5.4 Hz, 18H).

^{13}C NMR (151 MHz, $CDCl_3$) δ 151.5, 131.6 (q, *J* = 33.0 Hz), 129.7, 128.7, 127.6 (q, *J* = 3.7 Hz), 123.7 (q, *J* = 272.6 Hz), 122.4 (q, *J* = 3.8 Hz), 18.0, 17.9, 11.9. (Note: For dimethyl carbons in tri-*iso*propylsilyl group, NSi(CH(CH_3)$_2$)$_3$, 2 peaks were found instead of 1 due to the loss of symmetry caused by chiral sulfur atom.)

^{19}F NMR (377 MHz, $CDCl_3$) δ -62.80 (s).

M.P. : 90-92 °C (CH_2Cl_2)

HRMS (ESI) calcd. for $C_{16}H_{27}F_3NOSSi^+$ $[M+H]^+$: 366.1529; found: 366.1542.

IR (ATR) (ν_{max} cm $^{-1}$) =3366, 1692, 1604, 1465, 1386, 1323, 1167, 1098, 905.

N-(Triisopropylsilyl)naphthalene-2-sulfinamide (1h)

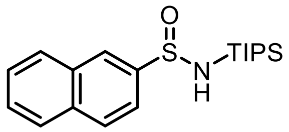

Synthesised according to **general procedure A**, using SPhos Pd G3 (15.6 mg, 0.02 mmol, 10 mol%), HCO_2Cs (53.4 mg, 0.30 mmol, 1.50 equiv.), 2-bromonaphthalene (41.4 mg, 0.20 mmol, 1.00 equiv.), TIPS-NSO (52.7 mg, 0.24 mmol, 1.20 equiv.) and 1,4-dioxane (1.0 mL).

Purification of the crude residue using column chromatography (9:1 to 4:1 petrol ether: ethyl acetate) yielded title compound **1h** (47.0 mg, 68%) as an off-white solid.

^{1}H NMR (400 MHz, $CDCl_3$) 8.32 (d, *J* = 1.8 Hz, 1H), 7.98 – 7.92 (m, 2H), 7.92 – 7.87 (m, 1H), 7.69 (dd, *J* = 8.6, 1.8 Hz, 1H), 7.61 – 7.53 (m, 2H), 3.74 (s, 1H), 1.30 (ddt, *J* = 13.6, 8.8, 6.6 Hz, 3H), 1.17 (dd, *J* = 7.3, 4.2 Hz, 18H);

^{13}C NMR (101 MHz, $CDCl_3$) 147.2, 134.4, 132.9, 129.2, 129.0, 128.0, 127.8, 127.2, 125.1, 121.6, 18.1, 18.0, 12.0; (Note: For dimethyl carbons in tri-*iso*propylsilyl group, $NSi(CH(\mathit{CH_3})_2)_3$, 2 peaks were found instead of 1 due to the loss of symmetry caused by chiral sulfur atom.)

M.P. (Et_2O) 112–114 °C;

IR (ATR) (ν_{max} cm $^{-1}$) = 3164, 3056, 2946, 2867, 1465, 1077, 1057, 908, 882, 757, 740, 678;

HRMS (ESI) calcd. for $C_{19}H_{30}NOSSi^+$ $[M+H]^+$: 348.1812; found: 348.1807.

Tert-butyl (2-cyano-4-(((triisopropylsilyl)amino)sulfinyl)phenyl)carbamate (1i)

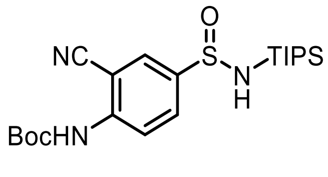

Synthesised according to **general procedure A**, using SPhos Pd G3 (15.6 mg, 0.02 mmol, 10 mol%), HCO_2Cs (53.4 mg, 0.30 mmol, 1.50 equiv.), tert-butyl (4-bromo-2-cyanophenyl)carbamate (59.4 mg, 0.20 mmol, 1.00 equiv.), TIPS-NSO (52.7 mg, 0.24 mmol, 1.20 equiv.) and 1,4-dioxane (1.0 mL). Purification of the crude residue using column chromatography (10:1 to 5:1 to 3:1 petrol ether: ethyl acetate) yielded title compound **1i** (76.2 mg, 87%) as an off-white solid.

^{1}H NMR (400 MHz, $CDCl_3$) δ 8.39 (d, *J* = 9.0 Hz, 1H), 7.90 (d, *J* = 2.1 Hz, 1H), 7.80 (dd, *J* = 9.0, 2.2 Hz, 1H), 7.23 (s, 1H), 3.77 (s, 1H), 1.53 (s, 9H), 1.24 (ddt, *J* = 13.4, 8.7, 6.5 Hz, 3H), 1.12 (dd, *J* = 7.3, 5.8 Hz, 18H).

^{13}C NMR (101 MHz, $CDCl_3$) δ 151.6, 144.2, 143.5, 131.1, 129.5, 119.4, 115.7, 100.9, 82.7, 28.2, 18.0, 17.9, 11.8. (Note: For dimethyl carbons in tri-*iso*propylsilyl group, $NSi(CH(CH_3)_2)_3$, 2 peaks were found instead of 1 due to the loss of symmetry caused by chiral sulfur atom.)

IR (ATR) (ν_{max} cm $^{-1}$) = 2933, 2868, 1743, 1578, 1520, 1366, 1300, 1231, 1155, 1058, 899, 881.

M.P. : 118-120°C (CH_2Cl_2)

HRMS (ESI) calcd. for $C_{21}H_{36}N_3O_3SSi^+$ $[M+H]^+$: 438.2241; found: 438.2249.

4-Methoxy-*N*-(triisopropylsilyl)benzenesulfinamide (1j)

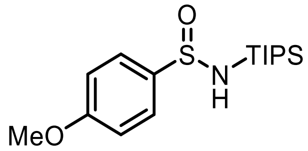

Synthesised according to **general procedure B**, using $Pd(OAc)_2$ (4.5 mg, 0.02 mmol, 10 mol%), PAd_2Bn (15.7 mg, 0.04 mmol. 20 mol%), HCO_2Cs (42.7 mg, 0.24 mmol, 1.20 equiv.), 1-bromo-4-methoxybenzene (25.0 µL, 0.20 mmol, 1.00 equiv.), TIPS-NSO (52.7 mg, 0.24 mmol, 1.20 equiv.) and 1,4-dioxane (1.0 mL). Purification of the crude residue using column chromatography (10:1 to 5:1 petrol ether: ethyl acetate) yielded title compound **XX** (39.7 mg, 61%) as a brown solid.

^{1}H NMR (400 MHz, $CDCl_3$) δ 7.64 (d, *J* = 8.9 Hz, 2H), 6.99 (d, *J* = 8.9 Hz, 2H), 3.84 (s, 3H), 3.64 (s, 1H), 1.31 – 1.20 (m, 3H), 1.14 (dd, *J* = 7.3, 6.0 Hz, 18H).

^{13}**C NMR** (101 MHz, $CDCl_3$) δ 161.6, 141.8, 126.8, 114.4, 55.6, 18.1, 18.0, 11.9. (Note: For dimethyl carbons in tri-*iso*propylsilyl group, $NSi(CH(CH_3)_2)_3$, 2 peaks were found instead of 1 due to the loss of symmetry caused by chiral sulfur atom.)

M.P. : 78-80 ˚C (CH_2Cl_2)

HRMS (ESI) calcd. for $C_{16}H_{29}NO_2SSiNa^+$ $[M+Na]^+$: 350.1581; found: 350.1586.

IR (ATR) (ν_{max} cm $^{-1}$) = 3323, 1595, 1494, 1462, 1251, 1087, 1053, 994, 899.

4-Methyl-*N*-(triisopropylsilyl)benzenesulfinamide (1k)

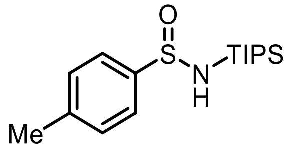

Synthesised according to **general procedure B**, using $Pd(OAc)_2$ (4.5 mg, 0.02 mmol, 10 mol%), PAd_2Bn (15.7 mg, 0.04 mmol. 20 mol%), HCO_2Cs (42.7 mg, 0.24 mmol, 1.20 equiv.), 1-bromo-4-methylbenzene (25.0 µL, 0.20 mmol, 1.00 equiv.), TIPS-NSO (52.7 mg, 0.24 mmol, 1.20 equiv.) and 1,4-dioxane (1.0 mL). Purification of the crude residue using column chromatography (10:1 to 5:1 petrol ether: ethyl acetate) yielded title compound **1k** (41.2 mg, 66%) as an off-white solid.

^{1}**H NMR** (400 MHz, $CDCl_3$) δ 7.61 (d, J = 8.3 Hz, 2H), 7.29 (d, J = 8.0 Hz, 2H), 3.67 (s, 1H), 2.40 (s, 3H), 1.30 - 1.20 (m, 3H), 1.14 (dd, J = 7.3, 5.8 Hz, 18H).

^{13}**C NMR** (101 MHz, $CDCl_3$) δ 147.3, 141.1, 129.7, 125.1, 21.4, 18.1, 18.0, 11.9. (Note: For dimethyl carbons in tri-*iso*propylsilyl group, $NSi(CH(CH_3)_2)_3$, 2 peaks were found instead of 1 due to the loss of symmetry caused by chiral sulfur atom.)

M.P. : 75-76 ˚C (CH_2Cl_2)

HRMS (ESI) calcd. for $C_{16}H_{29}NOSSi^+$ $[M+H]^+$: 312.1812; found: 312.1818.

IR (ATR) (ν_{max} cm $^{-1}$) =3300, 1690, 1463, 1382, 1258, 1080, 1051, 877, 771, 678.

4-(2,5-Dioxopyrrolidin-1-yl)-*N*-(triisopropylsilyl)benzenesulfinamide (1l)

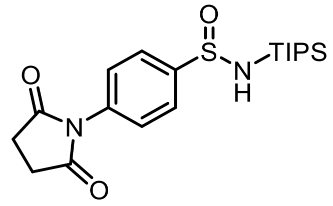

Synthesised according to **general procedure B**, using $Pd(OAc)_2$ (4.5 mg, 0.02 mmol, 10 mol%), PAd_2Bn (15.7 mg, 0.04 mmol. 20 mol%), HCO_2Cs (42.7 mg, 0.24 mmol, 1.20 equiv.), 1-(4-bromophenyl)pyrrolidine-2,5-dione (50.8 mg, 0.20 mmol, 1.00 equiv.), TIPS-NSO (52.7 mg, 0.24 mmol, 1.20 equiv.) and 1,4-dioxane (1.0 mL) at ***85 °C***. Purification of the crude residue using column chromatography (13:1 to 1:1 to 0:1 petrol ether: ethyl acetate) yielded title compound **1l** (41.7 mg, 53%) as a brown solid.

^{1}H NMR (400 MHz, $CDCl_3$) δ 7.84 (d, *J* = 8.6 Hz, 2H), 7.45 (d, *J* = 8.6 Hz, 2H), 3.77 (s, 1H), 2.91 (s, 4H), 1.32 – 1.22 (m, 3H), 1.14 (dd, *J* = 7.3, 5.5 Hz, 18H).

^{13}C NMR (101 MHz, $CDCl_3$) δ 175.9, 150.2, 134.1, 126.9, 126.2, 28.5, 18.1, 18.0, 11.9. (Note: For dimethyl carbons in tri-*iso*propylsilyl group, NSi(CH(CH_3)$_2$)$_3$, 2 peaks were found instead of 1 due to the loss of symmetry caused by chiral sulfur atom.)

M.P. : 130-132 °C (CH_2Cl_2)

HRMS (ESI) calcd. for $C_{19}H_{31}N_2O_3SSi^+$ $[M+H]^+$: 395.1819; found: 395.1834.

IR (ATR) (ν_{max} cm $^{-1}$) = 2942, 2865, 1725, 1712, 1496, 1460, 1381, 1167, 1083, 1058, 878, 669.

2-Methyl-*N*-(triisopropylsilyl)benzenesulfinamide (1m)

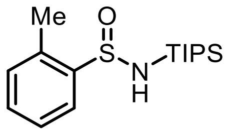

Synthesised according to **general procedure B**, using $Pd(OAc)_2$ (4.5 mg, 0.02 mmol, 10 mol%), PAd_2Bn (15.7 mg, 0.04 mmol. 20 mol%), HCO_2Cs (42.7 mg, 0.24 mmol, 1.20 equiv.), 1-bromo-2-methylbenzene (24.0 μL, 0.20 mmol, 1.00 equiv.), TIPS-NSO (52.7 mg, 0.24 mmol, 1.20 equiv.) and 1,4-dioxane (1.0 mL). Purification of the crude residue using column chromatography (10:1 to 5:1 petrol ether: ethyl acetate) yielded title compound **1m** (39.3 mg, 63%) as an off-white solid.

^{1}H NMR (400 MHz, $CDCl_3$) δ 8.04 – 7.96 (m, 1H), 7.42 – 7.31 (m, 2H), 7.22 – 7.14 (m, 1H), 3.59 (s, 1H), 2.49 (s, 3H), 1.29 – 1.19 (m, 3H), 1.12 (t, *J* = 7.5 Hz, 18H).

^{13}C NMR (101 MHz, $CDCl_3$) δ 148.5, 134.8, 131.0, 130.7, 127.0, 122.3, 18.9, 18.1, 18.0, 12.1. (Note: For dimethyl carbons in tri-*iso*propylsilyl group, $NSi(CH(CH_3)_2)_3$, 2 peaks were found instead of 1 due to the loss of symmetry caused by chiral sulfur atom.)

M.P. : 79-80 °C (CH_2Cl_2)

HRMS (ESI) calcd. for $C_{16}H_{29}NOSSi^+$ $[M+H]^+$: 312.1812; found: 312.1825.

IR (ATR) (ν_{max} cm $^{-1}$) = 2966, 2866, 1469, 1382, 1064, 1033, 878, 773, 744.

3-Methoxy-*N*-(triisopropylsilyl)benzenesulfinamide (1n)

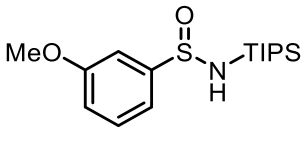

Synthesised according to **general procedure B**, using $Pd(OAc)_2$ (4.5 mg, 0.02 mmol, 10 mol%), PAd_2Bn (15.7 mg, 0.04 mmol. 20 mol%), HCO_2Cs (42.7 mg, 0.24 mmol, 1.20 equiv.), 1-bromo-3-methoxybenzene (25.0 μL, 0.20 mmol, 1.00 equiv.), TIPS-NSO (52.7 mg, 0.24 mmol, 1.20 equiv.) and 1,4-dioxane (1.0 mL). Purification of the crude residue using column chromatography (10:1 to 5:1 petrol ether: ethyl acetate) yielded title compound **XX** (42.0 mg, 64%) as a yellow solid.

^{1}H NMR (400 MHz, $CDCl_3$) δ 7.37 (t, J = 7.9 Hz, 1H), 7.31 (dd, J = 2.6, 1.6 Hz, 1H), 7.25 (ddd, J = 7.7, 1.6, 1.0 Hz, 1H), 6.97 (ddd, J = 8.2, 2.6, 1.0 Hz, 1H), 3.83 (s, 3H), 3.71 (s, 1H), 1.30 – 1.19 (m, 3H), 1.13 (dd, J = 7.2, 5.6 Hz, 18H).

^{13}C NMR (101 MHz, $CDCl_3$) δ 160.2, 151.7, 130.1, 117.3, 109.4, 55.6, 18.1, 18.0, 11.9. (Note: 1) One aromatic carbon is missing. 2) For dimethyl carbons in tri-*iso*propylsilyl group, $NSi(CH(CH_3)_2)_3$, 2 peaks were found instead of 1 due to the loss of symmetry caused by chiral sulfur atom.)

M.P. : 68-70 °C (CH_2Cl_2)

HRMS (ESI) calcd. for $C_{16}H_{30}NO_2SSi^+$ $[M+H]^+$: 328.1761; found: 328.1765.

IR (ATR) (ν_{max} cm $^{-1}$) = 1691, 1468, 1250, 1045, 878, 858, 765.

N-(Triisopropylsilyl)-2-vinylbenzenesulfinamide (1o)

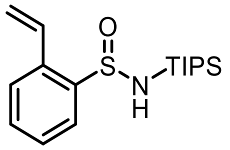

Synthesised according to **general procedure B**, using $Pd(OAc)_2$ (4.5 mg, 0.02 mmol, 10 mol%), PAd_2Bn (15.7 mg, 0.04 mmol. 20 mol%), HCO_2Cs (42.7 mg, 0.24 mmol, 1.20 equiv.), 1-bromo-2-vinylbenzene (25.1 μL, 0.20 mmol, 1.00 equiv.), TIPS-NSO (52.7 mg, 0.24 mmol,

1.20 equiv.) and 1,4-dioxane (1.0 mL). Purification of the crude residue using column chromatography (10:1 to 5:1 petrol ether: ethyl acetate) yielded title compound **1o** (31.5 mg, 47%) as a yellow solid.

^{1}H NMR (400 MHz, $CDCl_3$) δ 8.05 – 7.97 (m, 1H), 7.60 – 7.51 (m, 1H), 7.49 – 7.38 (m, 2H), 7.26 (dd, *J* = 17.3, 11.0 Hz, 1H), 5.75 (dd, *J* = 17.3, 1.0 Hz, 1H), 5.38 (dd, *J* = 11.0, 1.0 Hz, 1H), 3.69 (s, 1H), 1.22 (ddt, *J* = 13.8, 9.2, 6.4 Hz, 3H), 1.10 (dd, *J* = 8.2, 7.2 Hz, 18H).

^{13}C NMR (101 MHz, $CDCl_3$) δ 147.7, 135.0, 132.4, 130.9, 128.8, 126.2, 122.5, 117.5, 18.1, 18.0, 12.0. (Note: For dimethyl carbons in tri-*iso*propylsilyl group, $NSi(CH(CH_3)_2)_3$, 2 peaks were found instead of 1 due to the loss of symmetry caused by chiral sulfur atom.)

M.P. : 84-85 °C (CH_2Cl_2)

HRMS (ESI) calcd. for $C_{17}H_{30}NOSSi^+$ $[M+H]^+$: 324.1812; found: 324.1815.

IR (ATR) (ν_{max} cm^{-1}) =1690, 1464, 1124, 1064, 913, 897, 770.

***N*-(Triisopropylsilyl)benzo[d][1,3]dioxole-5-sulfinamide (1p)**

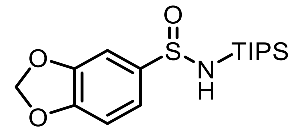

Synthesised according to **general procedure B**, using $Pd(OAc)_2$ (4.5 mg, 0.02 mmol, 10 mol%), PAd_2Bn (15.7 mg, 0.04 mmol. 20 mol%), HCO_2Cs (42.7 mg, 0.24 mmol, 1.20 equiv.), 5-bromobenzo[d][1,3]dioxole (24.1 µL, 0.20 mmol, 1.00 equiv.), TIPS-NSO (52.7 mg, 0.24 mmol, 1.20 equiv.) and 1,4-dioxane (1.0 mL). Purification of the crude residue using column chromatography (10:1 to 5:1 petrol ether: ethyl acetate) yielded title compound **1p** (36.0 mg, 53%) as a brown solid.

^{1}H NMR (400 MHz, $CDCl_3$) δ 7.22 (dd, *J* = 8.1, 1.8 Hz, 1H), 7.16 (d, *J* = 1.7 Hz, 1H), 6.88 (d, *J* = 8.1 Hz, 1H), 6.03 (s, 2H), 3.67 (s, 1H), 1.30 – 1.18 (m, 3H), 1.13 (dd, *J* = 7.3, 6.1 Hz, 18H).

^{13}C NMR (101 MHz, $CDCl_3$) δ 149.9, 148.3, 143.9, 119.6, 108.5, 105.4, 101.9, 18.1, 19.0, 11.9. (Note: For dimethyl carbons in tri-*iso*propylsilyl group, NSi(CH(CH_3)$_2$)$_3$, 2 peaks were found instead of 1 due to the loss of symmetry caused by chiral sulfur atom.)

M.P. : 79-80 ˚C (CH_2Cl_2)

HRMS (ESI) calcd. for $C_{16}H_{28}NO_3SSi^+$ $[M+H]^+$: 342.1554; found: 342.1567.

IR (ATR) (ν_{max} cm $^{-1}$) = 1500, 1476, 1252, 1234, 1082, 996, 876, 760.

3-Methyl-1-oxo-*N*-(triisopropylsilyl)-2,3-dihydro-1H-indene-4-sulfinamide (1q)

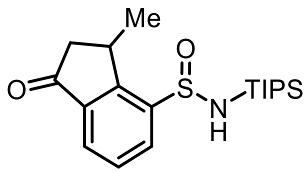

Synthesised according to **general procedure B**, using $Pd(OAc)_2$ (4.5 mg, 0.02 mmol, 10 mol%), PAd_2Bn (15.7 mg, 0.04 mmol. 20 mol%), HCO_2Cs (42.7 mg, 0.24 mmol, 1.20 equiv.), 4-bromo-3-methyl-2,3-dihydro-1H-inden-1-one (45.1 mg, 0.20 mmol, 1.00 equiv.), TIPS-NSO (52.7 mg, 0.24 mmol, 1.20 equiv.) and 1,4-dioxane (1.0 mL). Purification of the crude residue (4.4:1 dr) using column chromatography (5:1 to 3:1 Petrol ether: ethyl acetate) yielded title major diastereoisomer **1qa** (34.4 mg, 47%) as a white solid, minor diastereoisomer **1qb** (7.5 mg, 10%) as a white solid.

*major diastereoisomer **1qa***

^{1}H NMR (400 MHz, $CDCl_3$) δ 8.29 (dd, *J* = 7.7, 1.2 Hz, 1H), 7.83 (dd, *J* = 7.6, 1.2 Hz, 1H), 7.62 – 7.54 (m, 1H), 3.79 (pd, *J* = 7.2, 2.0 Hz, 1H), 3.68 (s, 1H), 2.93 (dd, *J* = 19.1, 7.7 Hz,

1H), 2.37 (dd, *J* = 19.1, 2.0 Hz, 1H), 1.46 (d, *J* = 7.1 Hz, 3H), 1.31-1.20 (m, 3H), 1.13 (t, *J* = 7.0 Hz, 18H).

^{13}C NMR (101 MHz, $CDCl_3$) δ 205.2, 155.9, 147.9, 137.5, 129.3, 128.9, 126.3, 45.4, 32.3, 22.8, 18.05, 18.00, 12.1. (Note: For dimethyl carbons in tri-*iso*propylsilyl group, NSi(CH(CH_3)$_2$)$_3$, 2 peaks were found instead of 1 due to the loss of symmetry caused by chiral sulfur atom.)

M.P. : 119-120 ˚C (CH_2Cl_2)

IR (ATR) (ν_{max} cm $^{-1}$) = 3204, 1703, 1590, 1427, 1088, 1046, 859.

HRMS (ESI) calcd. for $C_{19}H_{32}NO_2SSi^+$ $[M+H]^+$:366.1918; found: 366.1914.

minor diastereoisomer ***1qb***

^{1}H NMR (400 MHz, $CDCl_3$) δ 7.94 (dd, *J* = 7.7, 1.1 Hz, 1H), 7.84 (dd, *J* = 7.6, 1.1 Hz, 1H), 7.53 (t, *J* = 7.6 Hz, 1H), 4.15 (pd, *J* = 7.2, 1.9 Hz, 1H), 3.89 (s, 1H), 2.97 (dd, *J* = 19.1, 7.7 Hz, 1H), 2.37 (dd, *J* = 19.1, 1.9 Hz, 1H), 1.48 (d, *J* = 7.1 Hz, 3H), 1.34 - 1.24 (m, 3H), 1.15 (dd, *J* = 16.0, 7.3 Hz, 18H).

^{13}C NMR (101 MHz, $CDCl_3$) δ 205.7, 158.4, 147.7, 137.9, 129.9, 128.7, 126.4, 45.5, 32.4, 22.6, 18.1, 18.0, 12.0. (Note: For dimethyl carbons in tri-*iso*propylsilyl group, NSi(CH(CH_3)$_2$)$_3$, 2 peaks were found instead of 1 due to the loss of symmetry caused by chiral sulfur atom.)

M.P. : 109-110 ˚C (CH_2Cl_2)

HRMS (ESI) calcd. for $C_{19}H_{32}NO_2SSi^+$ $[M+H]^+$:366.1918; found: 366.1919.

IR (ATR) (ν_{max} cm $^{-1}$) = 3069, 1719, 1459, 1328, 1280, 1045, 904, 883, 777.

6-Methoxy-*N*-(triisopropylsilyl)pyridine-3-sulfinamide (1r)

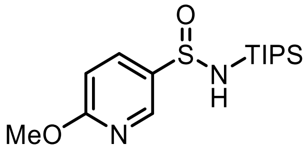

Synthesised according to **general procedure A**, using SPhos Pd G3 (15.6 mg, 0.02 mmol, 10 mol%), HCO_2Cs (53. 4 mg, 0.30 mmol, 1.50 equiv.), 5-bromo-2-methoxypyridine (25.9 µL, 0.20 mmol, 1.00 equiv.), TIPS-NSO (52.7 mg, 0.24 mmol, 1.20 equiv.) and 1,4-dioxane (1.0 mL). Purification of the crude residue using column chromatography (10:1 to 3:1 petrol ether: ethyl acetate) yielded title compound **1r** (60.0 mg, 91%) as a yellow solid.

^{1}H NMR (400 MHz, $CDCl_3$) δ 8.44 (dd, *J* = 2.6, 0.7 Hz, 1H), 7.88 (dd, *J* = 8.7, 2.5 Hz, 1H), 6.81 (dd, *J* = 8.7, 0.7 Hz, 1H), 3.96 (s, 3H), 3.77 (s, 1H), 1.25 (ddt, *J* = 13.3, 8.7, 6.4 Hz, 3H), 1.13 (t, *J* = 7.1 Hz, 18H).

^{13}C NMR (101 MHz, $CDCl_3$) δ 165.7, 145.2, 138.8, 135.9, 111.4, 54.1, 18.0, 17.9, 11.9. (Note: For dimethyl carbons in tri-*iso*propylsilyl group, $NSi(CH(CH_3)_2)_3$, 2 peaks were found instead of 1 due to the loss of symmetry caused by chiral sulfur atom.)

M.P. : 95-96 ˚C (CH_2Cl_2)

HRMS (ESI) calcd. for $C_{15}H_{29}N_2O_2SSi^+$ $[M+H]^+$: 329.1714; found: 329.1717.

IR (ATR) (ν_{max} cm $^{-1}$) = 3058, 1590, 1562, 1477, 1366, 1280, 1255, 1055, 1044, 880.

6-(Trifluoromethyl)-*N*-(triisopropylsilyl)pyridine-3-sulfinamide (1s)

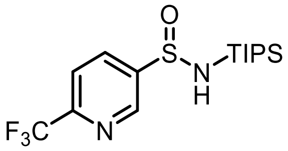

Synthesised according to **general procedure A**, using SPhos Pd G3 (15.6 mg, 0.02 mmol, 10 mol%), HCO_2Cs (53.4 mg, 0.30 mmol, 1.50 equiv.), 5-bromo-2-(trifluoromethyl)pyridine (45.2 mg, 0.20 mmol, 1.00 equiv.), TIPS-NSO (52.7 mg, 0.24 mmol, 1.20 equiv.) and 1,4-dioxane (1.0 mL). Purification of the crude residue using column chromatography (10:1 to 5:1 petrol ether: ethyl acetate) yielded title compound **1s** (52.6 mg, 72%) as a white solid.

^{1}H NMR (400 MHz, $CDCl_3$) δ 8.99 (d, *J* = 2.2 Hz, 1H), 8.28 (dd, *J* = 8.2, 2.2 Hz, 1H), 7.82 (dd, *J* = 8.2, 0.8 Hz, 1H), 3.93 (s, 1H), 1.28 (ddt, *J* = 13.6, 8.7, 6.6 Hz, 3H), 1.14 (dd, *J* = 7.4, 6.1 Hz, 18H).

^{13}C NMR (101 MHz, $CDCl_3$) δ 150.0 (q, *J* = 35.0 Hz), 149.4, 147.6, 135.2, 121.3 (q, *J* = 274.5 Hz), 120.8 (q, *J* = 2.8 Hz), 17.99, 17.87, 11.9. (Note: For dimethyl carbons in tri-*iso*propylsilyl group, $NSi(CH(CH_3)_2)_3$, 2 peaks were found instead of 1 due to the loss of symmetry caused by chiral sulfur atom.)

^{19}F NMR (377 MHz, $CDCl_3$) δ -67.91 (s).

M.P. : 117-118˚C (CH_2Cl_2)

HRMS (ESI) calcd. for $C_{15}H_{26}F_3N_2OSSi^+$ $[M+H]^+$: 367.1482; found: 367.1485.

IR (ATR) (ν_{max} cm $^{-1}$) = 2962, 2868, 1710, 1471, 1374, 1340, 1176, 1131, 1079, 884, 859, 737.

***Tert*-butyl (5-(((triisopropylsilyl)amino)sulfinyl)pyridin-2-yl)carbamate (1t)**

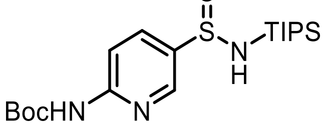

Synthesised according to **general procedure A**, using SPhos Pd G3 (15.6 mg, 0.02 mmol, 10 mol%), HCO_2Cs (53. 4 mg, 0.30 mmol, 1.50 equiv.), *tert*-butyl (5-

(((triisopropylsilyl)amino)sulfinyl)pyridin-2-yl)carbamate (54.6 mg, 0.20 mmol, 1.00 equiv.), TIPS-NSO (52.7 mg, 0.24 mmol, 1.20 equiv.) and 1,4-dioxane (1.0 mL). Purification of the crude residue using column chromatography (10:1 to 3:1 to Petrol ether: ethyl acetate) yielded title compound **1t** (57.8 mg, 70%) as an off-white solid.

^{1}H NMR (400 MHz, $CDCl_3$) δ 8.80 (s, 1H), 8.56 (dd, *J* = 2.5, 0.8 Hz, 1H), 8.14 – 8.06 (m, 1H), 7.95 (dd, *J* = 8.8, 2.4 Hz, 1H), 3.81 (s, 1H), 1.53 (s, 9H), 1.30 – 1.21 (m, 3H), 1.12 (t, *J* = 6.9 Hz, 18H).

^{13}C NMR (101 MHz, $CDCl_3$) δ 154.2, 152.4, 145.5, 139.9, 135.7, 112.3, 81.8, 28.4, 18.04, 17.93, 11.9. (Note: For dimethyl carbons in tri-*iso*propylsilyl group, $NSi(CH(CH_3)_2)_3$, 2 peaks were found instead of 1 due to the loss of symmetry caused by chiral sulfur atom.)

M.P. : 128-129°C (CH_2Cl_2)

HRMS (ESI) calcd. for $C_{19}H_{35}N_3O_3SSiNa^+$ $[M+Na]^+$:436.2061; found: 436.2074.

IR (ATR) (ν_{max} cm $^{-1}$) = 2985, 2868, 1736, 1586, 1524, 1468, 1382, 1366, 1290, 1270, 1154, 1114, 882.

6-(Tert-butyl)-*N*-(triisopropylsilyl)pyridine-2-sulfinamide (1u)

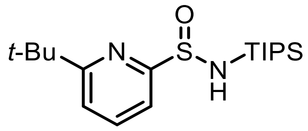

Synthesised according to **general procedure A**, using SPhos Pd G3 (15.6 mg, 0.02 mmol, 10 mol%), HCO_2Cs (53.4 mg, 0.30 mmol, 1.50 equiv.), 2-bromo-6-(tert-butyl)pyridine (42.8 mg, 0.20 mmol, 1.00 equiv.), TIPS-NSO (52.7 mg, 0.24 mmol, 1.20 equiv.) and 1,4-dioxane (1.0 mL). Purification of the crude residue using column chromatography (5:1 to 3:1 petrol ether: ethyl acetate) yielded title compound **u** (29.8 mg, 42%) as an off-white solid.

^{1}H NMR (400 MHz, $CDCl_3$) δ 7.80 (t, *J* = 7.7 Hz, 1H), 7.67 (dd, *J* = 7.6, 0.9 Hz, 1H), 7.43 (dd, *J* = 7.8, 1.0 Hz, 1H), 5.23 (s, 1H), 1.38 (s, 9H), 1.31 – 1.21 (m, 3H), 1.12 (t, *J* = 7.6 Hz, 18H).

^{13}C NMR (101 MHz, $CDCl_3$) δ 169.5, 163.4, 138.4, 121.3, 118.4, 38.0, 30.2, 18.02, 18.00, 11.9. (Note: For dimethyl carbons in tri-*iso*propylsilyl group, NSi(CH(CH_3)$_2$)$_3$, 2 peaks were found instead of 1 due to the loss of symmetry caused by chiral sulfur atom.)

M.P. : 68-70 °C (CH_2Cl_2)

HRMS (ESI) calcd. for $C_{18}H_{35}N_2OSSi^+$ $[M+H]^+$: 355.2234; found: 355.2243.

IR (ATR) (ν_{max} cm $^{-1}$) = 2955, 2885, 1736, 1585, 1524, 1463, 1318, 1270, 1155, 1056, 882, 678.

2-Methoxy-*N*-(triisopropylsilyl)pyrimidine-5-sulfinamide (1v)

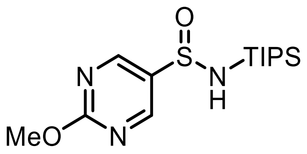

Synthesised according to **general procedure A**, using SPhos Pd G3 (15.6 mg, 0.02 mmol, 10 mol%), HCO_2Cs (53. 4 mg, 0.30 mmol, 1.50 equiv.), 5-bromo-2-methoxypyrimidine (37.8 mg, 0.20 mmol, 1.00 equiv.), TIPS-NSO (52.7 mg, 0.24 mmol, 1.20 equiv.) and 1,4-dioxane (1.0 mL). Purification of the crude residue using column chromatography (10:1 to 5:1 to 3:1 petrol ether: ethyl acetate) yielded title compound **1v** (56.9 mg, 86%) as an off-white solid.

^{1}H NMR (400 MHz, $CDCl_3$) δ 8.74 (s, 2H), 4.05 (s, 3H), 3.97 (s, 1H), 1.25 (ddt, *J* = 13.4, 8.6, 6.5 Hz, 3H), 1.12 (t, *J* = 7.4 Hz, 18H).

^{13}C NMR (101 MHz, $CDCl_3$) δ 166.7, 157.7, 137.2, 55.7, 17.99, 17.87, 11.8. (Note: For dimethyl carbons in tri-*iso*propylsilyl group, NSi(CH(CH_3)$_2$)$_3$, 2 peaks were found instead of 1 due to the loss of symmetry caused by chiral sulfur atom.)

M.P. : 100-102 ˚C (CH_2Cl_2)

HRMS (ESI) calcd. for $C_{14}H_{28}N_3O_2SSi^+$ $[M+H]^+$: 330.1666; found: 330.1676.

IR (ATR) (ν_{max} cm $^{-1}$) = 1573, 1547, 1470, 1398, 1327, 1117, 1060, 1043, 947, 907, 801, 680.

***N*-(Triisopropylsilyl)quinoline-6-sulfinamide (1w)**

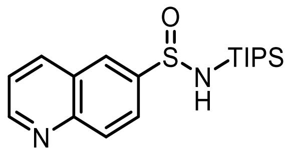

Synthesised according to **general procedure A**, using SPhos Pd G3 (15.6 mg, 0.02 mmol, 10 mol%), HCO_2Cs (53.4 mg, 0.30 mmol, 1.50 equiv.), 6-bromoquinoline (41.6 mg, 0.20 mmol, 1.00 equiv.), TIPS-NSO (52.7 mg, 0.24 mmol, 1.20 equiv.) and 1,4-dioxane (1.0 mL). Purification of the crude residue using column chromatography (1:1 to 1:4 petrol ether: ethyl acetate) yielded title compound **1w** (51.3 mg, 74%) as a white solid.

^{1}H NMR (400 MHz, $CDCl_3$) 9.01 – 8.94 (m, 1H), 8.29 (d, J = 2.1 Hz, 1H), 8.23 (dd, J = 8.4, 1.7 Hz, 1H), 8.15 (d, J = 8.8 Hz, 1H), 7.88 (dd, J = 8.8, 2.0 Hz, 1H), 7.48 (dd, J = 8.3, 4.3 Hz, 1H), 3.88 (bs, 1H), 1.35 – 1.23 (m, 3H), 1.17 (dd, J = 7.3, 4.3 Hz, 18H);

^{13}C NMR (101 MHz, $CDCl_3$) 152.1, 149.1, 147.9, 137.0, 130.7, 127.9, 125.4, 125.2, 122.3, 18.1, 18.0, 12.0; (Note: For dimethyl carbons in tri-*iso*propylsilyl group, NSi(CH(CH_3)$_2$)$_3$, 2 peaks were found instead of 1 due to the loss of symmetry caused by chiral sulfur atom.)

M.P. 91–93 °C (Et_2O);

HRMS (ESI) calcd. for $C_{18}H_{28}N_2OSSiK^+$ $[M+K]^+$: 387.1323; found: 387.1317.

IR (ATR) (ν_{max} cm $^{-1}$) = 3133, 2945, 2867, 1492, 1464, 1319, 1085, 1054, 879, 772, 737, 679;

1-Acetyl-*N*-(triisopropylsilyl)-1H-indole-5-sulfinamide (1x)

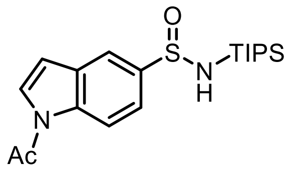

Synthesised according to **general procedure A**, using SPhos Pd G3 (15.6 mg, 0.02 mmol, 10 mol%), HCO_2Cs (53.4 mg, 0.30 mmol, 1.50 equiv.), 1-(5-bromo-1H-indol-1-yl)ethan-1-one (47.6 mg, 0.20 mmol, 1.00 equiv.), TIPS-NSO (52.7 mg, 0.24 mmol, 1.20 equiv.) and 1,4-dioxane (1.0 mL). Purification of the crude residue using column chromatography (5:1 to 3:1 to 1:1 petrol ether: ethyl acetate) yielded title compound **1x**(44.0 mg, 58%) as a brown solid.

^{1}H NMR (400 MHz, $CDCl_3$) δ 8.52 (d, *J* = 8.7 Hz, 1H), 8.03 – 7.98 (m, 1H), 7.61 (dd, *J* = 8.7, 1.8 Hz, 1H), 7.50 (d, *J* = 3.8 Hz, 1H), 6.70 (dd, *J* = 3.8, 0.8 Hz, 1H), 3.76 (s, 1H), 2.65 (s, 3H), 1.27 (ddt, *J* = 13.4, 8.9, 6.5 Hz, 3H), 1.15 (dd, *J* = 7.3, 4.8 Hz, 18H).

^{13}C NMR (101 MHz, $CDCl_3$) δ 168.8, 145.5, 136.6, 130.7, 126.8, 121.8, 117.8, 117.0, 109.4, 24.1, 18.1, 18.0, 11.9. (Note: For dimethyl carbons in tri-*iso*propylsilyl group, NSi(CH(*CH$_3$*)$_2$)$_3$, 2 peaks were found instead of 1 due to the loss of symmetry caused by chiral sulfur atom.)

M.P. : 110-112 ˚C (CH_2Cl_2)

HRMS (ESI) calcd. for $C_{19}H_{31}N_2O_2SSi^+$ $[M+H]^+$: 379.1870; found: 379.1877.

IR (ATR) (ν_{max} cm $^{-1}$) = 3019, 1699, 1534, 1449, 1382, 1330, 1199, 1070, 878.

***N*-(triisopropylsilyl)thiophene-3-sulfinamide (1y)**

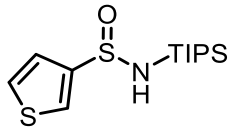

Synthesised according to **general procedure C**, using $Pd(OAc)_2$ (4.5 mg, 0.02 mmol, 10 mol%), PAd_2Bn (15.7 mg, 0.04 mmol. 20 mol%), HCO_2Cs (42.7 mg, 0.24 mmol, 1.20 equiv.), 3-bromothiophene (18.7 μL, 0.20 mmol, 1.00 equiv.), TIPS-NSO (52.7 mg, 0.24 mmol, 1.20 equiv.) and 1,4-dioxane (1.0 mL). Purification of the crude residue using column chromatography (10:1 to 5:1 to 3:1 Petrol ether: ethyl acetate) yielded title compound **1y** (29.5 mg, 48%) as a brown solid.

^{1}H NMR (400 MHz, $CDCl_3$) δ 7.73 (dd, *J* = 3.1, 1.3 Hz, 1H), 7.42 (dd, *J* = 5.1, 3.1 Hz, 1H), 7.21 (dd, *J* = 5.1, 1.3 Hz, 1H), 3.92 (s, 1H), 1.29 – 1.21 (m, 3H), 1.13 (t, *J* = 7.1 Hz, 18H).

^{13}C NMR (101 MHz, $CDCl_3$) δ 150.7, 127.9, 126.1, 124.7, 18.05, 17.94, 11.9. (Note: For dimethyl carbons in tri-*iso*propylsilyl group, $NSi(CH(CH_3)_2)_3$, 2 peaks were found instead of 1 due to the loss of symmetry caused by chiral sulfur atom.)

M.P. : 74-75 °C (CH_2Cl_2)

HRMS (ESI) calcd. for $C_{13}H_{26}NOS_2Si^+$ $[M+H]^+$:304.1220; found: 304.1224.

IR (ATR) (ν_{max} cm $^{-1}$) = 3186, 1460, 1310, 1095, 1071, 901, 883, 785.

4-(5-(*p*-Tolyl)-3-(trifluoromethyl)-1H-pyrazol-1-yl)-*N*-(triisopropylsilyl)benzenesulfinamide (1z)

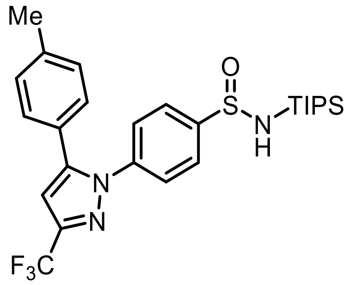

Synthesised according to **general procedure B**, using $Pd(OAc)_2$ (4.5 mg, 0.02 mmol, 10 mol%), PAd_2Bn (15.7 mg, 0.04 mmol. 20 mol%), HCO_2Cs (42.7 mg, 0.24 mmol, 1.20 equiv.), 1-(4-bromophenyl)-5-(p-tolyl)-3-(trifluoromethyl)-1H-pyrazole (76.2 mg, 0.20 mmol, 1.00 equiv.), TIPS-NSO (52.7 mg, 0.24 mmol, 1.20 equiv.) and 1,4-dioxane (1.0 mL). Purification of the crude residue using column chromatography (10:1 to 5:1 petrol ether: ethyl acetate) yielded title compound **1z** (86.3 mg, 83%) as a brown oil.

^{1}H NMR (400 MHz, $CDCl_3$) δ 7.72 (d, J = 8.6 Hz, 2H), 7.44 (d, J = 8.6 Hz, 2H), 7.15 (d, J = 8.0 Hz, 2H), 7.10 (d, J = 8.3 Hz, 2H), 6.73 (s, 1H), 3.72 (s, 1H), 2.36 (s, 3H), 1.26 (ddt, J = 13.5, 8.7, 6.5 Hz, 3H), 1.13 (dd, J = 7.3, 5.6 Hz, 18H).

^{13}C NMR (101 MHz, $CDCl_3$) δ 150.0, 145.2, 143.8 (q, J = 38.5 Hz), 141.2, 139.6, 129.7, 128.8, 126.0, 126.0, 125.8, 121.3 (q, J = 268.9 Hz), 106.0 (d, J = 2.1 Hz), 21.4, 18.0, 17.9, 11.9. (Note: For dimethyl carbons in tri-*iso*propylsilyl group, $NSi(CH(CH_3)_2)_3$, 2 peaks were found instead of 1 due to the loss of symmetry caused by chiral sulfur atom.)

^{19}F NMR (377 MHz, $CDCl_3$) δ -62.34 (s).

IR (ATR) (ν_{max} cm $^{-1}$) = 3233, 1472, 1375, 1272, 1236, 1163, 1097, 976, 880.

Tert-butyl 4-((5-(((triisopropylsilyl)amino)sulfinyl)pyridin-2-yl)oxy)piperidine-1-carboxylate (1aa)

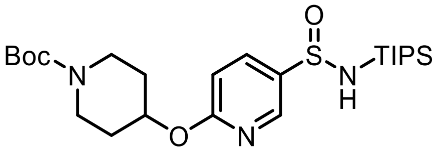

Synthesised according to **general procedure A**, using SPhos Pd G3 (15.6 mg, 0.02 mmol, 10 mol%), HCO_2Cs (53.4 mg, 0.30 mmol, 1.50 equiv.), tert-butyl 4-((5-bromopyridin-2-yl)oxy)piperidine-1-carboxylate (71.4 mg, 0.20 mmol, 1.00 equiv.), TIPS-NSO (52.7 mg, 0.24 mmol, 1.20 equiv.) and 1,4-dioxane (1.0 mL). Purification of the crude residue using column chromatography (5:1 to 3:1 petrol ether: ethyl acetate) yielded title compound **1aa** (89.5 mg, 90%) as a brown oil.

^{1}H NMR (400 MHz, $CDCl_3$) δ 8.40 (dd, *J* = 2.6, 0.7 Hz, 1H), 7.87 (dd, *J* = 8.7, 2.6 Hz, 1H), 6.78 (dd, *J* = 8.7, 0.7 Hz, 1H), 5.26 (tt, *J* = 7.8, 3.7 Hz, 1H), 3.81-3.68 (m,3H), 3.27 (ddd, *J* = 12.9, 8.5, 3.5 Hz, 2H), 2.04 – 1.91 (m, 2H), 1.71 (dtt, *J* = 12.5, 8.2, 4.0 Hz, 2H), 1.45 (s, 9H), 1.30 – 1.21 (m, 3H), 1.13 (t, *J* = 7.2 Hz, 18H).

^{13}C NMR (101 MHz, $CDCl_3$) δ 164.6, 154.9, 145.1, 138.7, 136.1, 112.0, 79.7, 71.3, 41.2 (bs), 30.8, 28.6, 18.1, 17.9, 11.9. (Note: For dimethyl carbons in tri-*iso*propylsilyl group, $NSi(CH(CH_3)_2)_3$, 2 peaks were found instead of 1 due to the loss of symmetry caused by chiral sulfur atom.)

HRMS (ESI) calcd. for $C_{24}H_{44}N_3O_4SSi^+$ $[M+H]^+$: 498.2816; found: 498.2818.

IR (ATR) (ν_{max} cm $^{-1}$) = 2945, 2867, 1681, 1586, 1467, 1426, 1366, 1302, 1277, 1238, 1171, 1095, 877, 756.

***N*-(4-(((Triisopropylsilyl)amino)sulfinyl)phenethyl)-6-(trimethylsilyl)hex-5-ynamide (1ab)**

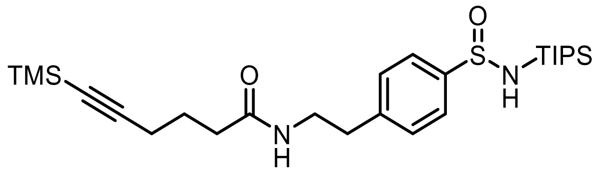

Synthesised according to **general procedure B**, using $Pd(OAc)_2$ (4.5 mg, 0.02 mmol, 10 mol%), PAd_2Bn (15.7 mg, 0.04 mmol. 20 mol%), HCO_2Cs (42.7 mg, 0.24 mmol, 1.20 equiv.), *N*-(4-bromophenethyl)-6-(trimethylsilyl)hex-5-ynamide (73.3 mg, 0.20 mmol, 1.00 equiv.), TIPS-NSO (52.7 mg, 0.24 mmol, 1.20 equiv.) and 1,4-dioxane (1.0 mL). Purification of the crude residue using column chromatography (10:1 to 3:1 dichloromethane: ethyl acetate) yielded title compound **1ab** (38.2 mg, 38%) as an off-white solid.

^{1}H NMR (400 MHz, $CDCl_3$) δ 7.67 (d, *J* = 8.3 Hz, 2H), 7.32 (d, *J* = 8.2 Hz, 2H), 5.57 (t, *J* = 6.1 Hz, 1H), 3.68 (s, 1H), 3.60 – 3.44 (m, 2H), 2.87 (t, *J* = 7.1 Hz, 2H), 2.25 (td, *J* = 7.2, 1.7 Hz, 4H), 1.86 – 1.76 (m, 2H), 1.32 – 1.21 (m,3H), 1.17 – 1.10 (m, 18H), 0.13 (s, 9H).

^{13}C NMR (101 MHz, $CDCl_3$) δ 172.5, 148.5, 142.1, 129.5, 125.5, 106.3, 85.7, 40.6, 35.7, 35.3, 24.4, 19.4, 18.1, 18.0, 11.9, 0.3. (Note: For dimethyl carbons in tri-*iso*propylsilyl group, $NSi(CH(CH_3)_2)_3$, 2 peaks were found instead of 1 due to the loss of symmetry caused by chiral sulfur atom.)

M.P. : 130-132 ˚C (CH_2Cl_2)

HRMS (ESI) calcd. for $C_{26}H_{46}N_2O_2SSi_2^+$ $[M+H]^+$:507.2891; found: 507.2912.

IR (ATR) (ν_{max} cm $^{-1}$) = 3152, 1650, 1557, 1248, 1082, 1054, 879, 843.

1-Tosyl-*N*-(triisopropylsilyl)-1,2,3,6-tetrahydropyridine-4-sulfinamide (3a)

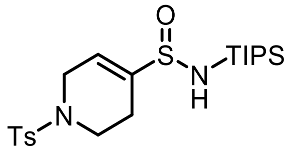

Synthesised according to **general procedure C**, using Pd(OAc)$_2$ (4.5 mg, 0.02 mmol, 10 mol%), PAd$_2$Bn (15.7 mg, 0.04 mmol. 20 mol%), HCO$_2$K (20.2 mg, 0.24 mmol, 1.20 equiv.), 1-tosyl-1,2,3,6-tetrahydropyridin-4-yl trifluoromethanesulfonate (77.1 mg, 0.2 mmol, 1.00 equiv.), TIPS-NSO (52.7 mg, 0.24 mmol, 1.20 equiv.) and 1,4-dioxane (1.0 mL). Purification of the crude residue using column chromatography (5:1 to 3:1 Petrol ether: ethyl acetate) yielded title compound **3a** (65.0 mg, 71%) as a brown solid.

^{1}H NMR (400 MHz, CDCl$_3$) δ 7.65 (d, *J* = 8.0 Hz, 2H), 7.31 (d, *J* = 8.0 Hz, 2H), 6.39 (tt, *J* = 3.3, 1.5 Hz, 1H), 3.97 – 3.86 (m, 1H), 3.70 – 3.59 (m, 1H), 3.54 (dt, *J* = 11.9, 4.9 Hz, 1H), 3.34 (s, 1H), 3.00 (ddd, *J* = 12.2, 8.2, 4.4 Hz, 1H), 2.53 (dtt, *J* = 16.0, 5.0, 2.4 Hz, 1H), 2.40 (s, 3H), 2.33 – 2.23 (m, 1H), 1.22 – 1.11 (m, 3H), 1.06 (dd, *J* = 9.3, 7.1 Hz, 18H).

^{13}C NMR (101 MHz, CDCl$_3$) δ 145.1, 144.1, 133.3, 129.9, 127.7, 124.6, 45.4, 42.8, 23.8, 21.6, 18.0, 17.9, 11.8. (Note: For dimethyl carbons in tri-*iso*propylsilyl group, NSi(CH(CH_3)$_2$)$_3$, 2 peaks were found instead of 1 due to the loss of symmetry caused by chiral sulfur atom.)

M.P. : 78-80 °C (CH_2Cl_2)

HRMS (ESI) calcd. for $C_{21}H_{36}N_2O_3S_2SiNa^+$ [M+Na]$^+$: 479.1829; found: 479.1829.

IR (ATR) (ν_{max} cm^{-1}) = 2942, 2865, 1596, 1460, 1339, 1164, 925, 877, 704.

Also prepared on gram scale: An oven-dried 100 mL round-bottom flask containing a magnetic stirring bar was charged with Pd(OAc)$_2$ (56.1 mg, 0.25 mmol, 5 mol%), PAd$_2$Bn (196.3 mg, 0.50 mmol. 10 mol%), HCO$_2$K (504.7 mg, 6.00 mmol, 1.20 equiv.), 1-tosyl-1,2,3,6-

tetrahydropyridin-4-yl trifluoromethanesulfonate (1.93 g, 5.00 mmol, 1.00 equiv.). The flask was then sealed with a suba seal and subjected to three evacuation/argon refill cycles. TIPS-NSO (1.32 g, 6.00 mmol, 1.20 equiv.) and anhydrous 1,4-dioxane (25.0 mL, 0.2 M). The resulting mixture was then put into a 75 °C oil bath with vigorous stirring (500-800 rpm) for 20 h. The resulting mixture was cooled to room temperature, diluted with EtOAc (15 mL), and washed with brine solution (50 mL). After separating the organic phase, the aqueous phase was extracted with EtOAc (20 ×2 mL). The combined organic phase were dried over anhydrous Na_2SO_4 and concentrated in *vacuo* and purified by column chromatography (5:1 to 3:1 to 2:1 Petrol ether: ethyl acetate) yielded title compound **3a** (2.02 g, 88%) as a brown solid.

Tert-butyl 4-(((triisopropylsilyl)amino)sulfinyl)-3,6-dihydropyridine-1(2H)-carboxylate (3b)

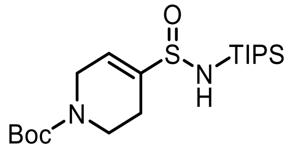

Synthesised according to **general procedure C**, using $Pd(OAc)_2$ (4.5 mg, 0.02 mmol, 10 mol%), PAd_2Bn (15.7 mg, 0.04 mmol. 20 mol%), HCO_2K (16.8 mg, 0.20 mmol, 1.00 equiv.), tert-butyl 4-(((trifluoromethyl)sulfonyl)oxy)-3,6-dihydropyridine-1(2H)-carboxylate (66.3 mg, 0.20 mmol, 1.00 equiv.), TIPS-NSO (52.7 mg, 0.24 mmol, 1.20 equiv.) and 1,4-dioxane (1.0 mL). Purification of the crude residue using column chromatography (5:1 to 3:1 Petrol ether: ethyl acetate) yielded title compound **3b** as a colourless oil (50.1 mg, 62%).

^{1}H NMR (400 MHz, $CDCl_3$) δ 6.44 (s, 1H), 4.16 (dq, *J* = 19.4, 2.8 Hz, 1H), 4.01 (dq, *J* = 19.3, 2.9 Hz, 1H), 3.81 (s, 1H), 3.36 (s, 2H), 2.49 – 2.38 (m, 1H), 2.31 – 2.19 (m, 1H), 1.47 (s, 9H), 1.24 – 1.15 (m, 3H), 1.12 – 1.07 (m, 18H).

^{13}C NMR (101 MHz, $CDCl_3$) δ 154.7, 145.2, 126.2, 80.3, 44.1, 40.8, 28.5, 23.7, 18.0, 17.9, 11.9. (Note: For dimethyl carbons in tri-*iso*propylsilyl group, NSi(CH(CH_3)$_2$)$_3$, 2 peaks were found instead of 1 due to the loss of symmetry caused by chiral sulfur atom.)

HRMS (ESI) calcd. for $C_{19}H_{39}N_2O_3SSi^+$ $[M+H]^+$: 403.2445; found: 403.2452.

IR (ATR) (ν_{max} cm^{-1}) =2966, 2867, 1701, 1647, 1463, 1423, 1392, 1334, 1172, 1116, 881, 735.

1-Benzyl-*N*-(triisopropylsilyl)-1,2,3,6-tetrahydropyridine-4-sulfinamide (3c)

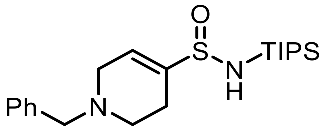

Synthesised according to **general procedure C**, using $Pd(OAc)_2$ (4.5 mg, 0.02 mmol, 10 mol%), PAd_2Bn (15.7 mg, 0.04 mmol. 20 mol%), HCO_2K (20.2 mg, 0.24 mmol, 1.20 equiv.), 1-benzyl-1,2,3,6-tetrahydropyridin-4-yl trifluoromethanesulfonate (64.3 mg, 0.20 mmol, 1.00 equiv.), TIPS-NSO (52.7 mg, 0.24 mmol, 1.20 equiv.) and 1,4-dioxane (1.0 mL). Purification of the crude residue using column chromatography (5:1 to 1:1 to 0:1 Petrol ether: ethyl acetate) yielded title compound **3c** (33.1 mg, 42%) as a brown oil.

^{1}H NMR (400 MHz, $CDCl_3$) δ 7.35 – 7.27 (m, 5H), 6.46 – 6.43 (m, 1H), 3.67 (d, *J* = 13.0 Hz, 1H), 3.62 (d, *J* = 13.0 Hz, 1H), 3.47 (s, 1H), 3.28 (dt, *J* = 17.7, 3.1 Hz, 1H), 3.13 (dq, *J* = 17.4, 2.9 Hz, 1H), 2.81 (dd, *J* = 10.6, 5.5 Hz, 1H), 2.60 – 2.43 (m, 2H), 2.30 – 2.20 (m, 1H), 1.25-1.14 (m, 3H), 1.09 (dd, *J* = 9.8, 7.1 Hz, 18H).

^{13}C NMR (101 MHz, $CDCl_3$) δ 144.6, 129.3, 128.5, 127.5, 62.3, 52.9, 49.3, 24.3, 18.02, 17.93, 11.9. (Note: i) Two carbon were missing from the phenyl ring. ii) For dimethyl carbons in tri-*iso*propylsilyl group, NSi(CH(CH_3)$_2$)$_3$, 2 peaks were found instead of 1 due to the loss of symmetry caused by chiral sulfur atom.)

HRMS (ESI) calcd. for $C_{21}H_{36}N_2OSSiNa^+$ $[M+H]^+$:415.2210; found: 415.2222.

IR (ATR) (ν_{max} cm $^{-1}$) = 3027, 2866, 1636, 1593, 1495, 1390, 1080, 1055, 921, 736, 700..

***N*-(Triisopropylsilyl)cyclopent-1-ene-1-sulfinamide (3d)**

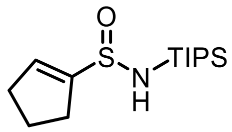

Synthesised according to **general procedure C**, using $Pd(OAc)_2$ (4.5 mg, 0.02 mmol, 10 mol%), PAd_2Bn (15.7 mg, 0.04 mmol. 20 mol%), HCO_2K (20.2 mg, 0.24 mmol, 1.20 equiv.), cyclopent-1-en-1-yl trifluoromethanesulfonate (43.4 mg, 0.20 mmol, 1.00 equiv.), TIPS-NSO (52.7 mg, 0.24 mmol, 1.20 equiv.) and 1,4-dioxane (1.0 mL). Purification of the crude residue using column chromatography (3:1 to 1:1 Petrol ether: ethyl acetate) yielded compound title **3d** (34.0 mg, 59%) as a brown solid.

^{1}H NMR (400 MHz, $CDCl_3$) δ 6.33 – 6.30 (m, 1H), 3.57 (s, 1H), 2.63 – 2.56 (m, 2H), 2.53 – 2.45 (m, 2H), 2.11 – 2.02 (m, 2H), 1.25 – 1.14 (m, 3H), 1.08 (dd, *J* = 9.5, 7.2 Hz, 18H).

^{13}C NMR (101 MHz, $CDCl_3$) δ 151.9, 134.8, 32.6, 29.7, 23.9, 17.99, 17.92, 11.8. (Note: For dimethyl carbons in tri-*iso*propylsilyl group, $NSi(CH(CH_3)_2)_3$, 2 peaks were found instead of 1 due to the loss of symmetry caused by chiral sulfur atom.)

M.P. : 79-80 ˚C (CH_2Cl_2)

HRMS (ESI) calcd. for $C_{14}H_{30}NOSSi^+$ $[M+H]^+$:288.1812; found: 288.1820.

IR (ATR) (ν_{max} cm $^{-1}$) = 2943, 2866, 1459, 1307, 1259, 1066, 1021, 898, 882, 766.

N-(Triisopropylsilyl)cyclohex-1-ene-1-sulfinamide (3e)

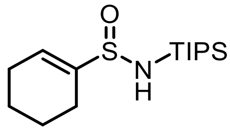

Synthesised according to **general procedure C**, using $Pd(OAc)_2$ (4.5 mg, 0.02 mmol, 10 mol%), PAd_2Bn (15.7 mg, 0.04 mmol. 20 mol%), HCO_2K (20.2 mg, 0.24 mmol, 1.20 equiv.), cyclohex-1-en-1-yl trifluoromethanesulfonate (46.0 mg, 0.20 mmol, 1.00 equiv.), TIPS-NSO (52.7 mg, 0.24 mmol, 1.20 equiv.) and 1,4-dioxane (1.0 mL). Purification of the crude residue using column chromatography (5:1 to 3:1 Petrol ether: ethyl acetate) yielded compound title **3e** (30.2 mg, 50%) as a brown solid.

^{1}H NMR (400 MHz, $CDCl_3$) δ 6.46 (dq, *J* = 3.6, 1.9 Hz, 1H), 3.35 (s, 1H), 2.34 – 2.10 (m, 4H), 1.82 – 1.55 (m, 4H), 1.21 – 1.14 (m, 3H), 1.09 (dd, *J* = 9.2, 7.0 Hz, 18H).

^{13}C NMR (101 MHz, $CDCl_3$) δ 146.3, 129.6, 25.4, 22.5, 22.13, 22.07, 18.03, 17.96, 11.9. (Note: For dimethyl carbons in tri-*iso*propylsilyl group, $NSi(CH(CH_3)_2)_3$, 2 peaks were found instead of 1 due to the loss of symmetry caused by chiral sulfur atom.)

M.P. : 63-64 °C (CH_2Cl_2)

HRMS (ESI) calcd. for $C_{15}H_{31}NOSSiNa^+$ $[M+H]^+$:324.1788; found: 324.1803.

IR (ATR) (ν_{max} cm $^{-1}$) = 2940, 2865, 1460, 1384, 1322, 1258, 1063, 879.

4,4-Difluoro-N-(triisopropylsilyl)cyclohex-1-ene-1-sulfinamide (3f)

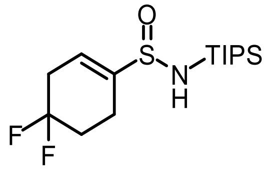

Synthesised according to **general procedure C**, using $Pd(OAc)_2$ (4.5 mg, 0.02 mmol, 10 mol%), PAd_2Bn (15.7 mg, 0.04 mmol. 20 mol%), HCO_2K (20.2 mg, 0.24 mmol, 1.20 equiv.), 4,4-difluorocyclohex-1-en-1-yl trifluoromethanesulfonate (53.2 mg, 0.20 mmol, 1.00 equiv.), TIPS-NSO (52.7 mg, 0.24 mmol, 1.20 equiv.) and 1,4-dioxane (1.0 mL). Purification of the crude residue using column chromatography (10:1 to 5:1 Petrol ether: ethyl acetate) yielded title compound **3f** (33.1 mg, 42%) as a brown solid.

^{1}H NMR (400 MHz, $CDCl_3$) δ 6.37 (td, *J* = 3.5, 1.7 Hz, 1H), 3.38 (s, 1H), 2.78 – 2.68 (m, 2H), 2.66 – 2.55 (m, 1H), 2.48-2.39 (m, 1H), 2.26 – 2.02 (m, 2H), 1.25 – 1.15 (m, 3H), 1.10 (dd, *J* = 8.7, 7.1 Hz, 18H).

^{13}C NMR (101 MHz, $CDCl_3$) δ 145.9, 124.5 (dd, *J* = 6.4, 4.3 Hz), 122.3 (t, *J* = 240.5 Hz), 34.9 (t, *J* = 27.7 Hz), 30.4 (t, *J* = 24.9 Hz), 21.2 (t, *J* = 5.6 Hz), 18.0, 17.9, 11.9. (Note: For dimethyl carbons in tri-*iso*propylsilyl group, NSi(CH(CH_3)$_2$)$_3$, 2 peaks were found instead of 1 due to the loss of symmetry caused by chiral sulfur atom.)

^{19}F NMR (377 MHz, $CDCl_3$) δ -94.70 – -98.84 (m).

M.P. : 59-60 ˚C (CH_2Cl_2)

HRMS (ESI) calcd. for $C_{15}H_{30}F_2NOSSi^+$ $[M+H]^+$:338.1780; found: 338.1779.

IR (ATR) (ν_{max} cm $^{-1}$) = 2944, 2867, 1463, 1372, 1352, 1259, 1144, 1119, 879.

4-(Tert-butyl)-*N*-(triisopropylsilyl)cyclohex-1-ene-1-sulfinamide (3g)

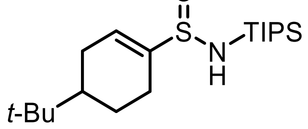

Synthesised according to **general procedure C**, using $Pd(OAc)_2$ (4.5 mg, 0.02 mmol, 10 mol%), PAd_2Bn (15.7 mg, 0.04 mmol. 20 mol%), HCO_2K (20.2 mg, 0.24 mmol, 1.20 equiv.),

4-(tert-butyl)cyclohex-1-en-1-yl trifluoromethanesulfonate (57.3 mg, 0.20 mmol, 1.00 equiv.), TIPS-NSO (52.7 mg, 0.24 mmol, 1.20 equiv.) and 1,4-dioxane (1.0 mL). Purification of the crude residue (1:1 dr) using column chromatography (10:1 to 5:1 Petrol ether: ethyl acetate) yielded title compound **3g** as an inseparable mixture of diasteroisomers as a brown oil (33.2 mg, 46%).

Major diastereomer:

^{1}H NMR (400 MHz, $CDCl_3$) δ 6.42 (dt, *J* = 5.0, 2.3 Hz, 1H), 3.41 (s, 1H), 2.56 (ddt, *J* = 16.9, 4.8, 2.4 Hz, 1H), 2.21 – 2.10 (m, 2H), 2.00 (dddd, *J* = 11.5, 9.5, 4.8, 3.0 Hz, 2H), 1.38 – 1.28 (m, 2H), 1.21 – 1.17 (m, 3H), 1.13 – 1.08 (m, 18H), 0.88 (s, 9H).

^{13}C NMR (101 MHz, $CDCl_3$) δ 146.5, 129.8, 43.8, 32.3, 27.3, 26.9, 24.9, 21.7, 17.98, 17.96, 11.9. (Note: For dimethyl carbons in tri-*iso*propylsilyl group, NSi(CH(*CH_3*)$_2$)$_3$, 2 peaks were found instead of 1 due to the loss of symmetry caused by chiral sulfur atom.)

HRMS (ESI) calcd. for $C_{19}H_{40}NOSSi^+$ $[M+H]^+$:358.2594; found: 358.2601.

IR (ATR) (ν_{max} cm $^{-1}$) = 2945, 2886, 1688, 1466, 1394, 1366, 1062, 881.

Minor diastereomer:

^{1}H NMR (400 MHz, $CDCl_3$) δ 6.49 (dt, *J* = 4.9, 2.2 Hz, 1H), 3.35 (s, 1H), 2.32 – 2.22 (m, 3H), 1.95 – 1.86 (m, 2H), 1.28 – 1.24 (m, 2H), 1.18 – 1.15 (m, 3H), 1.08 – 1.04 (m, 18H), 0.88 (s, 9H).

^{13}C NMR (101 MHz, $CDCl_3$) δ 145.7, 130.2, 44.2, 27.3, 27.3, 27.0, 24.2, 23.9, 17.98, 17.96, 11.9. (Note: For dimethyl carbons in tri-*iso*propylsilyl group, NSi(CH(*CH_3*)$_2$)$_3$, 2 peaks were found instead of 1 due to the loss of symmetry caused by chiral sulfur atom.)

***N*-(triisopropylsilyl)-3,4-dihydronaphthalene-1-sulfinamide (3h)**

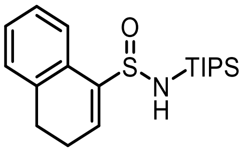

Synthesised according to **general procedure C**, using $Pd(OAc)_2$ (4.5 mg, 0.02 mmol, 10 mol%), PAd_2Bn (15.7 mg, 0.04 mmol. 20 mol%), HCO_2K (20.2 mg, 0.24 mmol, 1.20 equiv.), 3,4-dihydronaphthalen-1-yl trifluoromethanesulfonate (55.6 mg, 0.20 mmol, 1.00 equiv.), TIPS-NSO (52.7 mg, 0.24 mmol, 1.20 equiv.) and 1,4-dioxane (1.0 mL). Purification of the crude residue using column chromatography (10:1 to 5:1 to 3:1 Petrol ether: ethyl acetate) yielded title compound **3h** (46.8 mg, 67%) as a brown solid.

^{1}H NMR (400 MHz, $CDCl_3$) δ 7.62 – 7.56 (m, 1H), 7.22 – 7.17 (m, 3H), 6.88 (dd, *J* = 5.2, 4.2 Hz, 1H), 3.71 (s, 1H), 2.79 (dd, *J* = 9.2, 7.2 Hz, 2H), 2.58 – 2.35 (m, 2H), 1.26 – 1.15 (m, 3H), 1.08 (dd, *J* = 10.2, 7.2 Hz, 18H).

^{13}C NMR (101 MHz, $CDCl_3$) δ 146.9, 136.5, 129.6, 129.6, 128.2, 128.1, 126.5, 123.6, 27.7, 23.4, 18.07, 18.01, 12.0. (Note: For dimethyl carbons in tri-*iso*propylsilyl group, $NSi(CH(CH_3)_2)_3$, 2 peaks were found instead of 1 due to the loss of symmetry caused by chiral sulfur atom.)

M.P. : 46-49 °C (CH_2Cl_2)

HRMS (ESI) calcd. for $C_{19}H_{32}NOSSi^+$ $[M+H]^+$:350.1968; found: 350.1977.

IR (ATR) (ν_{max} cm $^{-1}$) = 2966, 2891, 1486, 1466, 1424, 1258, 1118, 881, 766, 732.

***N*-(Triisopropylsilyl)bicyclo[2.2.1]hept-2-ene-2-sulfinamide (3i)**

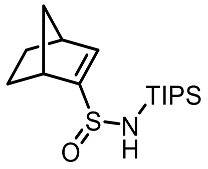

Synthesised according to **general procedure C**, using $Pd(OAc)_2$ (4.5 mg, 0.02 mmol, 10 mol%), PAd_2Bn (15.7 mg, 0.04 mmol. 20 mol%), HCO_2K (20.2 mg, 0.24 mmol, 1.20 equiv.), bicyclo[2.2.1]hept-2-en-2-yl trifluoromethanesulfonate (48.4 mg, 0.20 mmol, 1.00 equiv.), TIPS-NSO (52.7 mg, 0.24 mmol, 1.20 equiv.) and 1,4-dioxane (1.0 mL). Purification of the crude residue (1:1 dr) using column chromatography (10:1 to 3:1 Petrol ether: ethyl acetate) yielded title compound **3i** as an inseparable mixture of diasteroisomers as a brown solid (30.0 mg, 48%).

Major diastereomer:

^{1}H NMR (400 MHz, $CDCl_3$) δ 6.49 (dd, J = 3.2, 1.0 Hz, 1H), 3.62 (s, 1H), 3.18 (dt, J = 3.1, 1.5 Hz, 1H), 3.05 (d, J = 1.5 Hz, 1H), 1.77 – 1.71 (m, 2H), 1.59 (ddt, J = 8.5, 4.3, 2.2 Hz, 1H), 1.30 – 1.23 (m, 3H), 1.20 – 1.16 (m, 3H), 1.09 (ddd, J = 8.5, 7.1, 3.5 Hz, 18H).

^{13}C NMR (101 MHz, $CDCl_3$) δ 155.8, 138.3, 49.4, 43.0, 42.1, 25.5, 25.0, 18.02, 17.93, 11.8. (Note: For dimethyl carbons in tri-*iso*propylsilyl group, $NSi(CH(CH_3)_2)_3$, 2 peaks were found instead of 1 due to the loss of symmetry caused by chiral sulfur atom.)

M.P. : 58-60 ˚C (CH_2Cl_2)

HRMS (ESI) calcd. for $C_{16}H_{32}NOSSi^+$ $[M+H]^+$:314.1968; found: 314.1986.

IR (ATR) (ν_{max} cm $^{-1}$) =2944, 2867, 1462, 1385, 1058, 879, 754.

Minor diastereomer:

^{1}H NMR (400 MHz, $CDCl_3$) δ 6.46 (dt, *J* = 3.2, 1.0 Hz, 1H), 3.55 (s, 1H), 3.27 (dd, *J* = 3.1, 1.7 Hz, 1H), 3.08 – 3.06 (m, 1H), 1.78 – 1.72 (m, 2H), 1.58 – 1.55 (m, 1H), 1.25 – 1.22 (m, 3H), 1.16 – 1.13 (m, 3H), 1.08 – 1.06 (m, 18H).

^{13}C NMR (101 MHz, $CDCl_3$) δ 155.8, 137.8, 49.6, 43.4, 41.8, 25.7, 25.0, 17.9, 17.8, 11.7. (Note: For dimethyl carbons in tri-*iso*propylsilyl group, NSi(CH(*CH_3*)$_2$)$_3$, 2 peaks were found instead of 1 due to the loss of symmetry caused by chiral sulfur atom.)

***(1S,4R)*-1,7,7-Trimethyl-N-(triisopropylsilyl)bicyclo[2.2.1]hept-2-ene-2-sulfinamide (3j)**

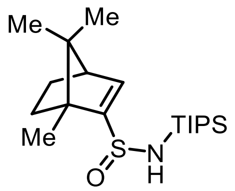

Synthesised according to **general procedure C**, using $Pd(OAc)_2$ (4.5 mg, 0.02 mmol, 10 mol%), PAd_2Bn (15.7 mg, 0.04 mmol. 20 mol%), HCO_2K (20.2 mg, 0.24 mmol, 1.20 equiv.), *(1S,4R)*-1,7,7-trimethylbicyclo[2.2.1]hept-2-en-2-yl trifluoromethanesulfonate (56.9 mg, 0.20 mmol, 1.00 equiv.), TIPS-NSO (52.7 mg, 0.24 mmol, 1.20 equiv.) and 1,4-dioxane (1.0 mL). Purification of the crude residue (3:1 dr) using column chromatography (10:1 to 5:1 to 3:1 Petrol ether: ethyl acetate) yielded title compound **3j** as an inseparable mixture of diasteroisomers as a brown solid (60.3 mg, 85%).

Major diastereomer:

^{1}H NMR (400 MHz, $CDCl_3$) δ 6.42 (d, *J* = 3.4 Hz, 1H), 3.61 (d, *J* = 4.2 Hz, 1H), 2.48 (dd, *J* = 4.6, 2.7 Hz, 1H), 1.95 – 1.84 (m, 1H), 1.67-1.61 (m, 1H), 1.38-1.32 (m, 1H), 1.23 (s, 3H), 1.20 – 1.14 (m, 3H), 1.11 – 1.06 (m, 18H), 0.82 (s, 3H), 0.79 (s, 3H).

^{13}C NMR (101 MHz, $CDCl_3$) δ 155.2, 135.7, 58.4, 55.2, 52.0, 32.3, 24.4, 19.20, 19.16, 17.92, 17.85, 11.8. (Note: For dimethyl carbons in tri-*iso*propylsilyl group, NSi(CH(CH_3)$_2$)$_3$, 2 peaks were found instead of 1 due to the loss of symmetry caused by chiral sulfur atom.)

HRMS (ESI) calcd. for $C_{19}H_{38}NOSSi^+$ $[M+H]^+$:356.2438; found: 356.2421.

IR (ATR) (ν_{max} cm $^{-1}$) =.2958, 2945, 1687, 1474, 1388, 1366, 1288, 1077, 882, 733.

Minor diastereomer:

^{1}H NMR (400 MHz, $CDCl_3$) δ 6.48 (d, *J* = 3.5 Hz, 1H), 3.62 (s, 1H), 2.50 – 2.49 (m, 1H), 1.97 – 1.92 (m, 1H), 1.71 – 1.68 (m, 1H), 1.33 – 1.30 (m, 1H), 1.26 (s, 3H), 1.22 – 1.20 (m, 3H), 1.12 – 1.09 (m, 18H), 0.84 (s, 3H), 0.79 (s, 3H).

^{13}C NMR (101 MHz, $CDCl_3$) δ 154.5, 137.1, 58.7, 55.6, 52.1, 32.7, 24.9, 19.4, 19.1, 17.92, 17.86, 11.8. (Note: For dimethyl carbons in tri-*iso*propylsilyl group, NSi(CH(CH_3)$_2$)$_3$, 2 peaks were found instead of 1 due to the loss of symmetry caused by chiral sulfur atom.)

(3*S*,8*R*,9*S*,10*R*,13*S*,14*S*)-10,13-Dimethyl-17-(((triisopropylsilyl)amino)sulfinyl)-2,3,4,7,8,9,10,11,12,13,14,15-dodecahydro-1H-cyclopenta[a]phenanthren-3-yl acetate (3k)

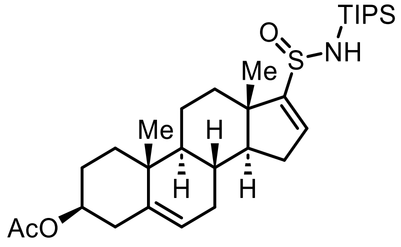

Synthesised according to **general procedure C**, using $Pd(OAc)_2$ (4.5 mg, 0.02 mmol, 10 mol%), PAd_2Bn (15.7 mg, 0.04 mmol. 20 mol%), HCO_2K (20.2 mg, 0.24 mmol, 1.20 equiv.), (3S,8R,9S,10R,13S,14S)-10,13-dimethyl-17-(((trifluoromethyl)sulfonyl)oxy)-2,3,4,7,8,9,10,11,12,13,14,15-dodecahydro-1H-cyclopenta[a]phenanthren-3-yl acetate (92.5 mg, 0.20 mmol, 1.00 equiv.), TIPS-NSO (52.7 mg, 0.24 mmol, 1.20 equiv.) and 1,4-dioxane

(1.0 mL). Purification of the crude residue (5:1 dr) using column chromatography (5:1 to 3:1 Petrol ether: ethyl acetate) yielded title compound **3k** as an inseparable mixture of diasteroisomers as a brown solid (61.5 mg, 58%).

^{1}H NMR (400 MHz, $CDCl_3$) δ 6.32 (dd, *J* = 3.2, 1.7 Hz, 1H), 5.37 (dt, *J* = 3.6, 1.7 Hz, 1H), 4.59 (tdd, *J* = 10.3, 6.1, 4.2 Hz, 1H), 3.65 (s, 1H), 2.35 – 2.25 (m, 3H), 2.09 – 2.04 (m, 2H), 2.02 (s, 3H), 1.90 – 1.76 (m, 2H), 1.74 – 1.51 (m, 8H), 1.26 – 1.19 (m, 3H), 1.18 – 1.17 (m, 1H), 1.16 – 1.14 (m, 1H), 1.12 – 1.08 (m, 18H), 1.05 (s, 3H), 1.04 (s, 3H).

^{13}C NMR (101 MHz, $CDCl_3$) δ 170.6, 162.5, 140.2, 134.1, 122.1, 73.9, 58.0, 50.3, 47.2, 38.2, 37.0, 36.9, 35.5, 31.4, 31.37, 30.39, 27.8, 21.5, 20.7, 19.3, 18.1, 18.0, 17.6, 11.2. (Note: For dimethyl carbons in tri-*iso*propylsilyl group, $NSi(CH(CH_3)_2)_3$, 2 peaks were found instead of 1 due to the loss of symmetry caused by chiral sulfur atom.)

HRMS (ESI) calcd. for $C_{30}H_{52}NO_3SSi^+$ $[M+H]^+$: 534.3432; found: 534.3451.

IR (ATR) (ν_{max} cm^{-1}) =2945, 2867, 1733, 1467, 1374, 1250, 1066, 1016, 923, 882, 733.

3.3 Derivatizations

Methyl 3-(aminosulfinyl)benzoate 4a

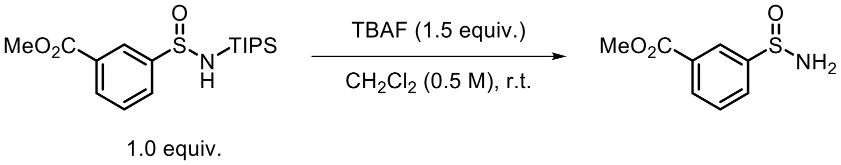

Methyl 3-(((triisopropylsilyl)amino)sulfinyl)benzoate (355.6 mg, 1.0 mmol, 1.0 equiv.) was added to a 25 mL round bottom flask. Then CH_2Cl_2 (2 mL, 0.5 M) was added, followed by addition of TBAF (1.5 mL, 1 M in THF, 1.5 mmol, 1.5 equiv.). After 30 min, the reaction was concentrated in *vacuo* and purified by column chromatography (3:1 to 1:1 petrol ether: ethyl acetate) to yield the desired product **4a** as a white solid (199.1 mg, 99%).

^{1}H NMR (400 MHz, $CDCl_3$) δ 8.39 – 8.33 (m, 1H), 8.14 (dt, *J* = 7.7, 1.4 Hz, 1H), 7.92 (ddd, *J* = 7.8, 1.9, 1.2 Hz, 1H), 7.57 (td, *J* = 7.8, 0.5 Hz, 1H), 4.62 (s, 2H), 3.93 (s, 3H).

^{13}C NMR (101 MHz, $CDCl_3$) δ 166.1, 147.3, 132.2, 131.1, 129.9, 129.2, 126.9, 52.6.

M.P. : 115-117 ˚C (CH_2Cl_2)

HRMS (ESI) calcd. for $C_8H_{10}NO_3S^+$ $[M+H]^+$: 200.0376; found: 200.0381.

IR (ATR) (ν_{max} cm $^{-1}$) = 3312, 1709, 1433, 1284, 1273, 1127, 1060, 1045, 890, 755.

Methyl 3-(morpholine-4-sulfonimidoyl)benzoate 4b

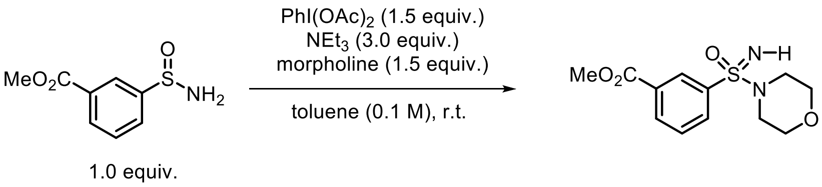

Methyl 3-(aminosulfinyl)benzoate (39.8 mg, 0.2 mmol, 1.0 equiv.) and $PhI(OAc)_2$ (96.6 mg, 0.3 mmol, 1.5 equiv.) were added to an oven-dried 25 mL round bottom flask. The flask was sealed and subjected to three Ar evacuation/refill cycles before anhydrous toluene (2 mL, 0.1

M) was added. Triethylamine (83.6 μL, 0.6 mmol, 3.0 equiv.) was added to the solution, followed by immediate addition of morpholine (26.1 μL, 0.3 mmol, 1.5 equiv.). After 4 hours, the crude mixture was diluted by 10 mL ethyl acetate, filtered through a pad of silica (1.5 to 2 cm) washing with EtOAc (40 mL). The resulting crude mixture was concentrated in *vacuo* and purified by column chromatography (ethyl acetate) yielded the desired product **4b** as a colourless oil (48.5 mg, 85%).

^{1}H NMR (400 MHz, $CDCl_3$) δ 8.49 (td, J = 1.8, 0.5 Hz, 1H), 8.25 (dt, J = 7.9, 1.4 Hz, 1H), 8.05 (ddd, J = 7.9, 1.9, 1.2 Hz, 1H), 7.62 (td, J = 7.8, 0.5 Hz, 1H), 3.95 (s, 3H), 3.74 – 3.67 (m, 4H), 3.03 – 2.96 (m, 4H), 2.63 (s, 1H).

^{13}C NMR (101 MHz, $CDCl_3$) δ 165.7, 136.1, 133.6, 132.3, 131.3, 129.3, 129.2, 66.5, 52.7, 47.2.

HRMS (ESI) calcd. for $C_{12}H_{17}N_2O_4S^+$ $[M+H]^+$: 285.0904; found: 285.0908.

IR (ATR) (ν_{max} cm $^{-1}$) = 2955, 2857, 1726, 1438, 1297, 1260, 1260, 1141, 1112, 1079, 971, 931, 754, 704.

Methyl 3-(*N'*-(triisopropylsilyl)sulfamidimidoyl)benzoate 4c'

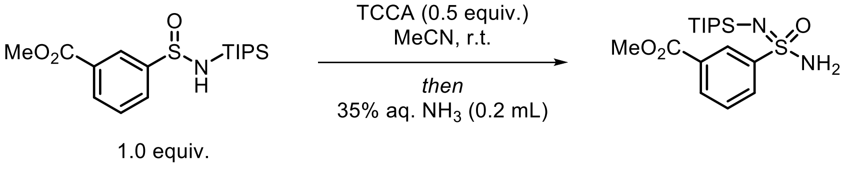

Methyl 3-(((triisopropylsilyl)amino)sulfinyl)benzoate (71.1 mg, 0.20 mmol, 1.0 equiv.) and trichloroisocyanuric acid (23.2 mg, 0.10 mmol, 0.5 equiv.) was added to a 25 mL round-bottom flask. Anhydrous acetonitrile (2.0 mL, 0.1 M) was added at room temperature, and the reaction was stirred for 20 min. 35% aq NH_3 (0.2 mL) was added and the reaction was stirred at room temperature for 1 h. After completion of the reaction (as judged by TLC), the resulting mixture

was diluted with EtOAc (5 mL), filtered through a pad of silica (1.5 to 2 cm) washing with EtOAc (40 mL). The resulting mixture was concentrated in *vacuo* and purified by column chromatography (5:1 to 3:1 petrol ether: ethyl acetate) to afford the desired product **4c'** as a white solid (67.0 mg, 90%).

^{1}H NMR (400 MHz, $CDCl_3$) δ 8.65 (t, J = 1.8 Hz, 1H), 8.20 – 8.10 (m, 2H), 7.54 (t, J = 7.8 Hz, 1H), 4.71 (s, 2H), 3.93 (s, 3H), 1.18 – 1.01 (m, 21H).

^{13}C NMR (101 MHz, $CDCl_3$) δ 166.0, 147.9, 132.3, 130.9, 130.1, 129.1, 127.3, 52.6, 18.4, 13.1.

M.P.: 110-112 ˚C (CH_2Cl_2)

HRMS (ESI) calcd. for $C_{17}H_{31}N_2O_3SSi^+$ $[M+H]^+$: 371.1819; found: 371.1838.

IR (ATR) (ν_{max} cm $^{-1}$) = 3343, 3228, 2960, 2863, 1718, 1391, 1297, 1262, 967, 815, 680.

Methyl 3-sulfamidimidoylbenzoate 4c

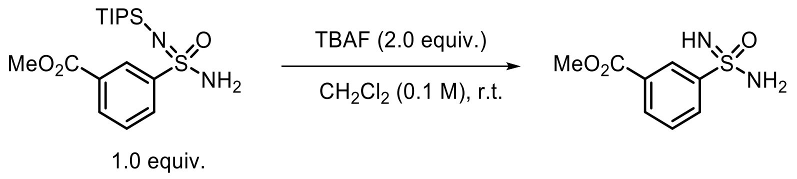

Methyl 3-(N'-(triisopropylsilyl)sulfamidimidoyl)benzoate (37.0 mg, 0.10 mmol, 1.0 equiv.) was added to a 10 mL vial containing a stir bar and dissolved with CH_2Cl_2 (1.0 mL, 0.1 M). Tetrabutylammonium fluoride solution (0.20 mL, 1.0 M in THF, 0.20 mmol, 2.0 equiv.) was added and the reaction was stirred at room temperature for 4 h. After completion (as judged by TLC), the reaction was concentrated under reduced pressure and purified by column chromatography (1:1 to 0:1 petrol ether: ethyl acetate) to afford the desired product **4c** as a white solid (16.4 mg, 77%).

^{1}H NMR (400 MHz, $(CD_3)_2SO$) δ 8.50 (td, *J* = 1.8, 0.5 Hz, 1H), 8.15 (ddd, *J* = 7.9, 2.0, 1.2 Hz, 1H), 8.09 (ddd, *J* = 7.8, 1.7, 1.2 Hz, 1H), 7.68 (td, *J* = 7.8, 0.5 Hz, 1H), 6.42 (br s, 2H), 3.90 (s, 3H). [N-H proton not detected]

^{13}C NMR (101 MHz, $(CD_3)_2SO$) δ 165.4, 147.5, 131.4, 130.5, 129.9, 129.3, 126.7, 52.5.

M.P. : 99-100 ˚C (CH_2Cl_2)

HRMS (ESI) calcd. for $C_8H_{10}N_2O_3SNa^+$ $[M+Na]^+$: 237.0304; found: 237.0308.

IR (ATR) (ν_{max} cm $^{-1}$) = 3300, 2955, 1728, 1600, 1578, 1440, 1305, 1270, 1166, 1135, 996, 737.

Methyl 3-(*N*-(p-tolyl)-*N'*-(triisopropylsilyl)sulfamidimidoyl)benzoate 4d'

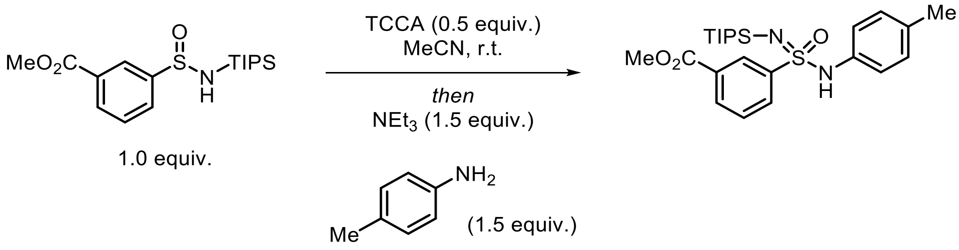

Methyl 3-(((triisopropylsilyl)amino)sulfinyl)benzoate (71.1 mg, 0.20 mmol, 1.0 equiv.) and trichloroisocyanuric acid (23.2 mg, 0.10 mmol, 0.5 equiv.) were added to a 25 mL round-bottom flask. Anhydrous acetonitrile (2.0 mL, 0.1 M) was added at room temperature, and the reaction was stirred for 20 min. Triethylamine (33.4 µL, 0.24 mmol, 1.2 equiv.) and *p*-toluidine (32.1 mg, 0.30 mmol, 1.5 equiv.) were added and the reaction was stirred at room temperature for 1 h. After completion of the reaction (as judged by TLC), the resulting mixture was diluted with EtOAc (5 mL), filtered through a pad of silica (1.5 to 2 cm) washing with EtOAc (40 mL). The resulting crude mixture was concentrated in *vacuo* and purified by column chromatography (20:1 to 10:1 petrol ether: ethyl acetate) yielded the desired product **4d'** as a yellow oil (84.5 mg, 91%).

^{1}H NMR (400 MHz, $CDCl_3$) δ 8.53 (t, *J* = 1.8 Hz, 1H), 8.08 (dt, *J* = 7.8, 1.4 Hz, 1H), 7.90 (ddd, *J* = 7.9, 2.0, 1.2 Hz, 1H), 7.42 (t, *J* = 7.8 Hz, 1H), 7.01 – 6.92 (m, 2H), 6.91 – 6.83 (m, 2H), 6.40 (s, 1H), 3.90 (s, 3H), 2.23 (s, 3H), 1.22 – 1.06 (m, 21H).

^{13}C NMR (101 MHz, $CDCl_3$) δ 166.0, 144.6, 135.8, 134.3, 132.4, 131.1, 130.8, 129.6, 128.7, 128.3, 121.8, 52.5, 20.9, 18.4, 13.2.

HRMS (ESI) calcd. for $C_{24}H_{37}N_2O_3SSi^+$ $[M+H]^+$: 461.2289; found: 461.2307.

IR (ATR) (ν_{max} cm $^{-1}$) = 3310, 2962, 2865, 1731, 1711, 1615, 1464, 1439, 1339, 1219, 1152, 996, 917, 776, 681.

Methyl 3-(*N*-(p-tolyl)sulfamidimidoyl)benzoate 4d

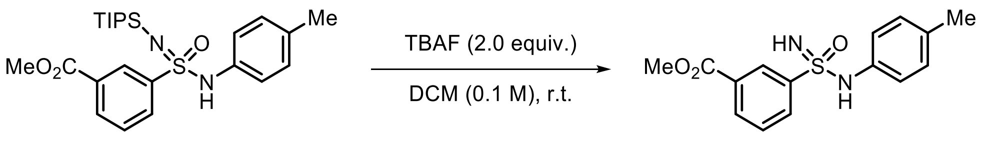

1.0 equiv.

Methyl 3-(*N*-(*p*-tolyl)-*N'*-(triisopropylsilyl)sulfamidimidoyl)benzoate (78.3 mg, 0.17 mmol, 1.0 equiv.) was added to a 10 mL vial containing a stir bar and dissolved with CH_2Cl_2 (2.0 mL, 0.1 M). Tetrabutylammonium fluoride solution (0.34 mL, 1.0 M in THF, 0.34 mmol, 2.0 equiv.) was added and the reaction was stirred at room temperature for 4 h. After completion (as judged by TLC), the reaction was concentrated in *vacuo* and purified by column chromatography (3:1 to 1:1 petrol ether: ethyl acetate) to afford the desired product **4d** as a yellow solid (48.7 mg, 80%, 0.16 mmol).

^{1}H NMR (400 MHz, $CDCl_3$) δ 8.64 (t, *J* = 1.8 Hz, 1H), 8.17 (dt, *J* = 7.9, 1.4 Hz, 1H), 8.12 (ddd, *J* = 7.9, 2.0, 1.2 Hz, 1H), 7.52 (t, *J* = 7.9 Hz, 1H), 7.03 – 6.93 (m, 4H), 4.54 (s, 2H), 3.92 (s, 3H), 2.25 (s, 3H).

^{13}**C NMR** (101 MHz, $CDCl_3$) δ 165.8, 142.2, 137.4, 133.8, 133.4, 131.5, 131.3, 129.9, 129.2, 128.5, 123.3, 52.7, 20.9.

M.P. : 87-88 ˚C (CH_2Cl_2)

HRMS (ESI) calcd. for $C_{15}H_{17}N_2O_3S^+$ $[M+H]^+$: 305.0954; found: 305.0956.

IR (ATR) (ν_{max} cm $^{-1}$) = 3300, 2953, 1728, 1507, 1439, 1301, 1164, 1131, 1091, 911, 753, 732.

Methyl 3-(*S*-fluoro-*N*-(triisopropylsilyl)sulfonimidoyl)benzoate 4e

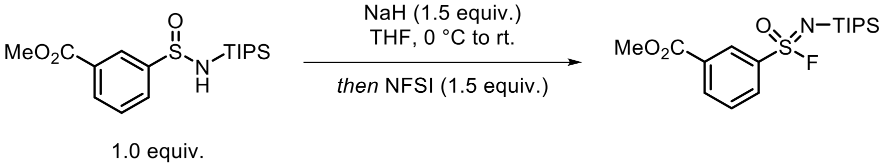

Methyl 3-(((triisopropylsilyl)amino)sulfinyl)benzoate (0.2 mmol, 71.1 mg, 1.0 equiv.) and NaH (12.0 mg, 0.3 mmol, 1.5 equiv., 60% in mineral oil) were added to an oven-dried 25 mL round bottom flask, sealed, evacuated and back-filled with Ar (×3). Then anhydrous THF (2 mL, 0.1 M) were added at 0 ˚C. The reaction was stirred for 5 mins before warmed to room temperature and stirred for another 25 min. NFSI (94.6 mg, 0.3 mmol, 1.5 equiv.) was added and the reaction was stirred at room temperature for 30 min. After reaction completion (as judged by TLC), the resulting mixture was diluted with EtOAc (5 mL), filtered through a pad of silica (1.5 to 2 cm) washing with EtOAc (40 mL). The resulting crude mixture was concentrated in *vacuo* and purified by column chromatography (20:1 to 10:1 petrol ether: ethyl acetate) yielded the desired product **4e** as a colourless oil (67.5 mg, 90%).

^{1}**H NMR** (400 MHz, $CDCl_3$) δ 8.69 (t, *J* = 1.8 Hz, 1H), 8.29 (dt, *J* = 7.8, 1.4 Hz, 1H), 8.19 (ddd, *J* = 7.9, 2.0, 1.2 Hz, 1H), 7.64 (t, *J* = 7.9 Hz, 1H), 3.96 (s, 3H), 1.25 – 1.17 (m, 3H), 1.14 – 1.10 (m, 18H).

^{13}C NMR (101 MHz, $CDCl_3$) δ 165.4, 139.5 (d, J = 34.8 Hz), 134.4, 131.7, 131.4, 129.5, 129.1, 52.8, 18.03, 18.02, 12.7 (d, J = 1.8 Hz). (Note: For dimethyl carbons in tri-*iso*propylsilyl group, NSi(CH(CH_3)$_2$)$_3$, 2 peaks were found instead of 1 due to the loss of symmetry caused by chiral sulfur atom.)

^{19}F NMR (377 MHz, $CDCl_3$) δ 92.73 (s).

HRMS (ESI) calcd. for $C_{17}H_{28}FNO_3SSiNa^+$ $[M+H]^+$:396.1435; found: 396.1443.

IR (ATR) (ν_{max} cm^{-1}) = 2963, 2945, 1735, 1466, 1440, 1300, 1270, 1221, 1129, 859, 738, 682..

Methyl 3-(*N*-(triisopropylsilyl)sulfamoyl)benzoate 4f'

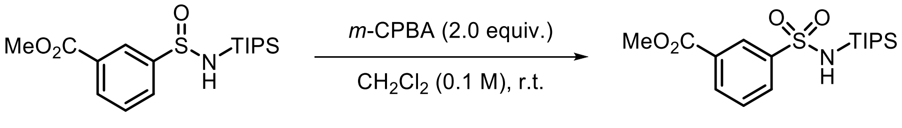

Methyl 3-(((triisopropylsilyl)amino)sulfinyl)benzoate (0.4 mmol, 142.2 mg, 1.0 equiv.) and *meta*-chloroperoxybenzoic acid (60% by weight) (0.8 mmol, 179.3 mg, 2.0 equiv.) were added to a 25 mL round bottom flask. CH_2Cl_2 (2 mL, 0.1 M) was added at room temperature. The reaction was stirred at room temperature for 30 min. Then the reaction was diluted by 30 mL ethyl acetate and quenched by 30 mL aq. $Na_2S_2O_3$. After collecting the organic phase, the aqueous phase was extracted with ethyl acetate (2 × 10 mL). The combined organic phases were dried over Na_2SO_4, filtered and concentrated in *vacuo*. The resulting crude residue was purified by column chromatography (10:1 to 5:1 petrol ether: ethyl acetate) to yield the desired product **4f'** as a white solid (155.1 mg, 99%).

^{1}H NMR (400 MHz, $CDCl_3$) δ 8.57 (t, J = 1.8 Hz, 1H), 8.15 (dt, J = 7.8, 1.4 Hz, 1H), 8.09 (ddd, J = 7.9, 2.0, 1.1 Hz, 1H), 7.52 (t, J = 7.8 Hz, 1H), 4.97 (s, 1H), 3.89 (s, 3H), 1.24 (tt, J = 8.4, 6.9 Hz, 3H), 1.00 (d, J = 7.5 Hz, 18H).

^{13}**C NMR** (101 MHz, $CDCl_3$) δ 165.7, 144.5, 132.8, 131.0, 130.2, 129.1, 127.3, 52.6, 17.9, 11.9.

M.P. : 78-80 °C (CH_2Cl_2)

HRMS (ESI) calcd. for $C_{17}H_{30}NO_4SSi^+$ $[M+H]^+$: 372.1659; found: 372.1672.

IR (ATR) (ν_{max} cm $^{-1}$) = 2924, 2866, 1706, 1461, 1372, 1288, 1270, 1191, 977, 926, 757.

Methyl 3-sulfamoylbenzoate 4f

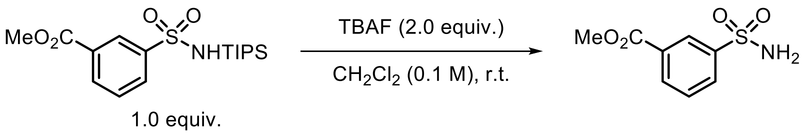

Methyl 3-(*N*-(triisopropylsilyl)sulfamoyl)benzoate (37.1 mg, 0.1 mmol, 1.0 equiv.) was added to a 25 mL round bottom flask. Then dichloromethane (1 mL, 0.1 M) was added, followed by addition of TBAF (0.2 mL, 1 M in THF, 0.2 mmol, 2.0 equiv.). After 30 min, the reaction was concentrated in *vacuo* and purified by column chromatography (3:1 to 1:1 petrol ether: ethyl acetate) to yield the desired product **4f** as a white solid (20.6 mg, 96%).

^{1}**H NMR** (400 MHz, $CDCl_3$) δ 8.58 (t, J = 1.8 Hz, 1H), 8.23 (dt, J = 7.8, 1.4 Hz, 1H), 8.11 (ddd, J = 7.9, 2.0, 1.2 Hz, 1H), 7.61 (t, J = 7.9 Hz, 1H), 5.19 (s, 2H), 3.95 (s, 3H).

^{13}**C NMR** (101 MHz, $CDCl_3$) δ 165.6, 142.7, 133.6, 131.3, 130.5, 129.4, 127.6, 52.7.

M.P. : 80-81 °C (CH_2Cl_2)

Data is consistent with the literature.[10]

1-Tosyl-1,2,3,6-tetrahydropyridine-4-sulfinamide (5a)

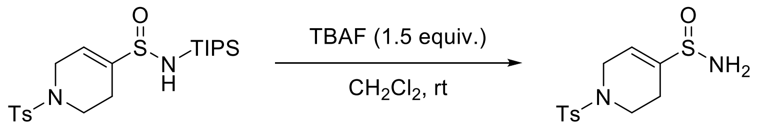

1-Tosyl-*N*-(triisopropylsilyl)-1,2,3,6-tetrahydropyridine-4-sulfinamide (**3a**) (137.0 mg, 0.30 mmol, 1.0 equiv.) was added to a 25 mL round bottom flask. Then CH_2Cl_2 (0.6 mL, 0.5 M) was added, followed by addition of TBAF (0.45 mL, 1 M in THF, 0.45 mmol, 1.5 equiv.). After 30 min, the reaction was concentrated in *vacuo* and purified by column chromatography (1:1 to 0:1 petrol ether: ethyl acetate) to yield the desired product **5a** as a white solid (76.2 mg, 85%).

^{1}H NMR (400 MHz, $(CD_3)_2SO$) δ 7.67 (d, J = 8.0 Hz, 2H), 7.45 (d, J = 7.9 Hz, 2H), 6.27 – 6.20 (m, 1H), 5.87 (s, 2H), 3.80 – 3.69 (m, 1H), 3.65 – 3.54 (m, 1H), 3.35 – 3.25 (m, 1H), 3.01 – 2.92 (m, 1H), 2.44 – 2.34 (m, 4H), 2.28 – 2.17 (m, 1H).

^{13}C NMR (101 MHz, $(CD_3)_2SO$) δ 143.8, 143.5, 132.6, 130.0, 127.4, 123.9, 45.1, 42.7, 23.4, 21.0.

M.P. : 147-148 ˚C (CH_2Cl_2)

HRMS (ESI) calcd. for $C_{12}H_{16}N_2O_3S_2Na^+$ $[M+Na]^+$: 323.0495; found: 323.0500.

IR (ATR) (ν_{max} cm $^{-1}$) = 3198, 2926, 1337, 1295, 1180, 1156, 1100, 1062, 1049, 931, 897, 780, 702.

***Tert*-butyl 4-(1-tosyl-1,2,3,6-tetrahydropyridine-4-sulfonimidoyl)piperazine-1-carboxylate (5b)**

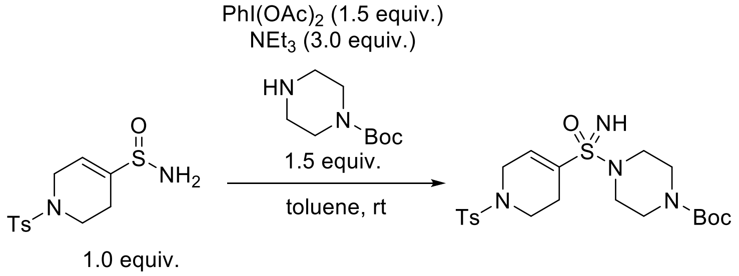

1-Tosyl-1,2,3,6-tetrahydropyridine-4-sulfinamide (**5a**) (45.0 mg, 0.15 mmol, 1.0 equiv.) and $PhI(OAc)_2$ (74.1 mg, 0.23 mmol, 1.5 equiv.) were added to an oven-dried 25 mL round bottom flask. The flask was sealed and subjected to three Ar evacuation/refill cycles before anhydrous toluene (1.5 mL, 0.1 M) was added. Triethylamine (63 µL, 0.45 mmol, 3.0 equiv.) was added to the solution, followed by immediate addition of *tert*-butyl piperazine-1-carboxylate (42.8 mg, 0.23 mmol, 1.5 equiv.). After 1 hour, the crude mixture was diluted by 10 mL ethyl acetate, filtered through a pad of silica (1.5 to 2 cm) washing with EtOAc (40 mL). The resulting crude mixture was concentrated in *vacuo* and purified by column chromatography (1:1 to 0:1 petrol ether: ethyl acetate) yielded the desired product **5b** as a white solid (47.2 mg, 65%).

^{1}H NMR (400 MHz, $CDCl_3$) δ 7.66 (d, J = 8.0 Hz, 2H), 7.33 (d, J = 8.0 Hz, 2H), 6.67 (dd, J = 4.1, 2.3 Hz, 1H), 3.97 – 3.85 (m, 1H), 3.76 – 3.67 (m, 1H), 3.51 – 3.35 (m, 5H), 3.12 – 3.00 (m, 5H), 2.67 – 2.57 (m, 1H), 2.50 – 2.39 (m, 4H), 2.31 (s, 1H), 1.45 (s, 9H).

^{13}C NMR (101 MHz, $CDCl_3$) δ 154.4, 144.3, 135.8, 133.1, 132.1, 130.1, 127.7, 80.6, 46.5, 44.8, 43.7, 42.6, 28.5, 26.0, 21.7.

M.P. : 155-157 ˚C (CH_2Cl_2)

HRMS (ESI) calcd. for $C_{21}H_{32}N_4O_5S_2Na^+$ $[M+Na]^+$: 507.1706; found: 507.1717.

IR (ATR) (ν_{max} cm $^{-1}$) = 3303, 2980, 1693, 1598, 1422, 1365, 1307, 1281, 1166, 1127, 917, 732.

1-Tosyl-*N*-(triisopropylsilyl)-1,2,3,6-tetrahydropyridine-4-sulfonimidoyl fluoride (5c)

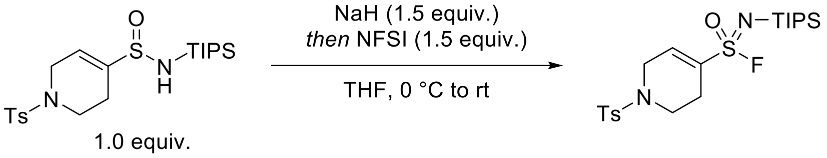

1-Tosyl-*N*-(triisopropylsilyl)-1,2,3,6-tetrahydropyridine-4-sulfinamide (**3a**) (91.3 mg, 0.20 mmol, 1.0 equiv.) and NaH (12.0 mg, 0.3 mmol, 1.5 equiv., 60% in mineral oil) were added to an oven-dried 25 mL round bottom flask, sealed, evacuated and back-filled with Ar (×3). Then anhydrous THF (2 mL, 0.1 M) were added at 0 ˚C. The reaction was stirred for 5 mins before warmed to room temperature and stirred for another 25 min. NFSI (94.6 mg, 0.30 mmol, 1.5 equiv.) was added and the reaction was stirred at room temperature for 30 min. After reaction completion (as judged by TLC), the resulting mixture was diluted with EtOAc (5 mL), filtered through a pad of silica (1.5 to 2 cm) washing with EtOAc (40 mL). The resulting crude mixture was concentrated in *vacuo* and purified by column chromatography (20:1 to 10:1 petrol ether: ethyl acetate) yielded the desired product **5c** as a white solid (82.0 mg, 86%).

^{1}H NMR (400 MHz, $CDCl_3$) δ 7.67 (d, *J* = 8.4 Hz, 2H), 7.33 (d, *J* = 8.0 Hz, 2H), 6.83 (tt, *J* = 3.4, 1.6 Hz, 1H), 3.97 – 3.87 (m, 1H), 3.84 – 3.76 (m, 1H), 3.39 (dt, *J* = 11.4, 5.5 Hz, 1H), 3.23 (dt, *J* = 12.0, 5.8 Hz, 1H), 2.68 – 2.59 (m, 2H), 2.43 (s, 3H), 1.14 – 0.99 (m, 21H).

^{13}C NMR (101 MHz, $CDCl_3$) δ 144.4, 137.0 (d, *J* = 35.1 Hz), 133.1, 132.7, 130.1, 127.7, 44.8, 42.3, 24.9, 21.7, 17.98, 17.96, 12.6 (d, *J* = 1.6 Hz). (Note: For dimethyl carbons in tri-*iso*propylsilyl group, NSi(CH(CH_3)$_2$)$_3$, 2 peaks were found instead of 1 due to the loss of symmetry caused by chiral sulfur atom.)

^{19}**F NMR** (377 MHz, $CDCl_3$) δ 78.00 (s).

M.P. : 75-78 ˚C (CH_2Cl_2)

HRMS (ESI) calcd. for $C_{21}H_{35}FN_2O_3S_2SiNa^+$ $[M+Na]^+$: 497.1735; found: 497.1748.

IR (ATR) (ν_{max} cm $^{-1}$) = 2945, 1418, 1350, 1218, 1168, 910, 883, 733, 672.

4. References

(1) Oliver, G. A.; Loch, M. N.; Augustin, A. U.; Steinbach, P.; Sharique, M.; Tambar, U. K.; Jones, P. G.; Bannwarth, C.; Werz, D. B. Cycloadditions of Donor–Acceptor Cyclopropanes and -butanes using S=N-Containing Reagents: Access to Cyclic Sulfinamides, Sulfonamides, and Sulfinamidines. *Angew. Chem. Int. Ed.* **2021**, *60*, 25825-25831.

(2) Ding, M.; Zhang, Z.-X.; Davies, T. Q.; Willis, M. C. A Silyl Sulfinylamine Reagent Enables the Modular Synthesis of Sulfonimidamides via Primary Sulfinamides. *Org Lett* **2022**, *24*, 1711-1715.

(3) Zhang, Z.-X.; Bell, C.; Ding, M.; Willis, M. C. Modular Two-Step Route to Sulfondiimidamides. *J Am Chem Soc* **2022**, *144*, 11851-11858.

(4) Zhang, Z.-X.; Davies, T. Q.; Willis, M. C. Modular Sulfondiimine Synthesis Using a Stable Sulfinylamine Reagent. *J Am Chem Soc* **2019**, *141*, 13022-13027.

(5) Davies, T. Q.; Hall, A.; Willis, M. C. One-Pot, Three-Component Sulfonimidamide Synthesis Exploiting the Sulfinylamine Reagent N-Sulfinyltritylamine, TrNSO. *Angew. Chem. Int. Ed.* **2017**, *56*, 14937-14941.

(6) Lou, T. S.-B.; Bagley, S. W.; Willis, M. C. Cyclic Alkenylsulfonyl Fluorides: Palladium-Catalyzed Synthesis and Functionalization of Compact Multifunctional Reagents. *Angew. Chem. Int. Ed.* **2019**, *58*, 18859-18863.

(7) Xu, Y.; Su, T.; Zhang, J.-R.; Ding, H.-J.; Gao, Y.; Xu, M.; Cao, P.; Hu, P.; Wang, B.-Q.; Chen, B. Nickel-Catalyzed Reductive Three-Component Cross-Coupling of Butadiene with Aldehydes and Alkenyl Triflates/Bromides: Access to Skipped Dienes. *Org. Let.* **2023**, *25*, 3136-3140.

(8) Tessier, P. E.; Nguyen, N.; Clay, M. D.; Fallis, A. G. Aryl Annulation of Cyclic Ketones via a Magnesium Carbometalation−6-π- Electrocyclization Protocol. *Org. Let.* **2005**, *7*, 767-770.

(9) Fehl, C.; Vogt, C. D.; Yadav, R.; Li, K.; Scott, E. E.; Aubé, J. Structure-Based Design of Inhibitors with Improved Selectivity for Steroidogenic Cytochrome P450 17A1 over Cytochrome P450 21A2. *J. Med. Chem.* **2018**, *61*, 4946-4960.

(10) Wang, M.; Fan, Q.; Jiang, X. Metal-free construction of primary sulfonamides through three diverse salts. *Green Chem.* **2018**, *20*, 5469-5473.

5. NMR spectra

4-Fluoro-*N*-(triisopropylsilyl)benzenesulfinamide (1a)

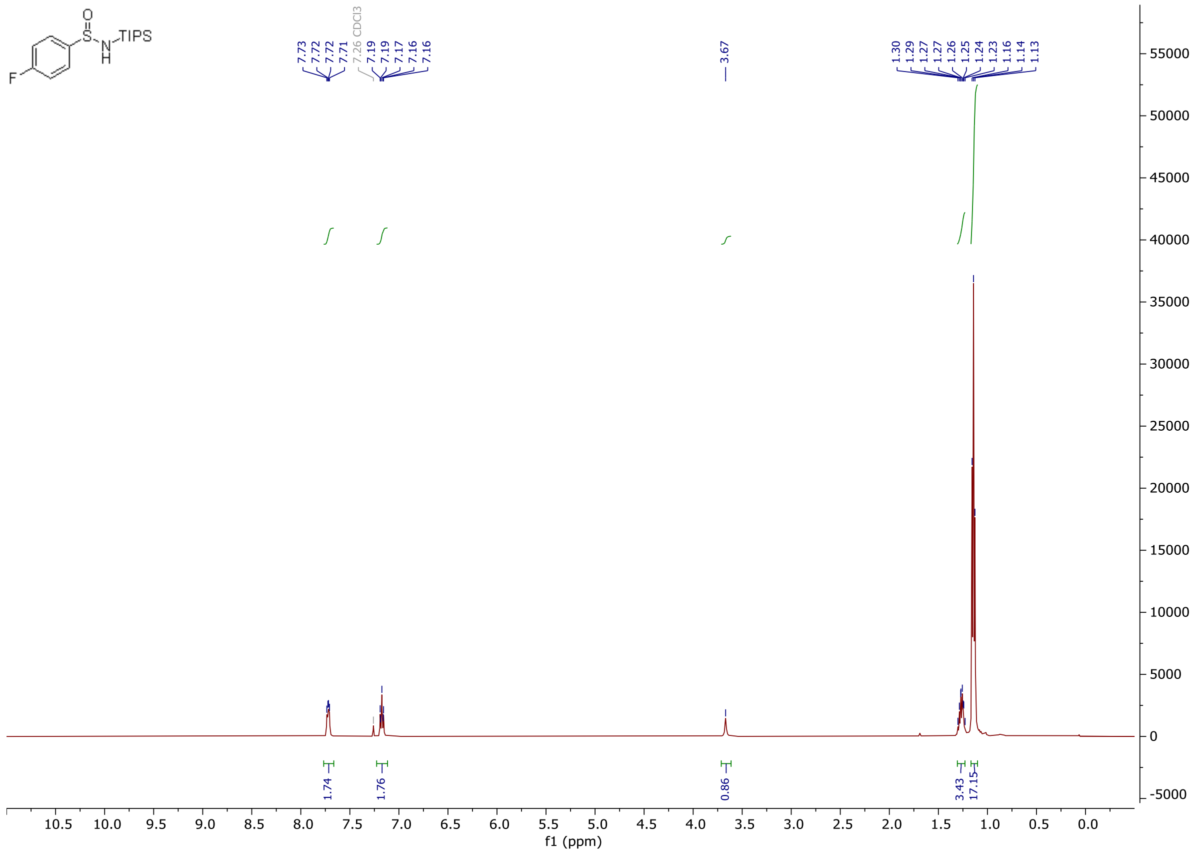

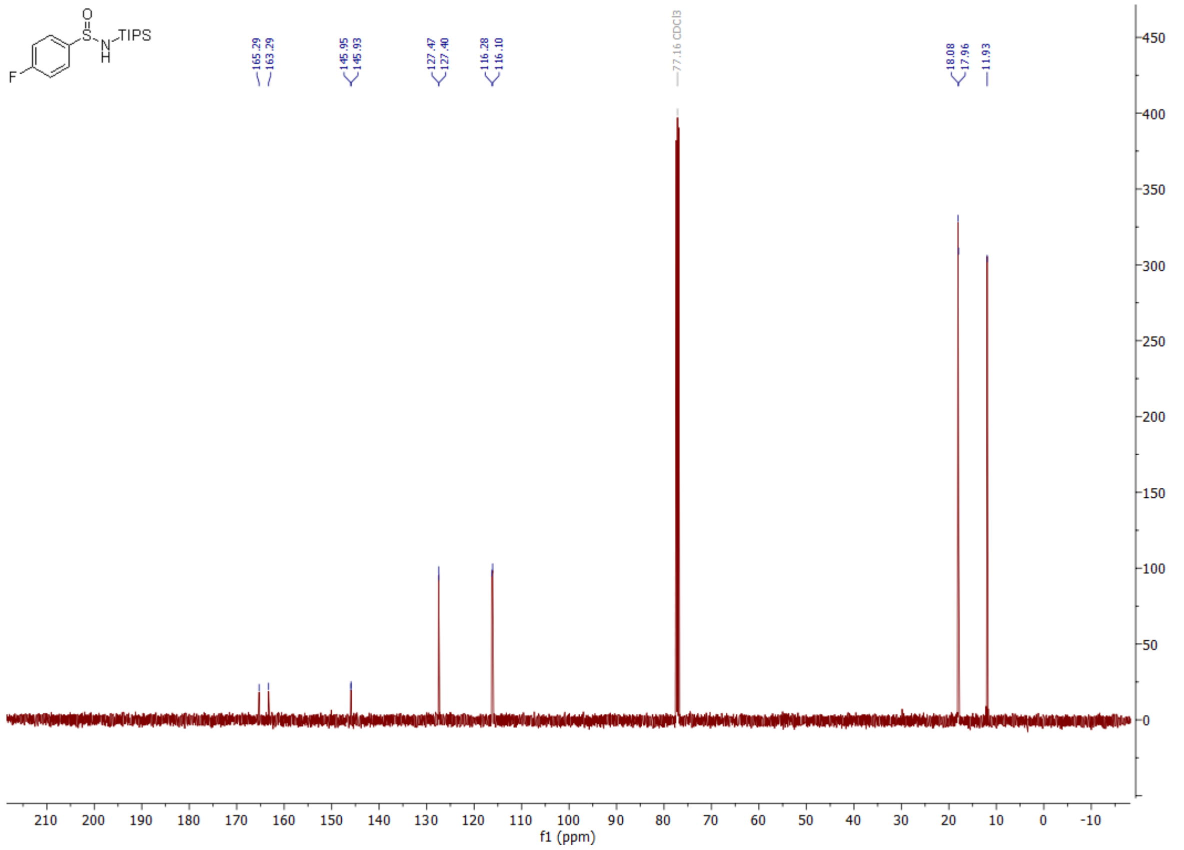

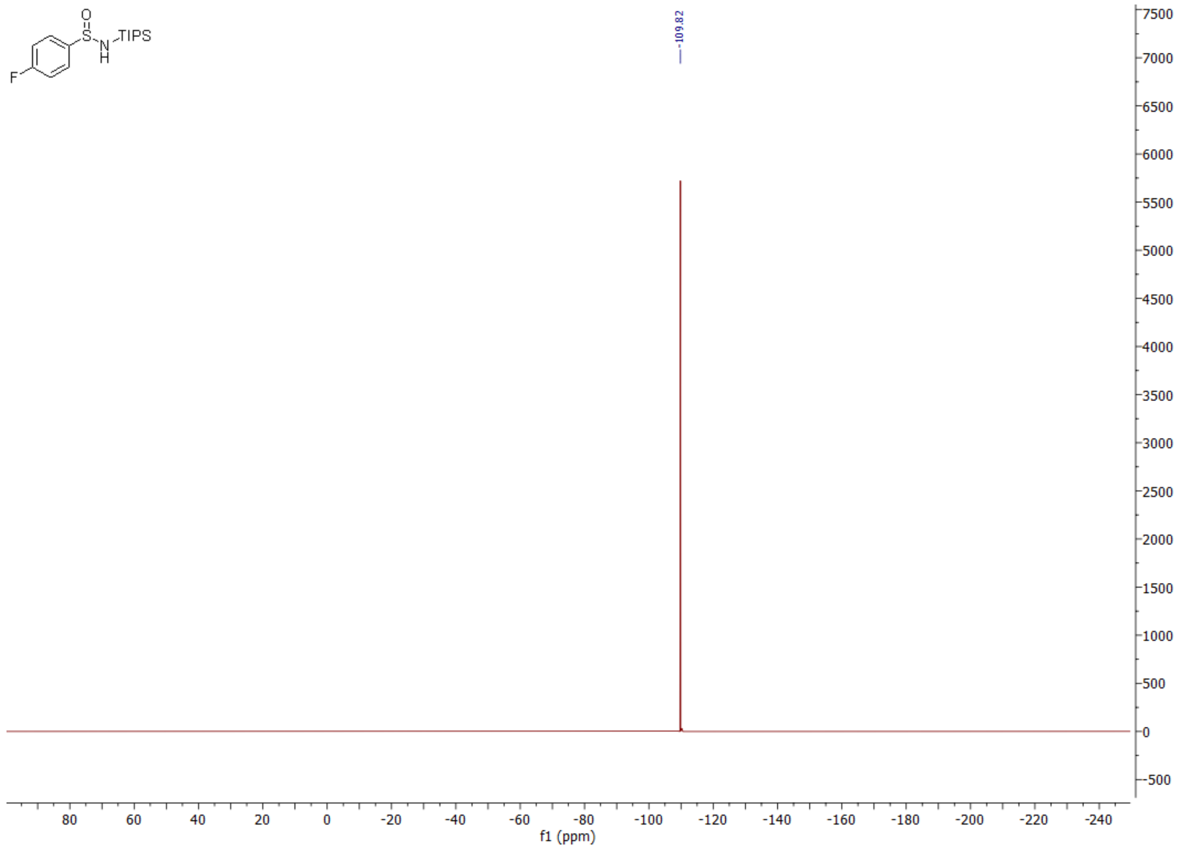

4-chloro-*N*-(triisopropylsilyl)benzenesulfinamide (1b)

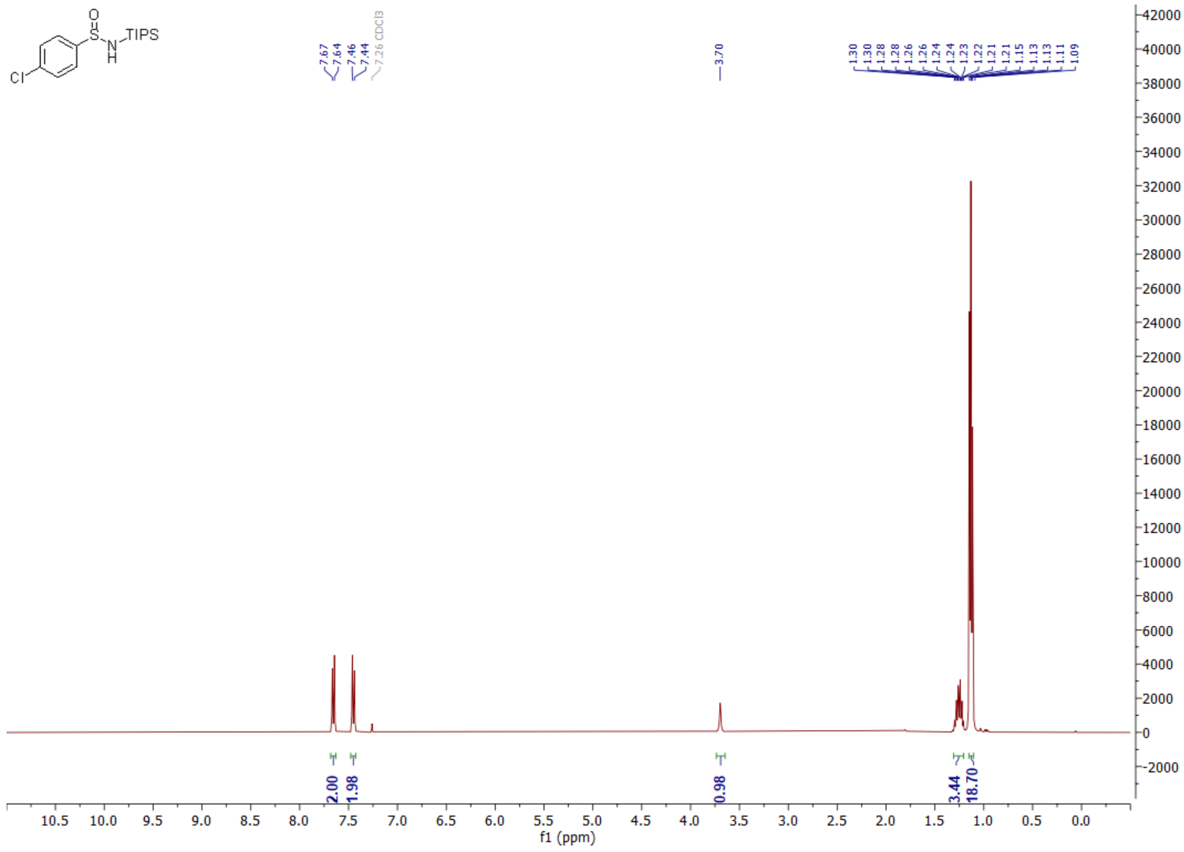

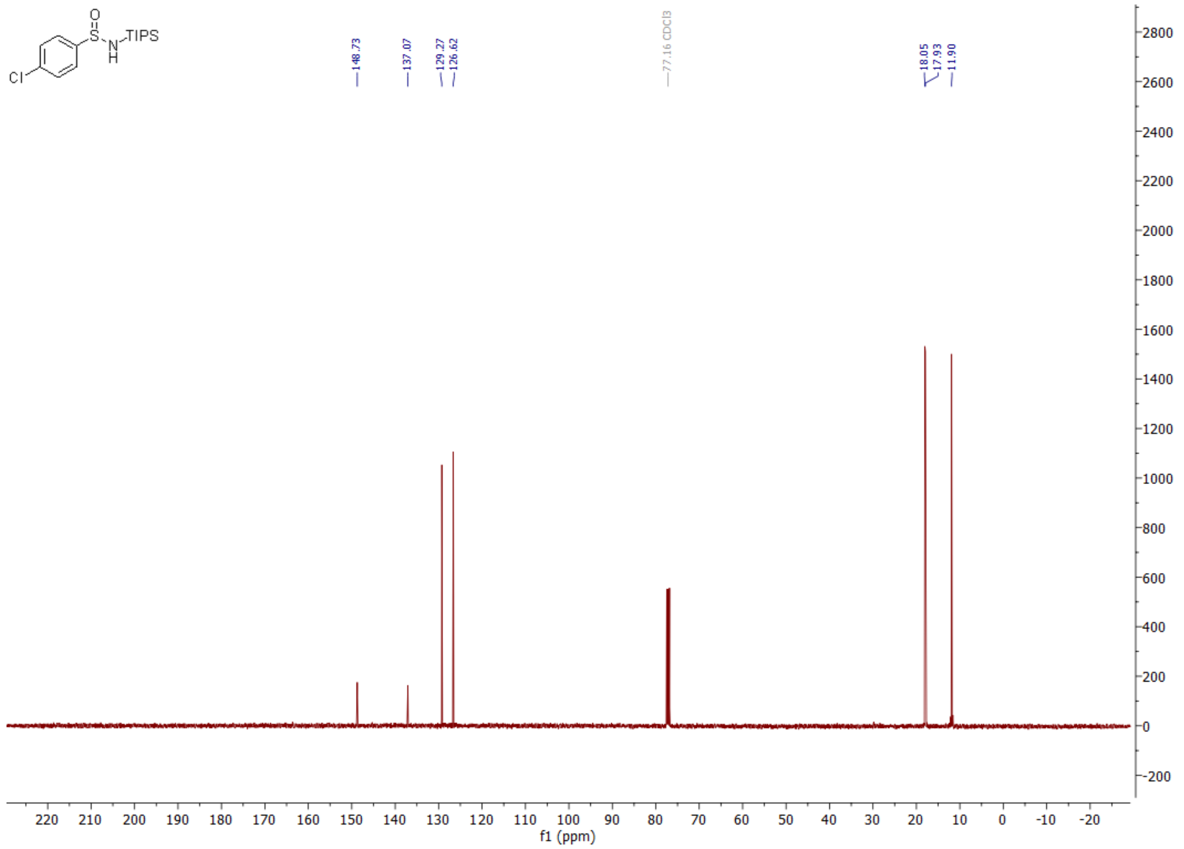

4-Nitro-*N*-(triisopropylsilyl)benzenesulfinamide (1c)

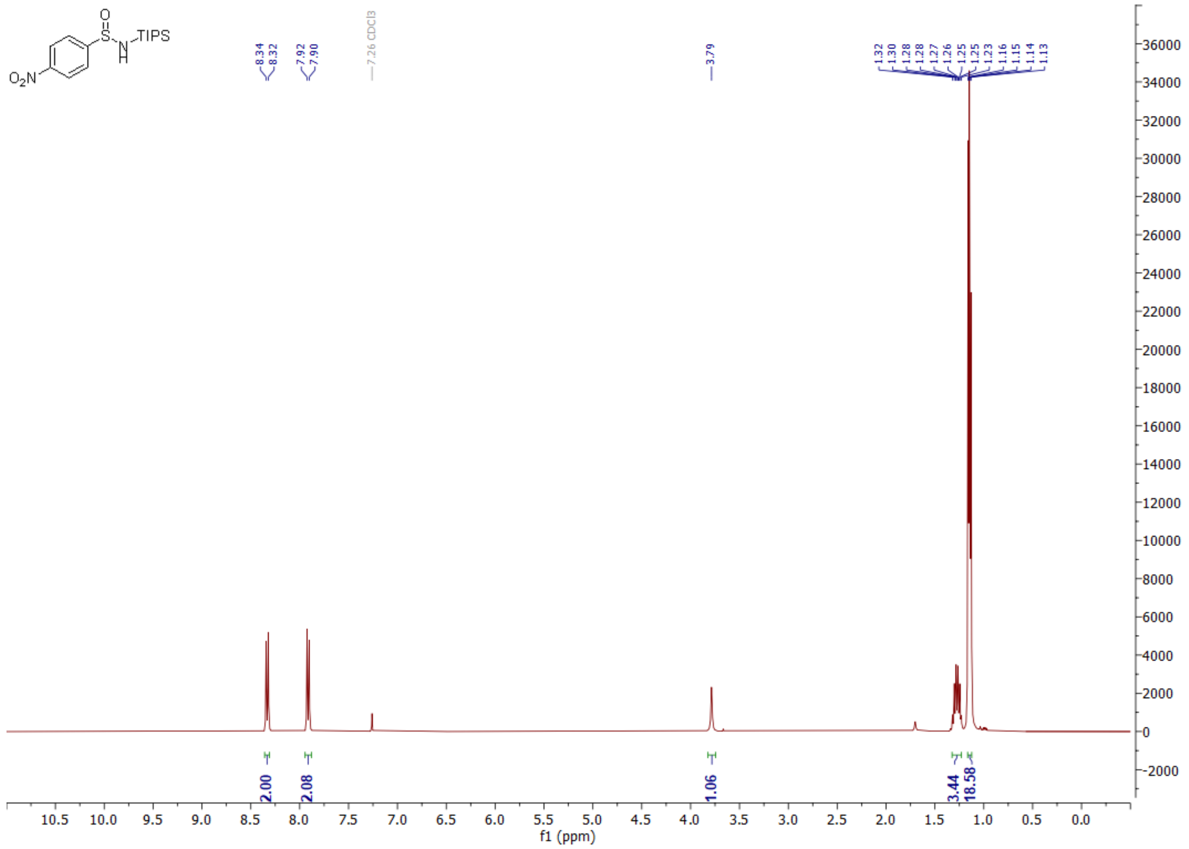

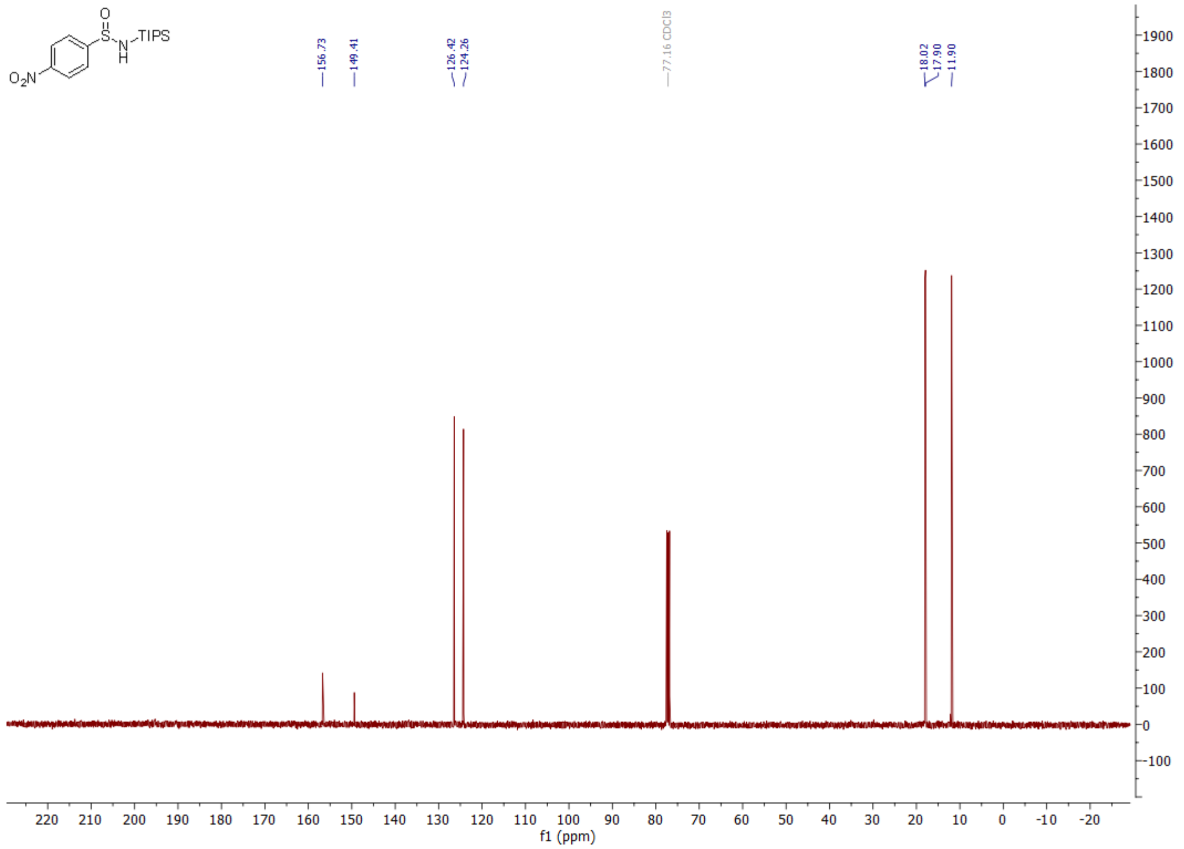

4-Cyano-*N*-(triisopropylsilyl)benzenesulfinamide (1d)

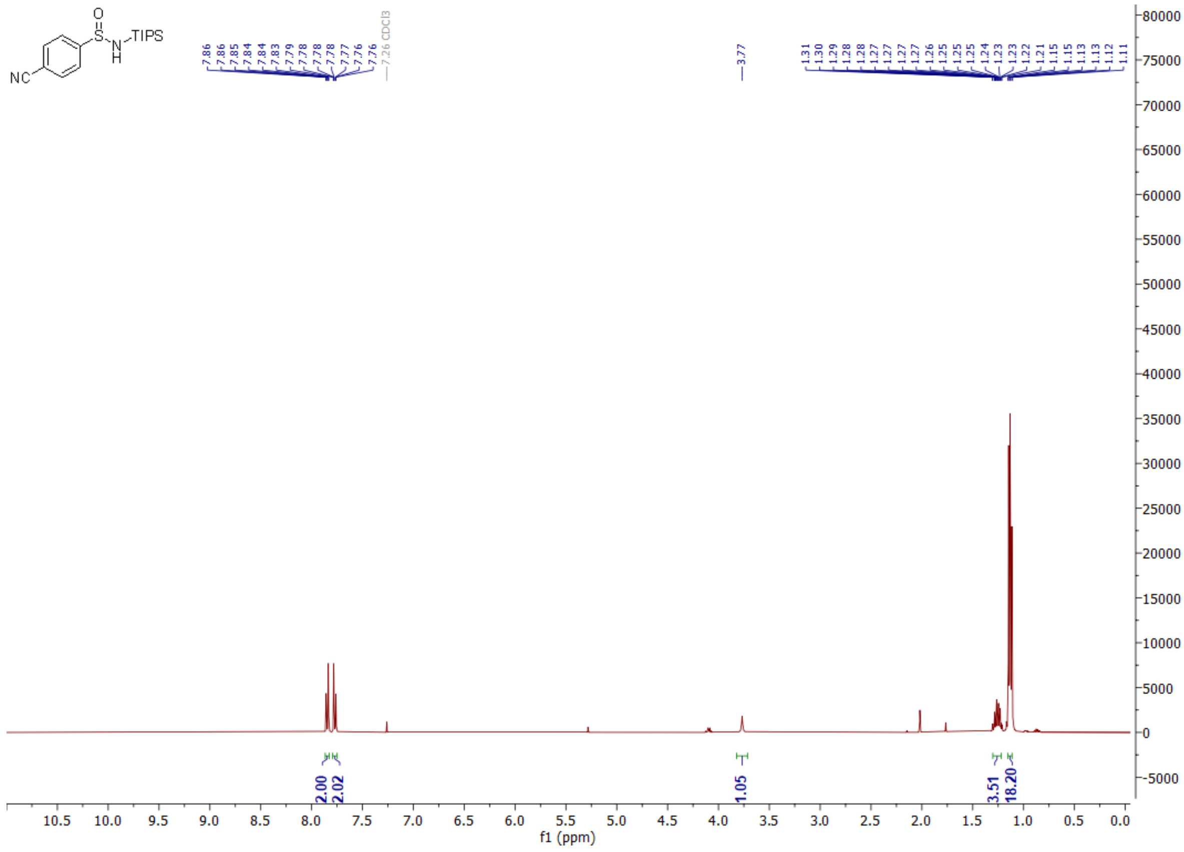

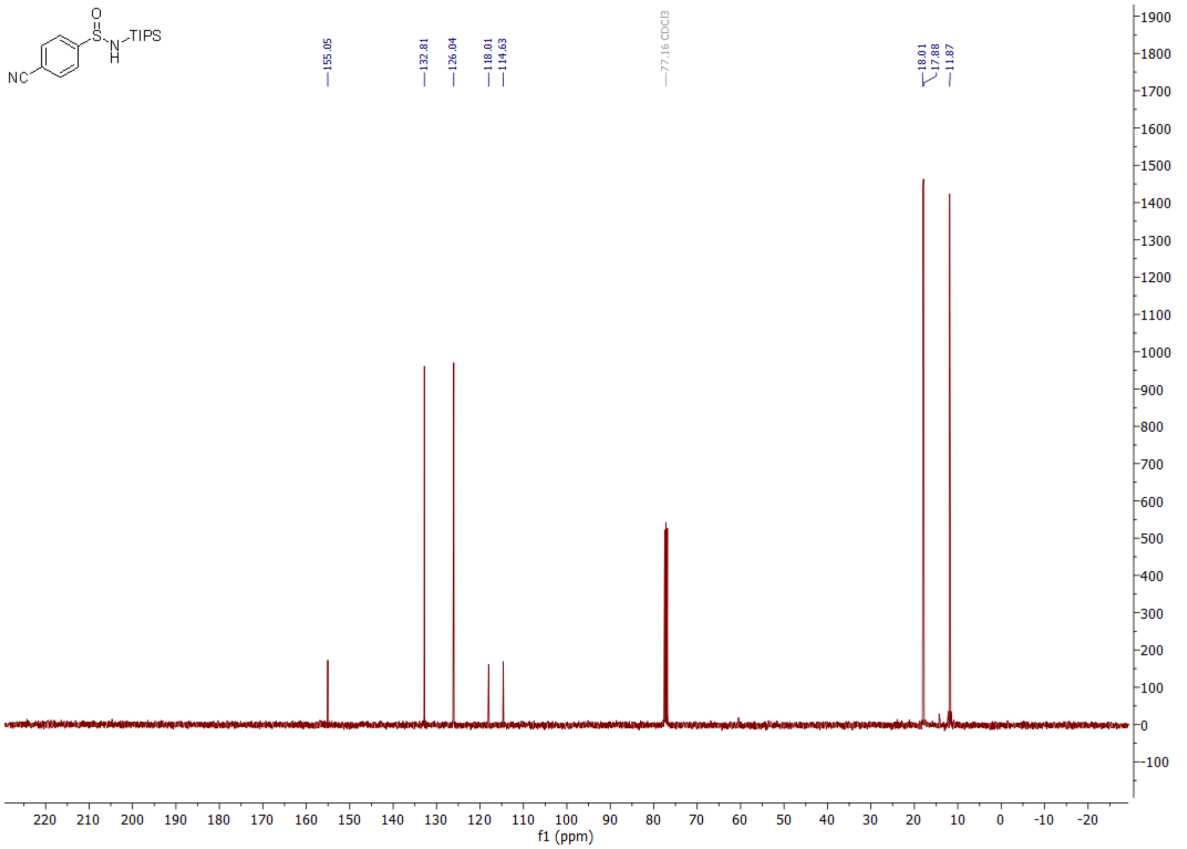

4-Acetyl-*N*-(triisopropylsilyl)benzenesulfinamide (1e)

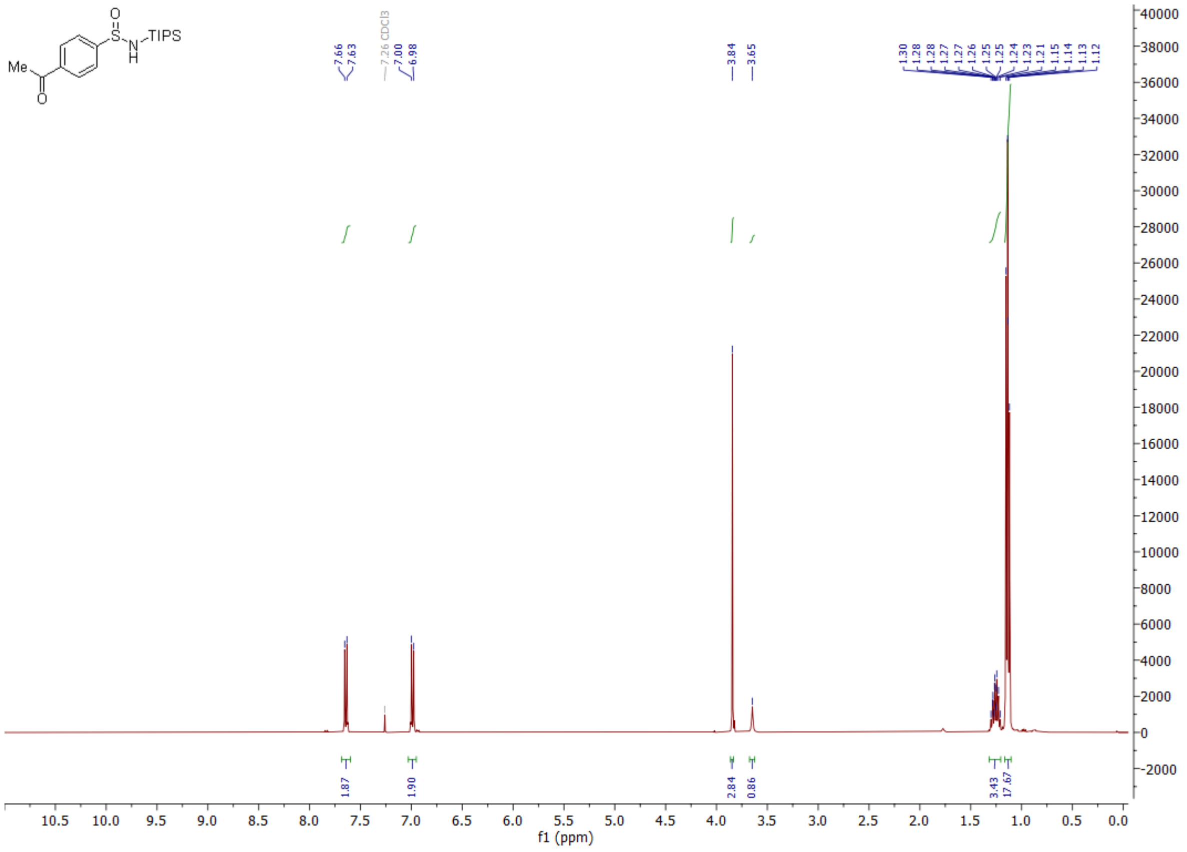

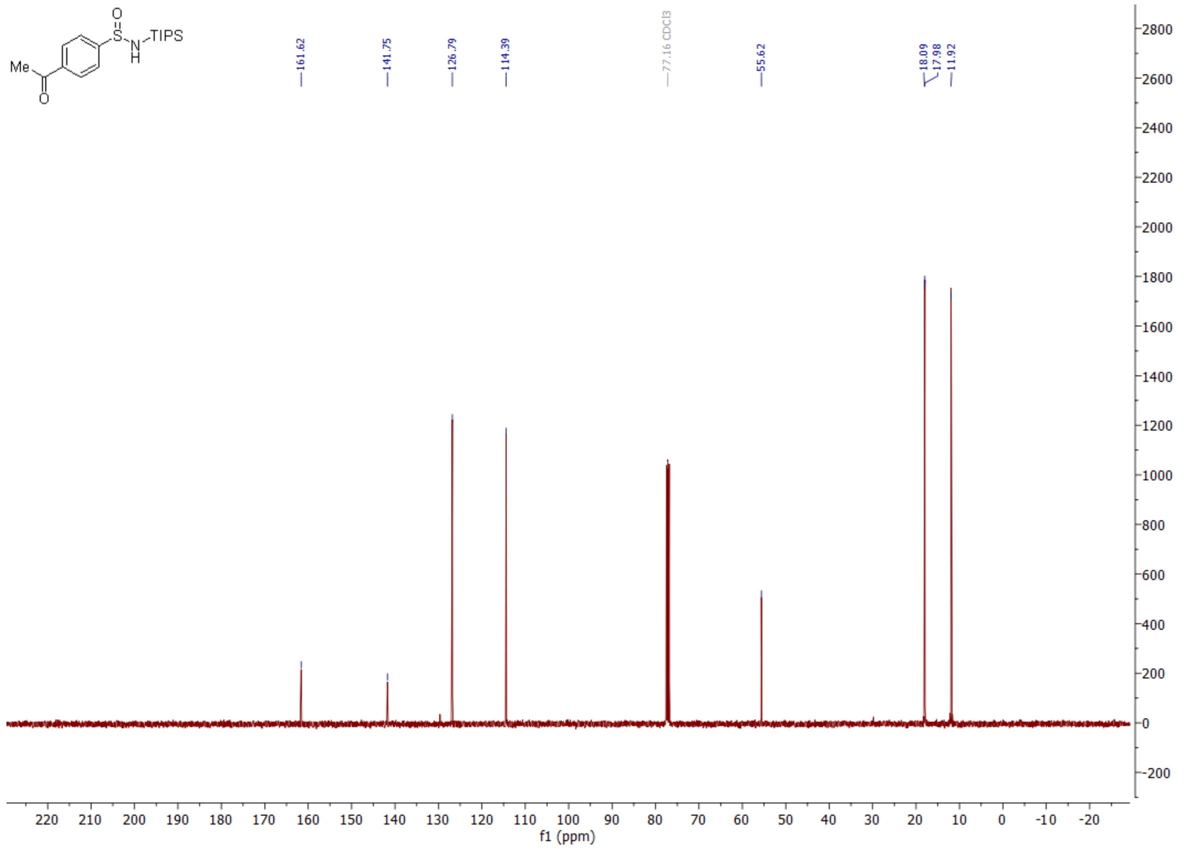

Methyl 3-(((triisopropylsilyl)amino)sulfinyl)benzoate (1f)

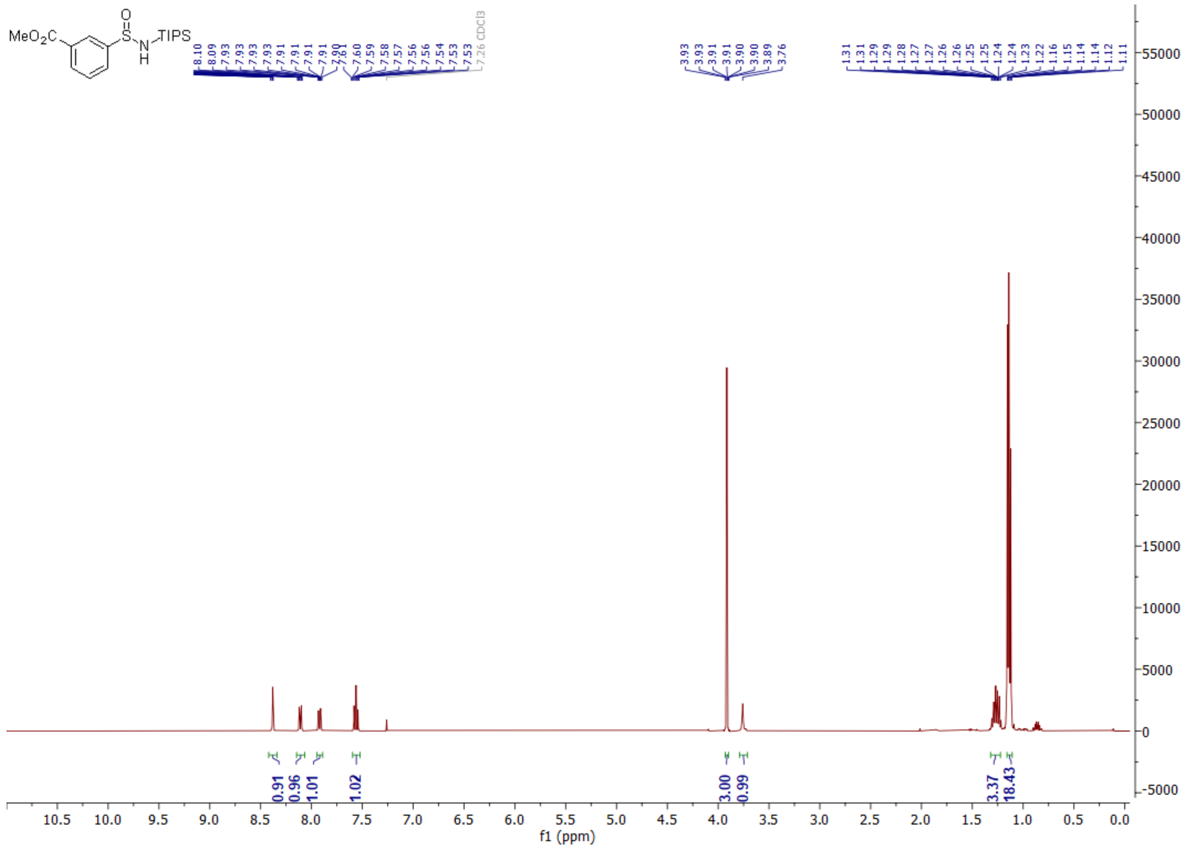

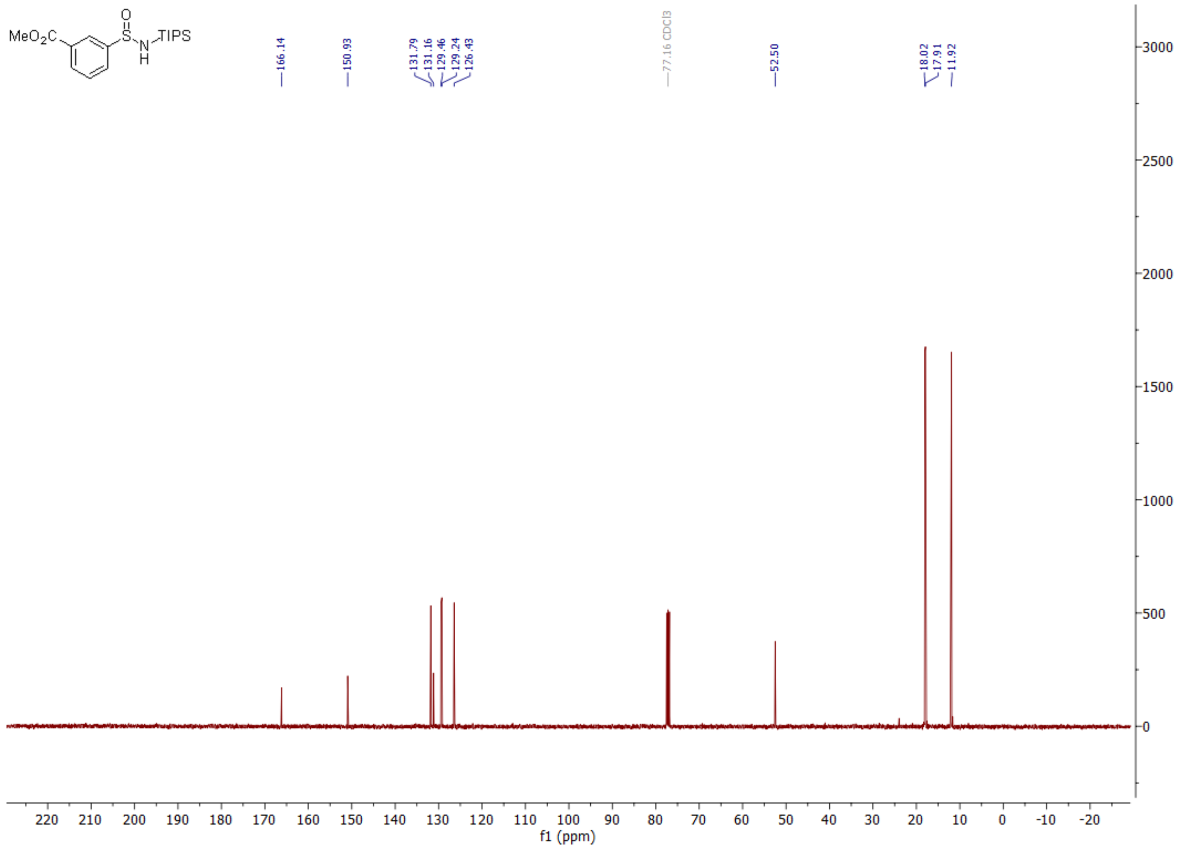

3-(Trifluoromethyl)-*N*-(triisopropylsilyl)benzenesulfinamide (1g)

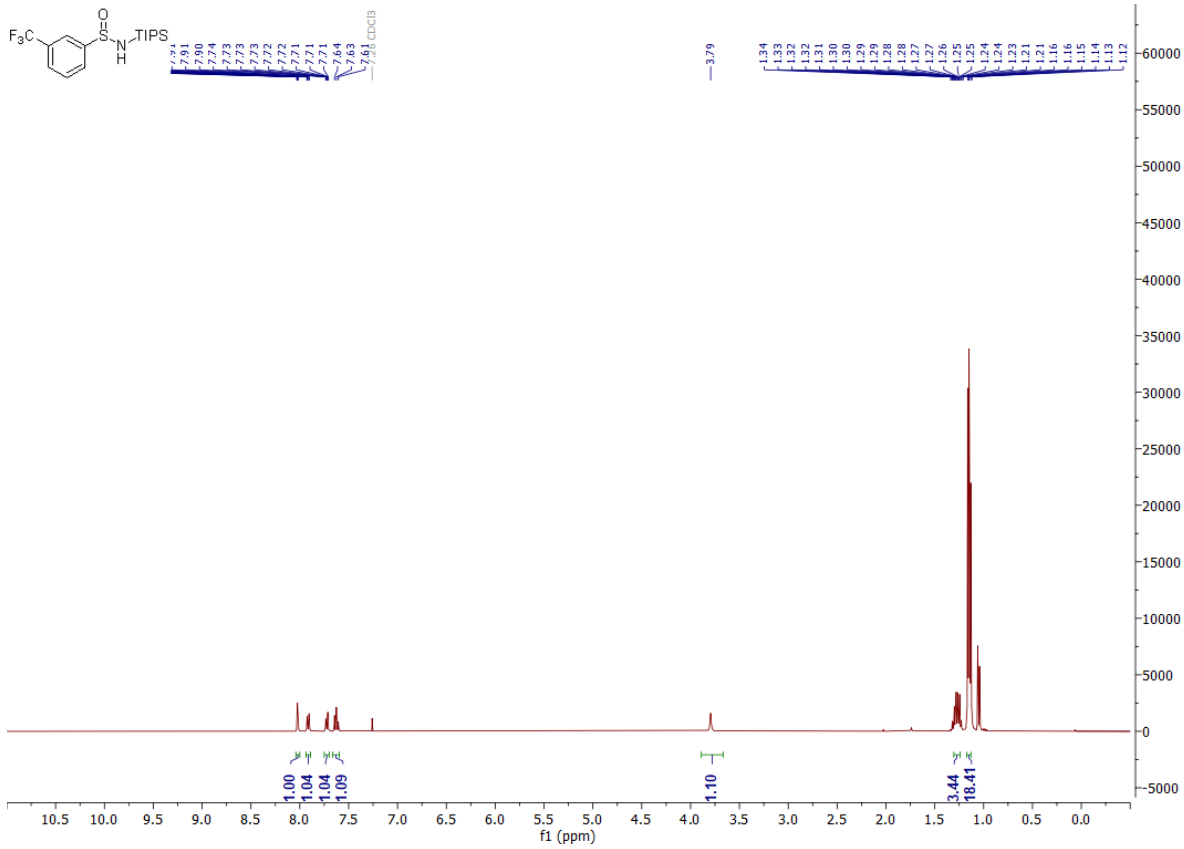

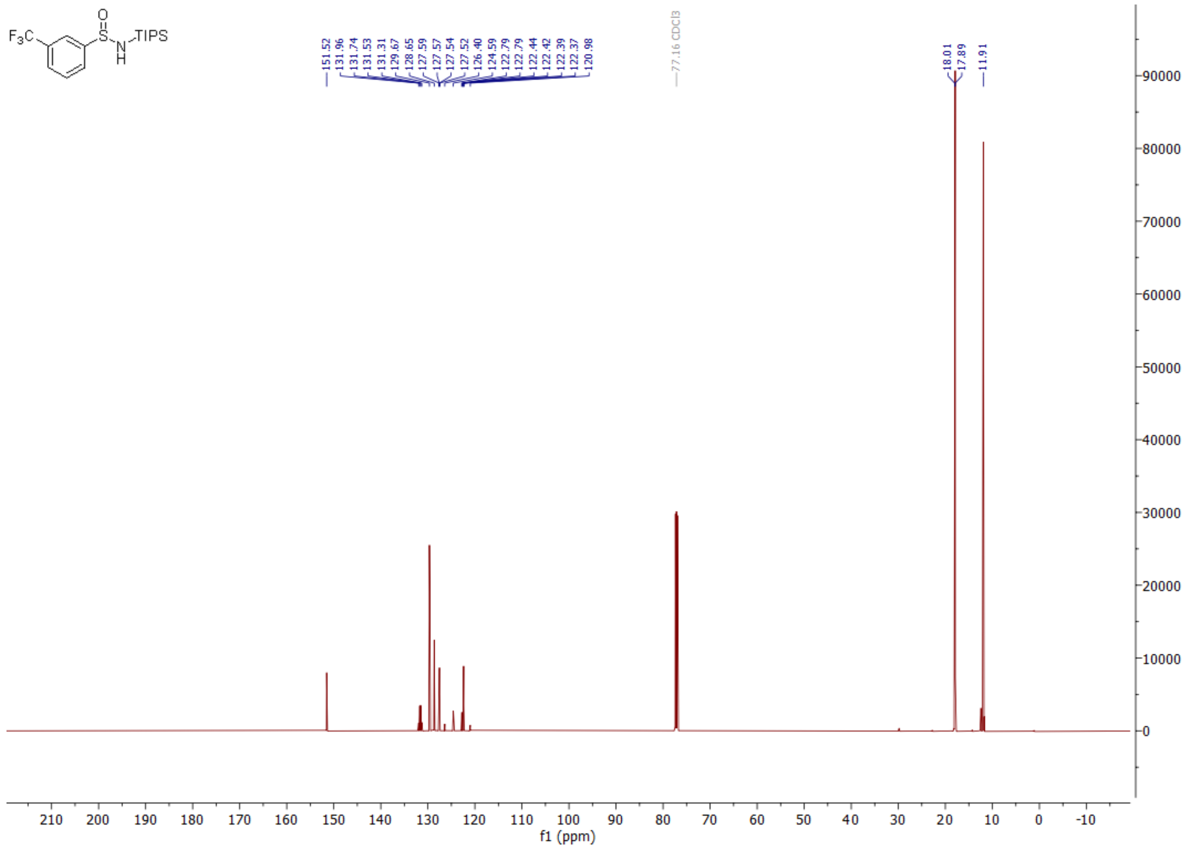
F3C
O
S
N
H
TIPS
151.52
131.96
131.74
131.53
131.31
129.67
128.65
127.59
127.57
127.54
127.52
126.40
124.59
122.79
122.79
122.44
122.42
122.39
122.37
120.98
77.16 CDCl3
18.01
17.89
11.91
f1 (ppm)

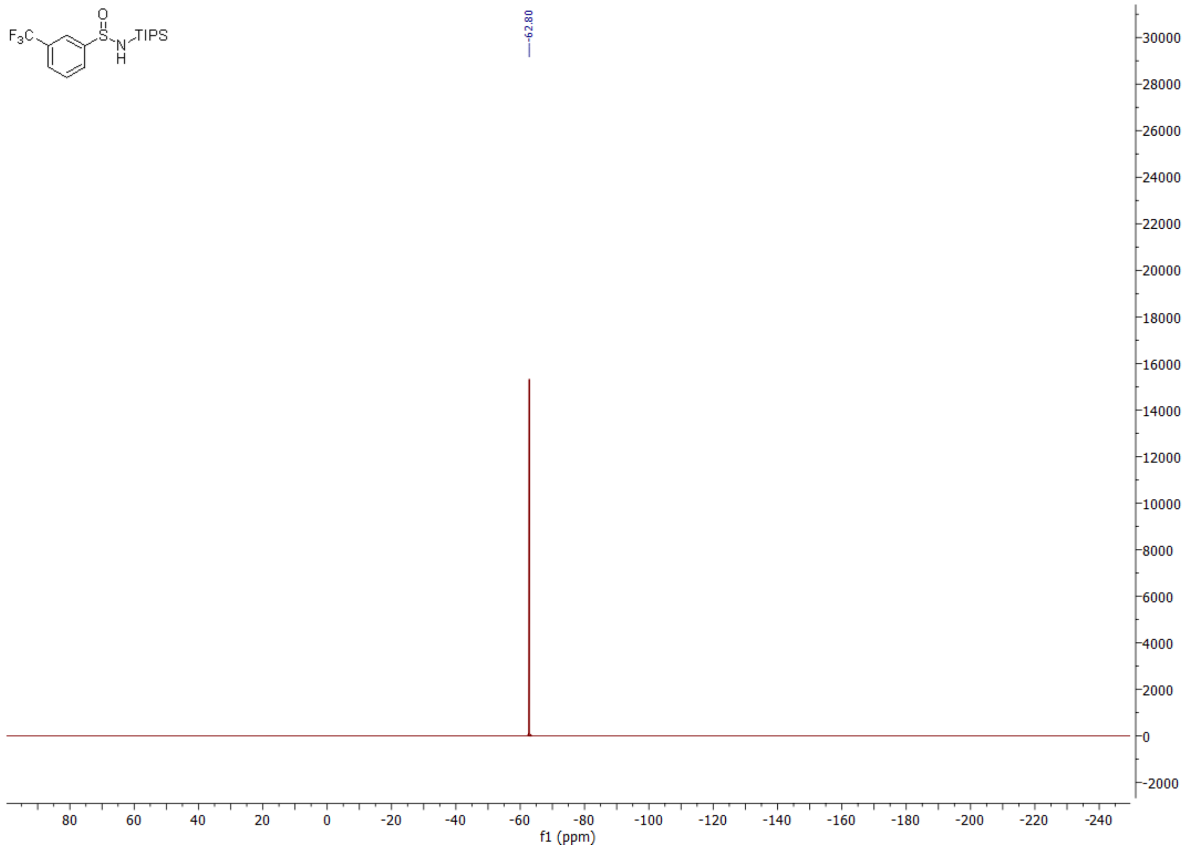
F3C
O
S
N
H
TIPS
-62.80
f1 (ppm)

N-(Triisopropylsilyl)naphthalene-2-sulfinamide (1h)

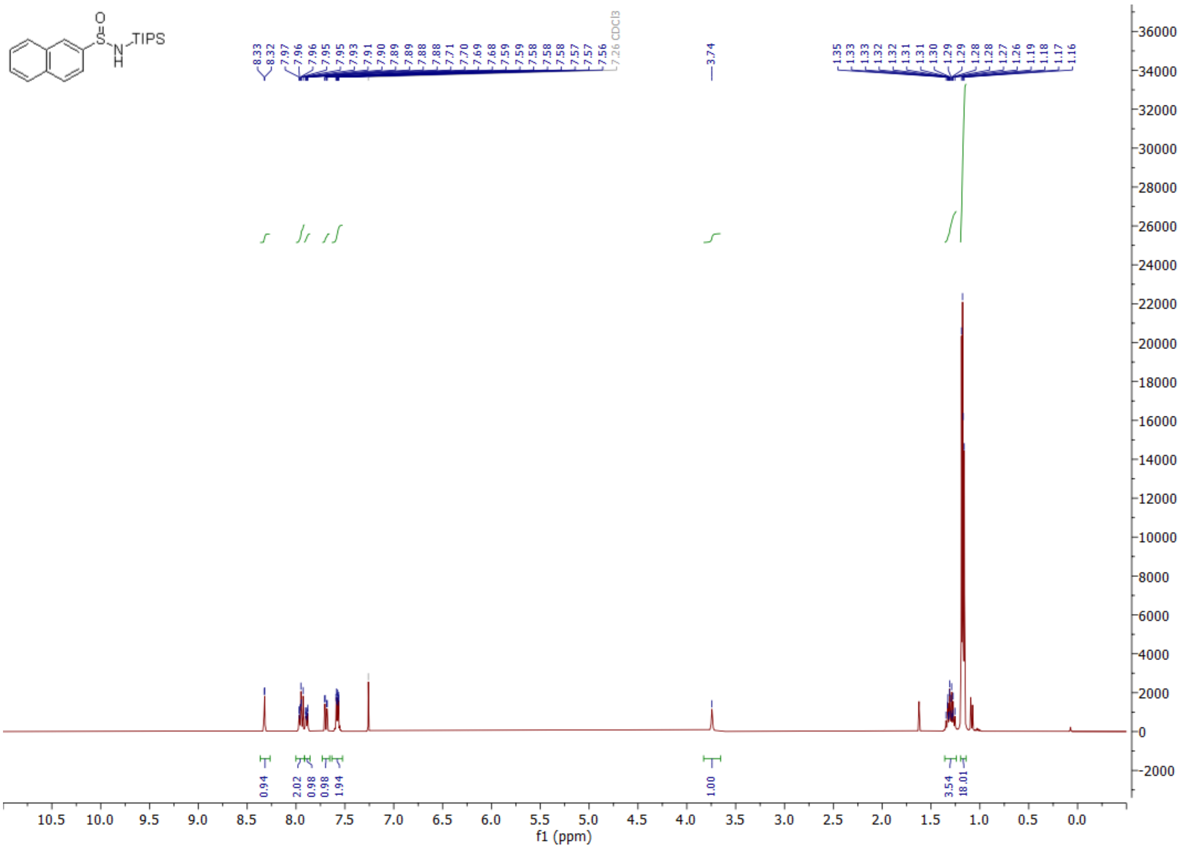

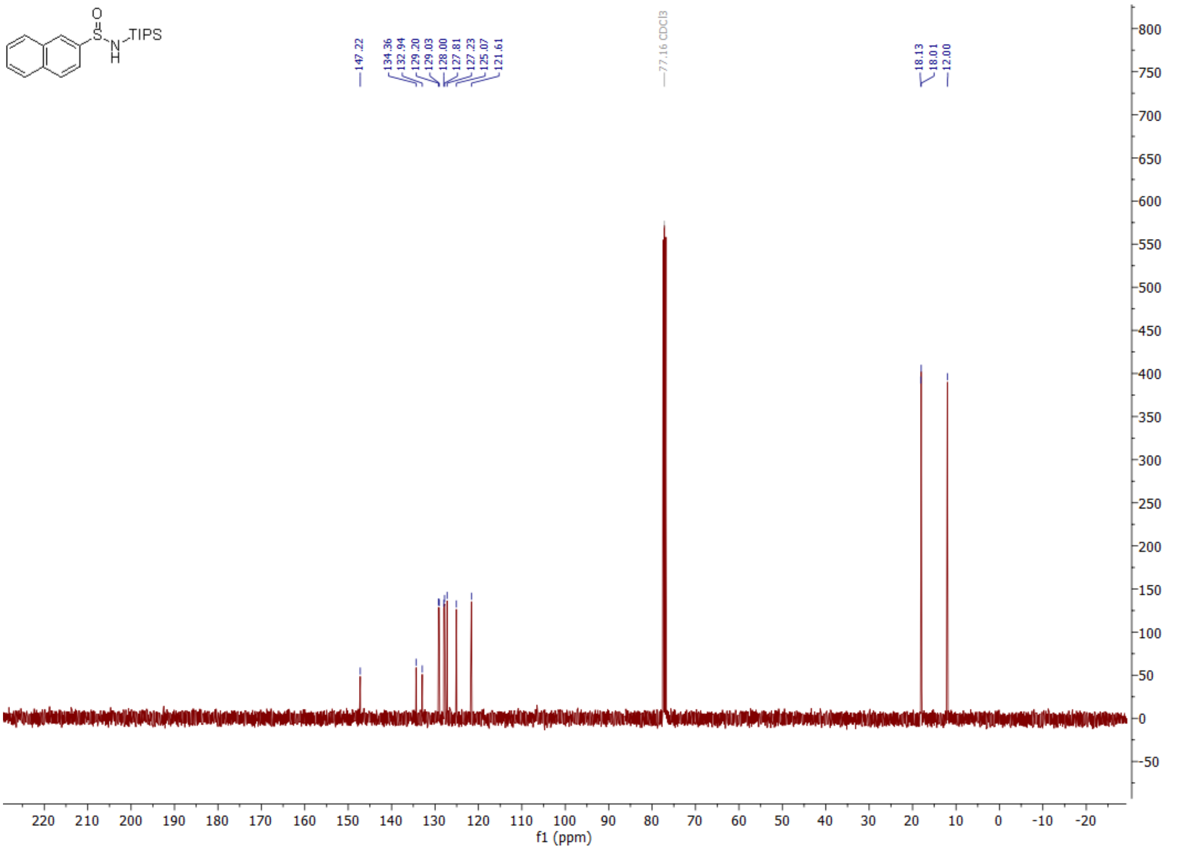

Tert-butyl (2-cyano-4-(((triisopropylsilyl)amino)sulfinyl)phenyl)carbamate (1i)

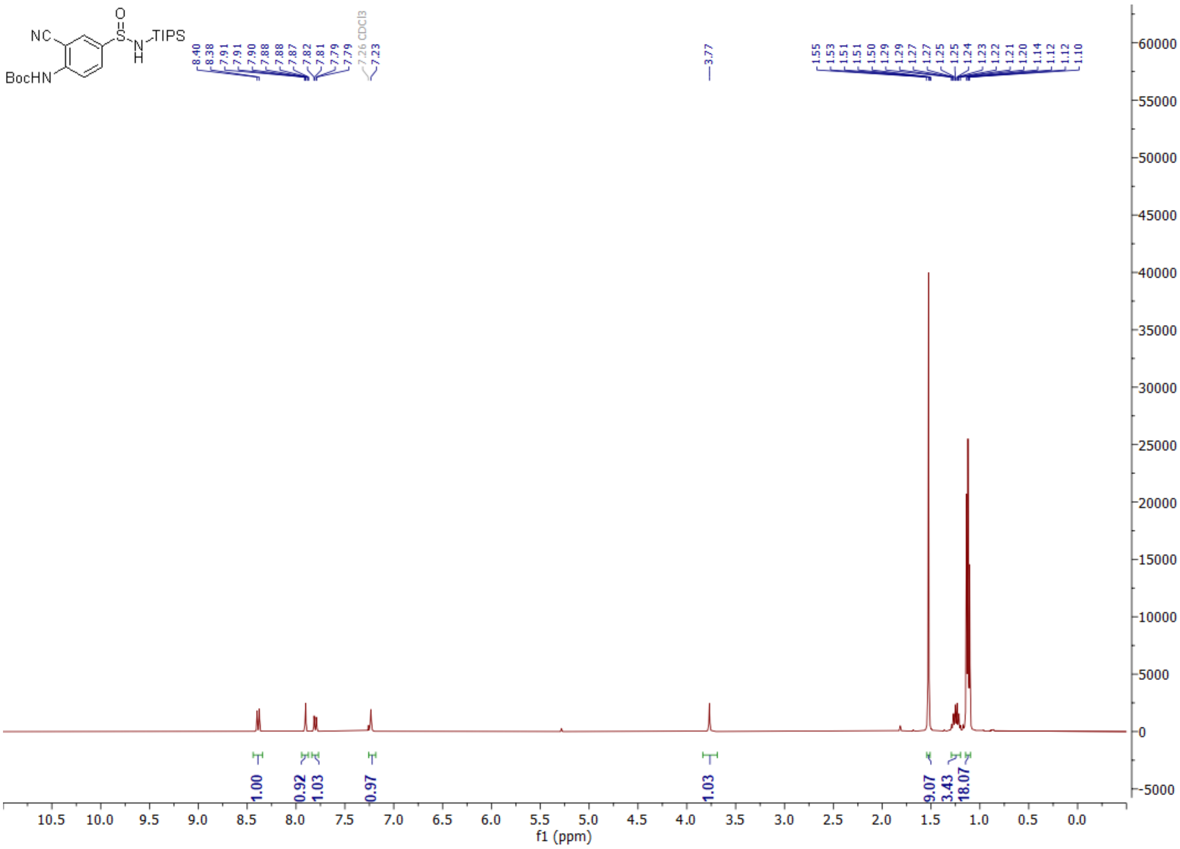

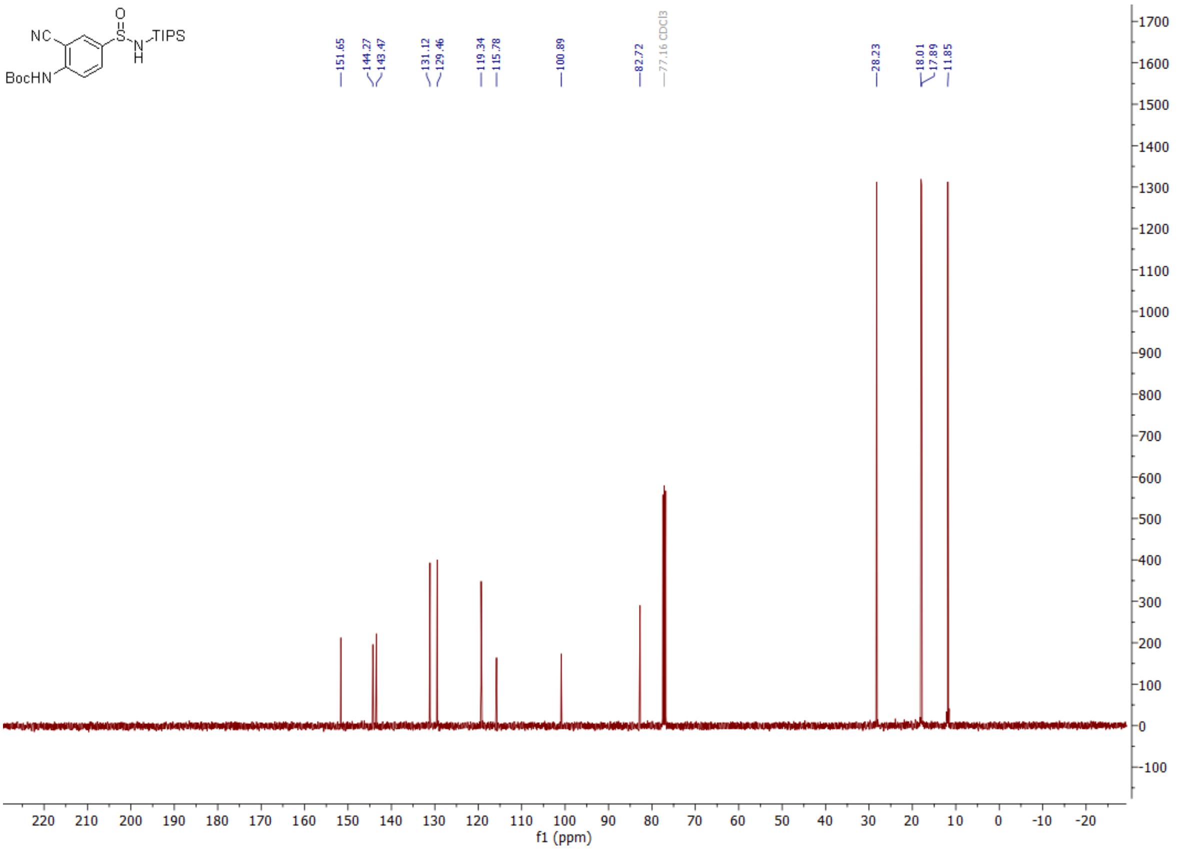

4-Methoxy-*N*-(triisopropylsilyl)benzenesulfinamide (1j)

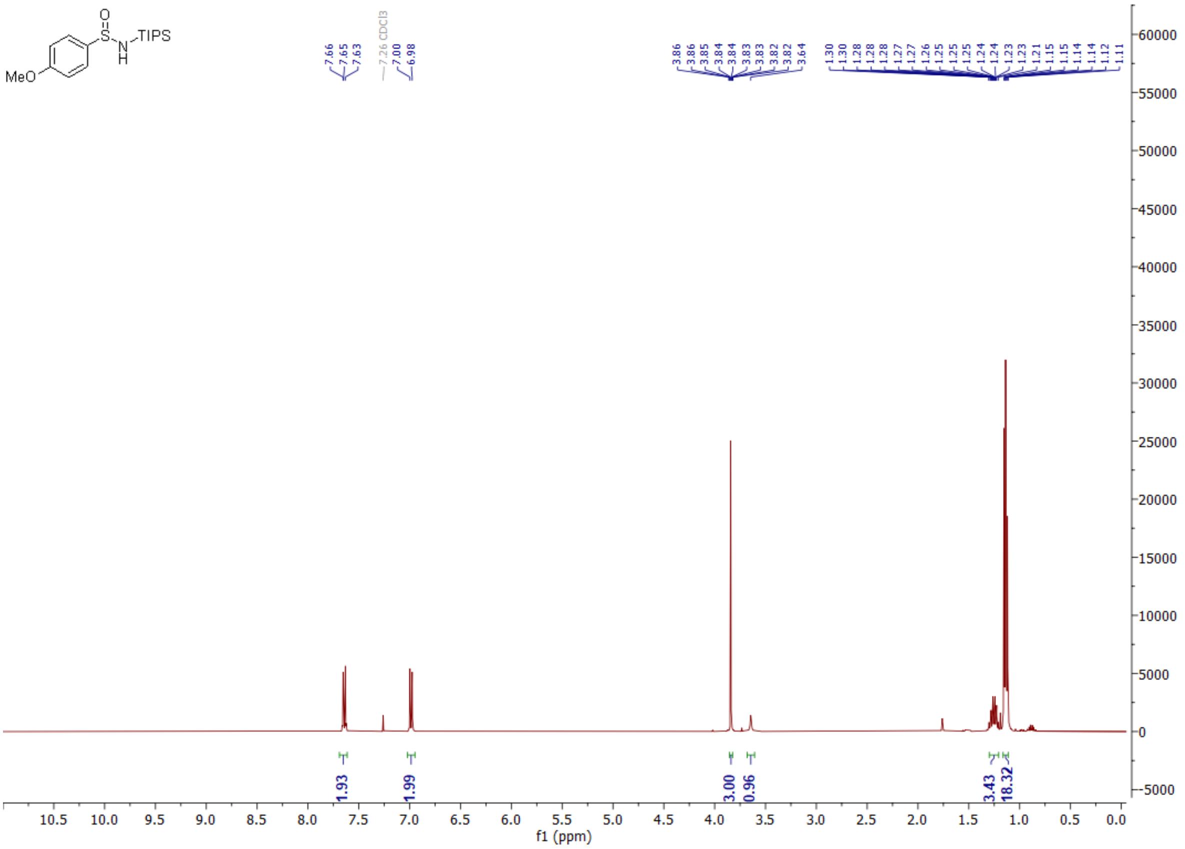

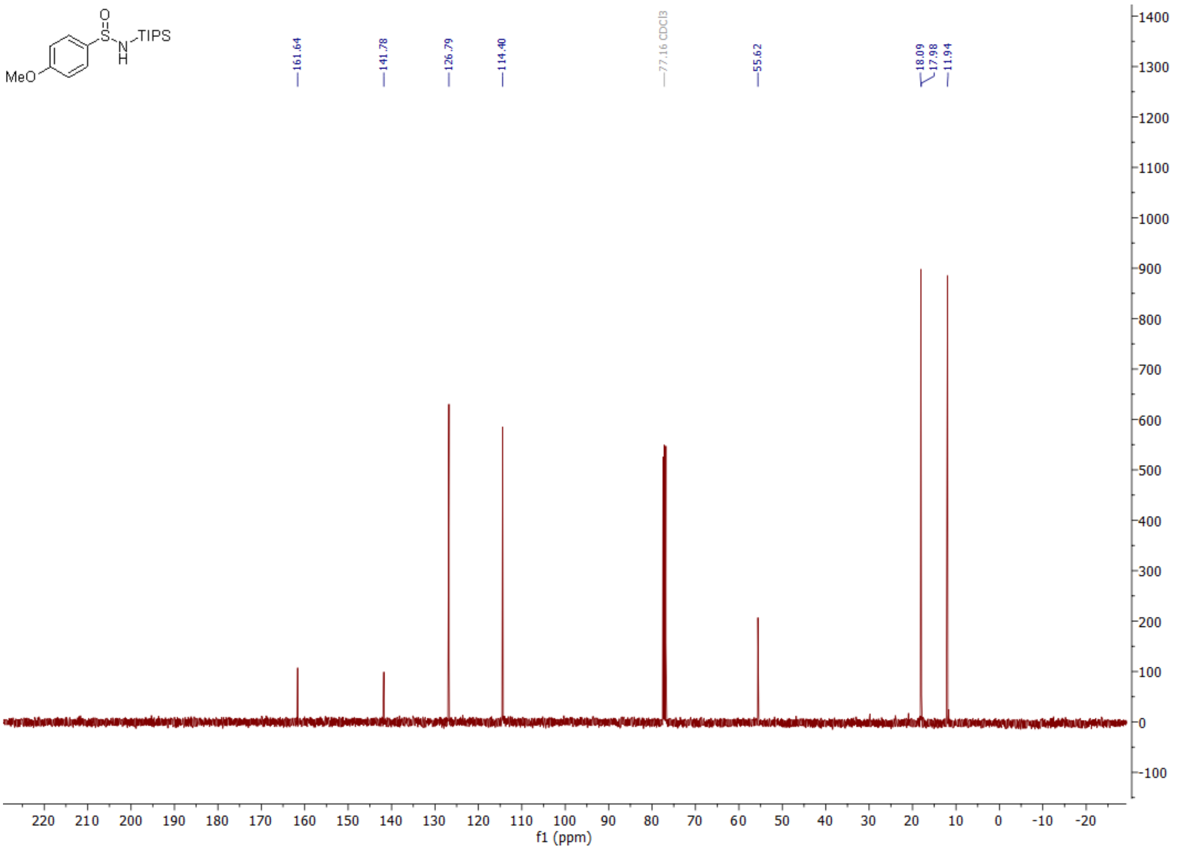

4-Methyl-*N*-(triisopropylsilyl)benzenesulfinamide (1k)

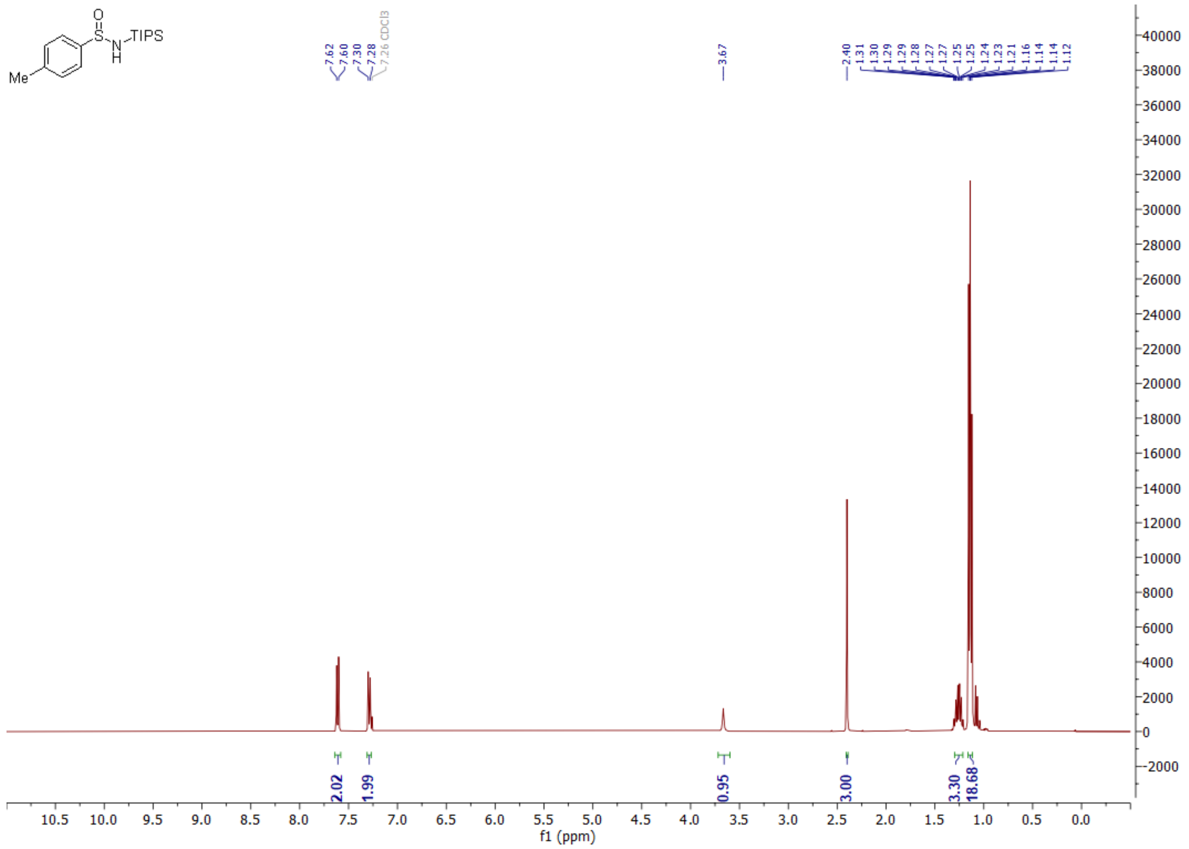

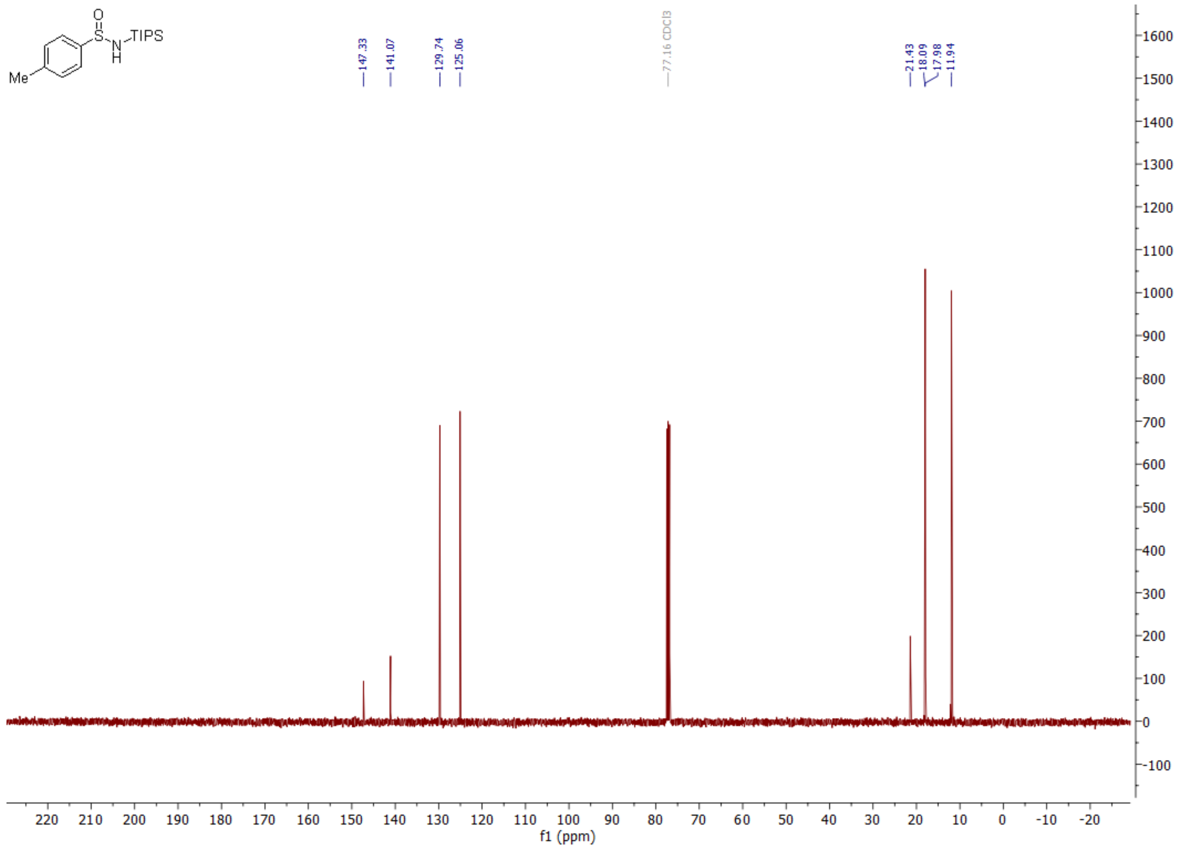

4-(2,5-Dioxopyrrolidin-1-yl)-*N*-(triisopropylsilyl)benzenesulfinamide (1l)

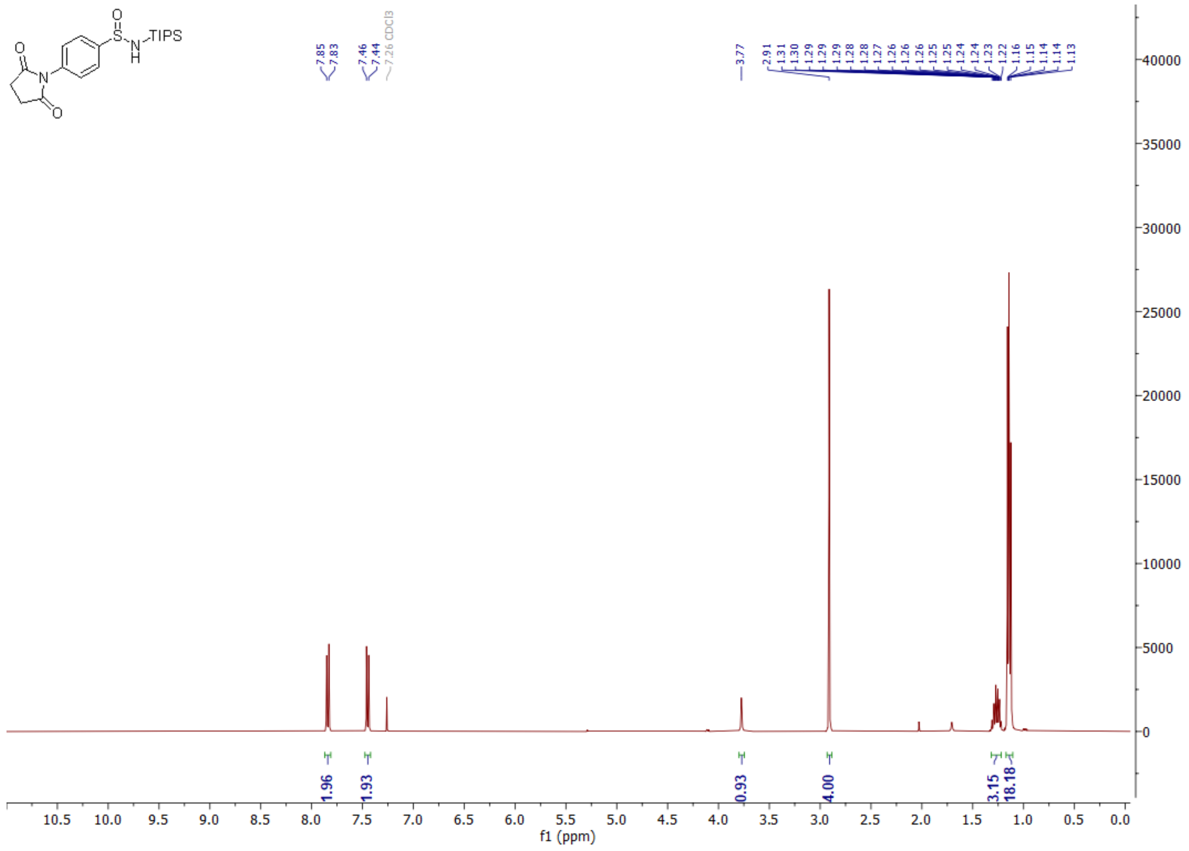

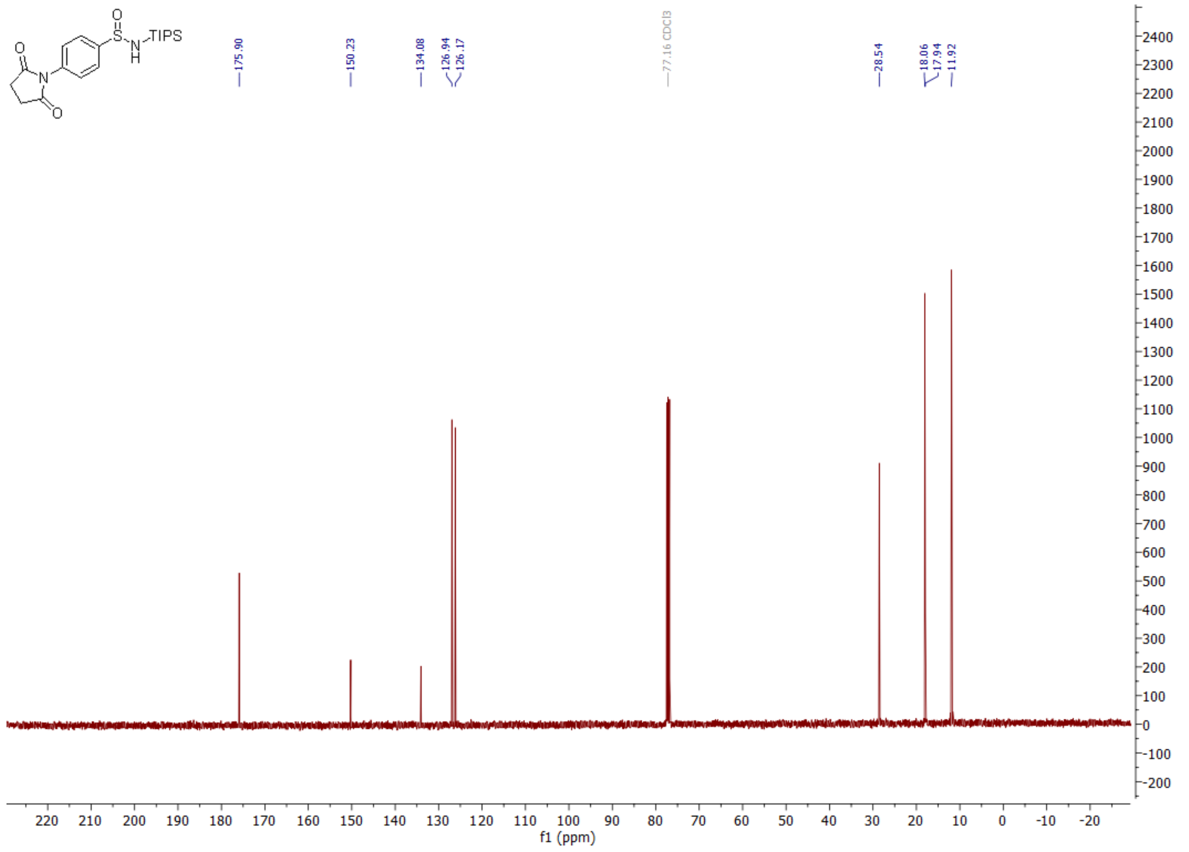

2-Methyl-*N*-(triisopropylsilyl)benzenesulfinamide (1m)

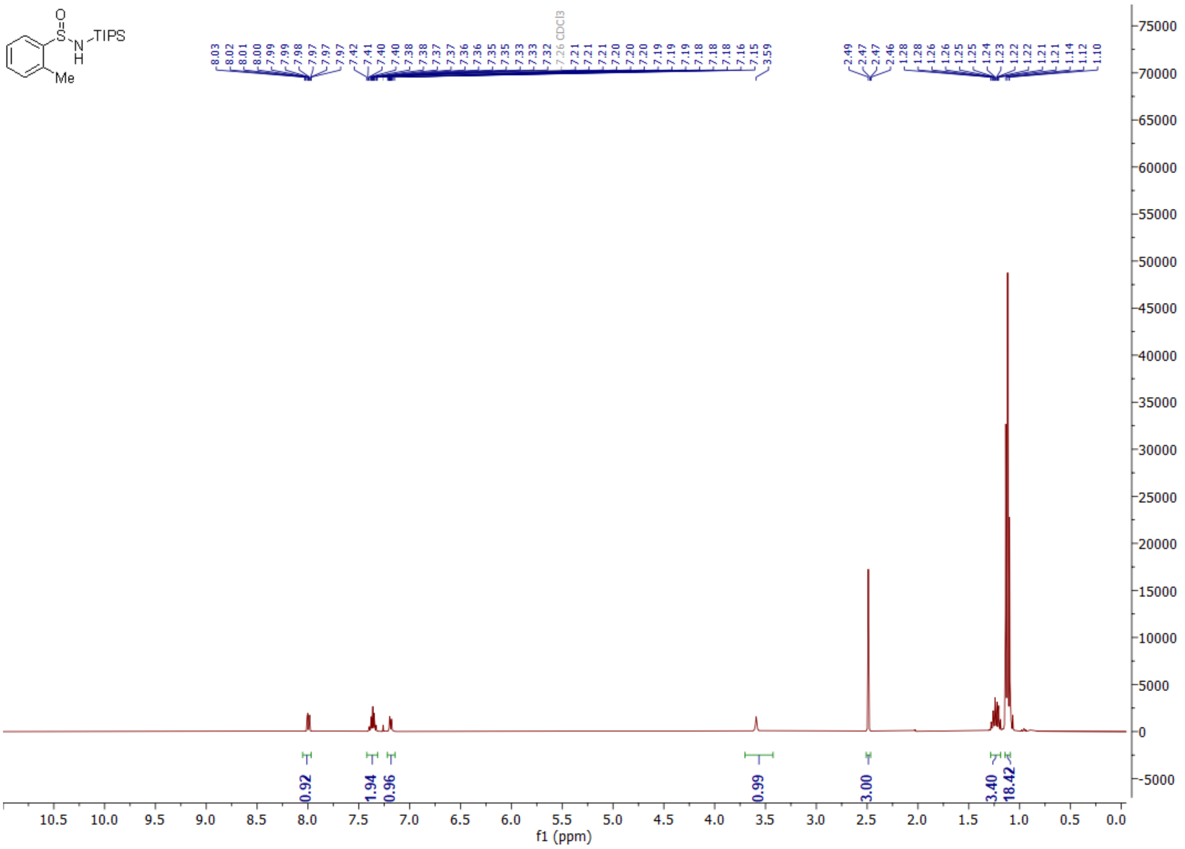

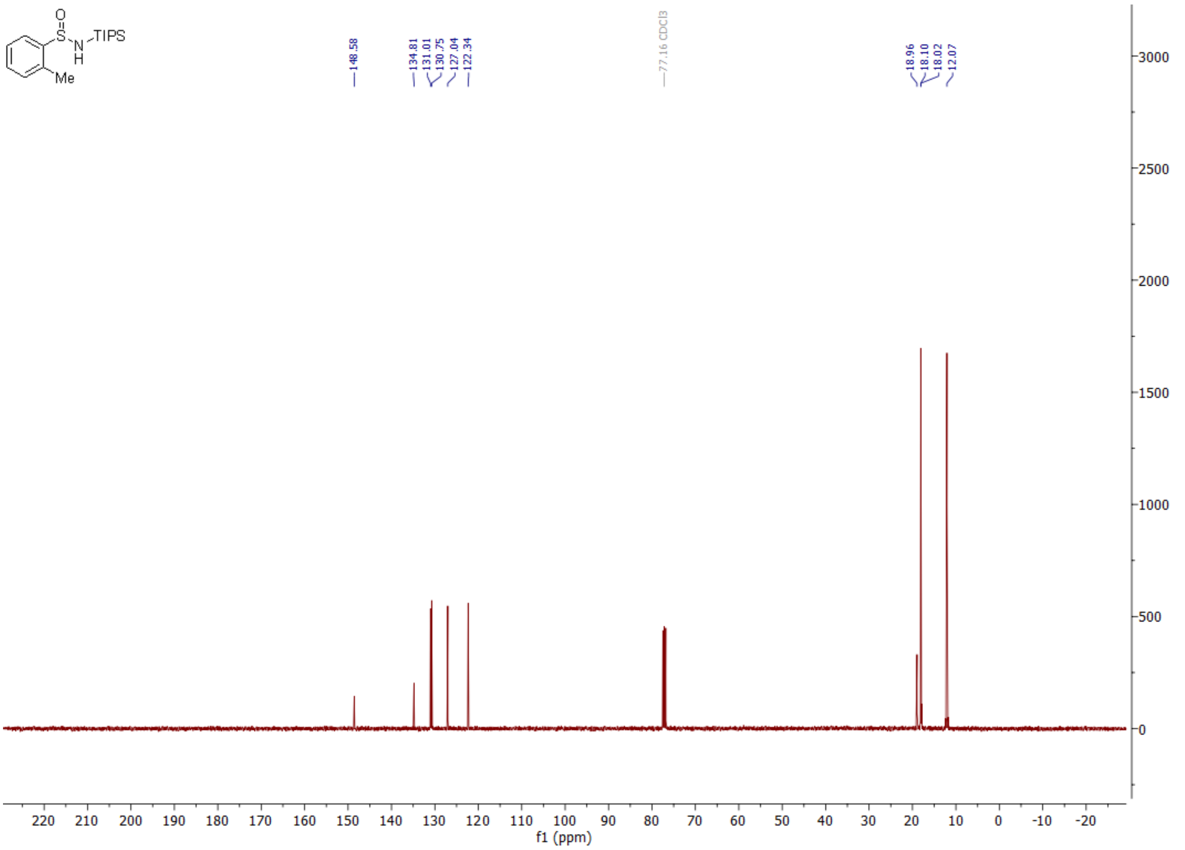

3-Methoxy-*N*-(triisopropylsilyl)benzenesulfinamide (1n)

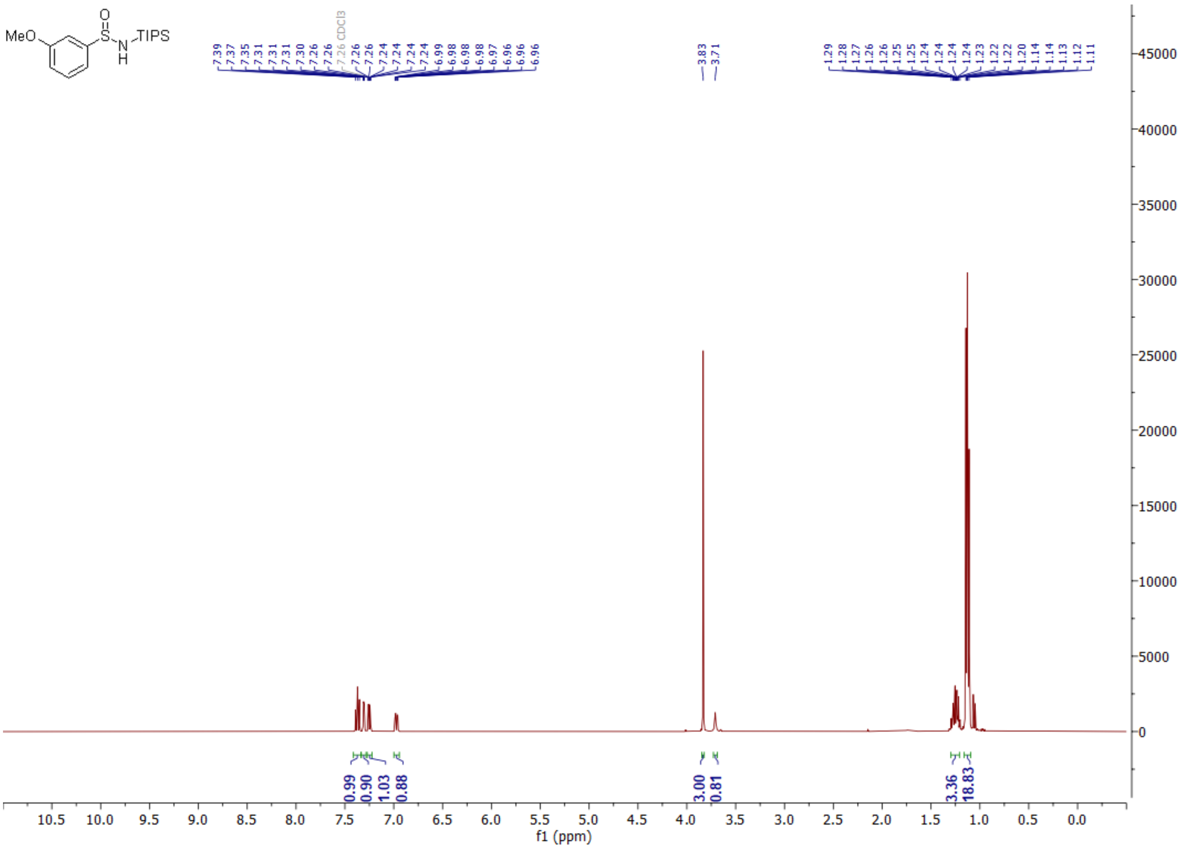

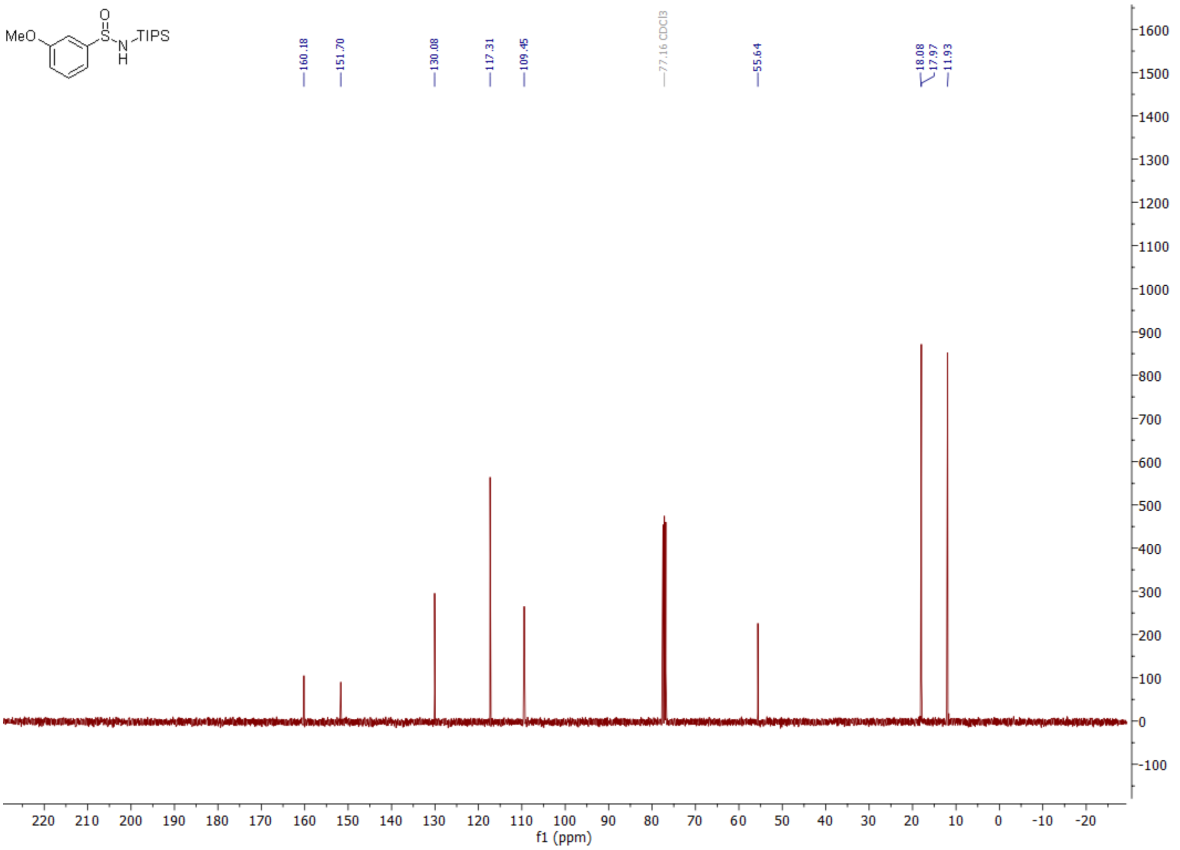

N-(Triisopropylsilyl)-2-vinylbenzenesulfinamide (1o)

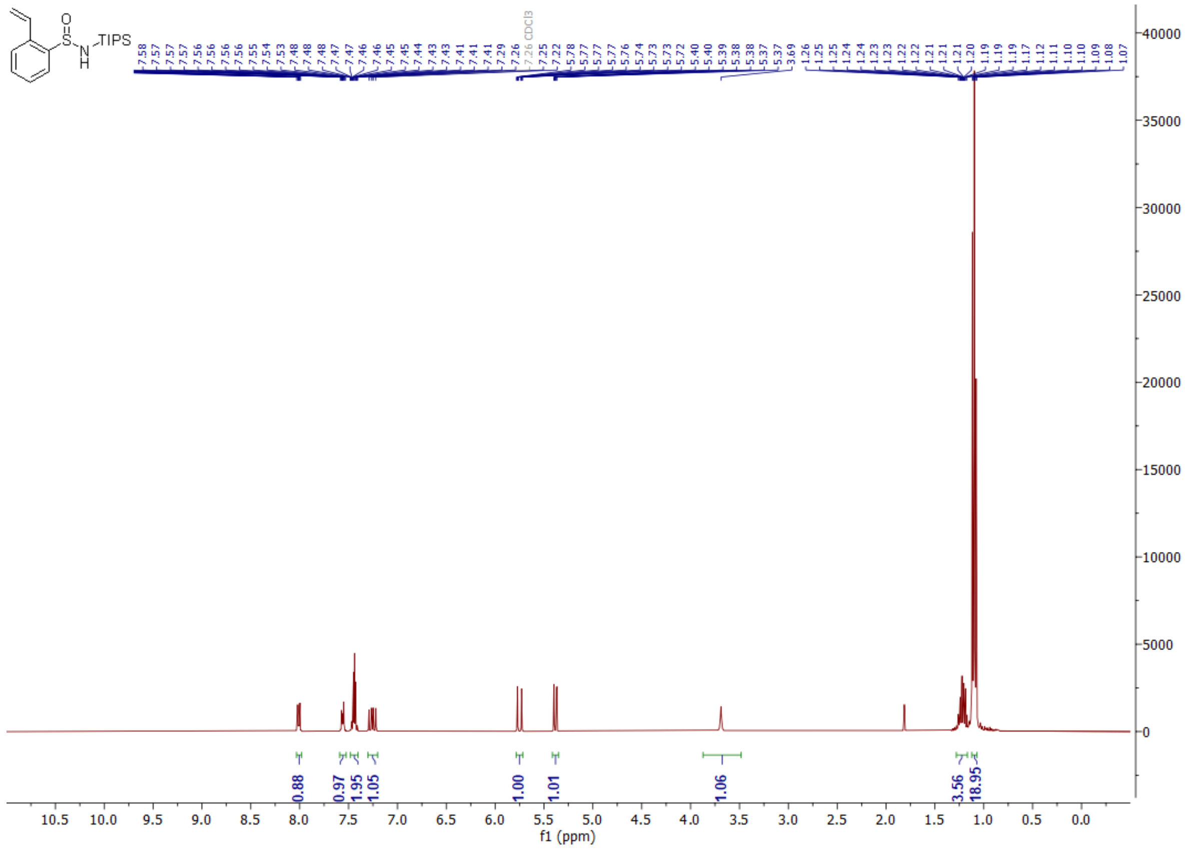

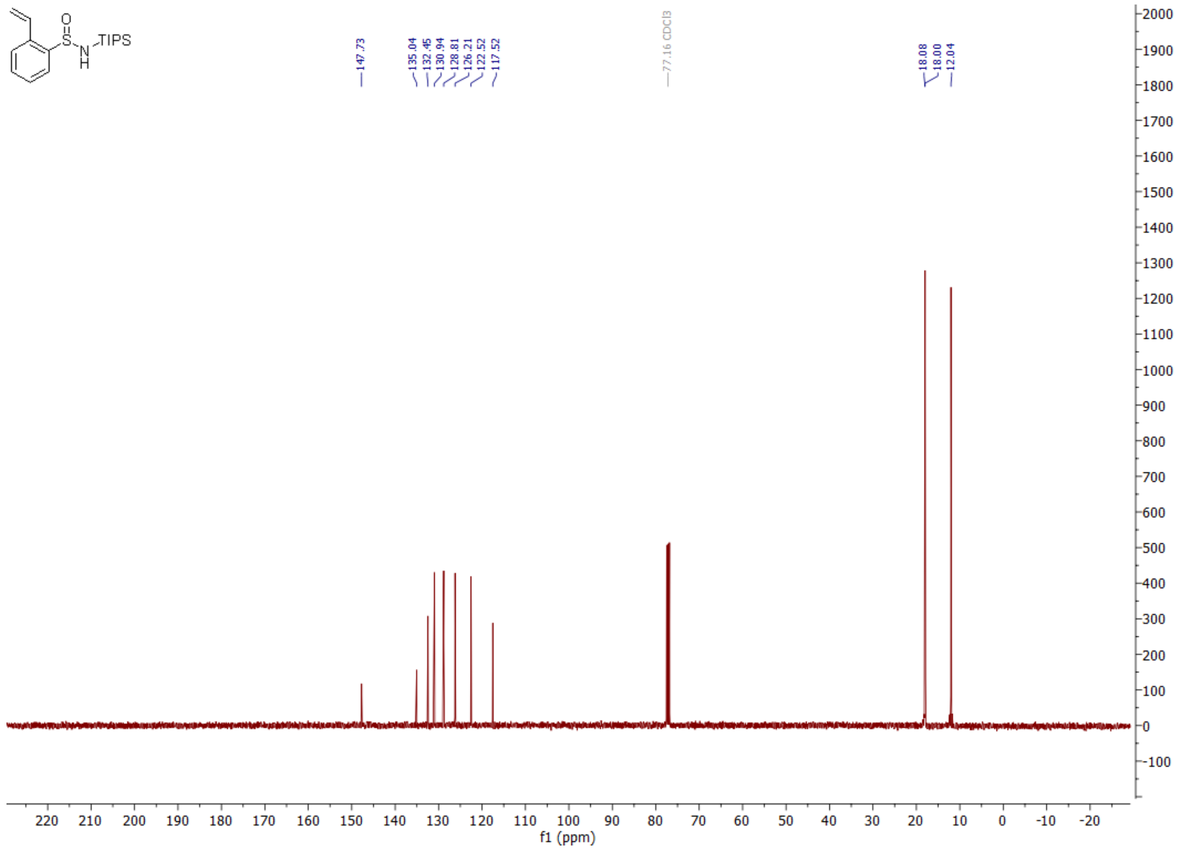

N-(Triisopropylsilyl)benzo[d][1,3]dioxole-5-sulfinamide (1p)

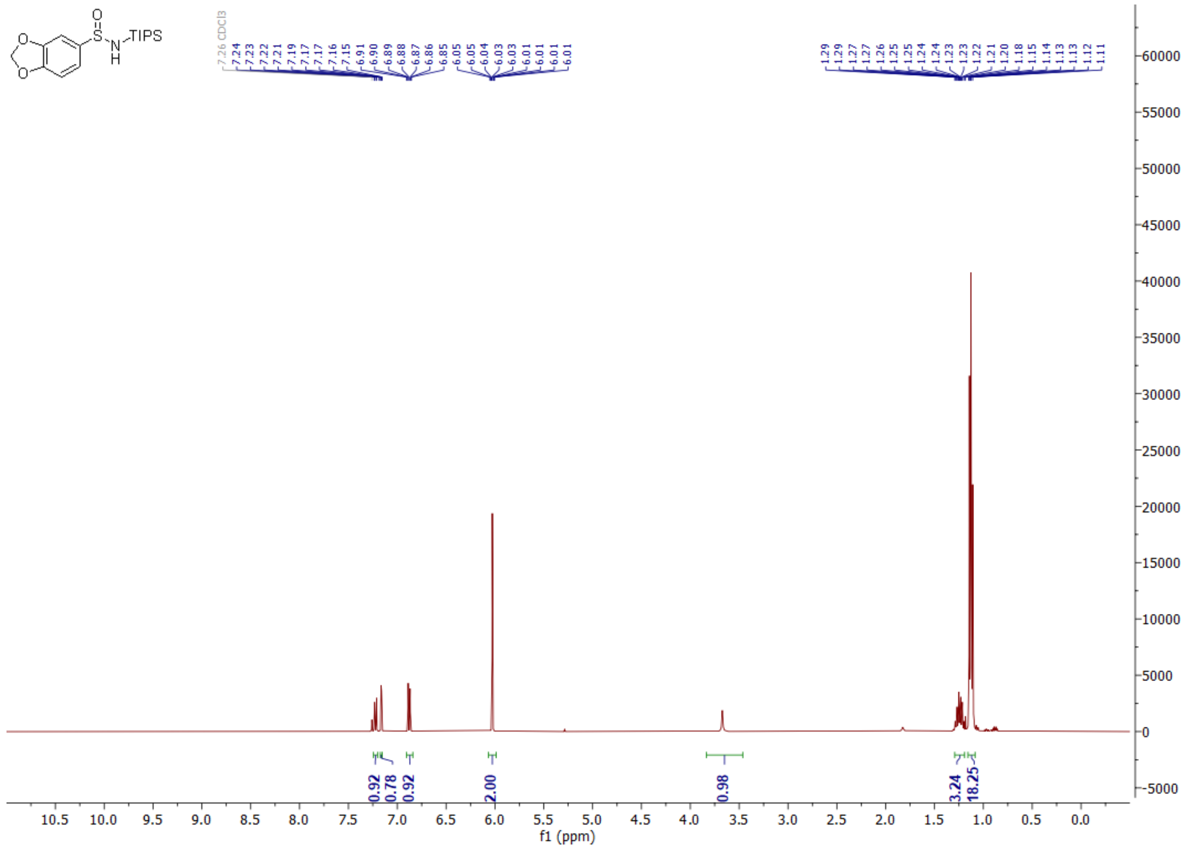

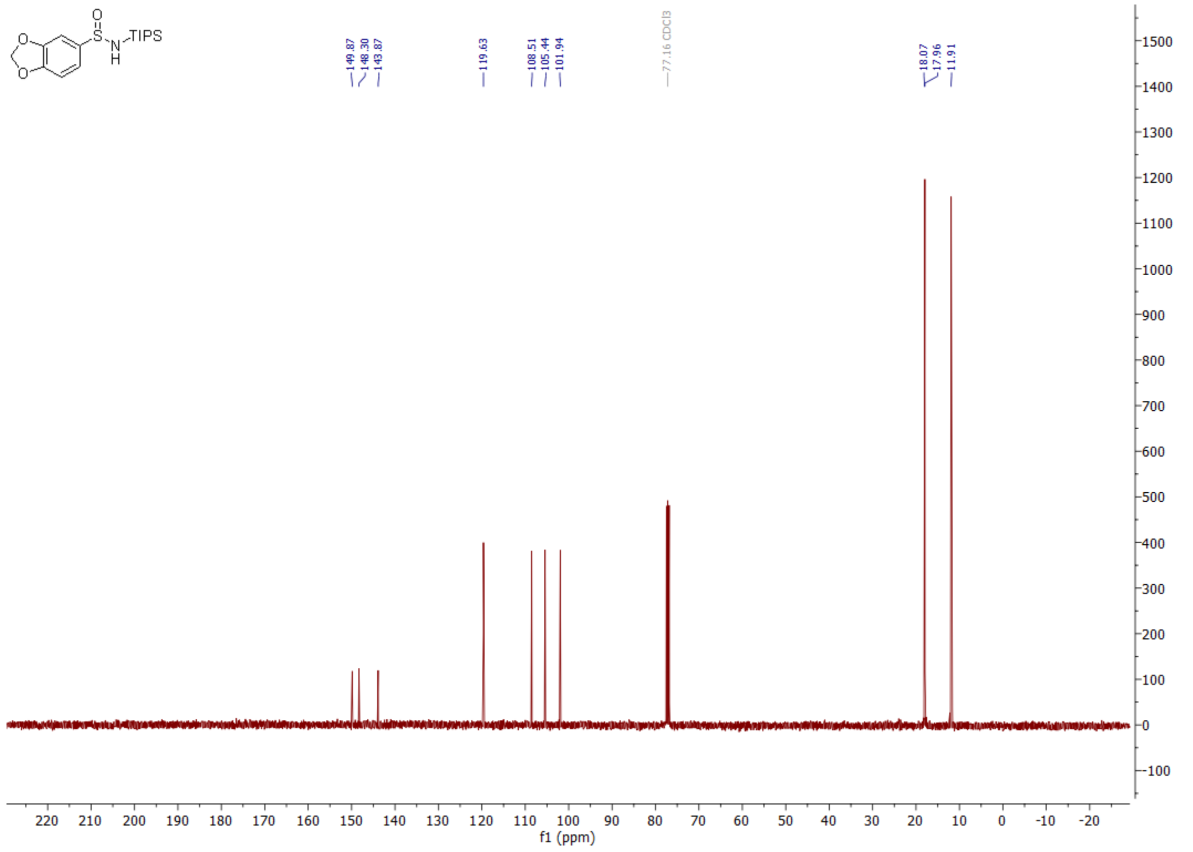

3-Methyl-1-oxo-*N*-(triisopropylsilyl)-2,3-dihydro-1H-indene-4-sulfinamide-major diastereoisomer (1qa)

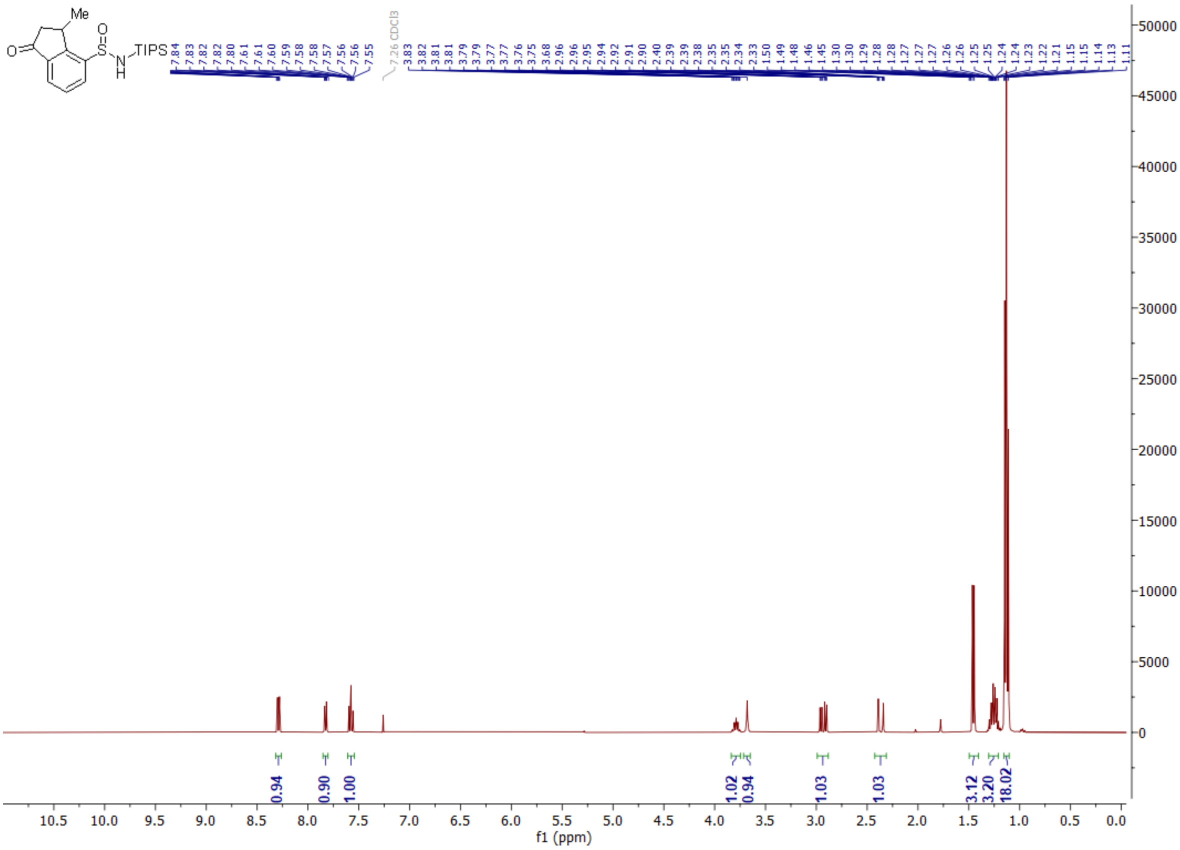

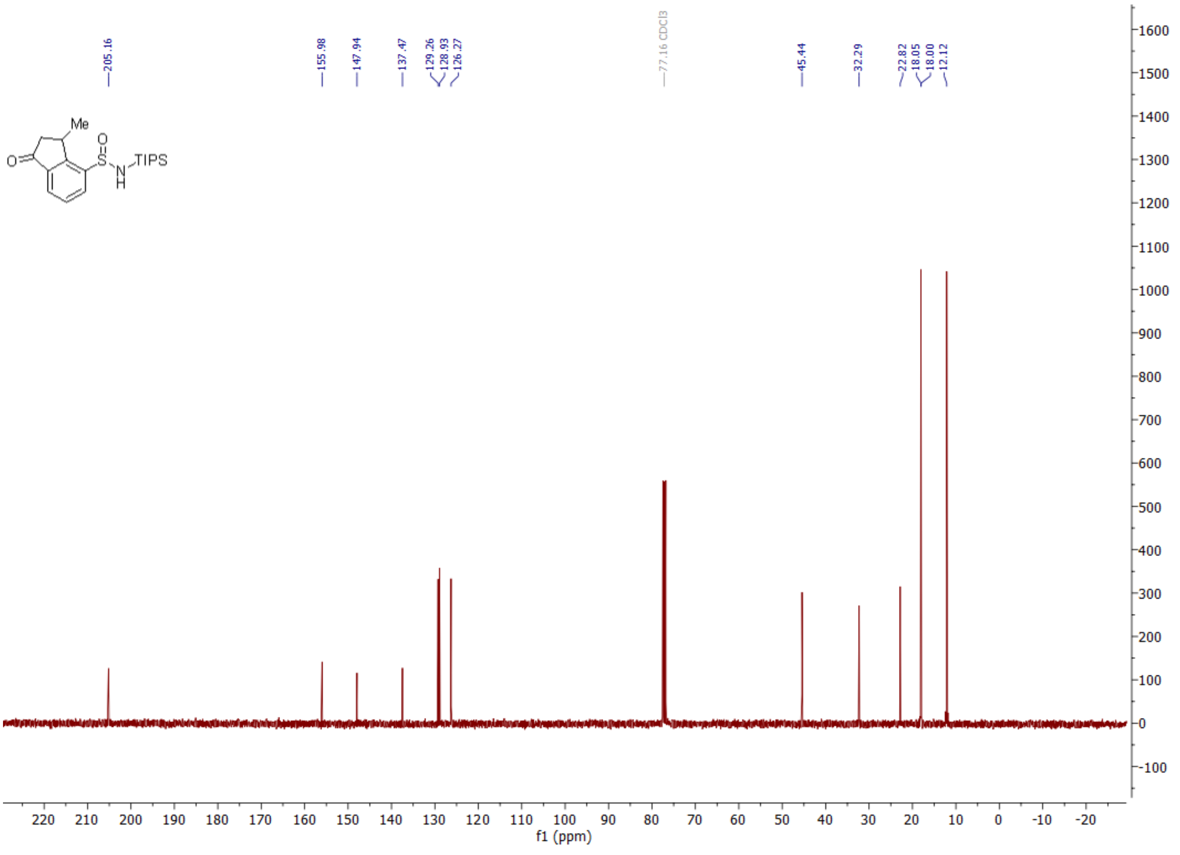

3-Methyl-1-oxo-*N*-(triisopropylsilyl)-2,3-dihydro-1H-indene-4-sulfinamide-minor diastereoisomer (1qb)

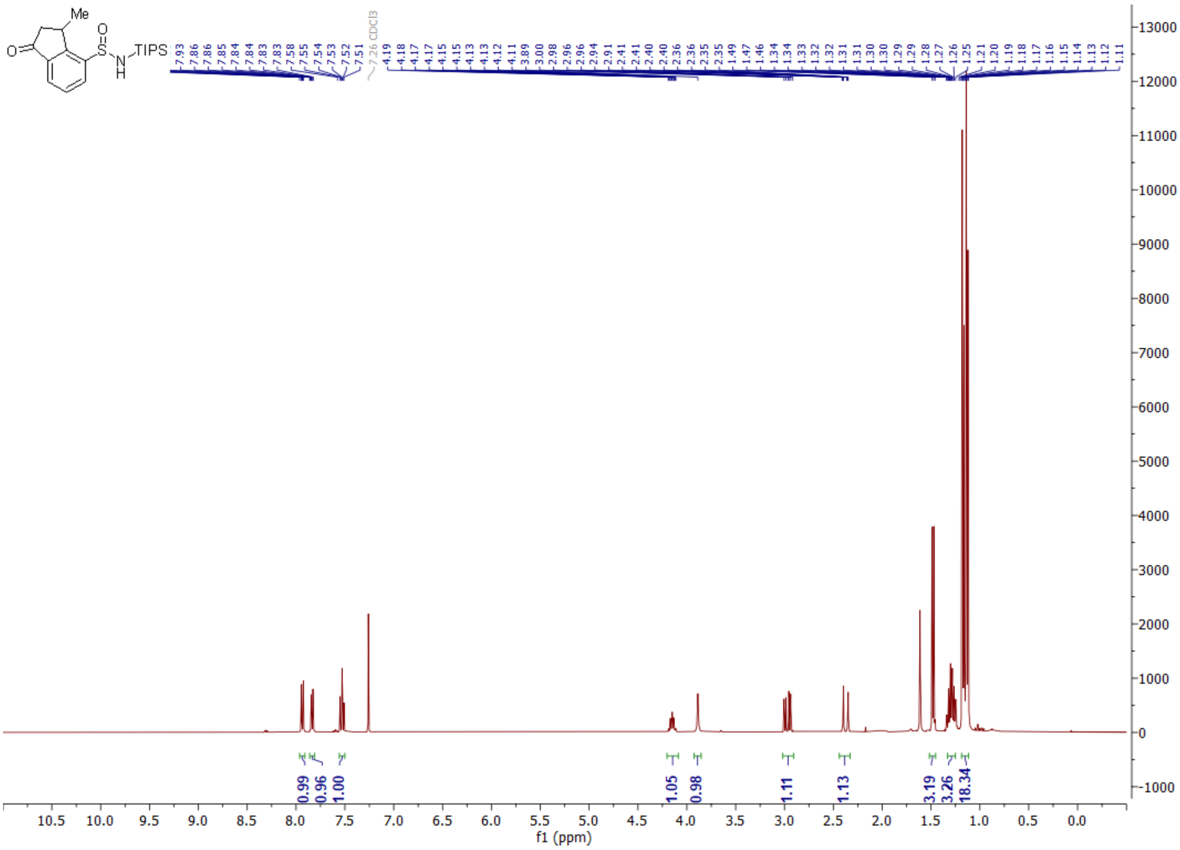

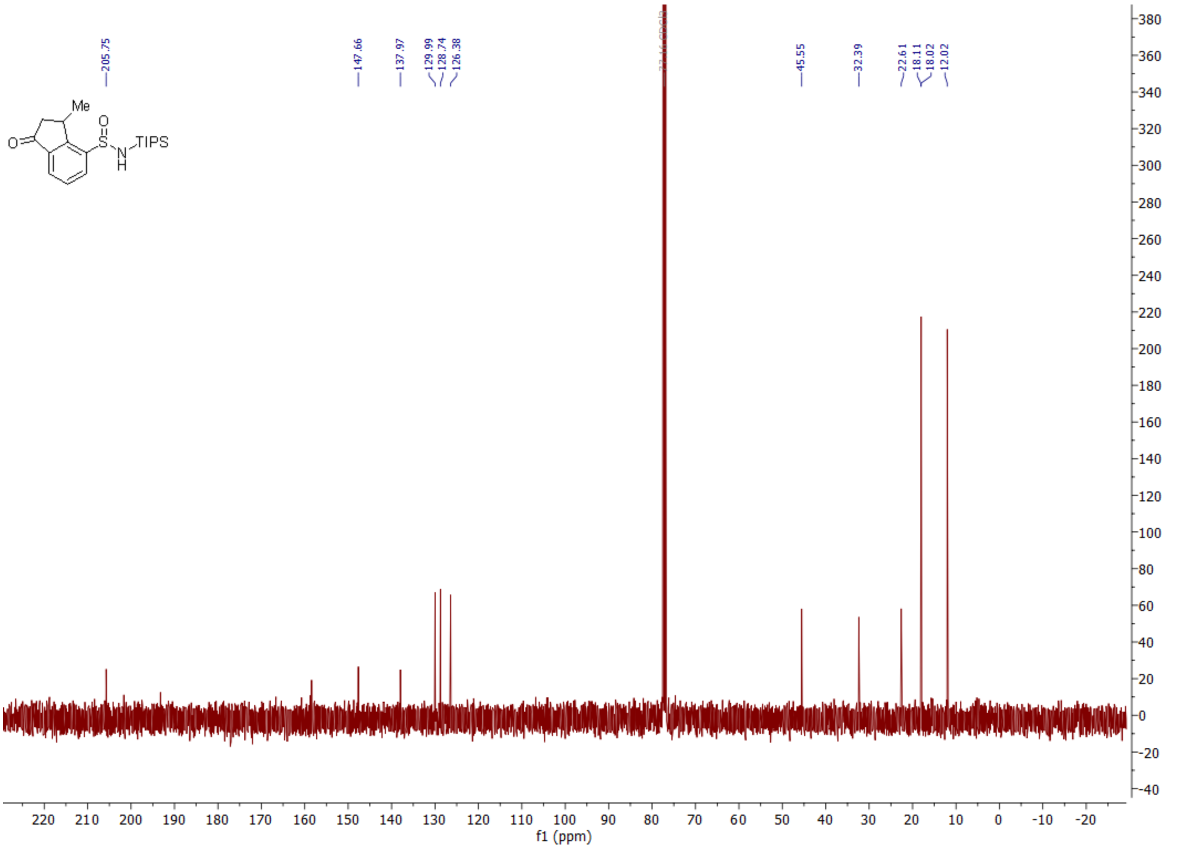

6-Methoxy-*N*-(triisopropylsilyl)pyridine-3-sulfinamide (1r)

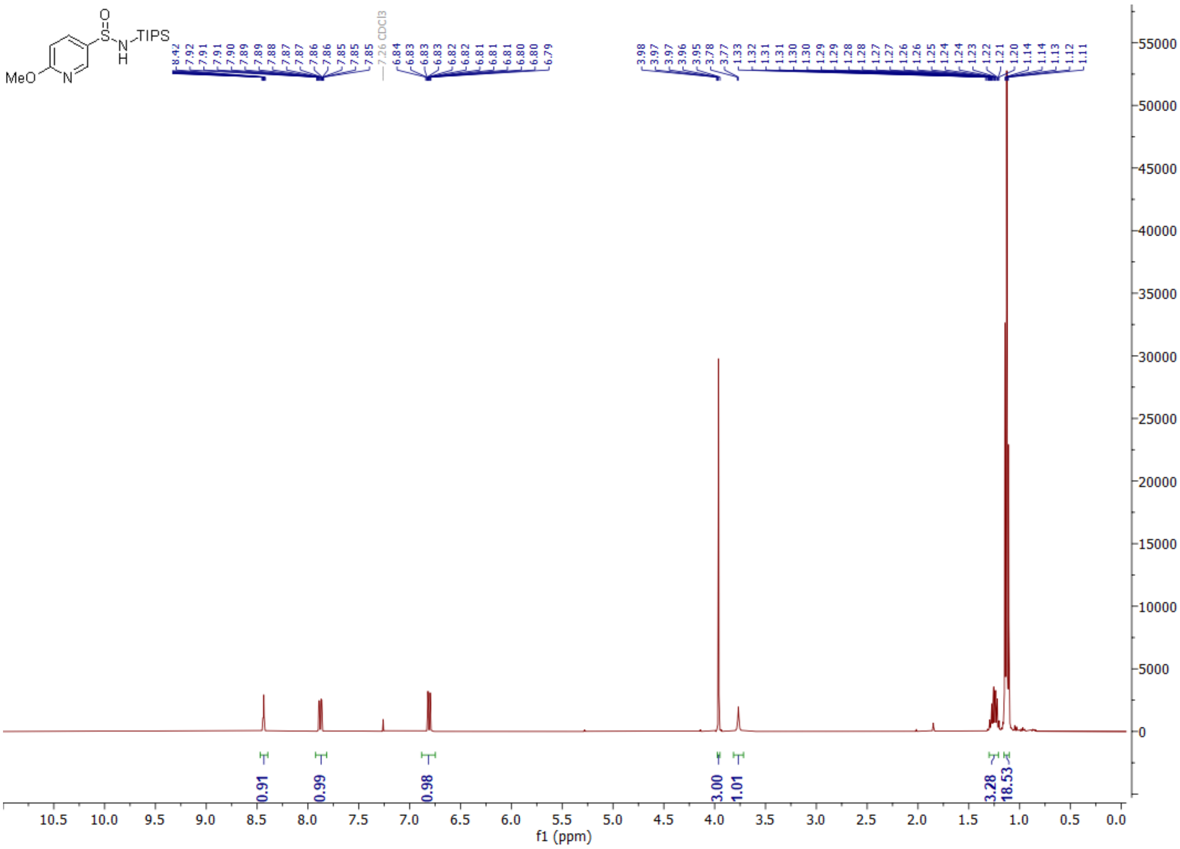

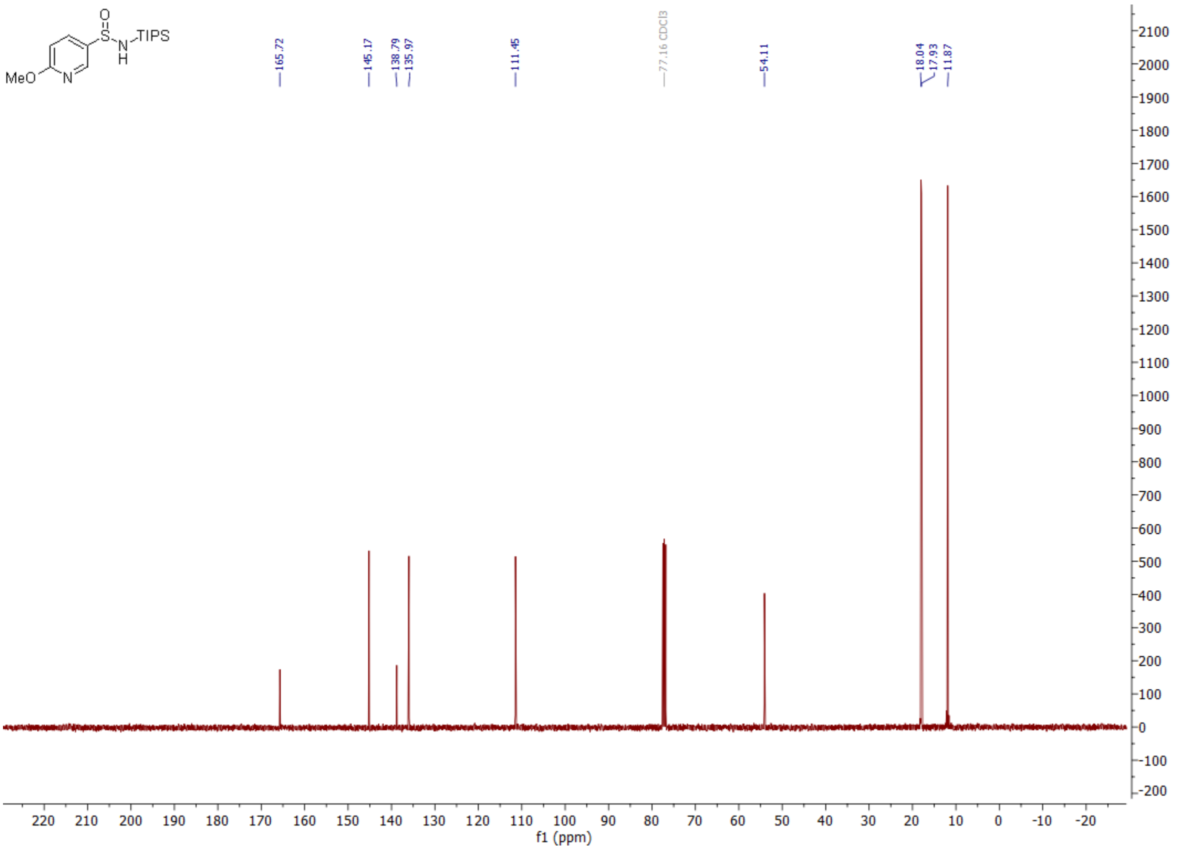

6-(Trifluoromethyl)-*N*-(triisopropylsilyl)pyridine-3-sulfinamide (1s)

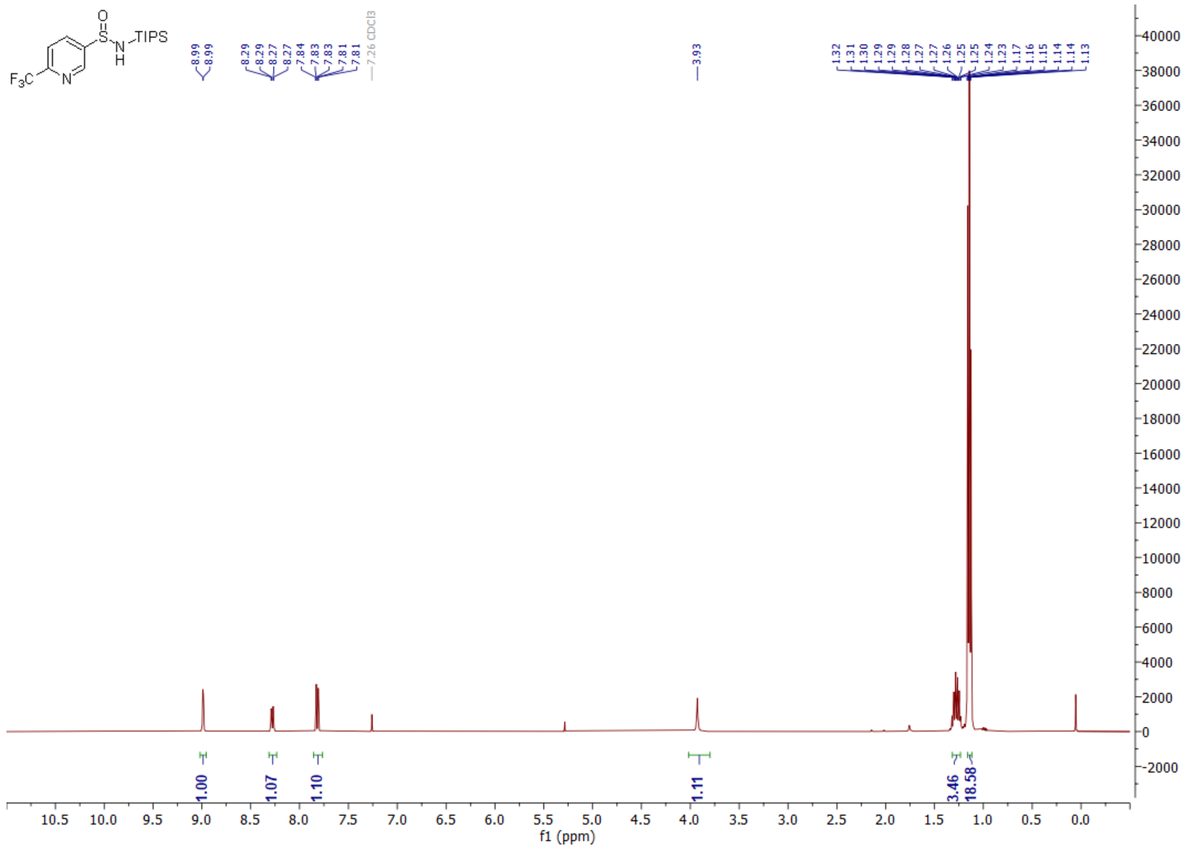

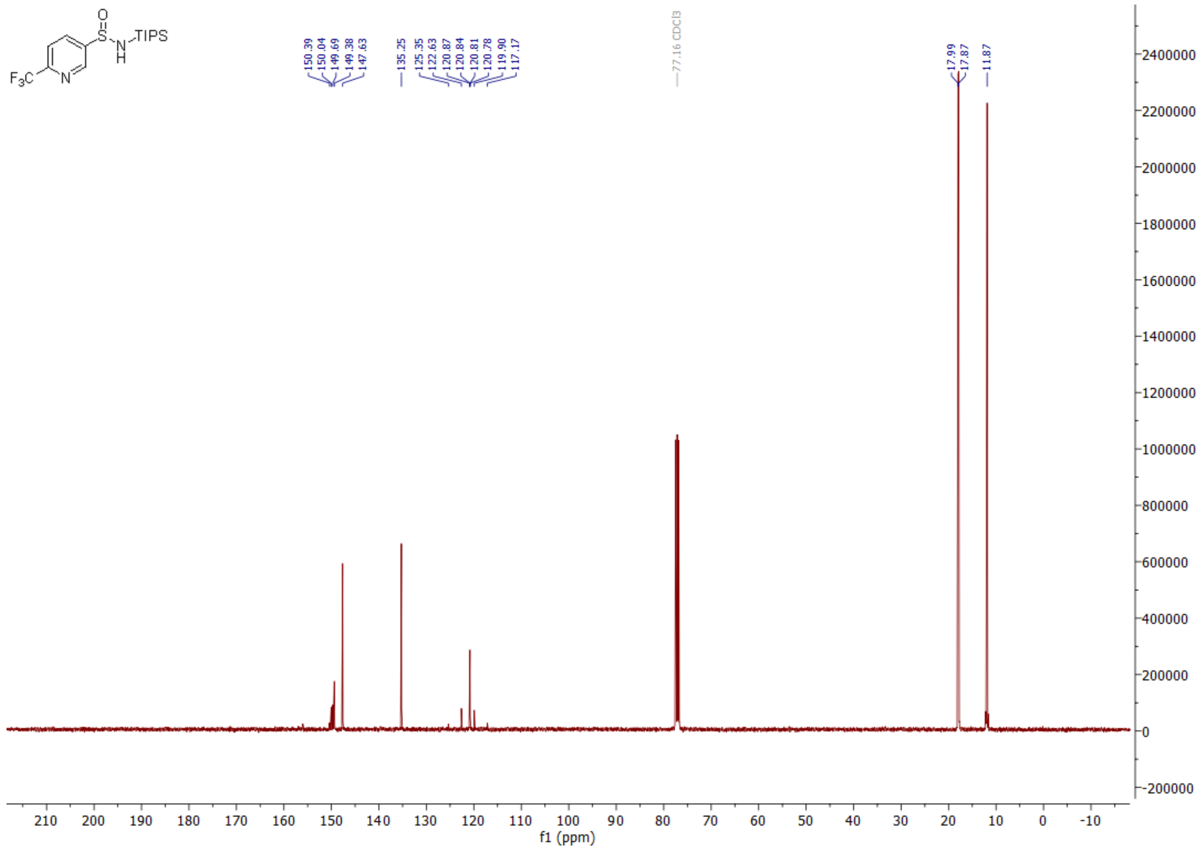

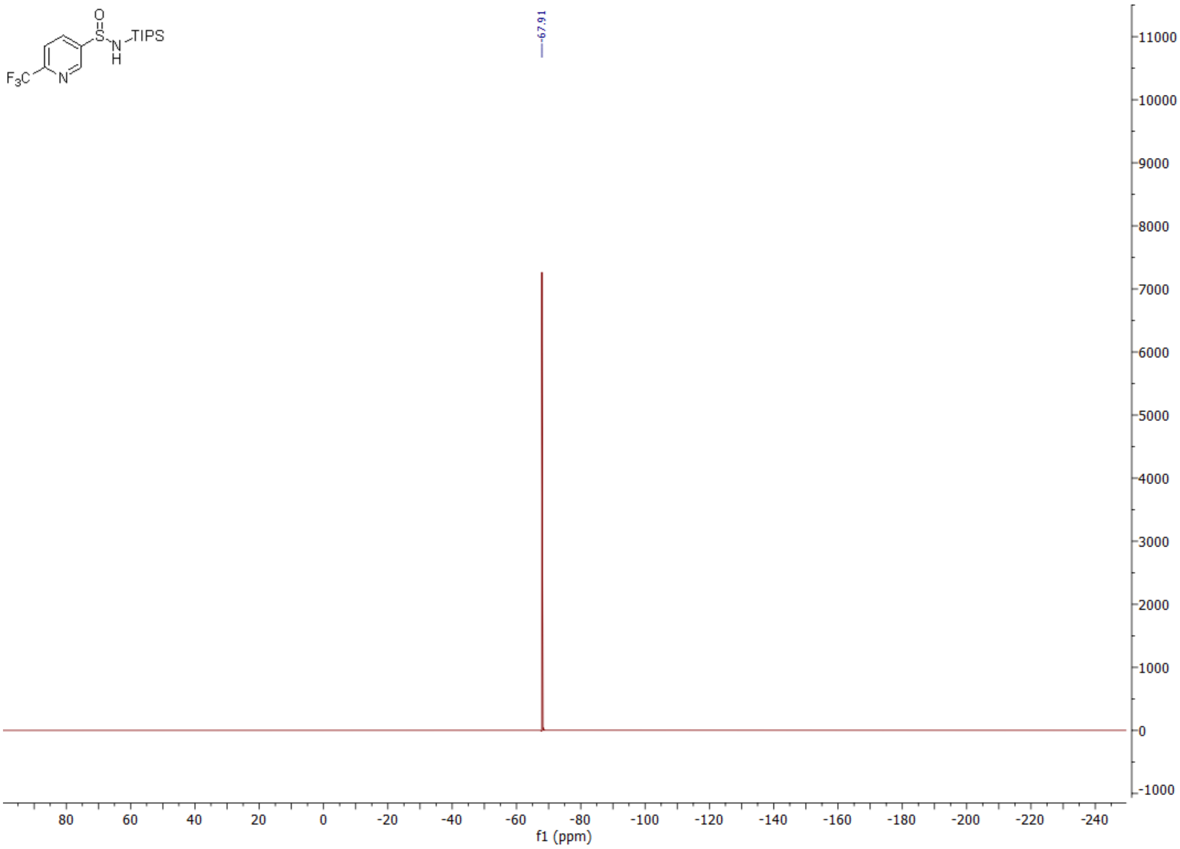

Tert-butyl (5-(((triisopropylsilyl)amino)sulfinyl)pyridin-2-yl)carbamate (1t)

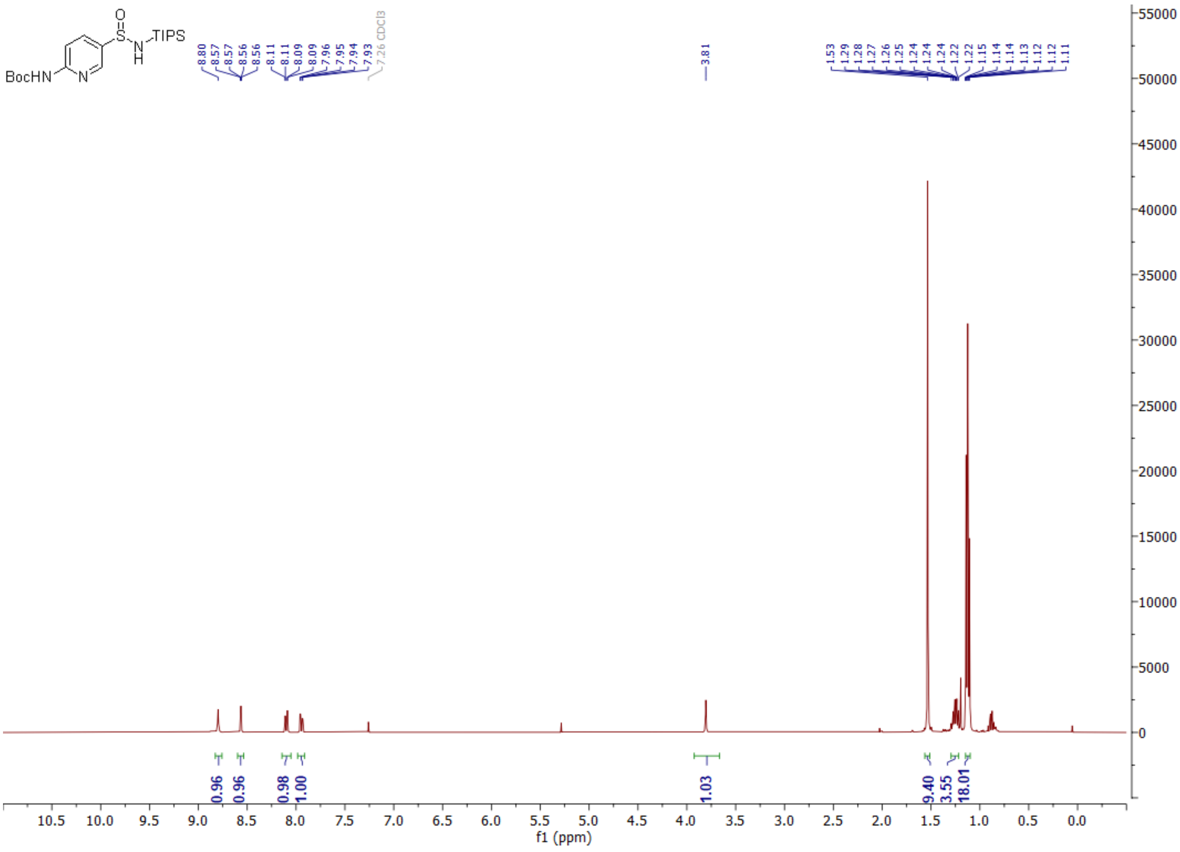

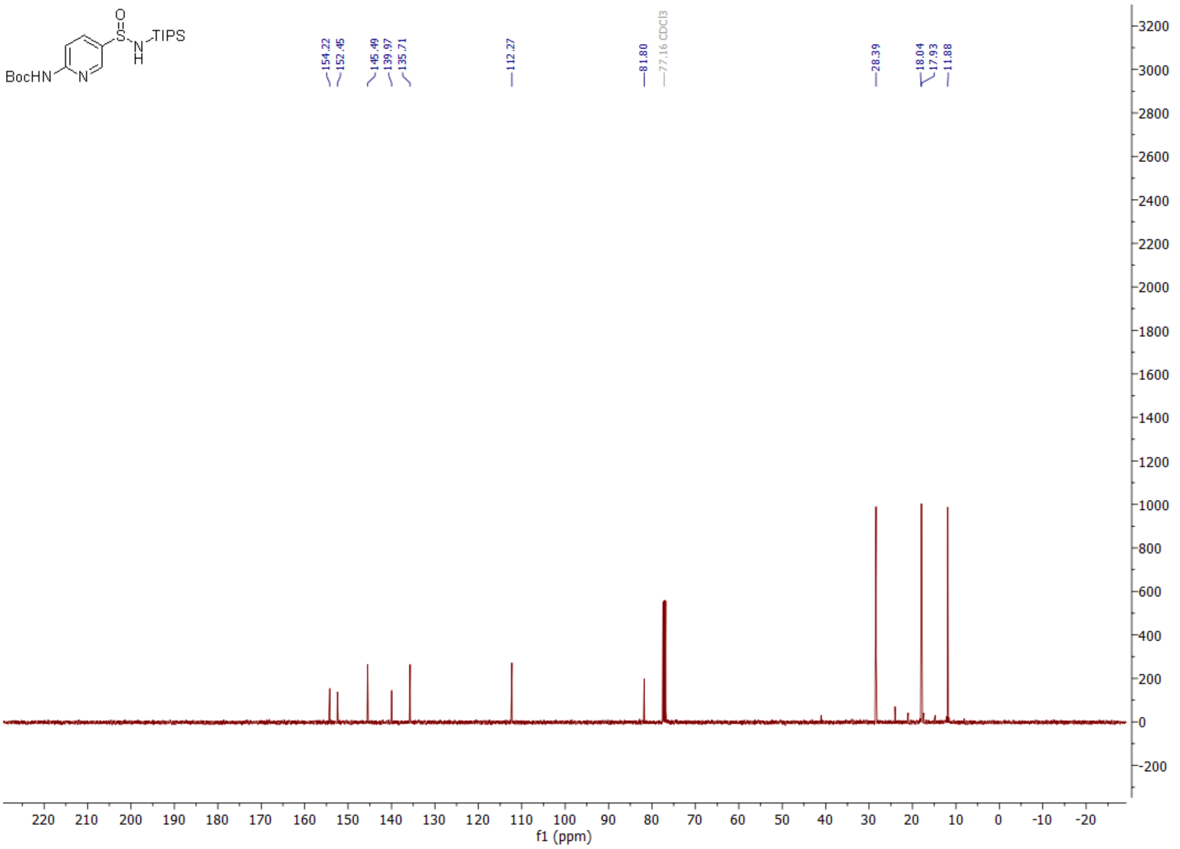

6-(Tert-butyl)-*N*-(triisopropylsilyl)pyridine-2-sulfinamide (1u)

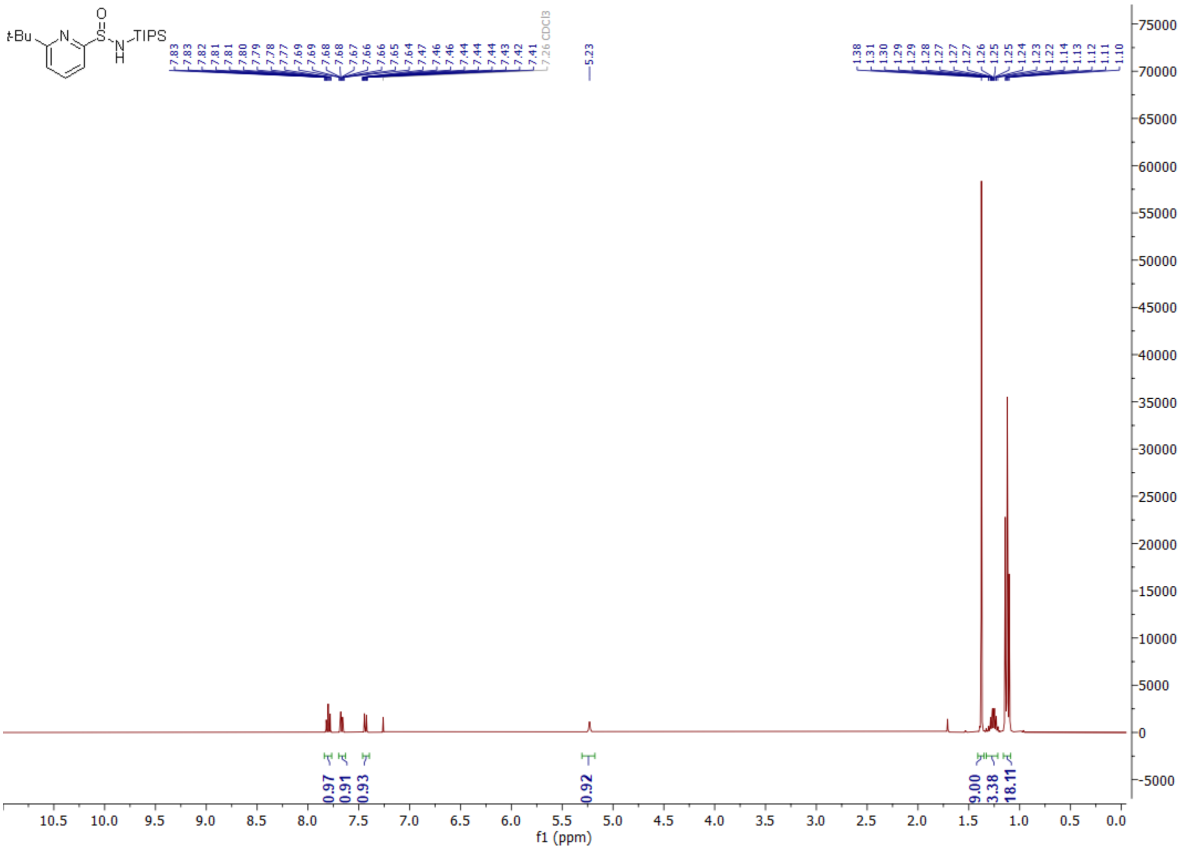

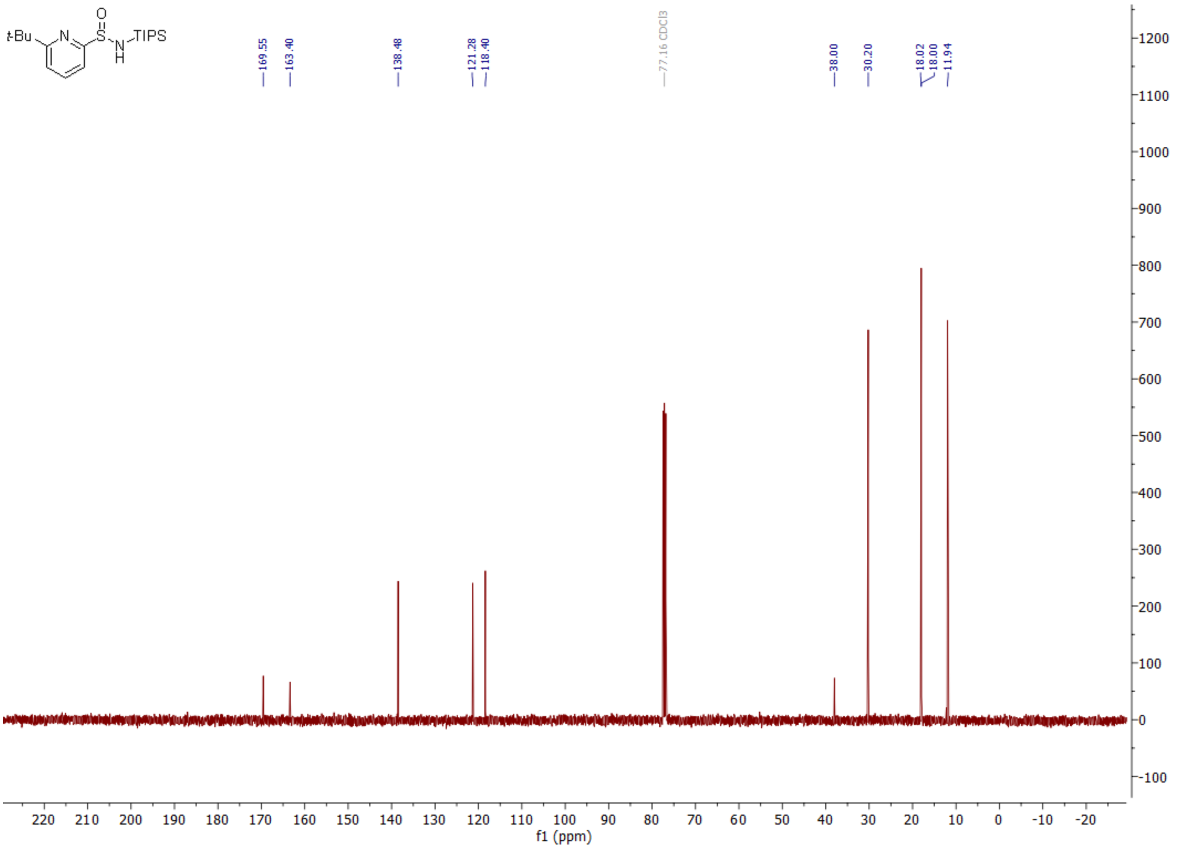

2-Methoxy-*N*-(triisopropylsilyl)pyrimidine-5-sulfinamide (1v)

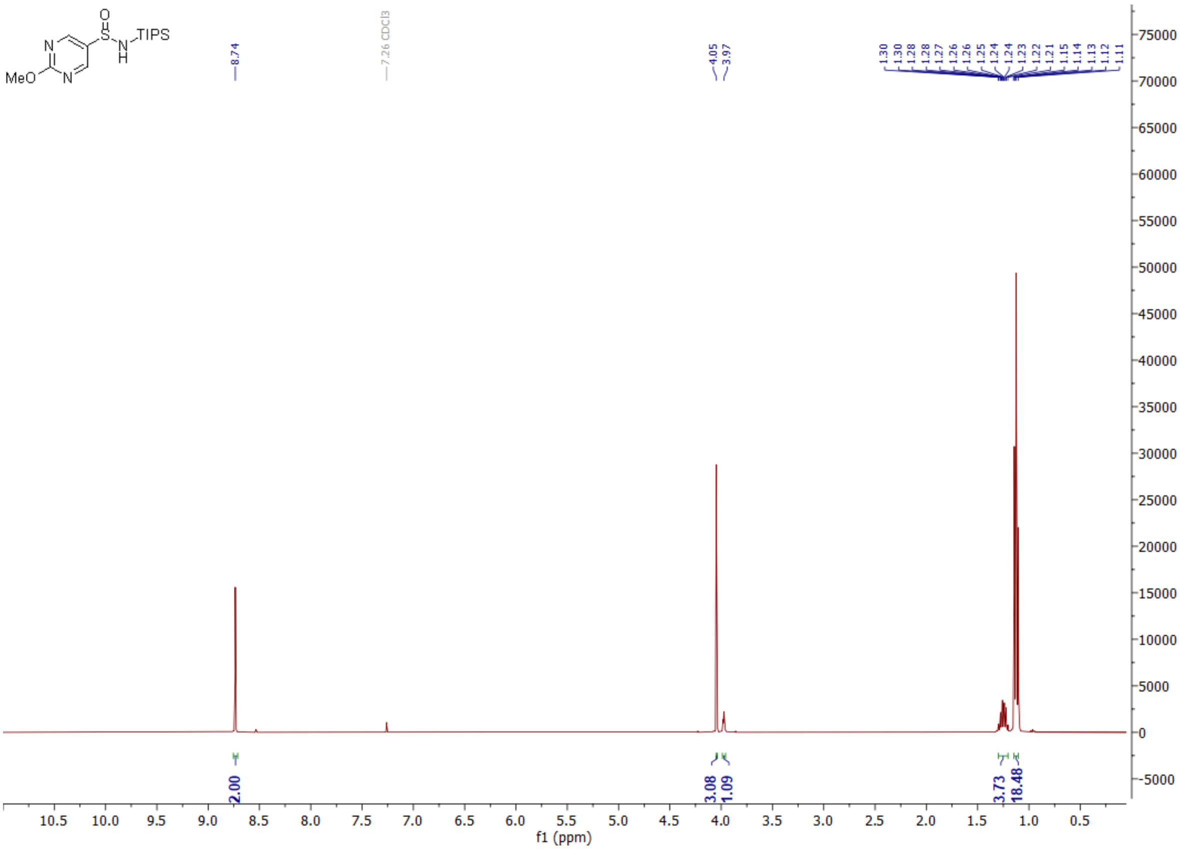

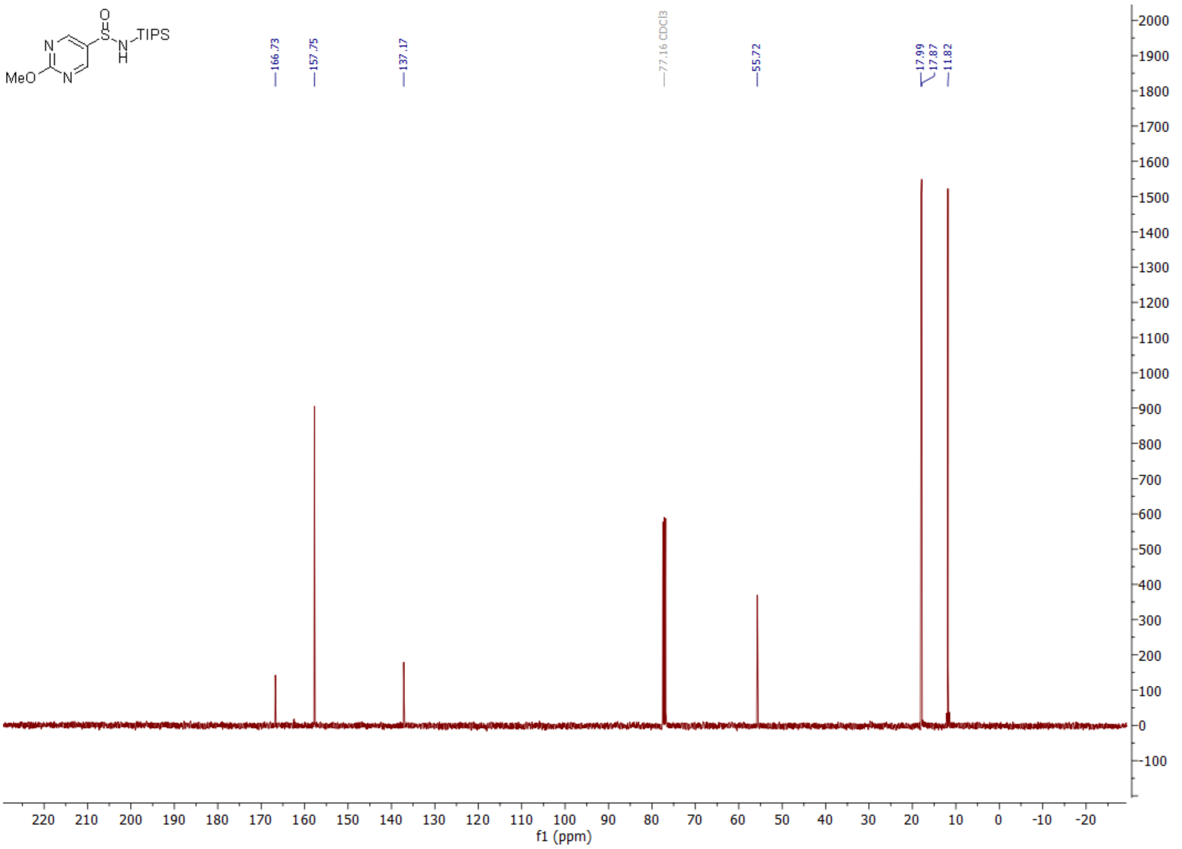

N-(Triisopropylsilyl)quinoline-6-sulfinamide (1w)

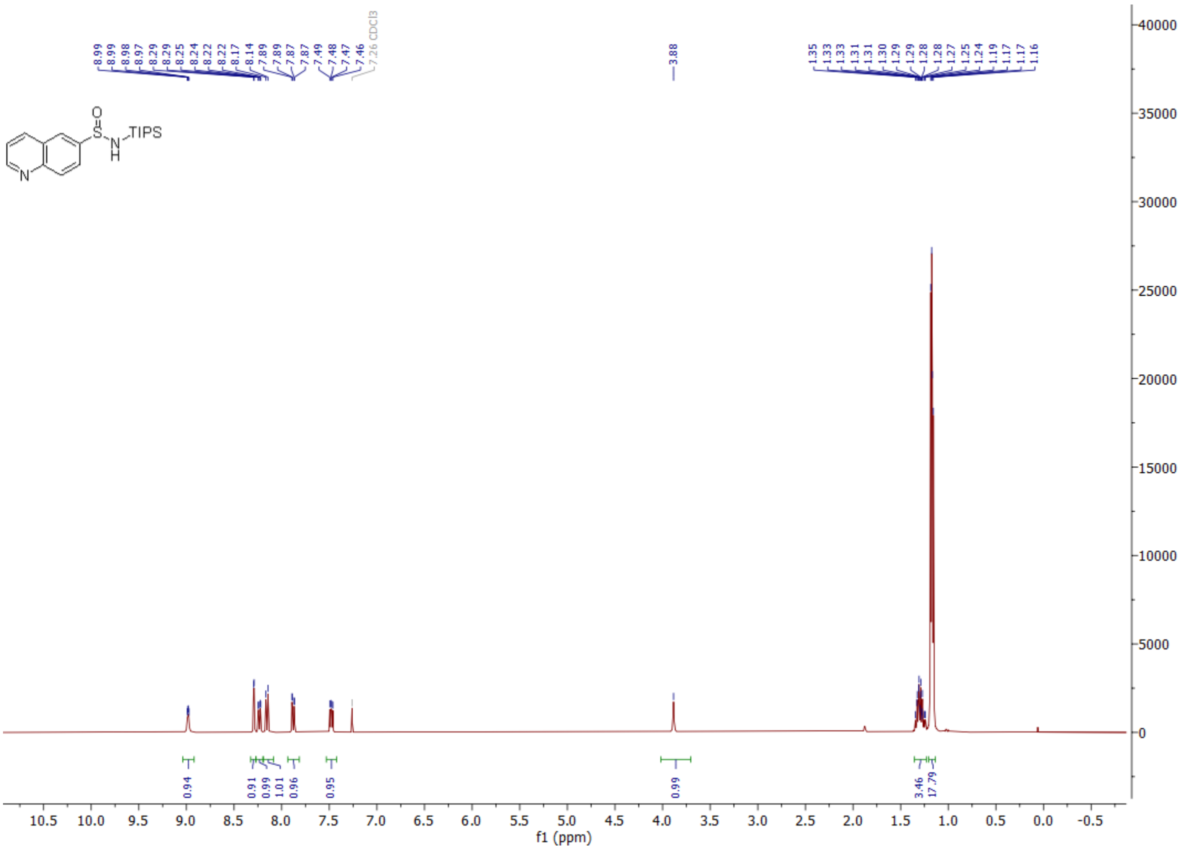

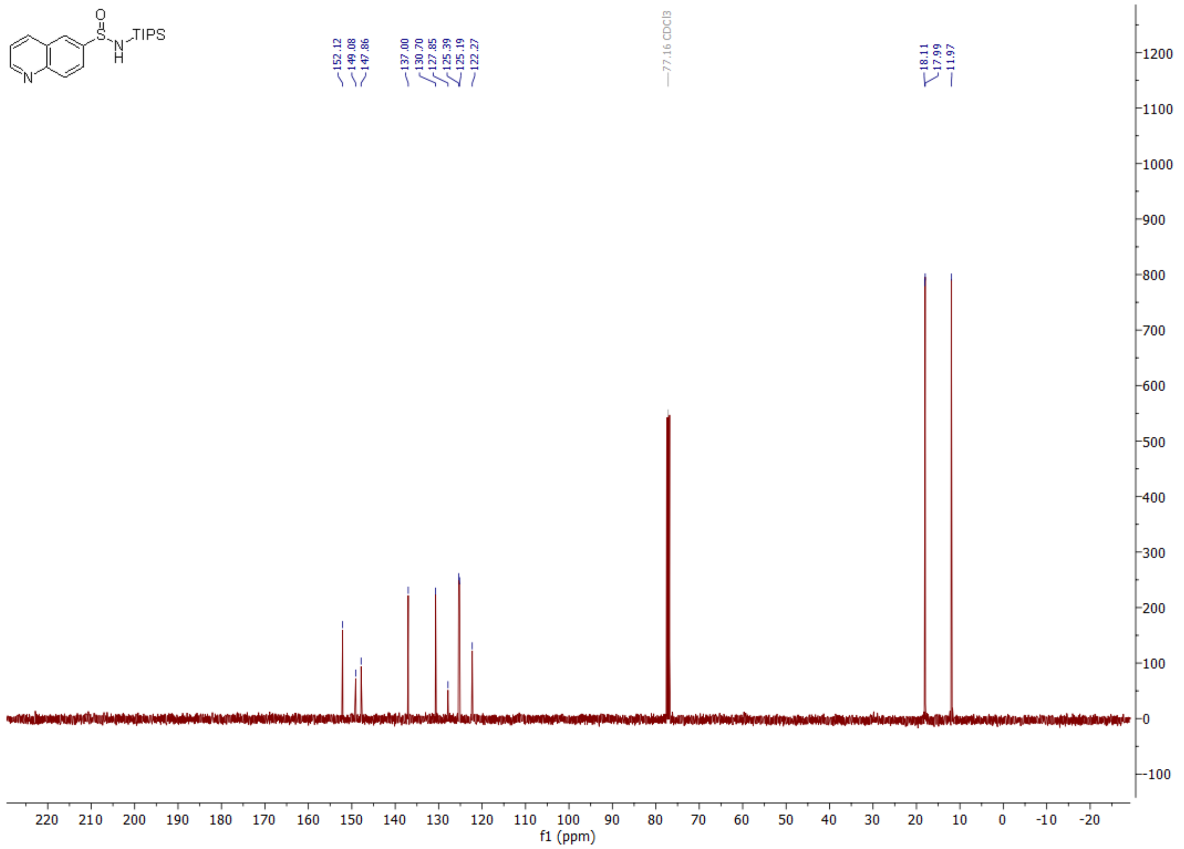

1-Acetyl-*N*-(triisopropylsilyl)-1H-indole-5-sulfinamide (1x)

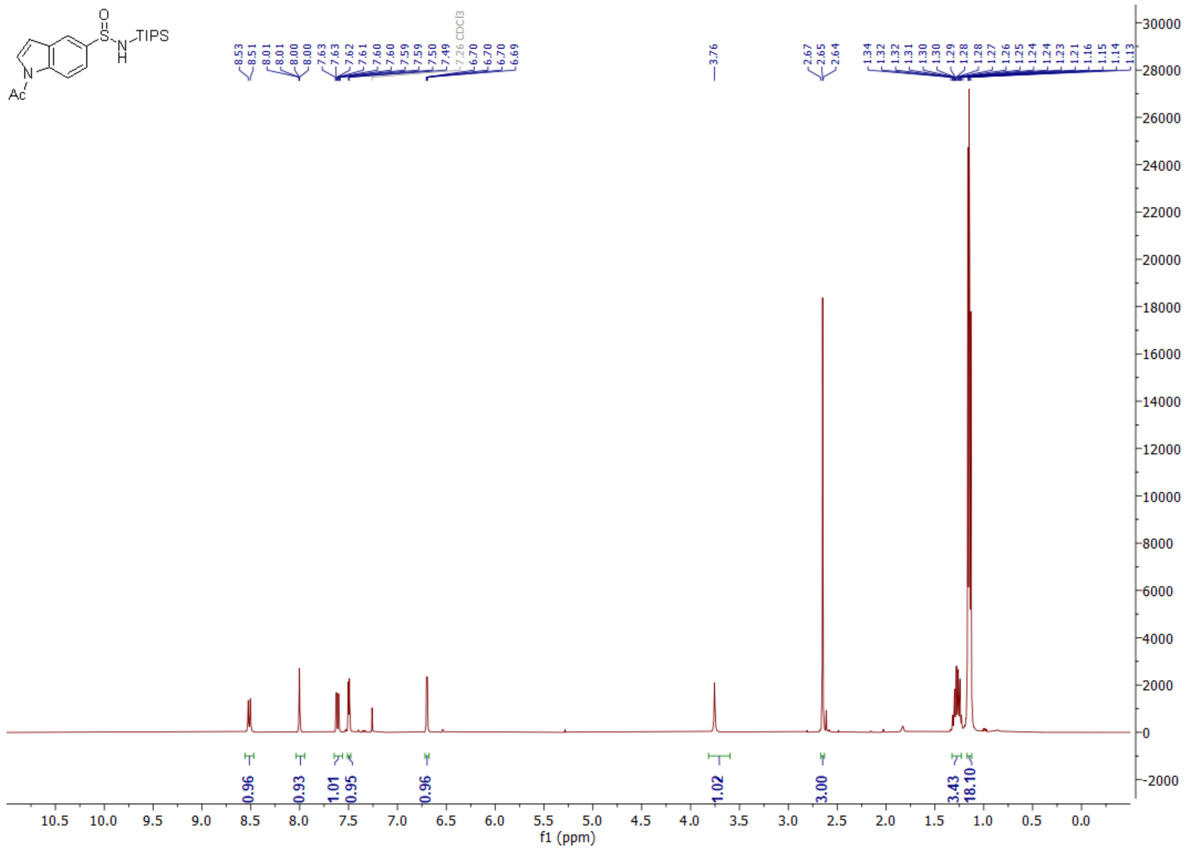

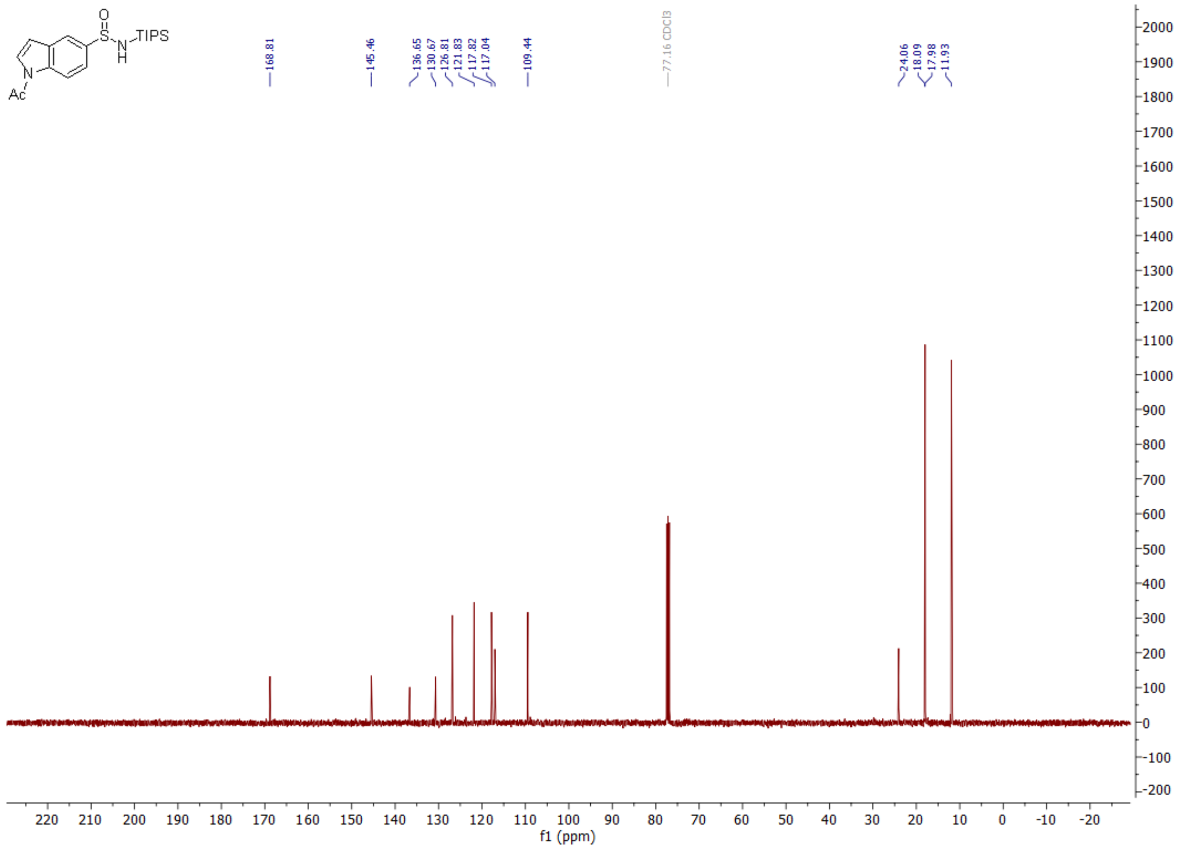

N-(triisopropylsilyl)thiophene-3-sulfinamide (1y)

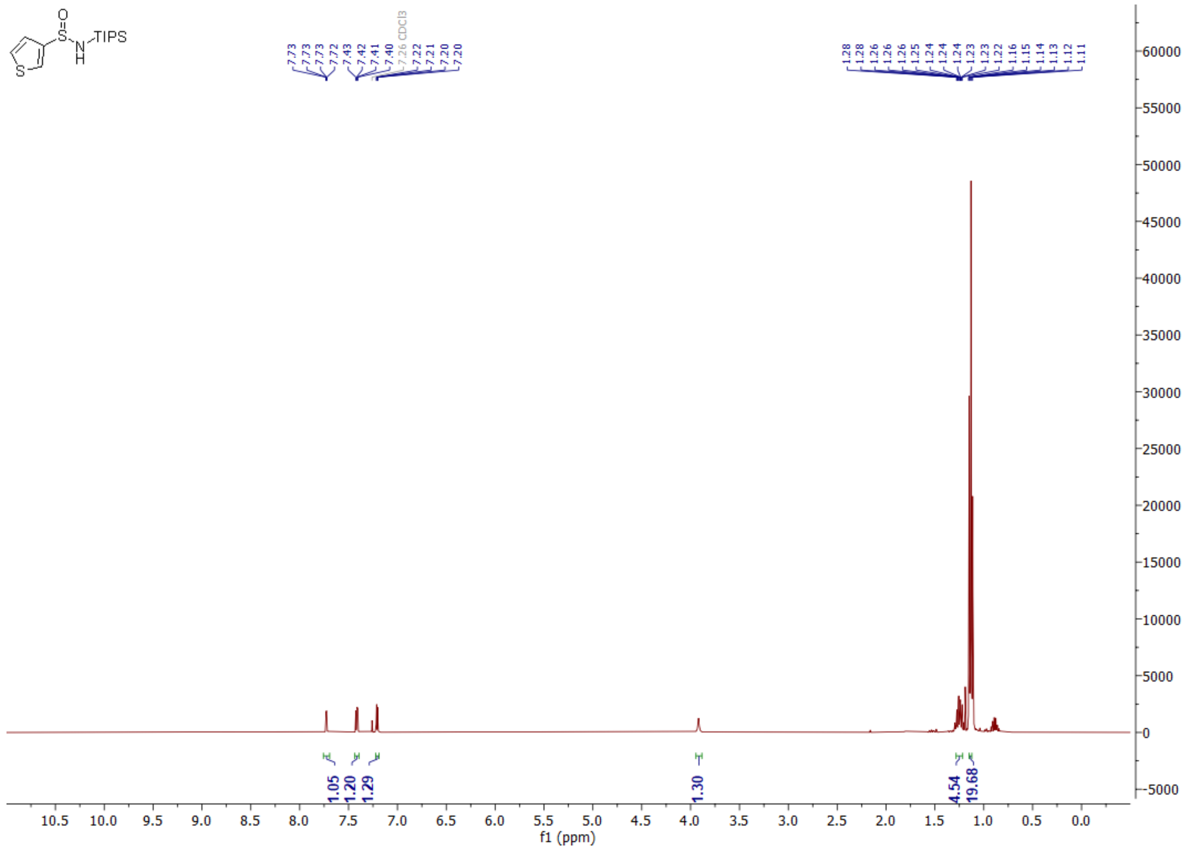

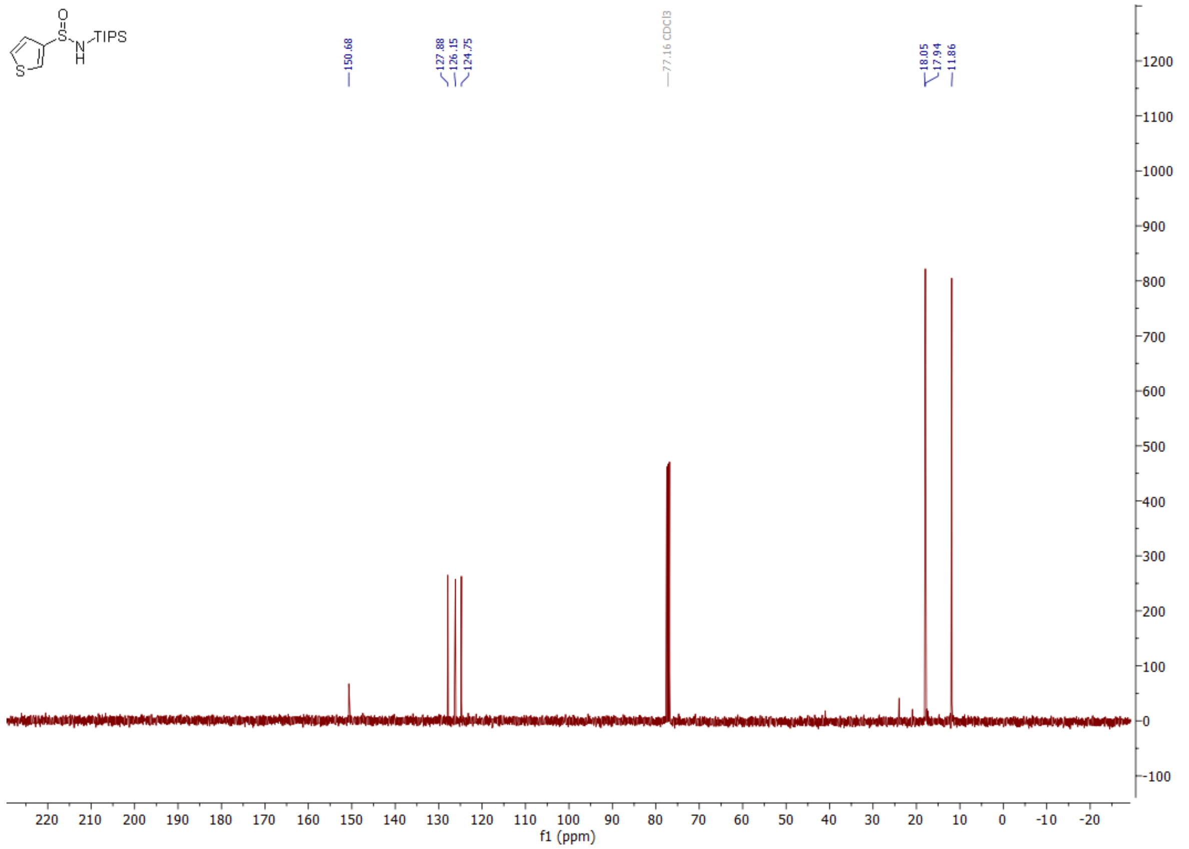

4-(5-(*p*-Tolyl)-3-(trifluoromethyl)-1H-pyrazol-1-yl)-*N*-(triisopropylsilyl)benzenesulfinamide (1z)

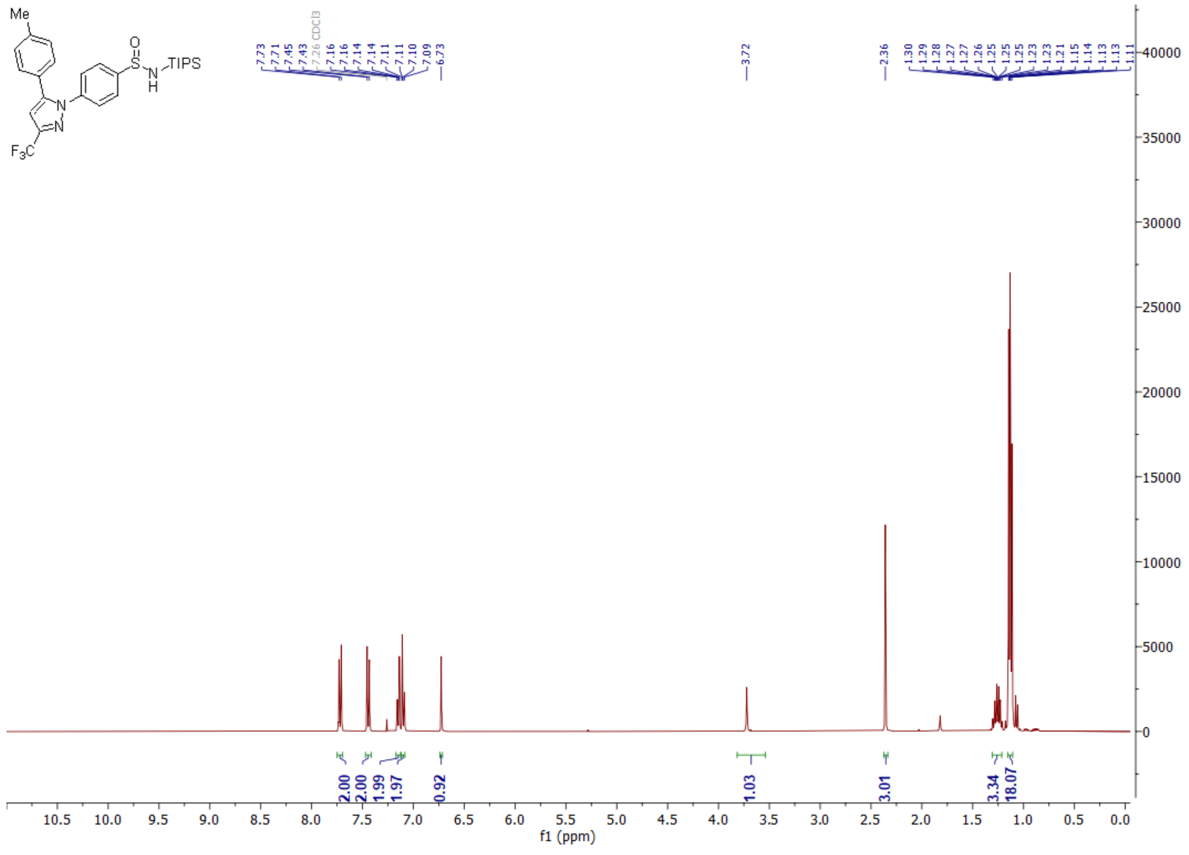

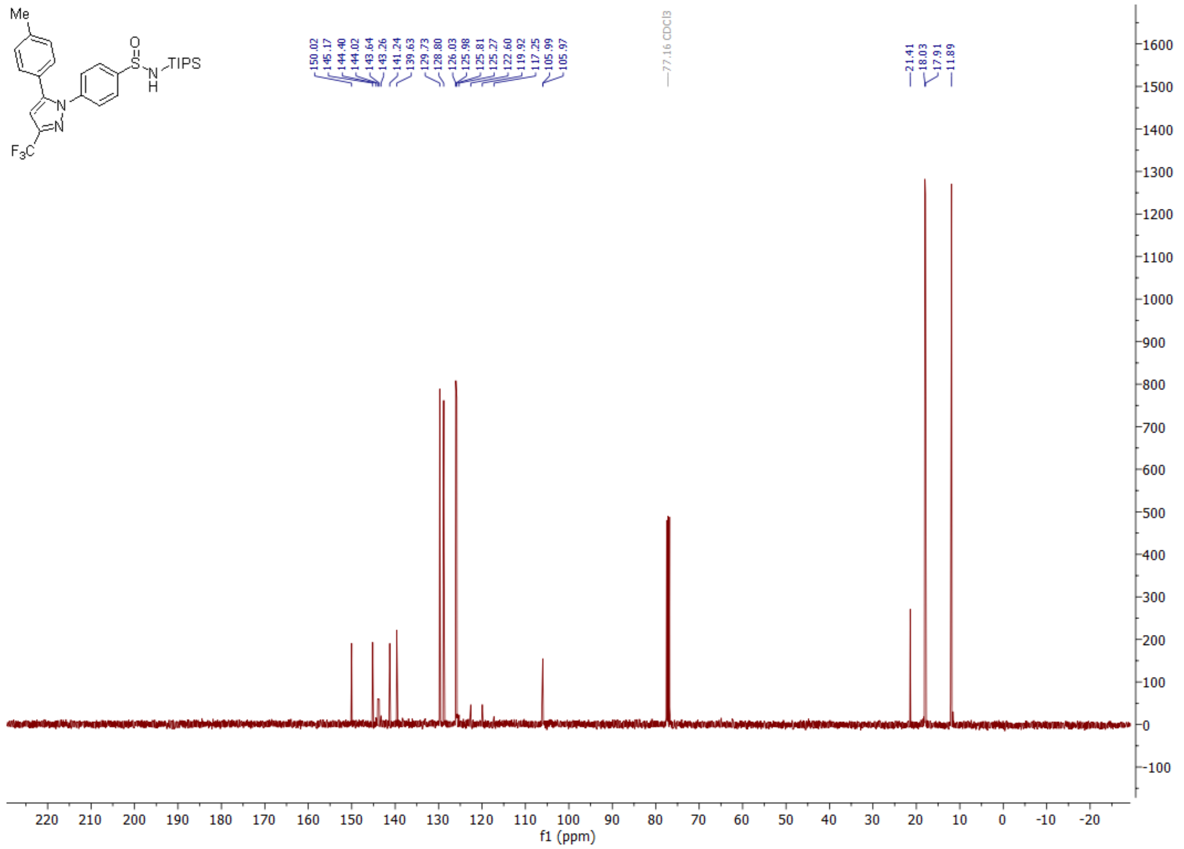
Me
TIPS
F3C
150.02
145.17
144.40
144.02
143.64
143.26
141.24
139.63
129.73
128.80
126.03
125.98
125.81
125.27
122.60
119.92
117.25
105.99
105.97
77.16 CDCl3
21.41
18.03
17.91
11.89
f1 (ppm)

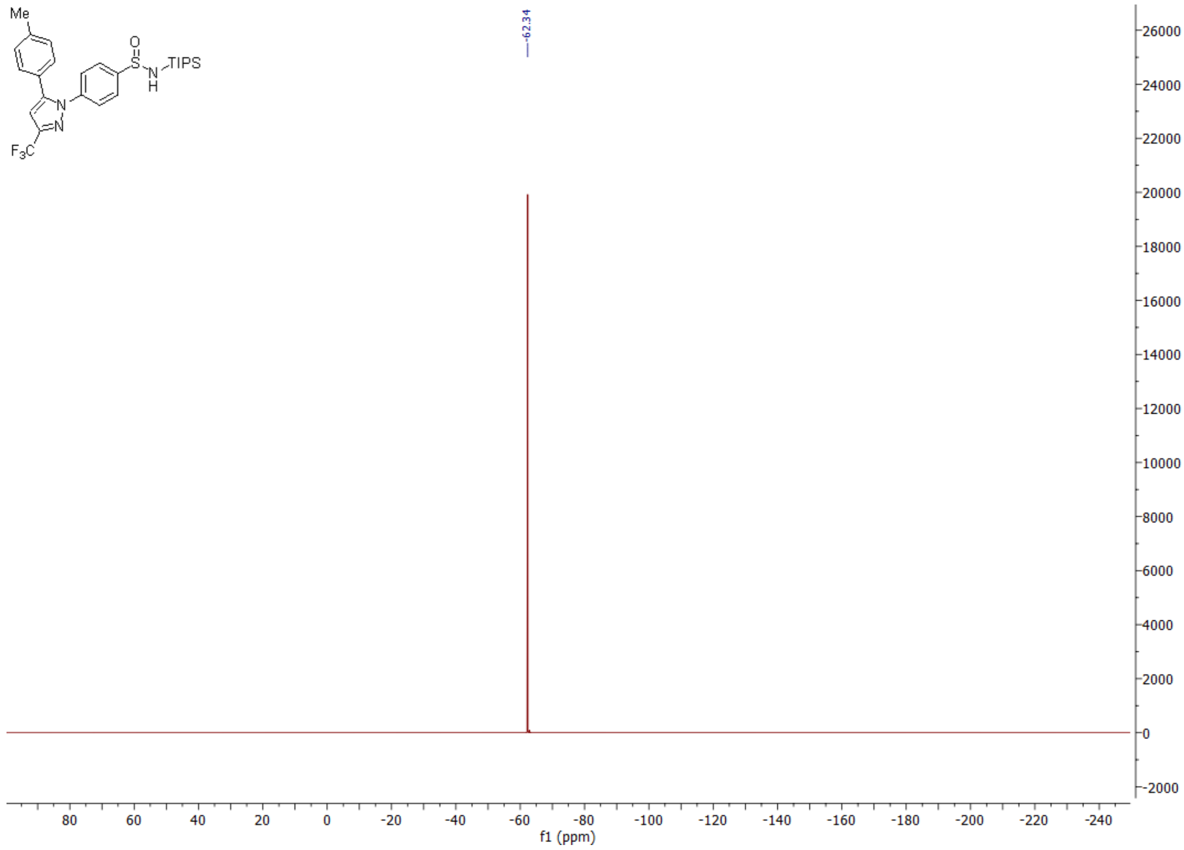
Me
TIPS
F3C
-62.34
f1 (ppm)

Tert-butyl 4-((5-(((triisopropylsilyl)amino)sulfinyl)pyridin-2-yl)oxy)piperidine-1-carboxylate (1aa)

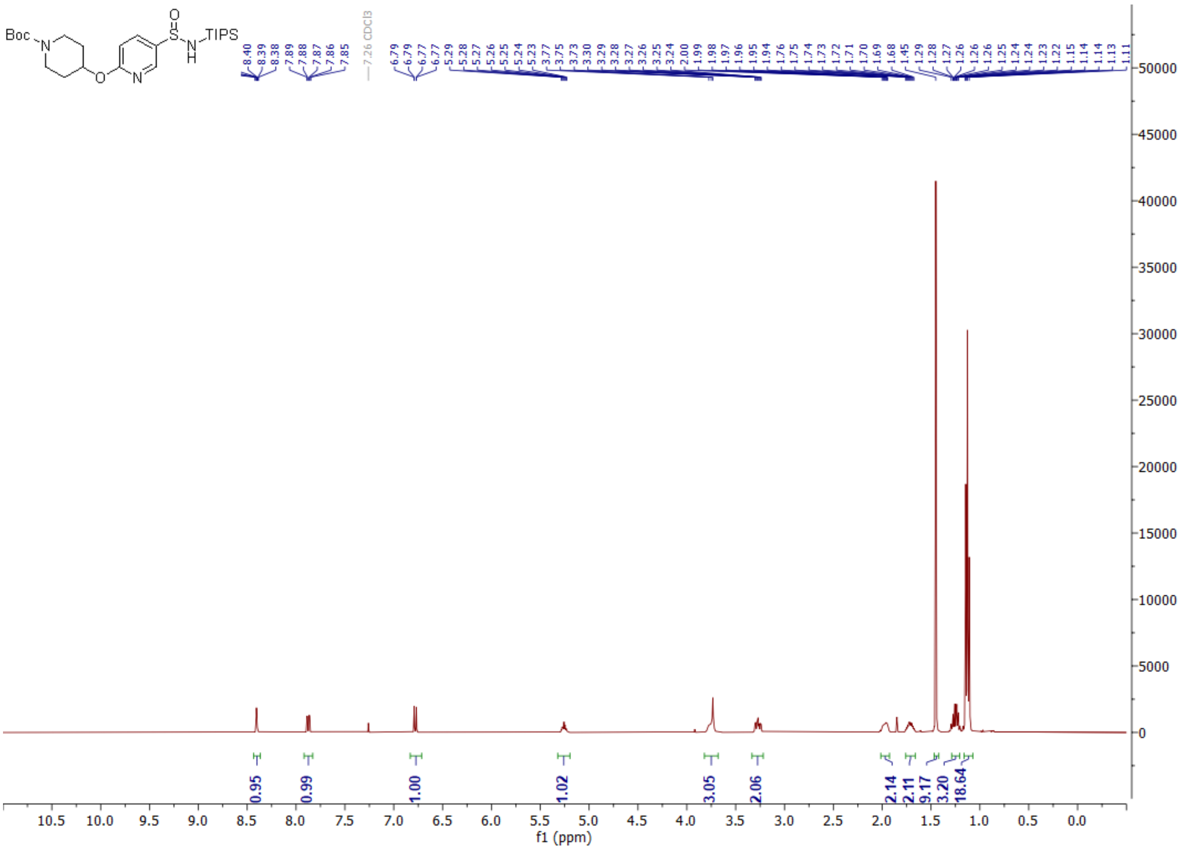

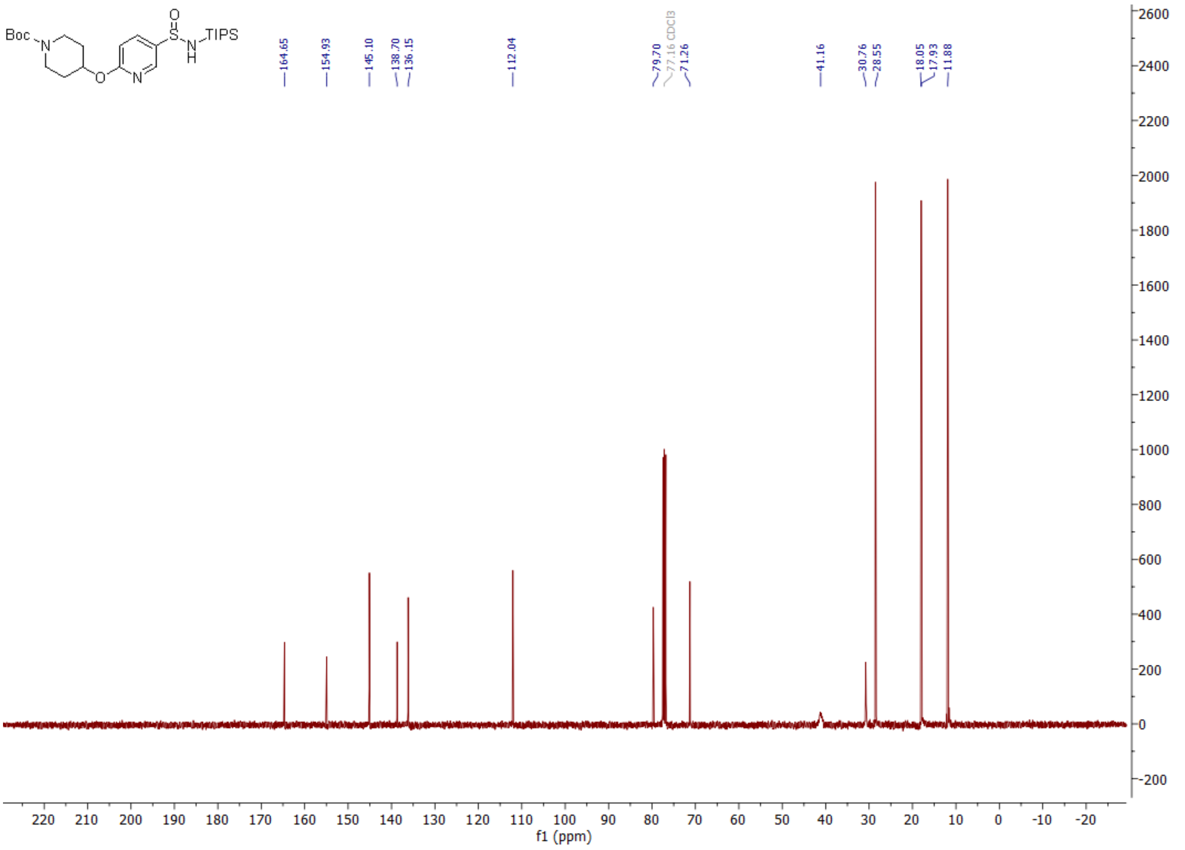

N-(4-(((Triisopropylsilyl)amino)sulfinyl)phenethyl)-6-(trimethylsilyl)hex-5-ynamide (1ab)

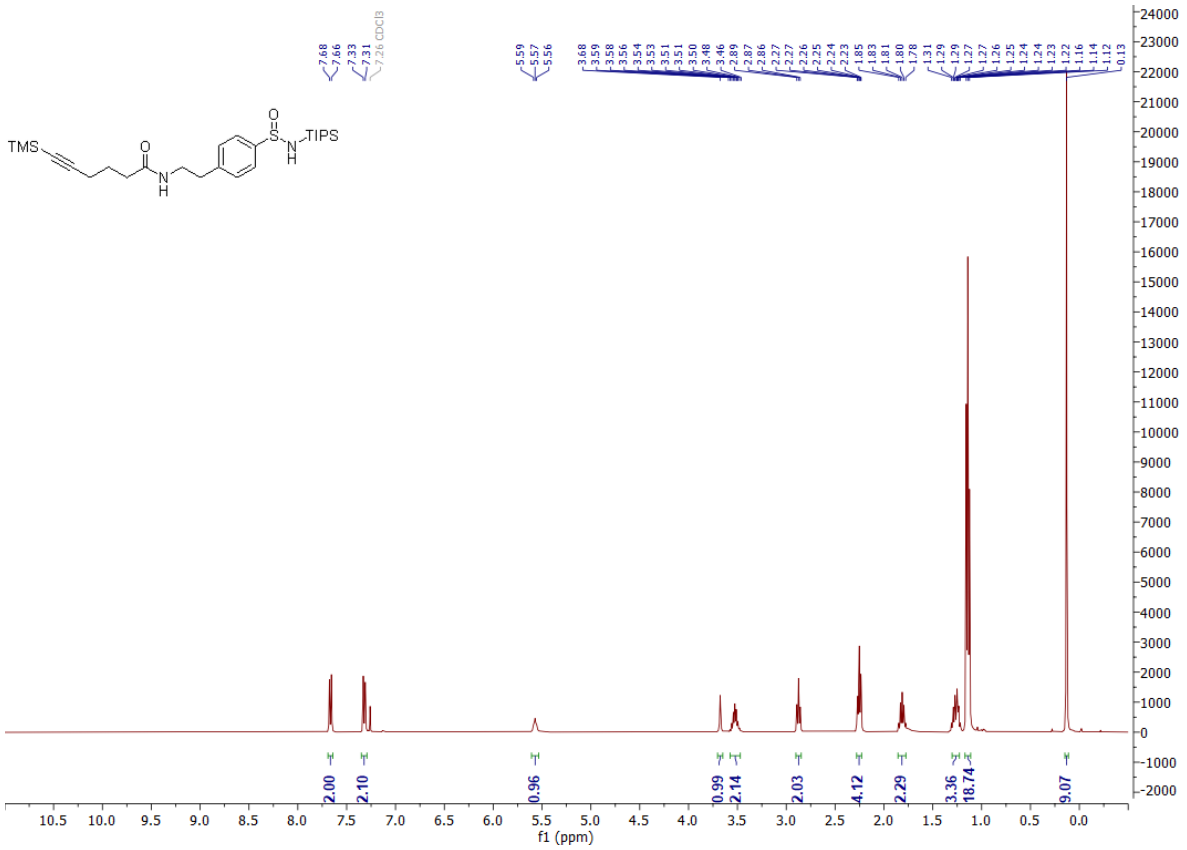

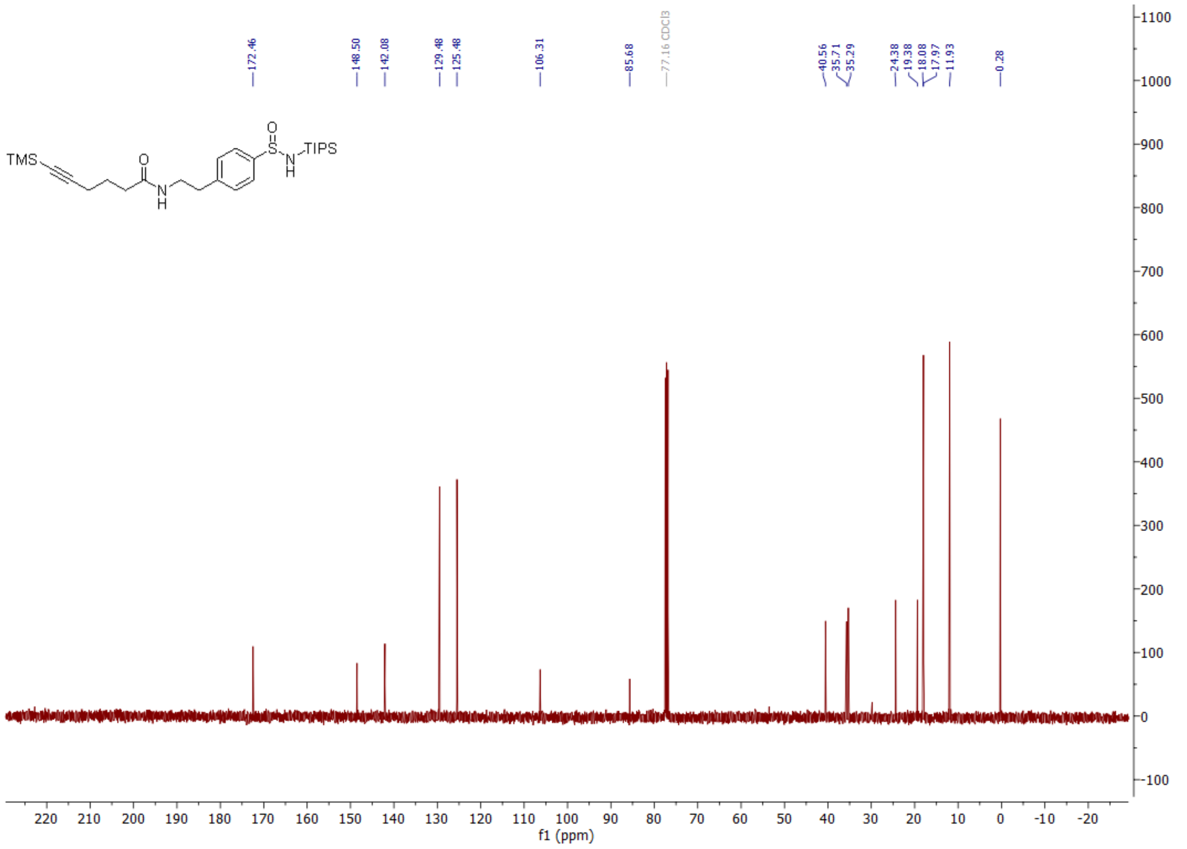

1-Tosyl-*N*-(triisopropylsilyl)-1,2,3,6-tetrahydropyridine-4-sulfinamide (3a)

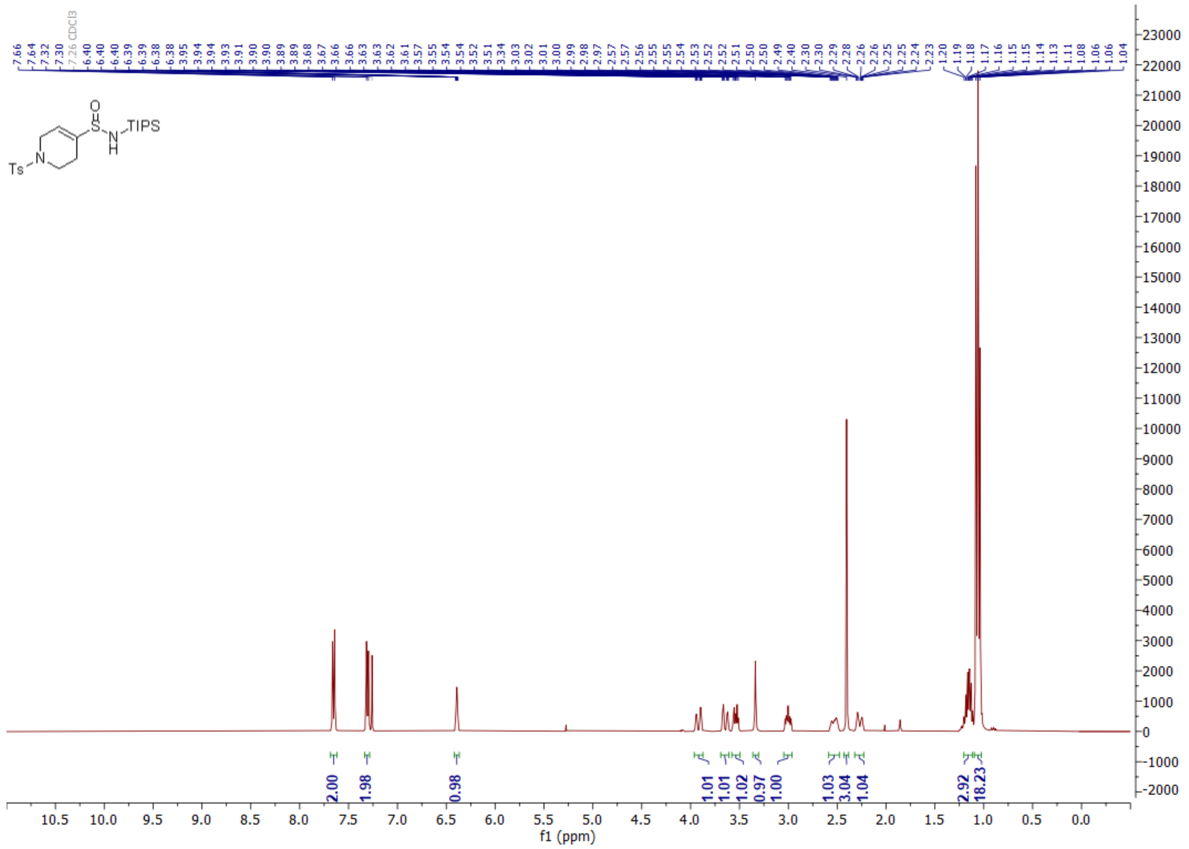

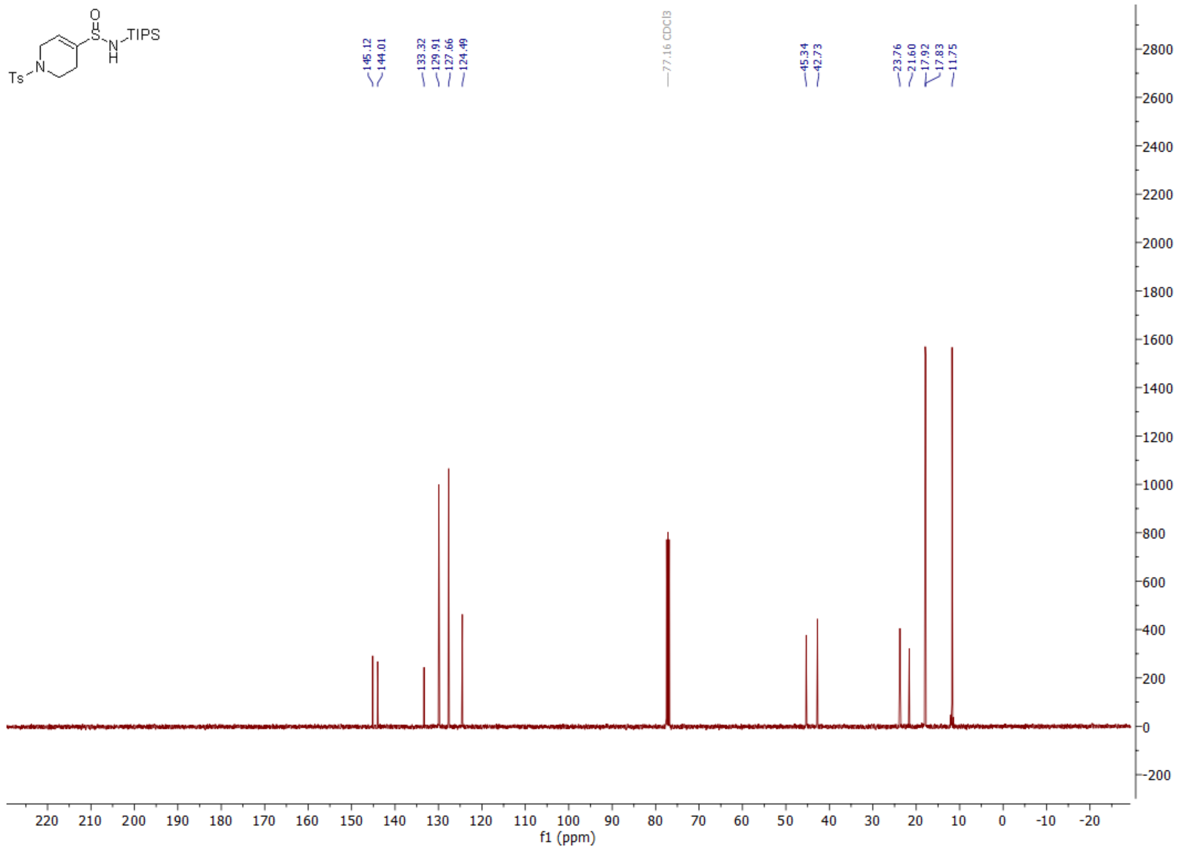

Tert-butyl 4-(((triisopropylsilyl)amino)sulfinyl)-3,6-dihydropyridine-1(2H)-carboxylate (3b)

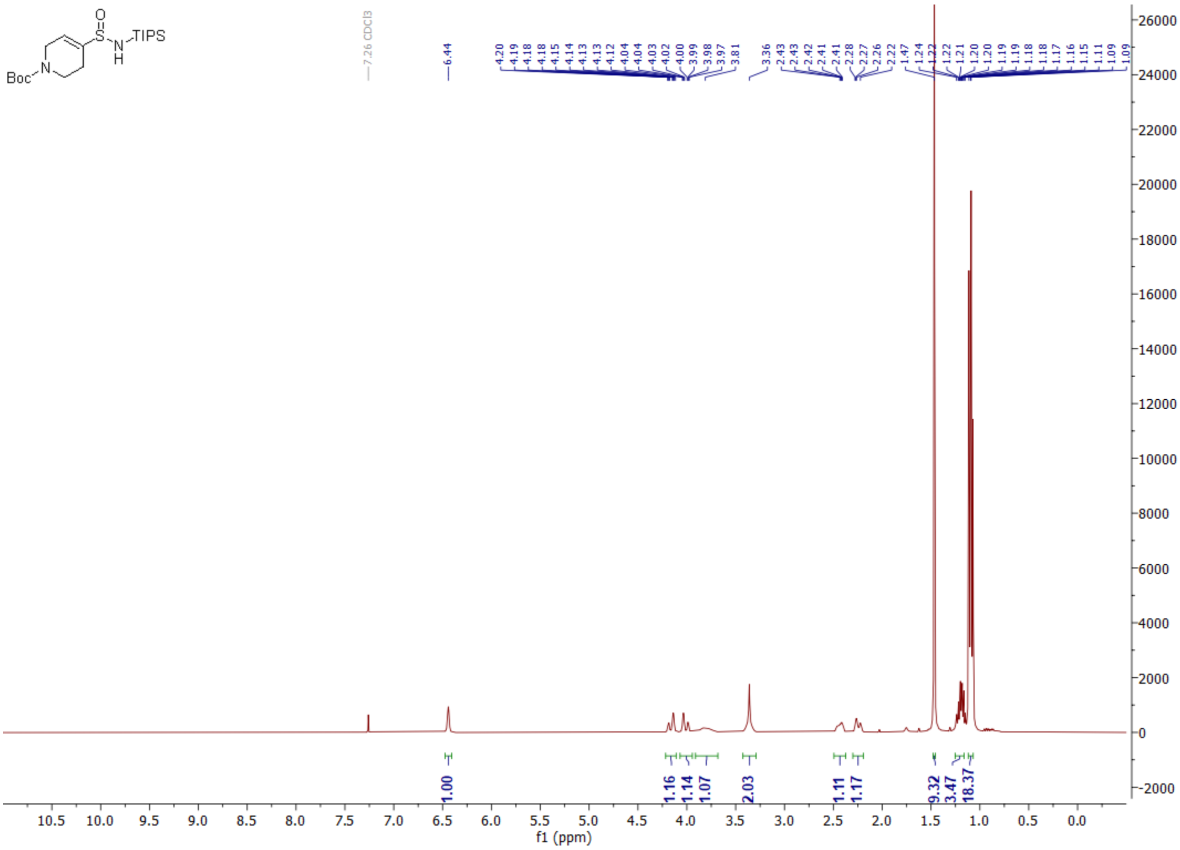

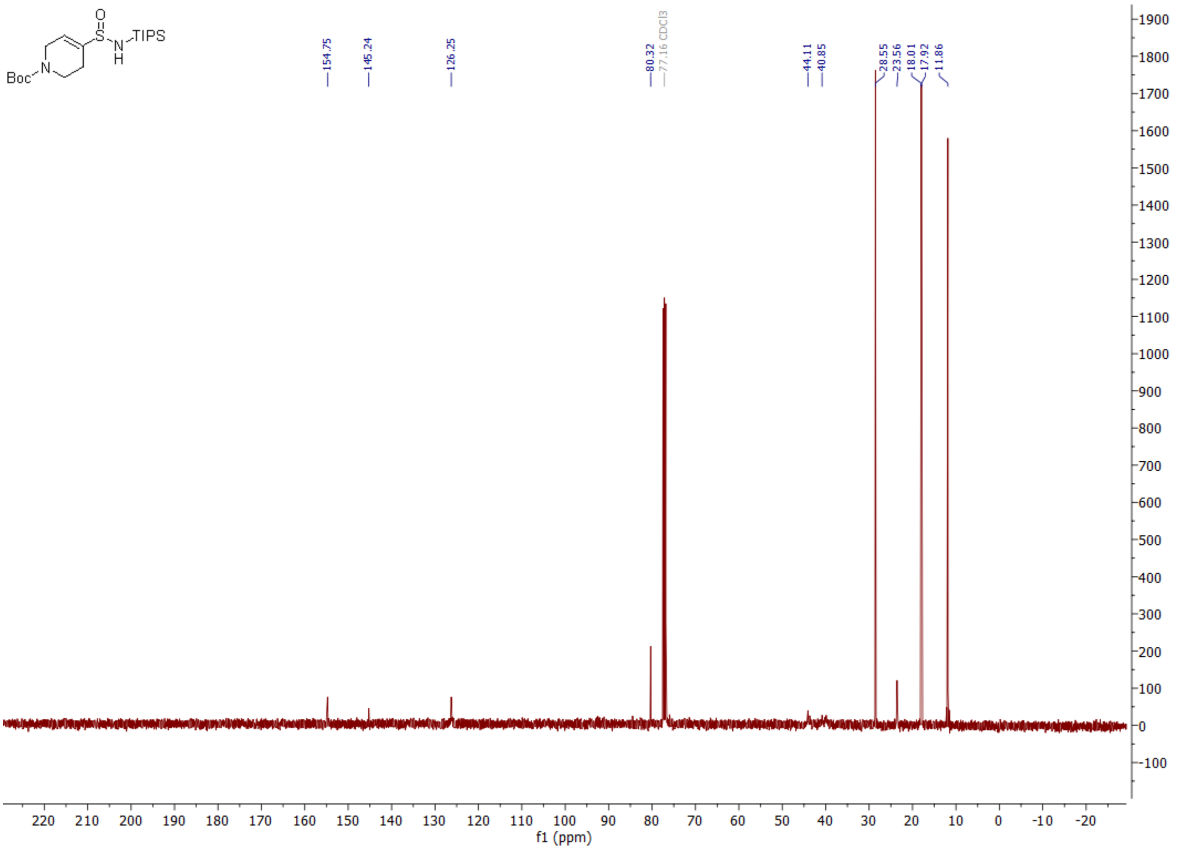

1-Benzyl-*N*-(triisopropylsilyl)-1,2,3,6-tetrahydropyridine-4-sulfinamide (3c)

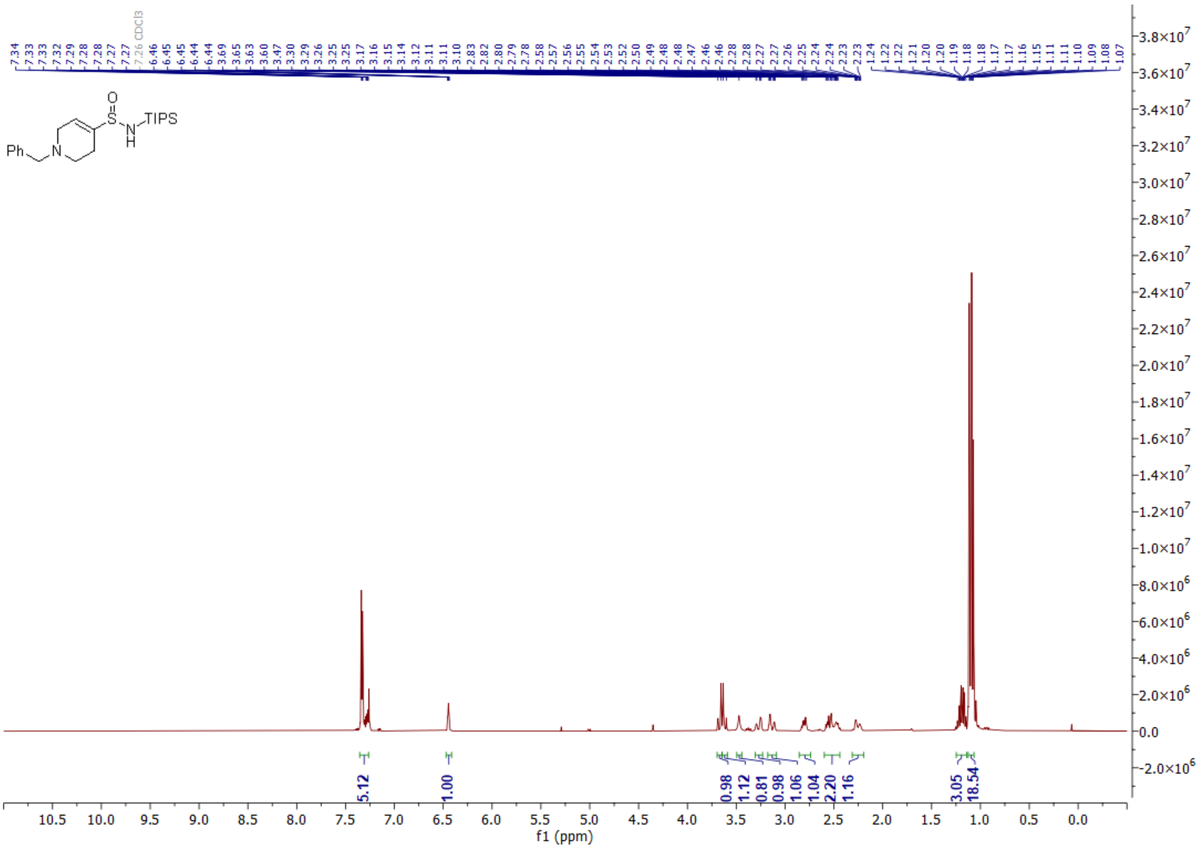

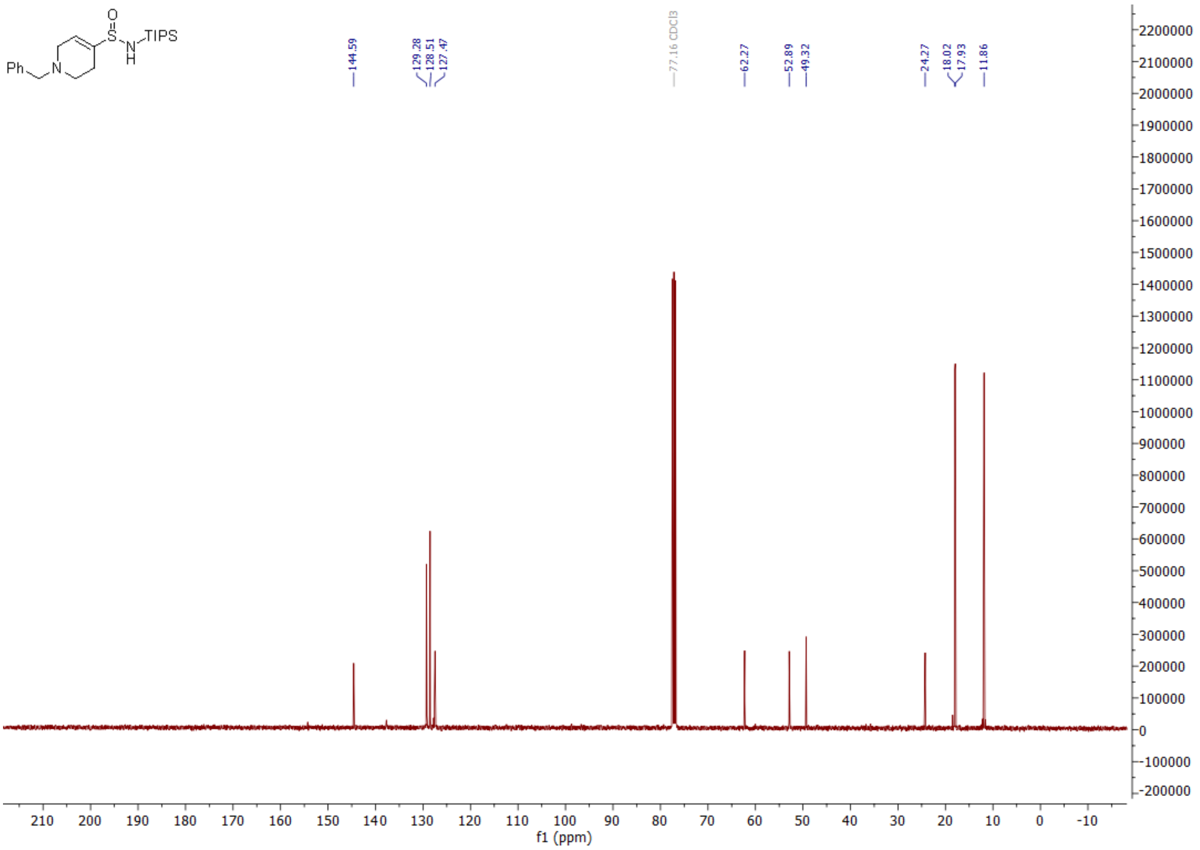

N-(Triisopropylsilyl)cyclopent-1-ene-1-sulfinamide (3d)

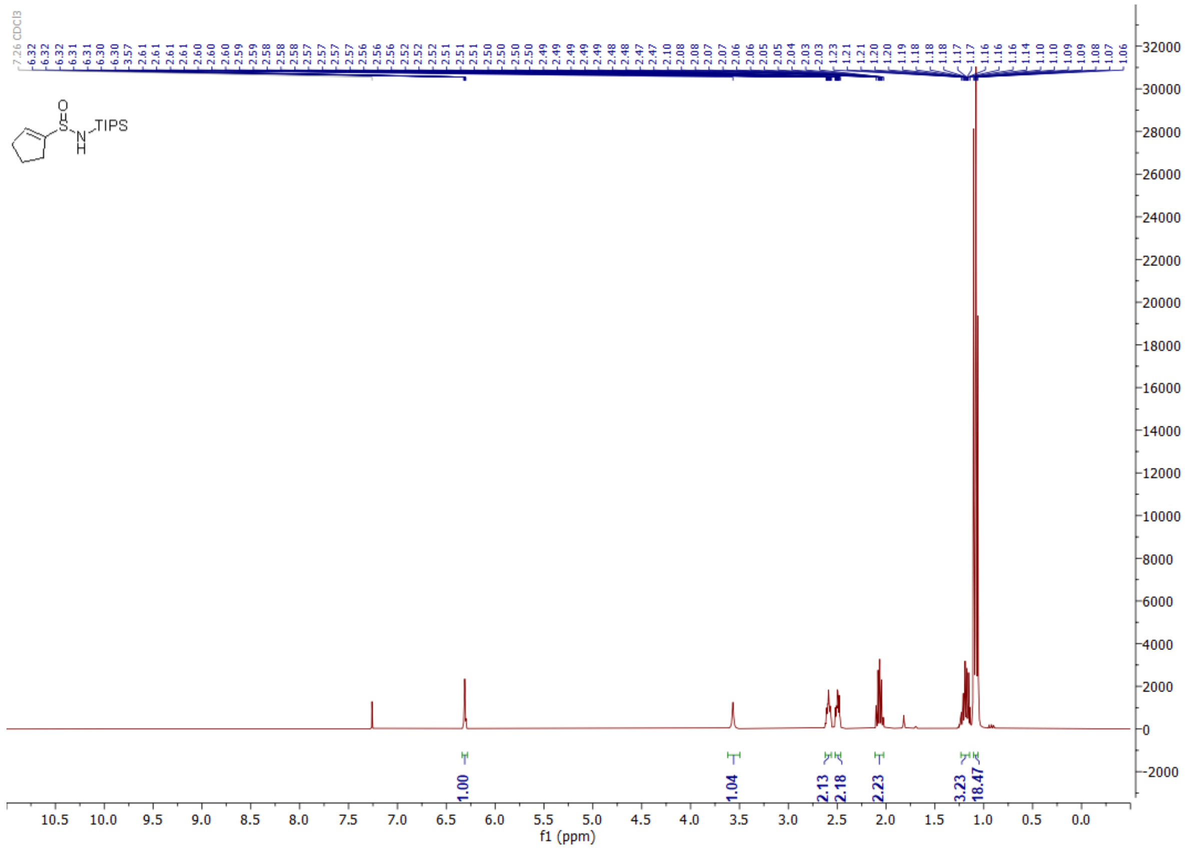

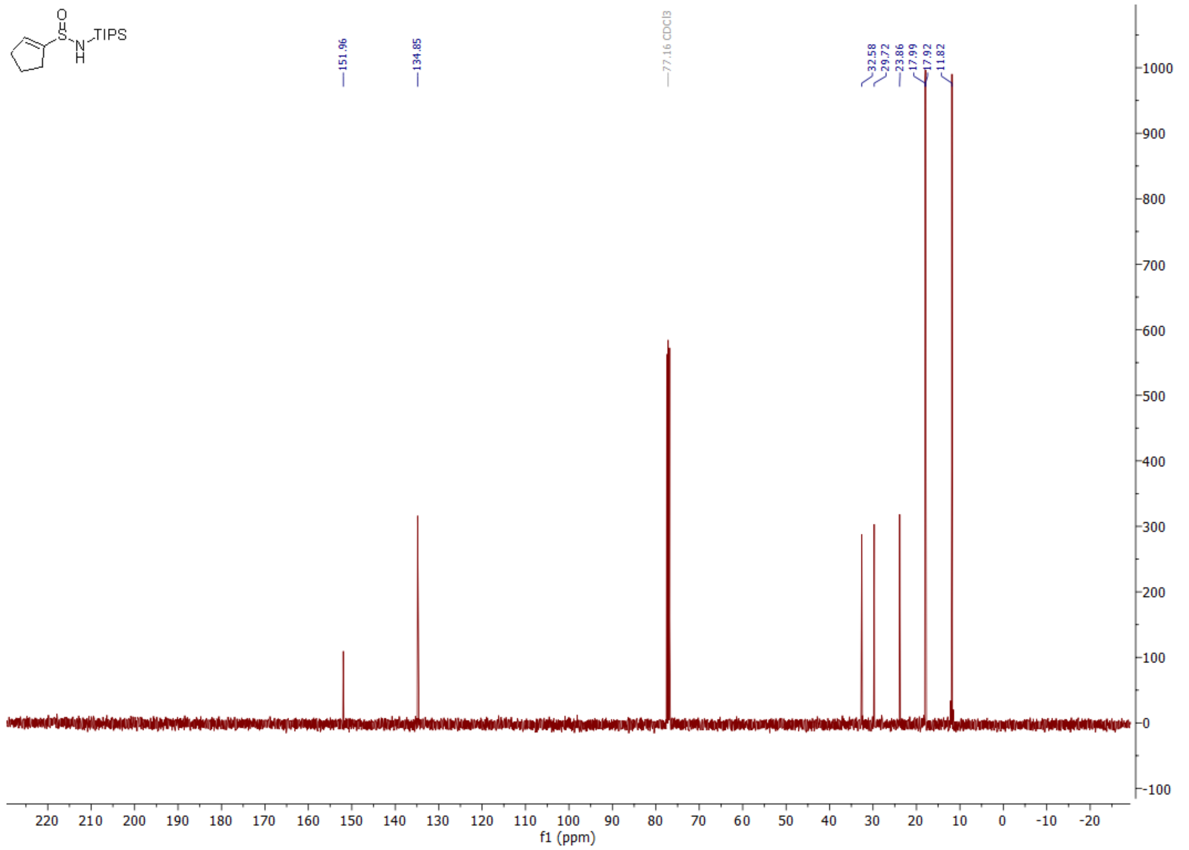

N-(Triisopropylsilyl)cyclohex-1-ene-1-sulfinamide (3e)

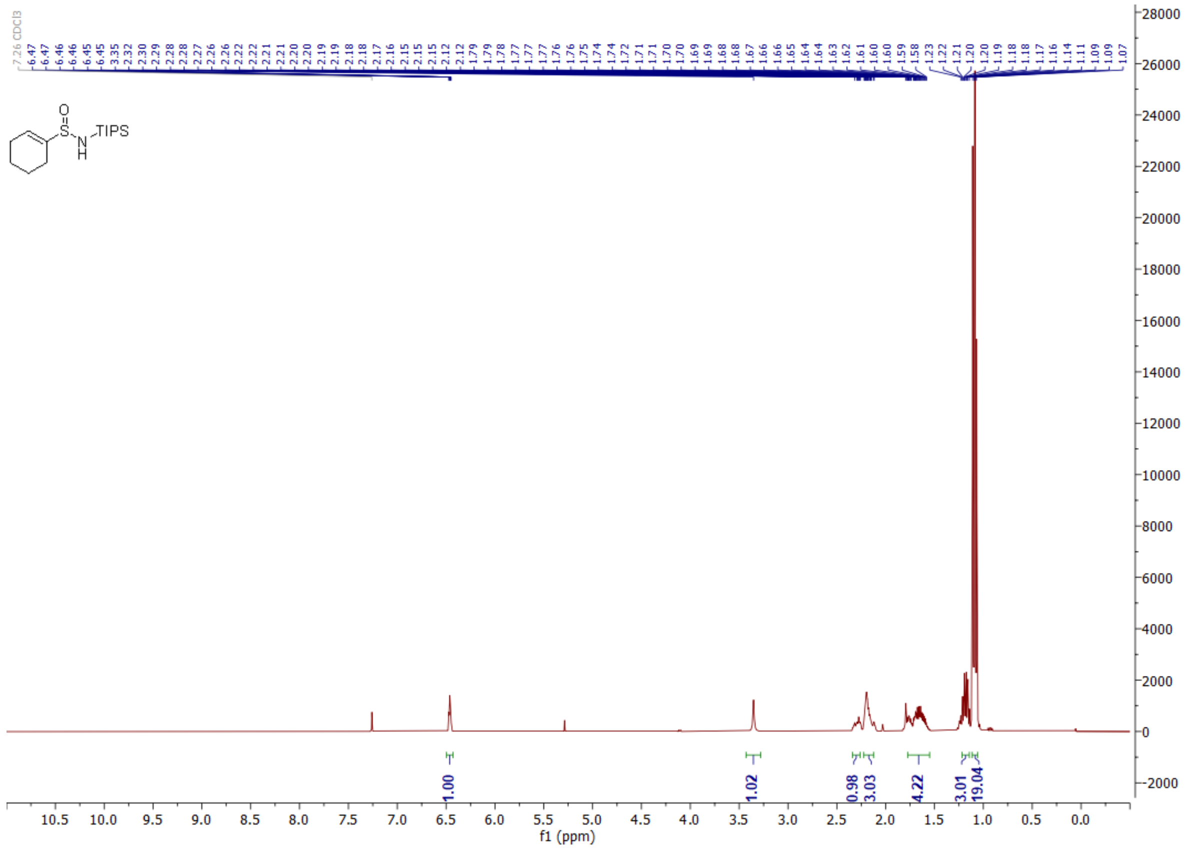

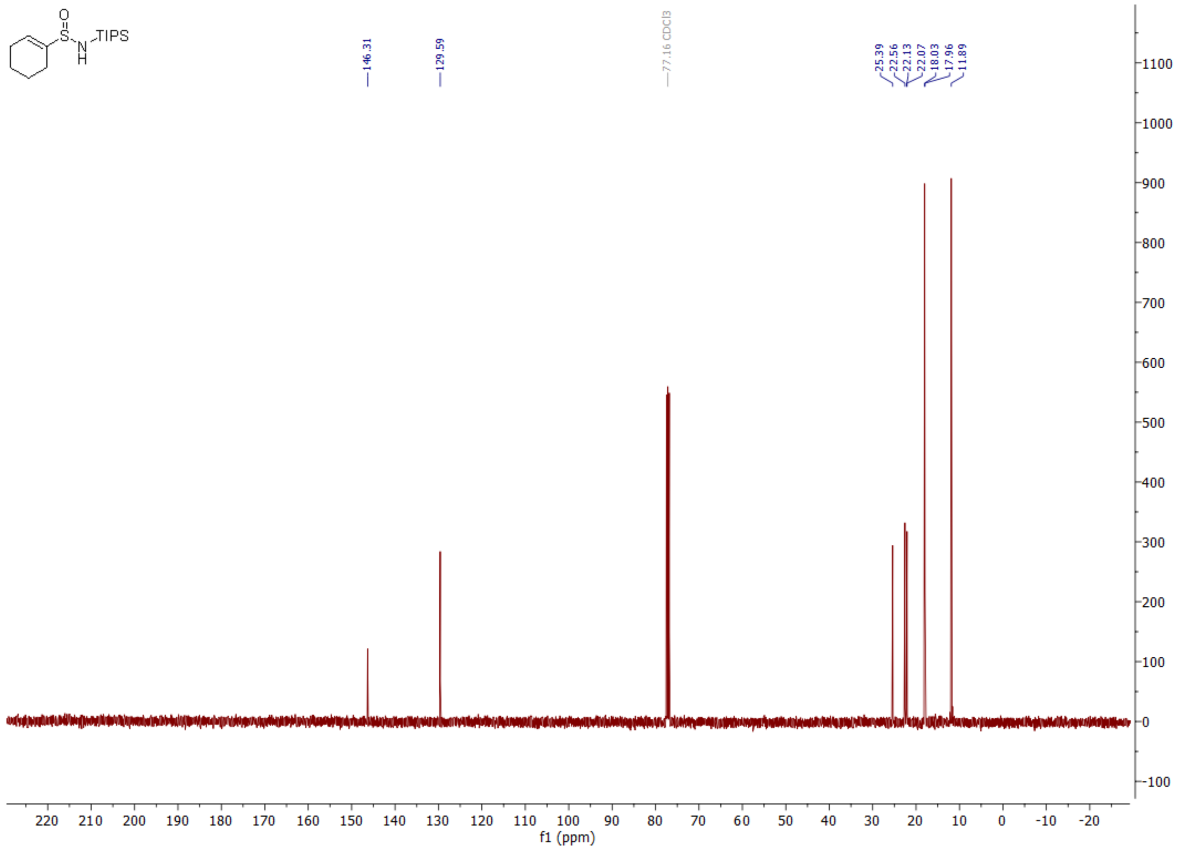

4,4-Difluoro-*N*-(triisopropylsilyl)cyclohex-1-ene-1-sulfinamide (3f)

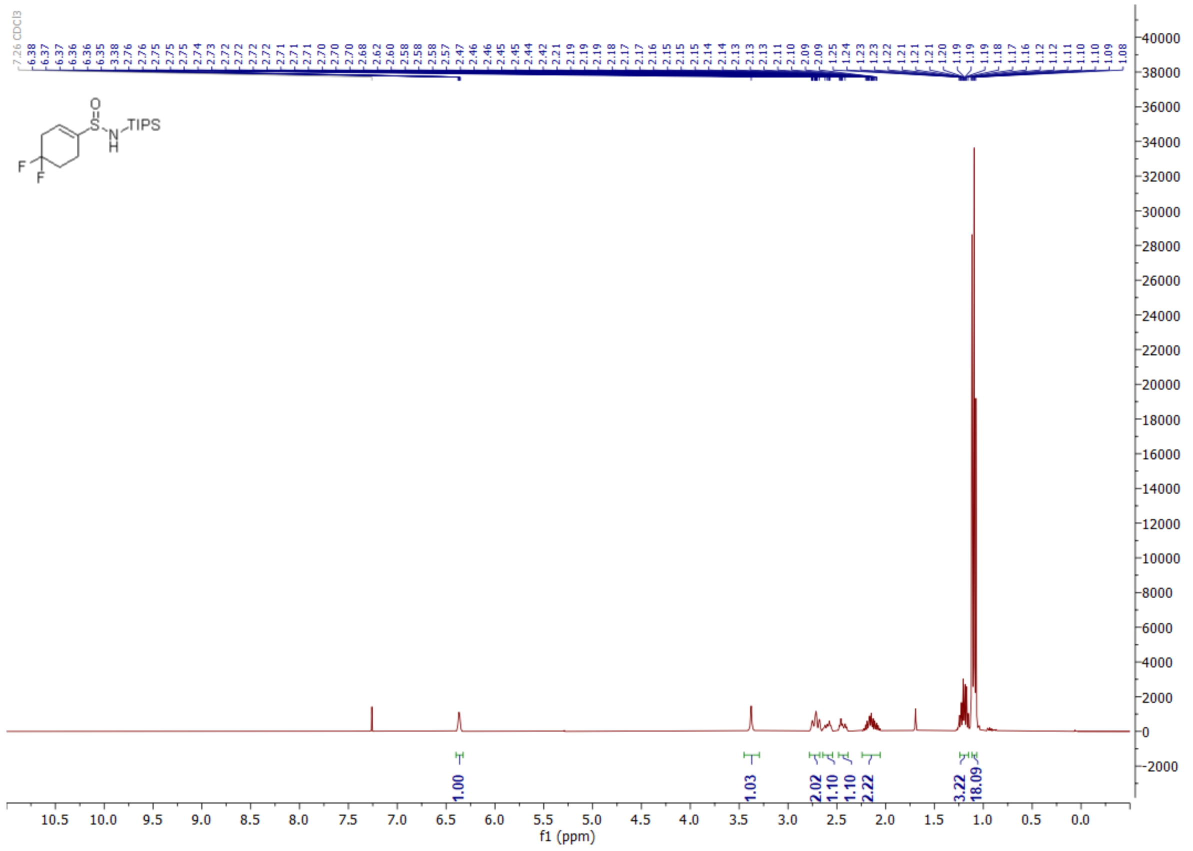

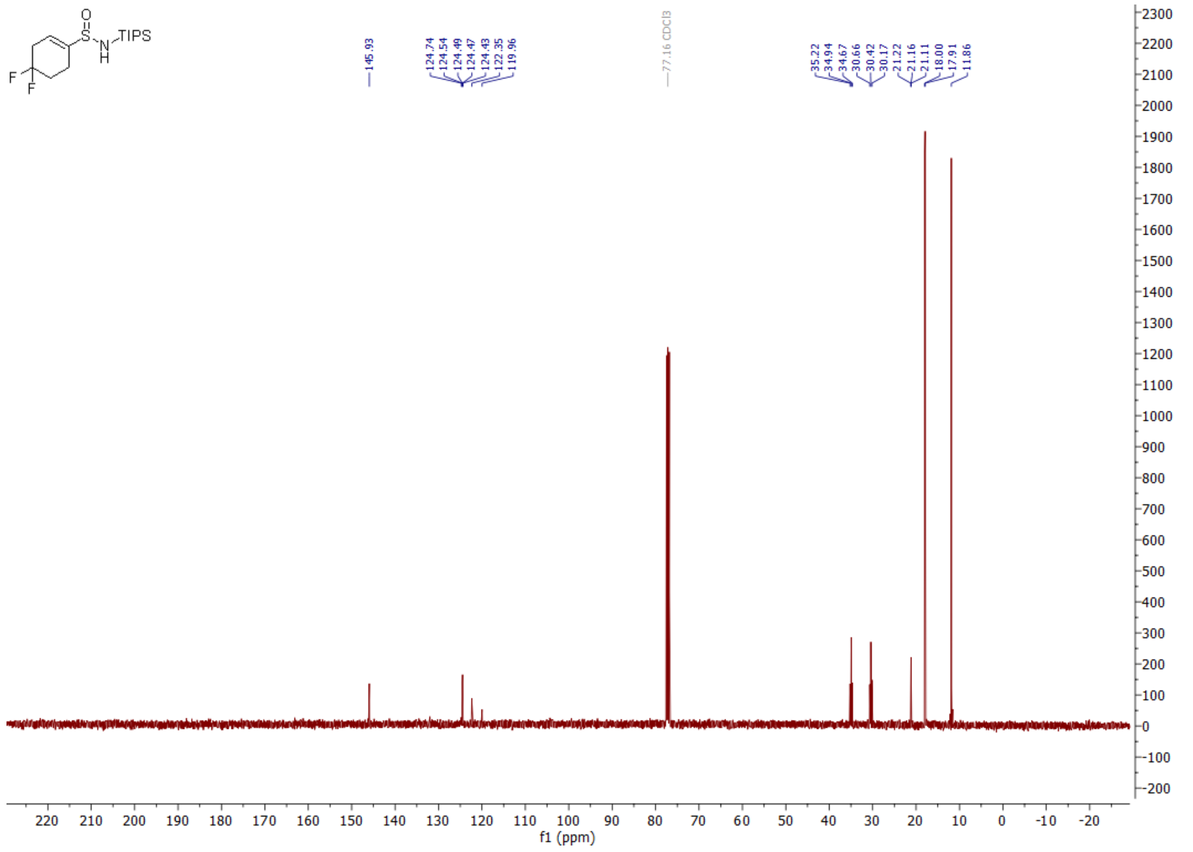

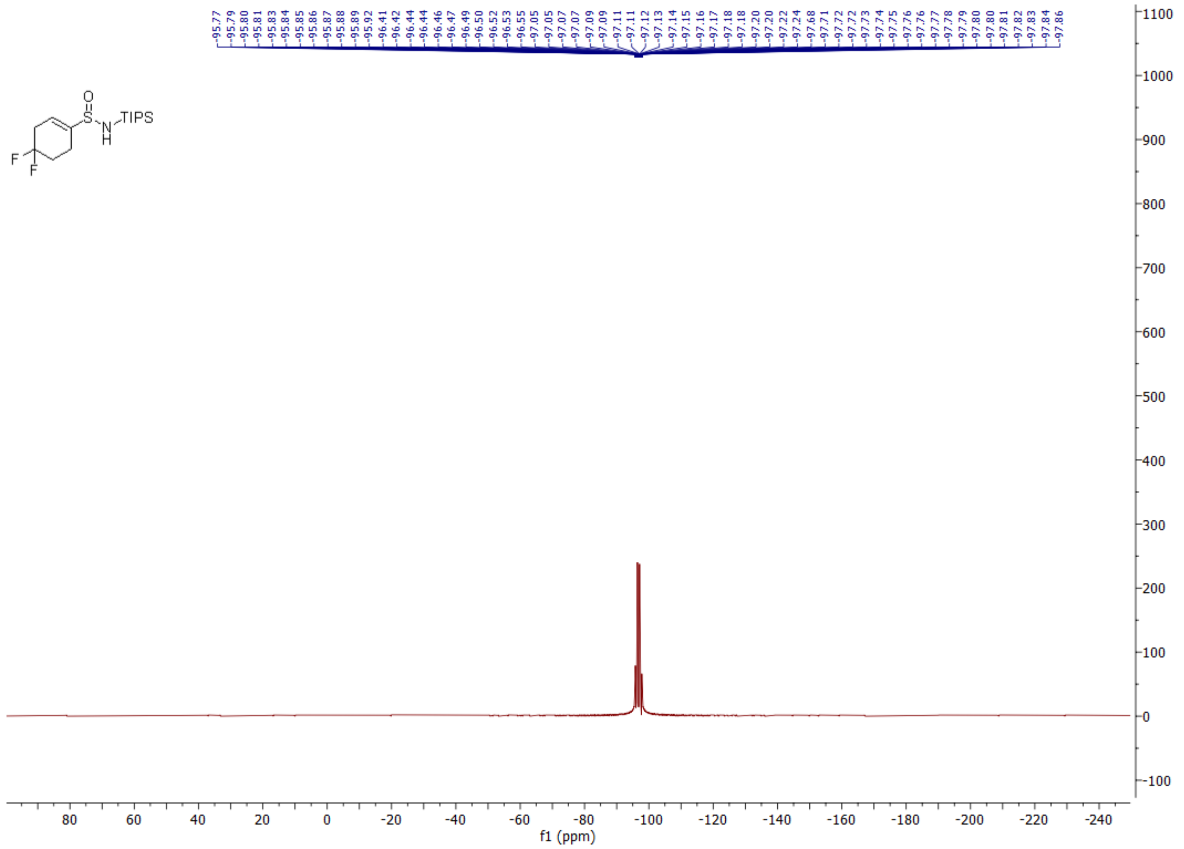

4-(Tert-butyl)-*N*-(triisopropylsilyl)cyclohex-1-ene-1-sulfinamide (3g)

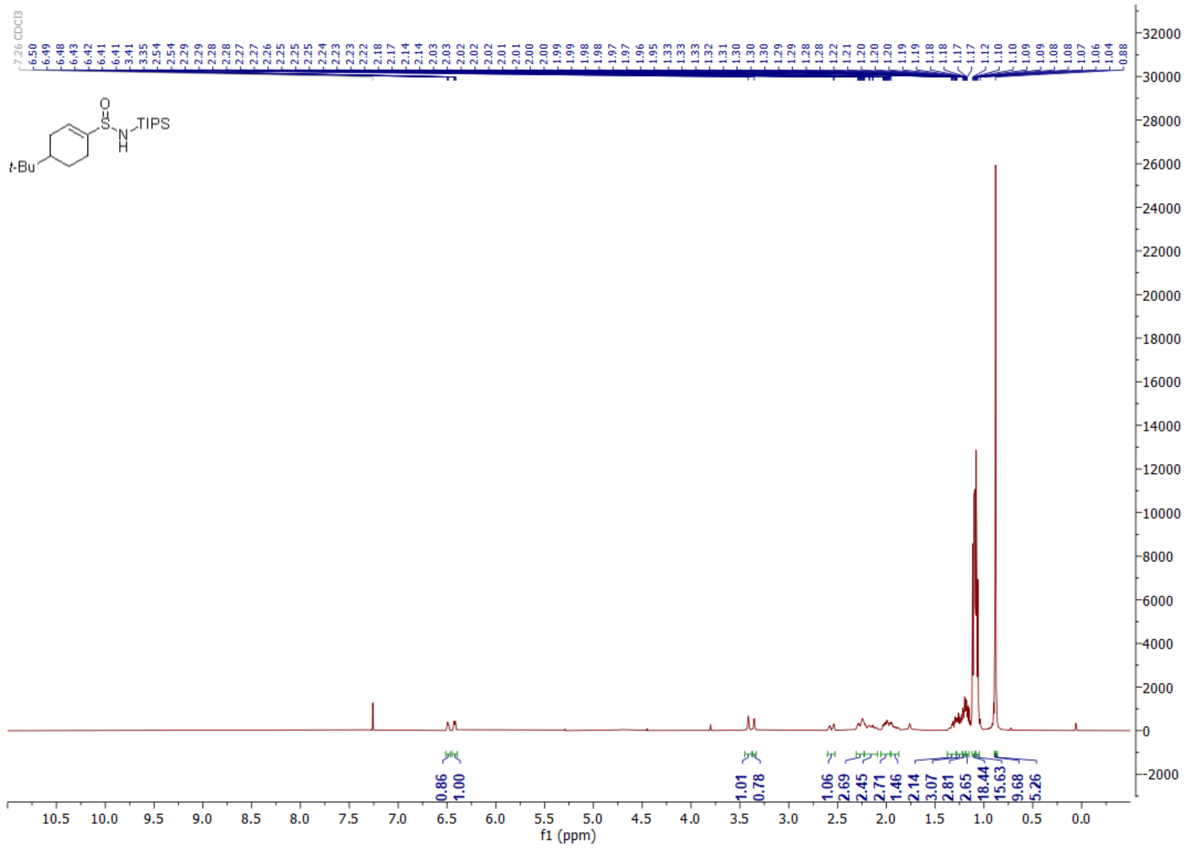

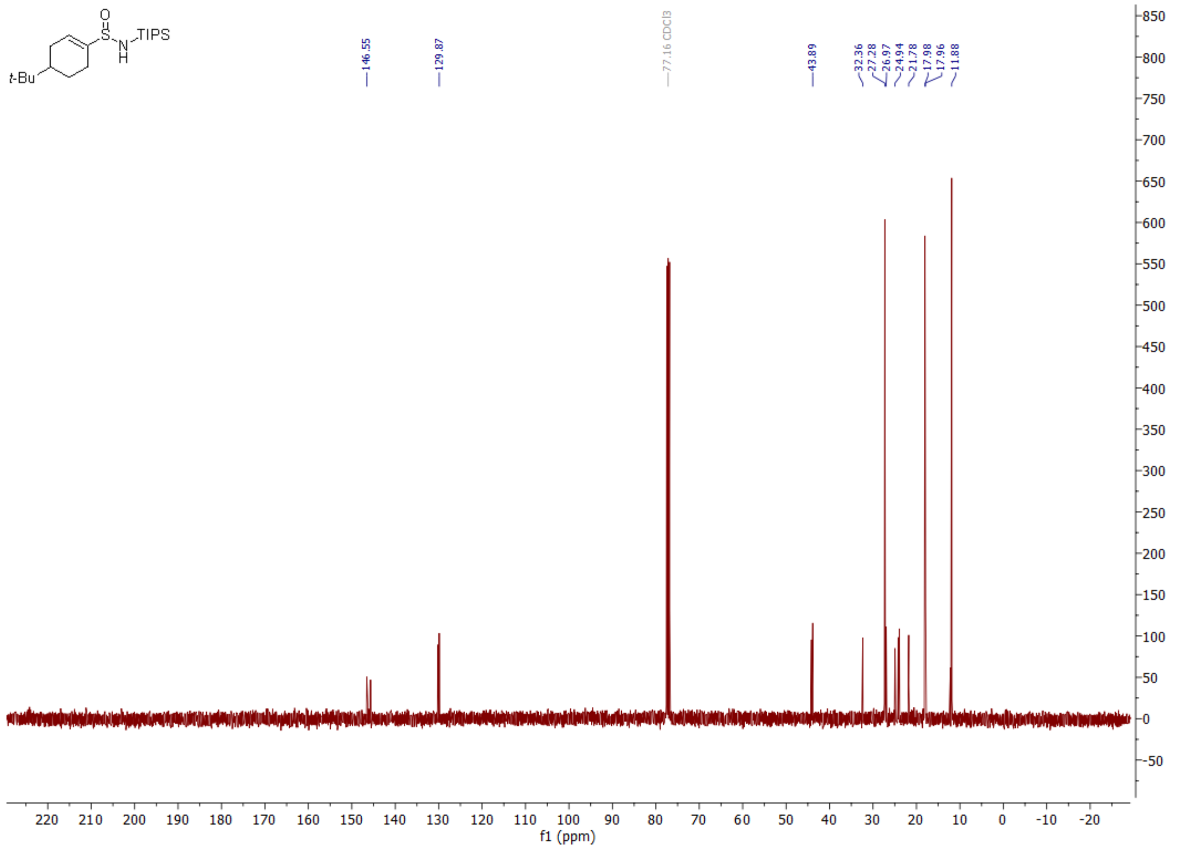

N-(triisopropylsilyl)-3,4-dihydronaphthalene-1-sulfinamide (3h)

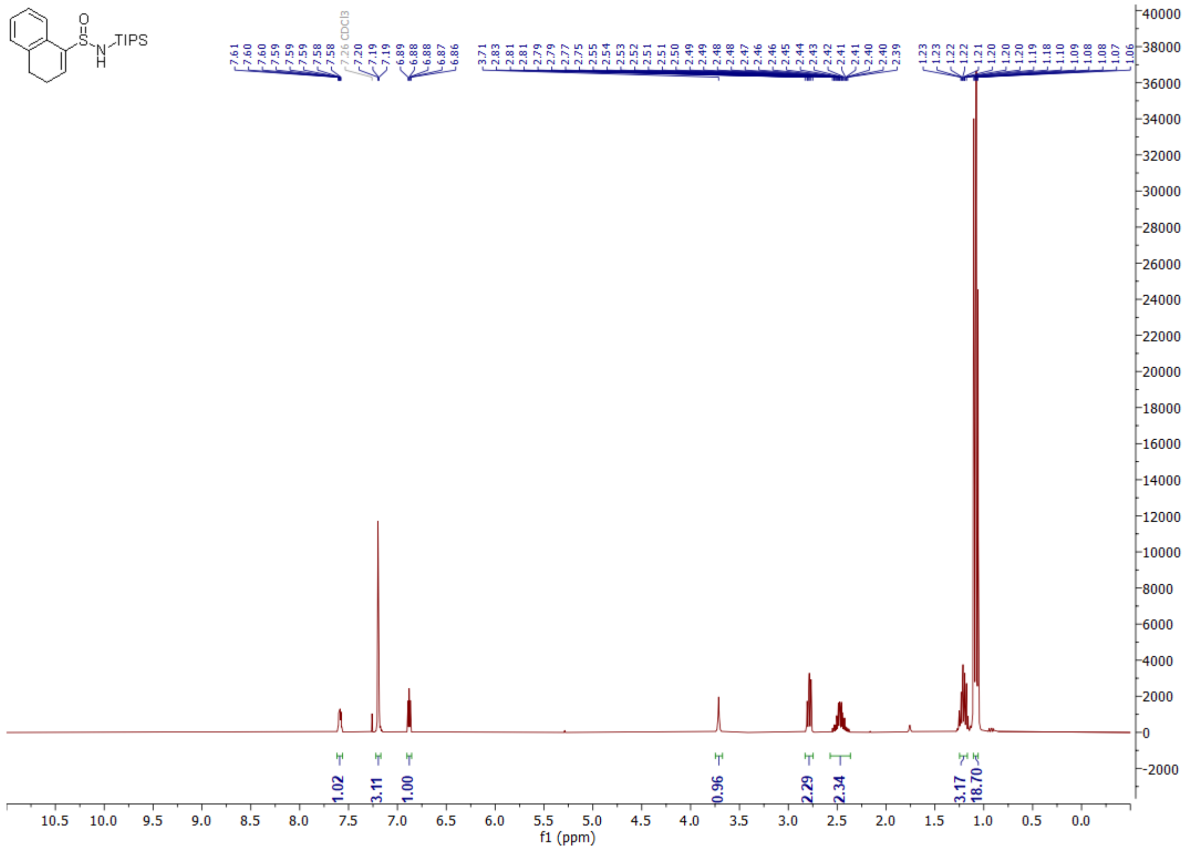

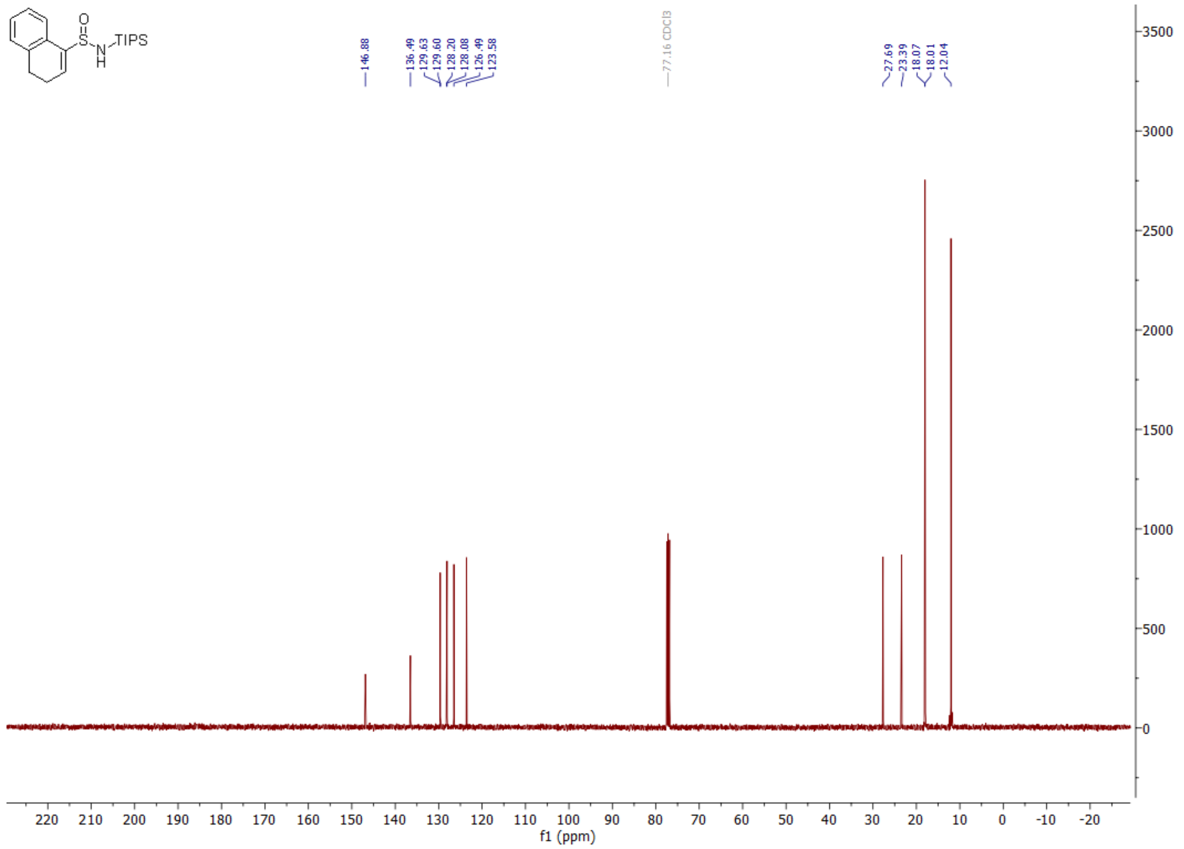

***N*-(triisopropylsilyl)bicyclo[2.2.1]hept-2-ene-2-sulfinamide (3i)**

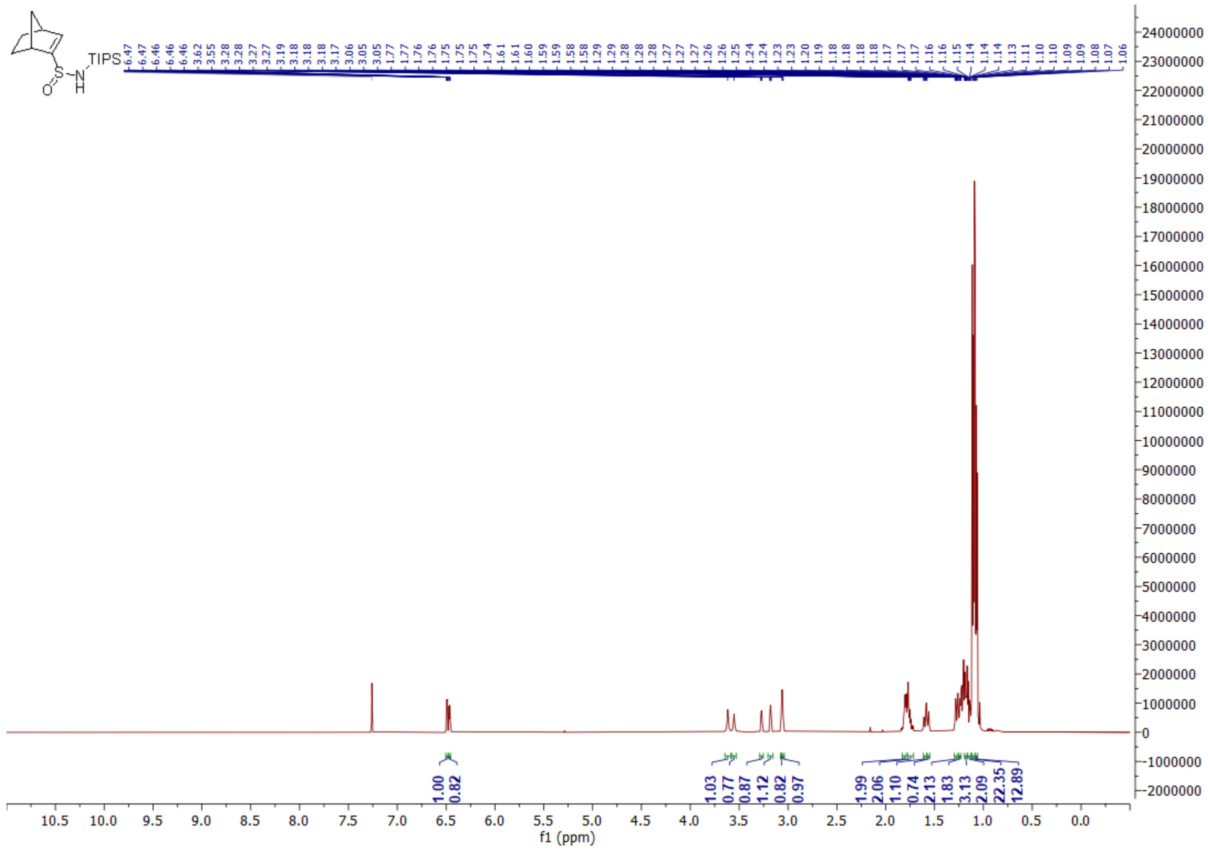

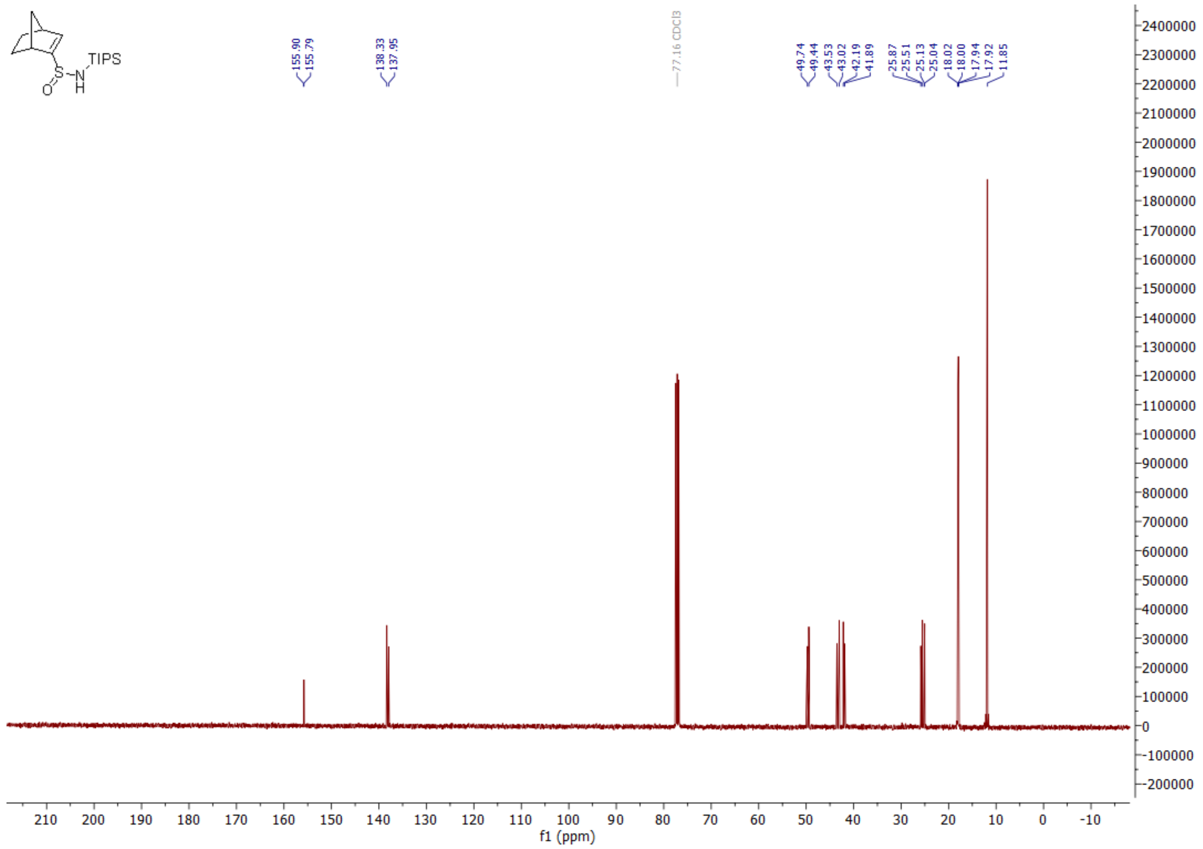

(1S,4R)-1,7,7-trimethyl-*N*-(triisopropylsilyl)bicyclo[2.2.1]hept-2-ene-2-sulfinamide (3j)

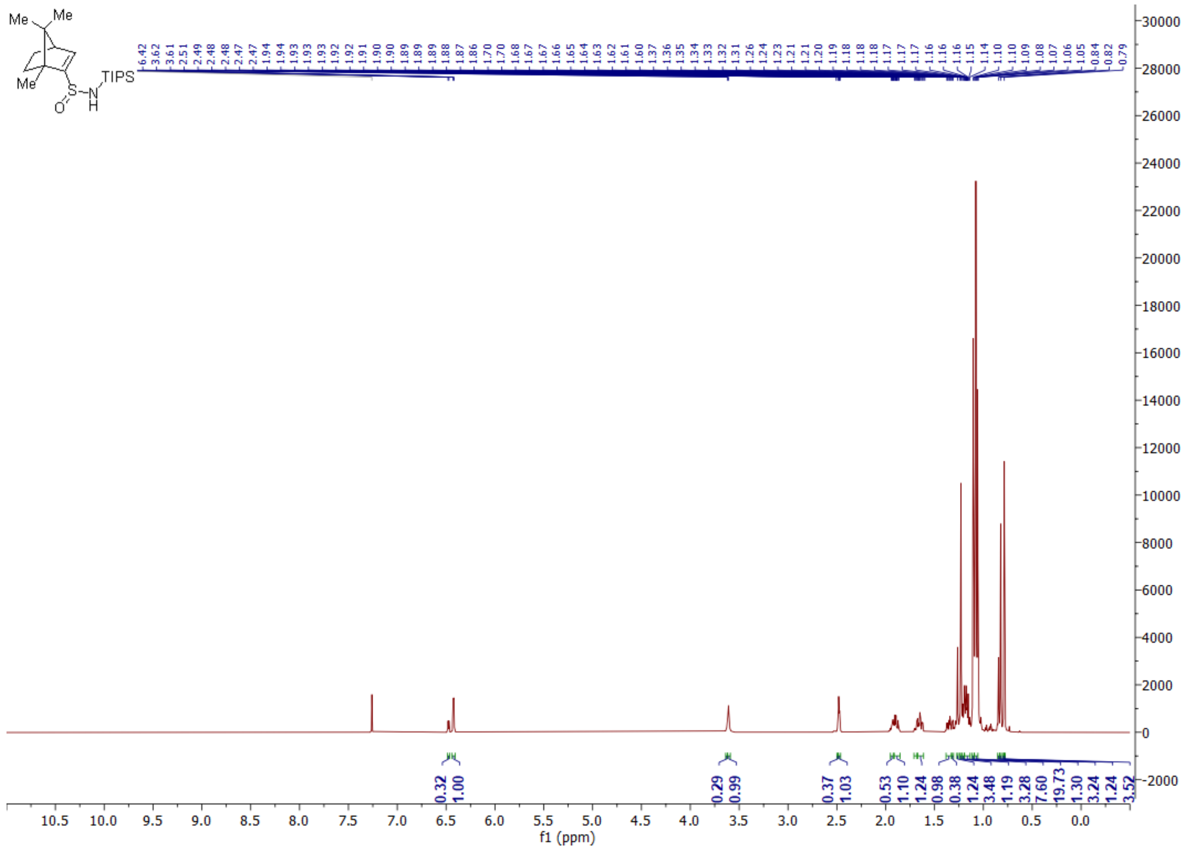

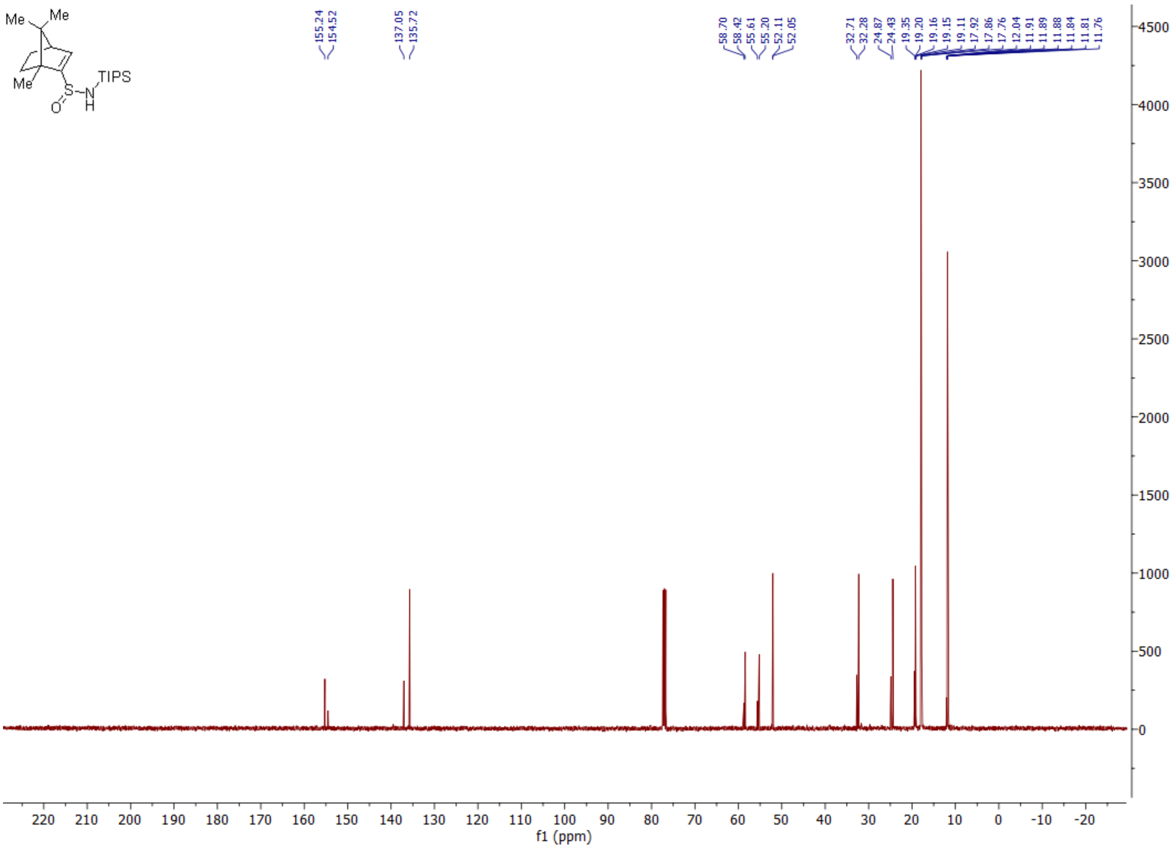

(3*S*,8*R*,9*S*,10*R*,13*S*,14*S*)-10,13-Dimethyl-17-(((triisopropylsilyl)amino)sulfinyl)-2,3,4,7,8,9,10,11,12,13,14,15-dodecahydro-1H-cyclopenta[a]phenanthren-3-yl cetate(3k)

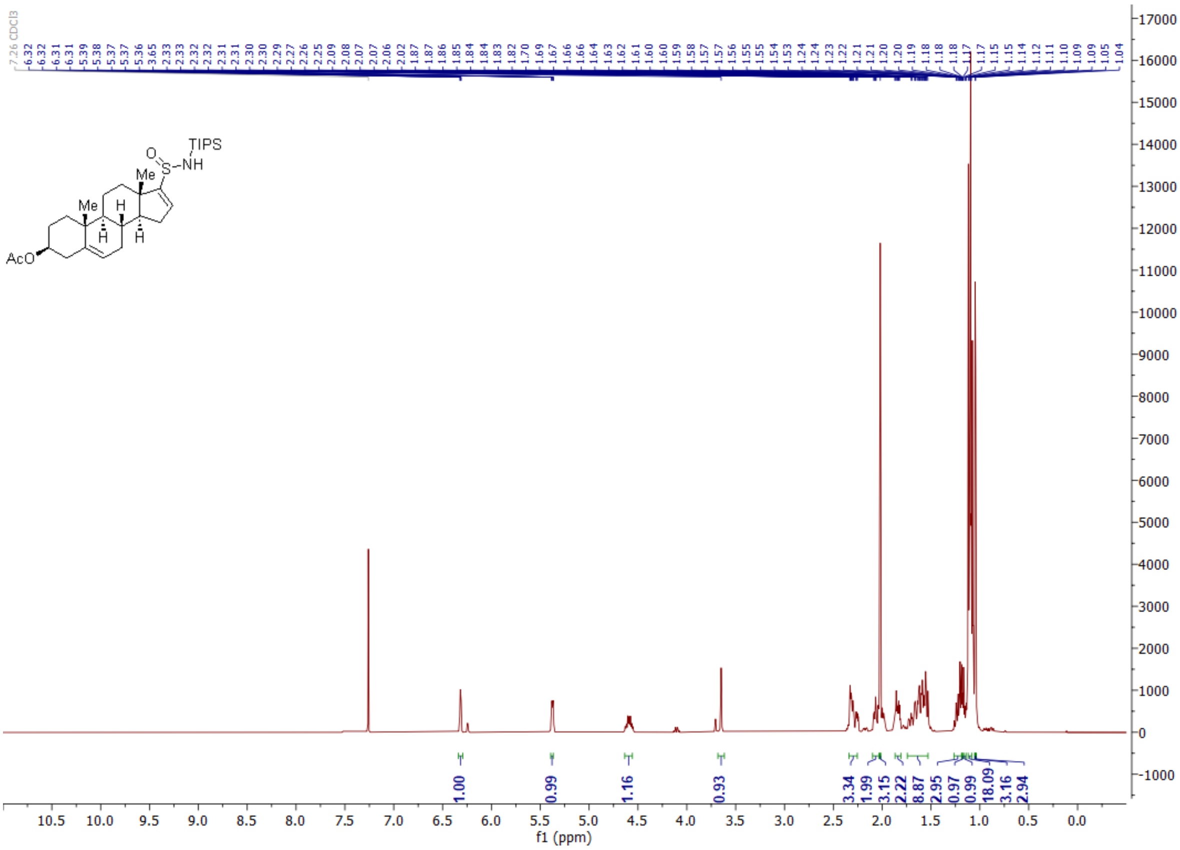

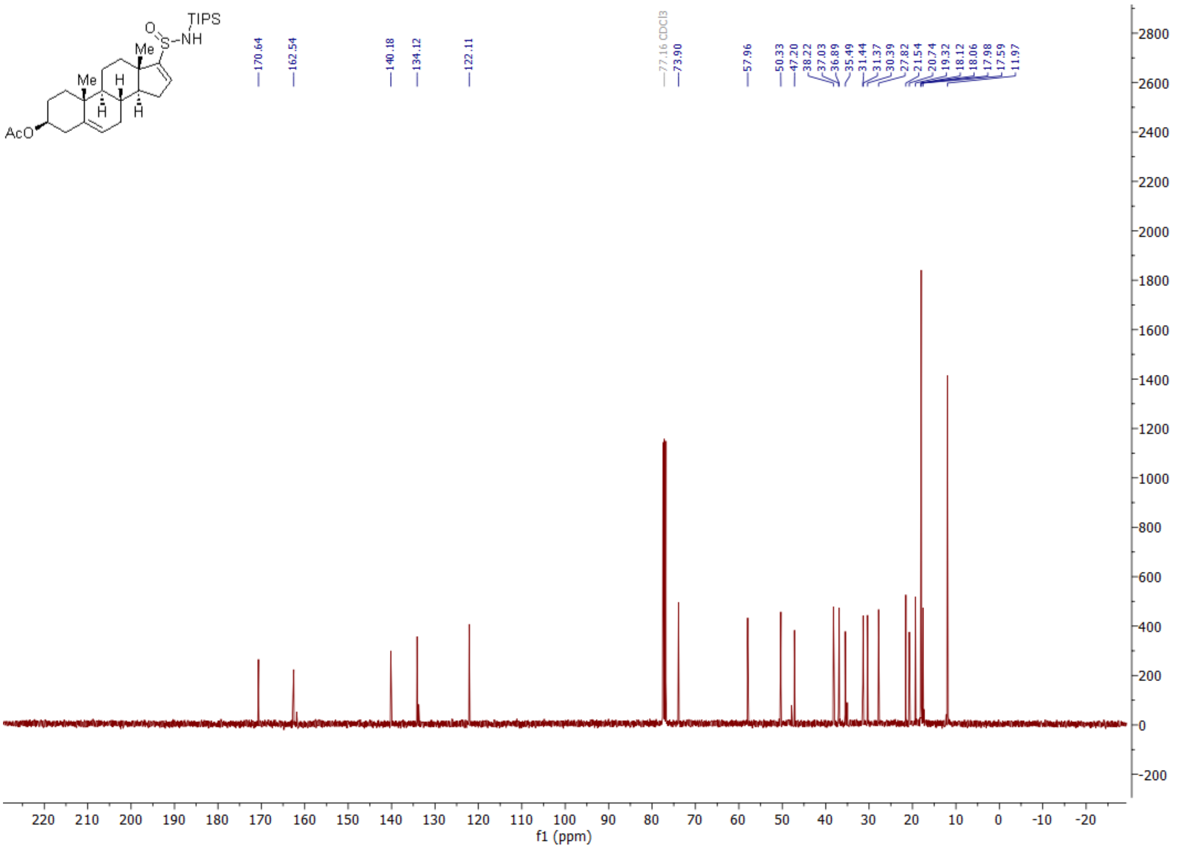

Methyl 3-(aminosulfinyl)benzoate (4a)

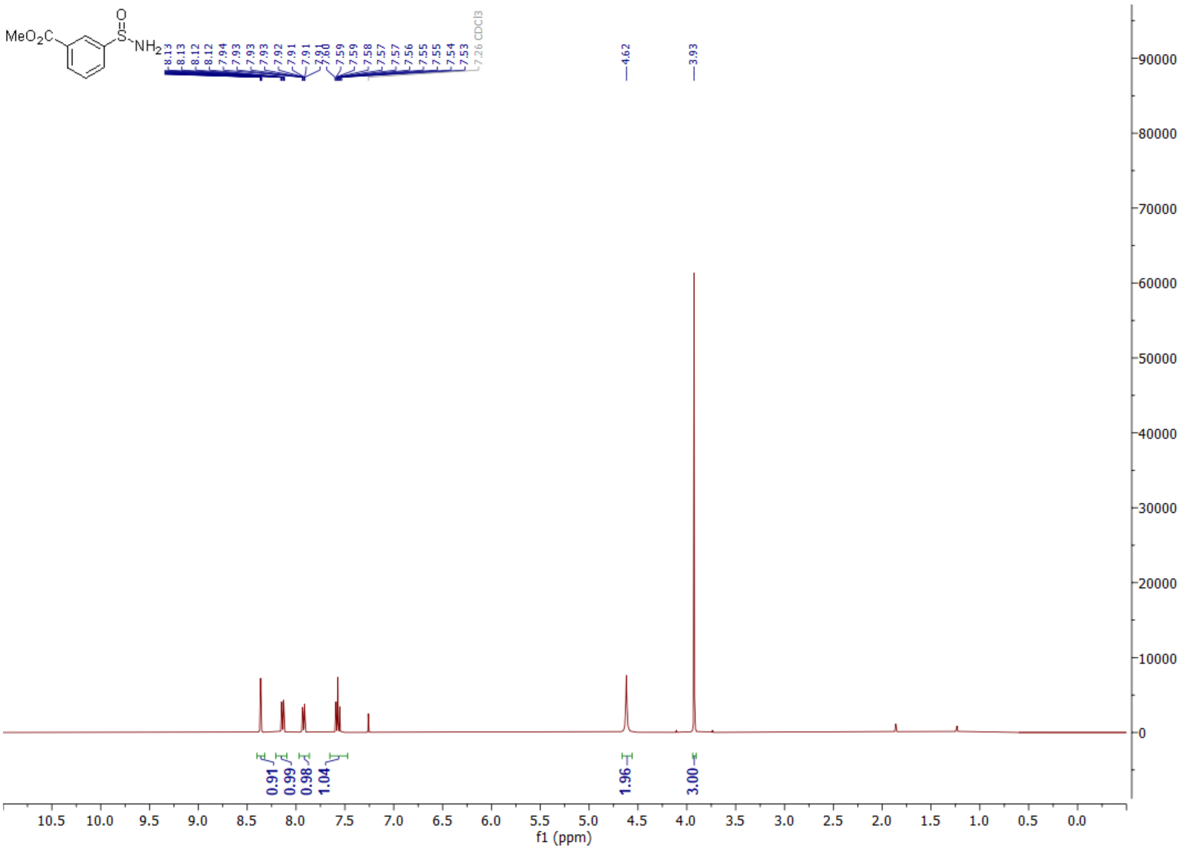

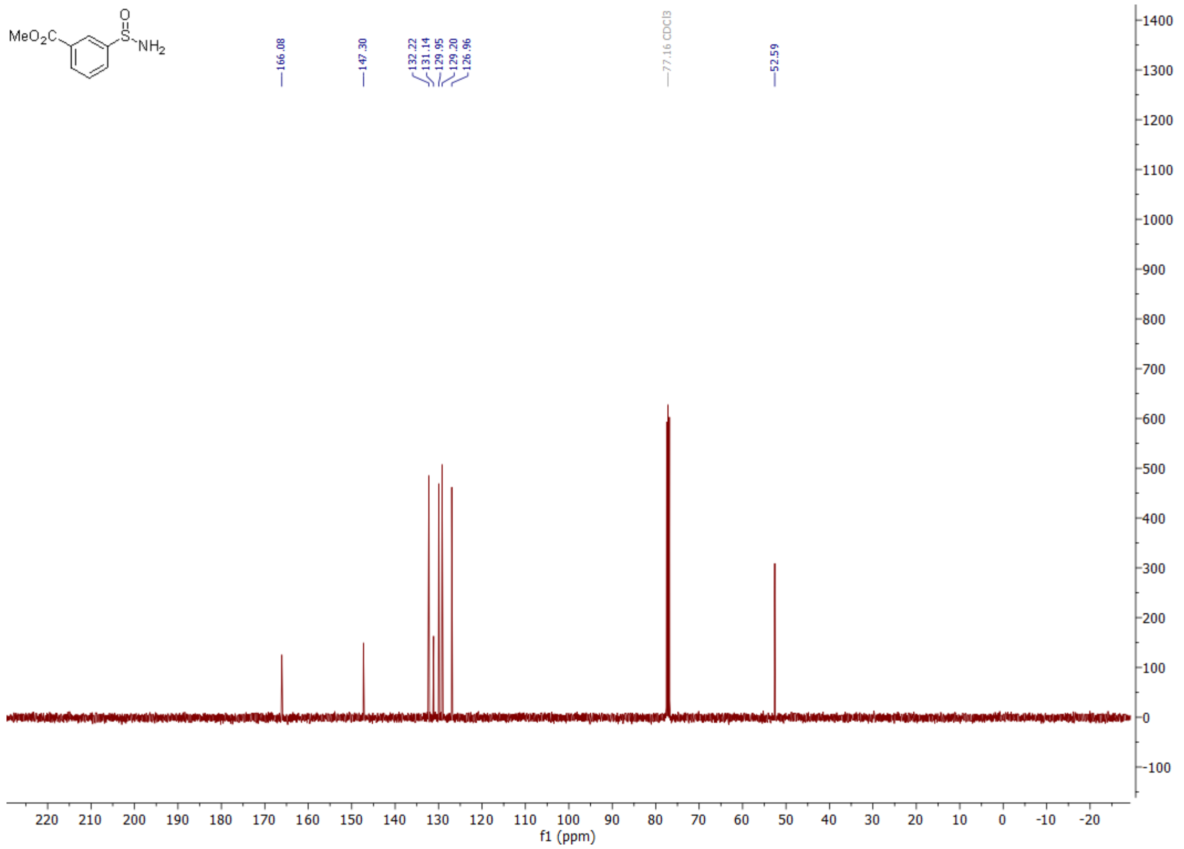

Methyl 3-(morpholine-4-sulfonimidoyl)benzoate (4b)

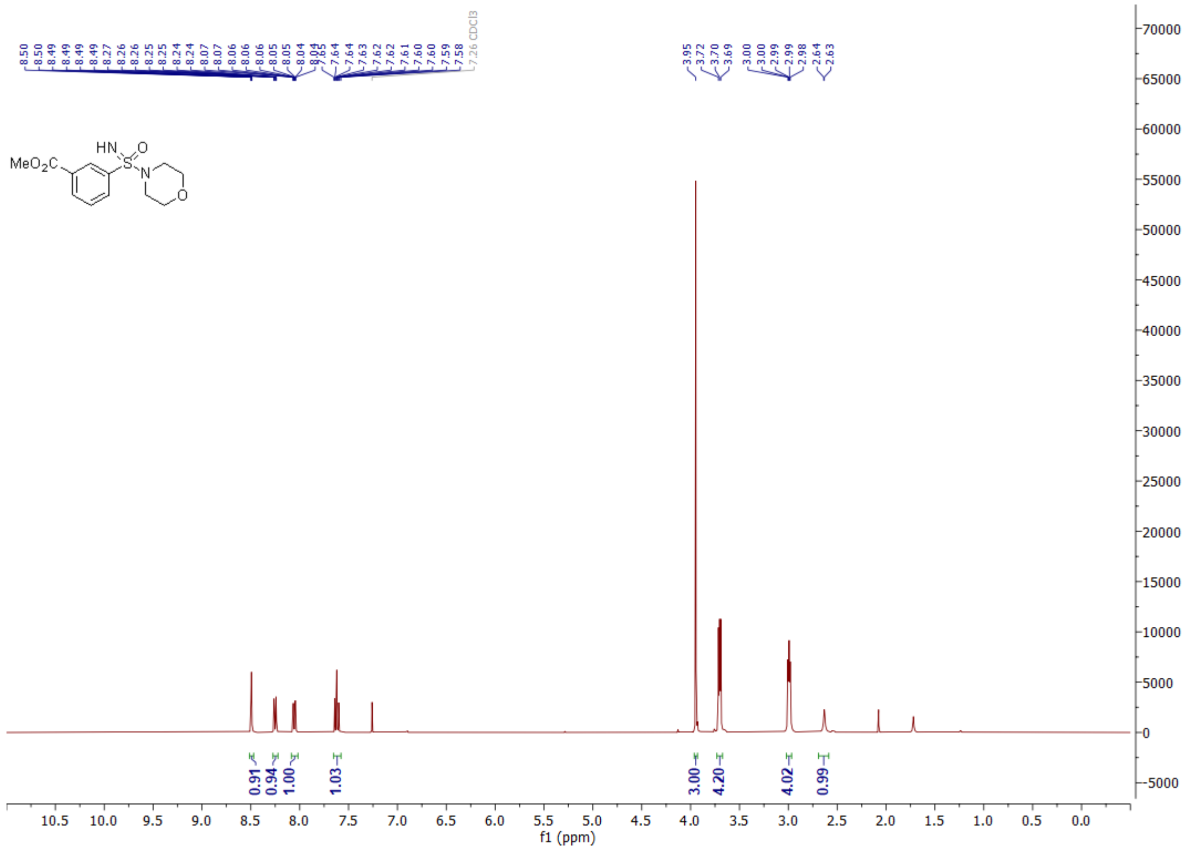

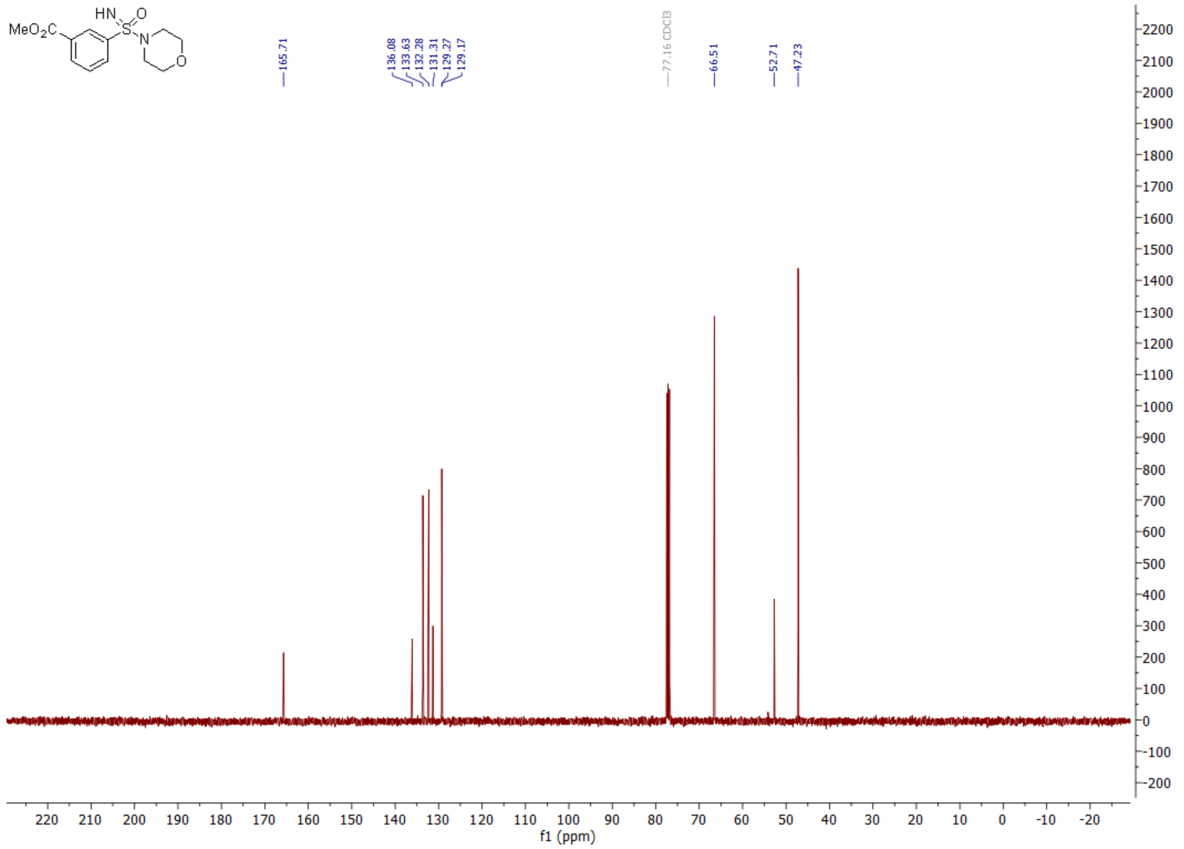

Methyl 3-(*N'*-(triisopropylsilyl)sulfamidimidoyl)benzoate (4c’)

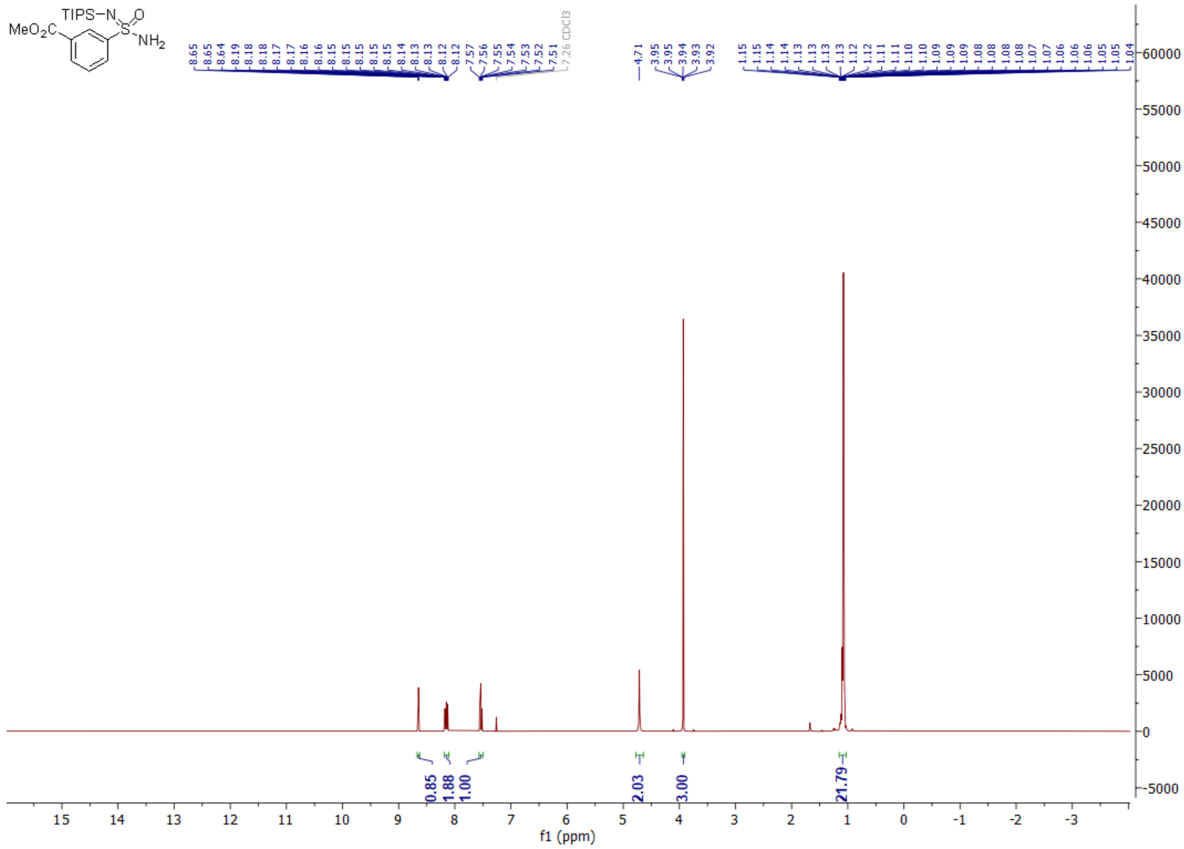

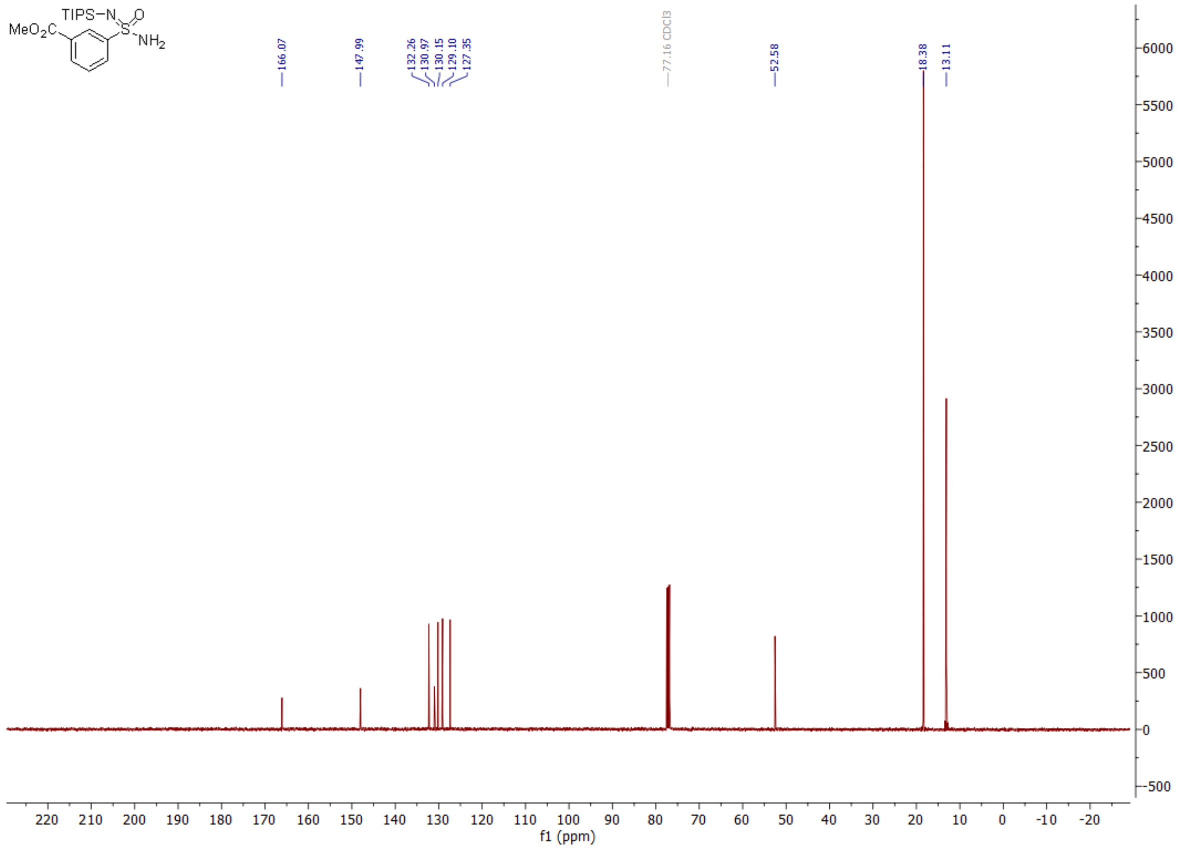

Methyl 3-sulfamidimidoylbenzoate (4c)

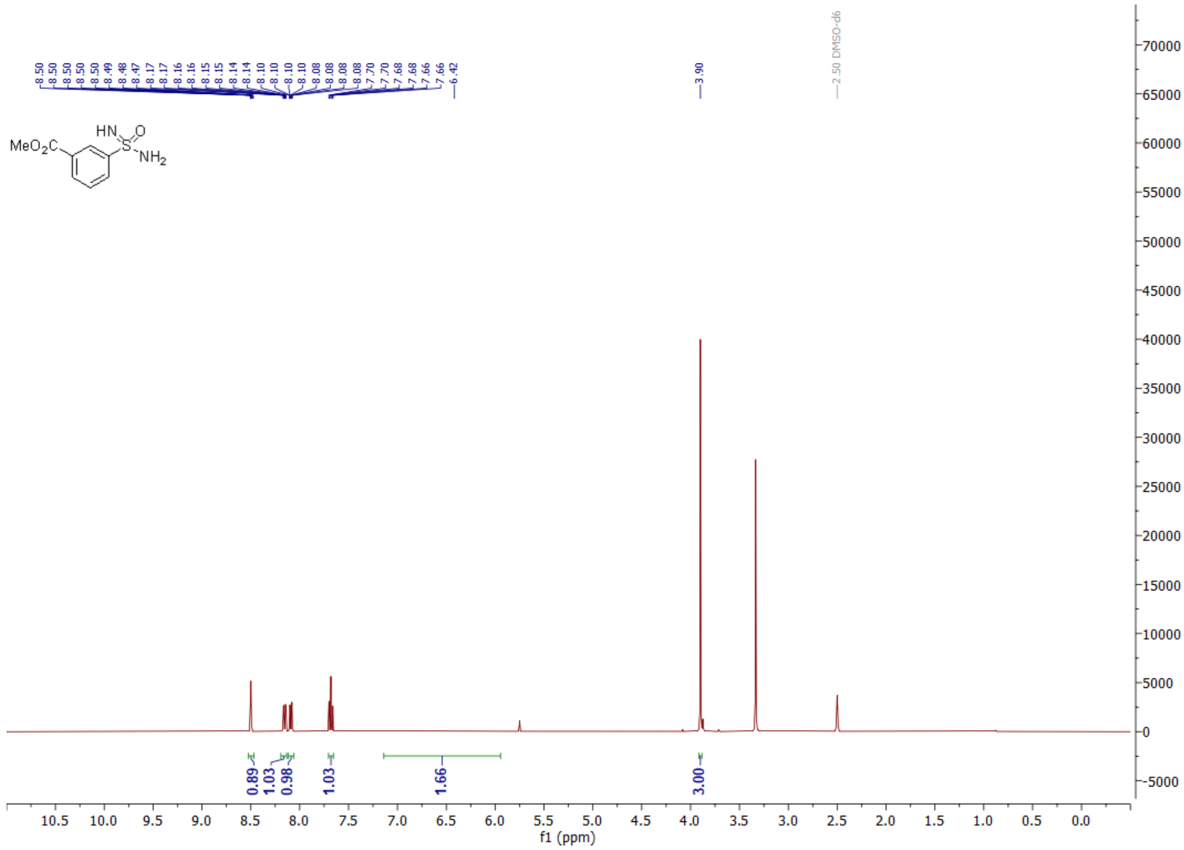

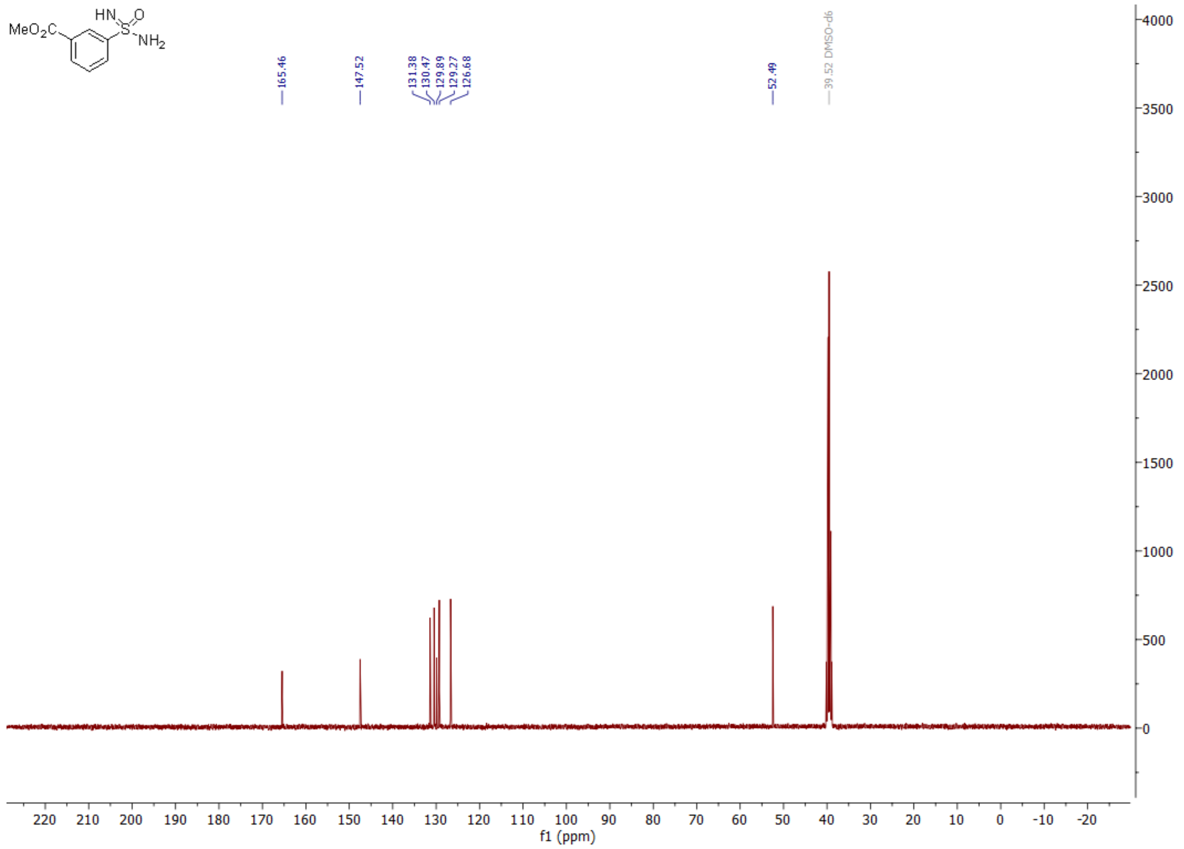

Methyl 3-(*N*-(p-tolyl)-*N*'-(triisopropylsilyl)sulfamidimidoyl)benzoate (4d')

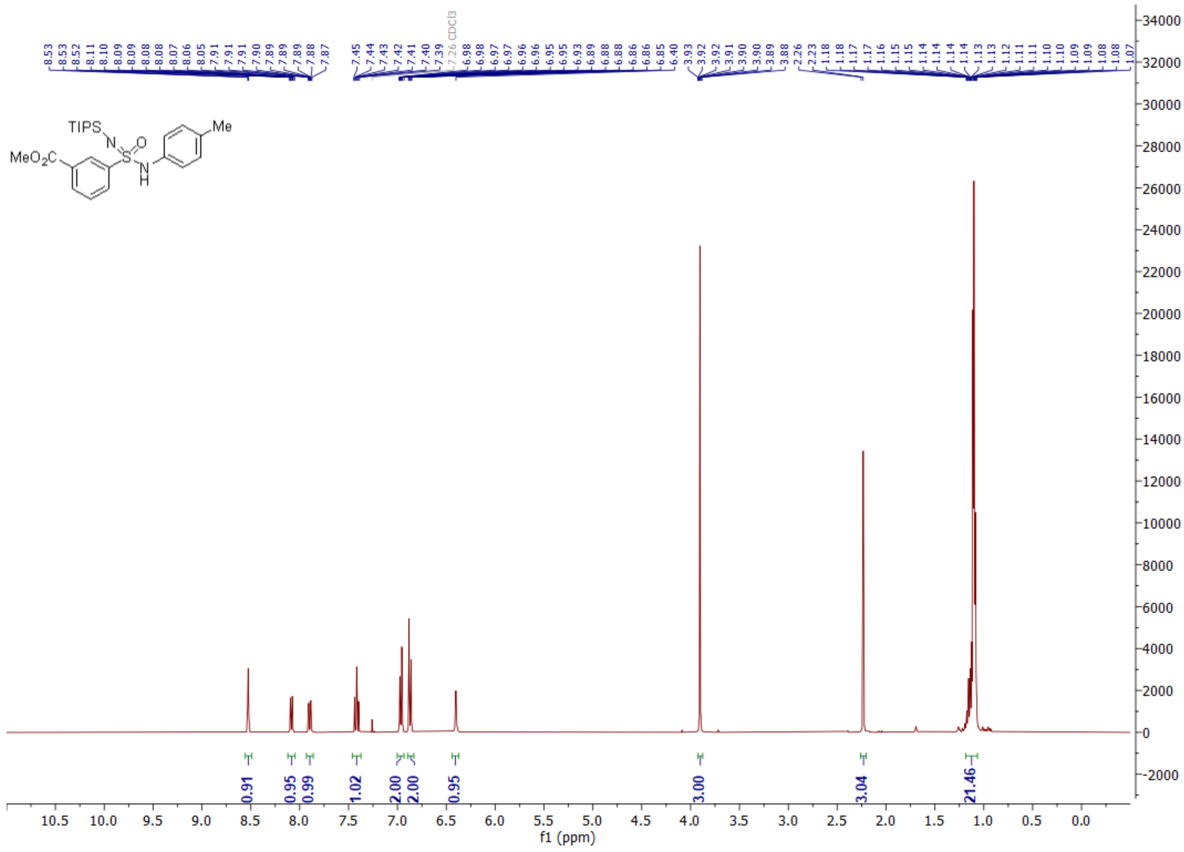

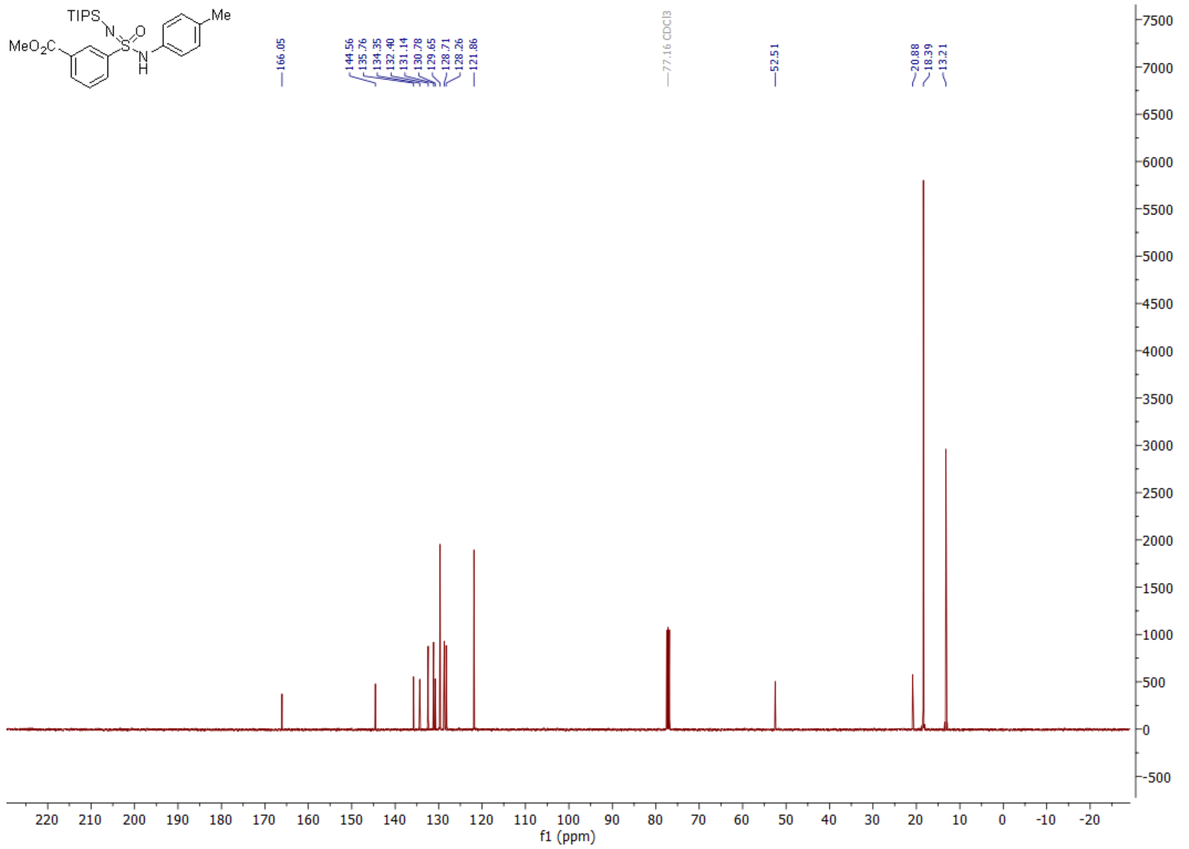

Methyl 3-(N-(p-tolyl)sulfamidimidoyl)benzoate (4d)

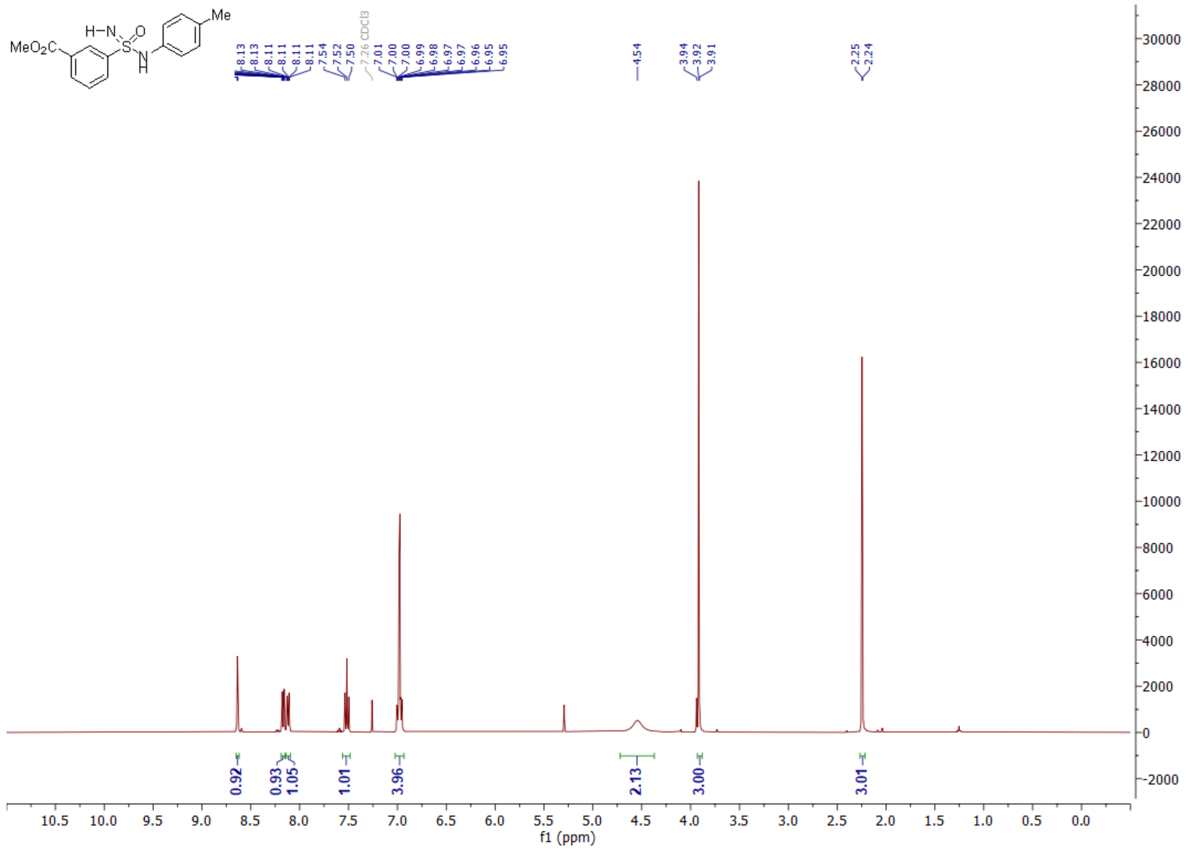

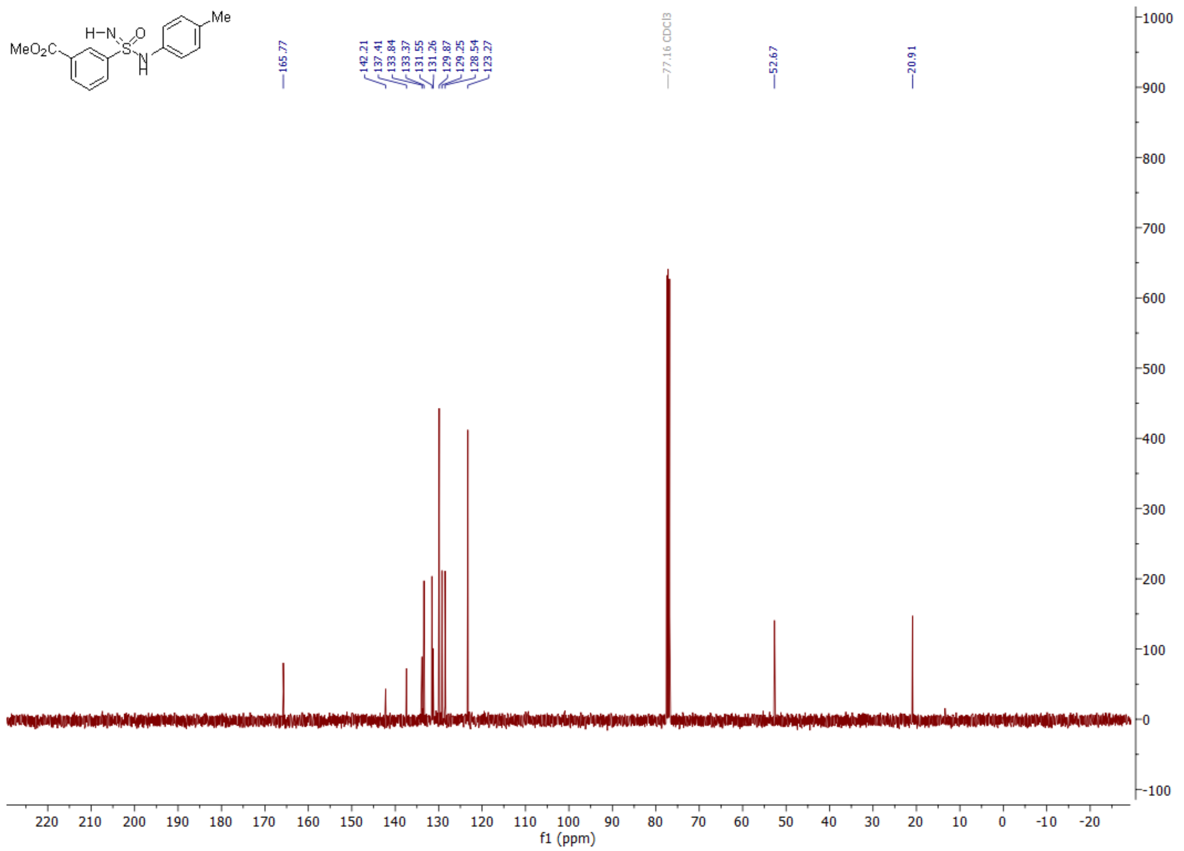

Methyl 3-(*S*-fluoro-*N*-(triisopropylsilyl)sulfonimidoyl)benzoate (4e)

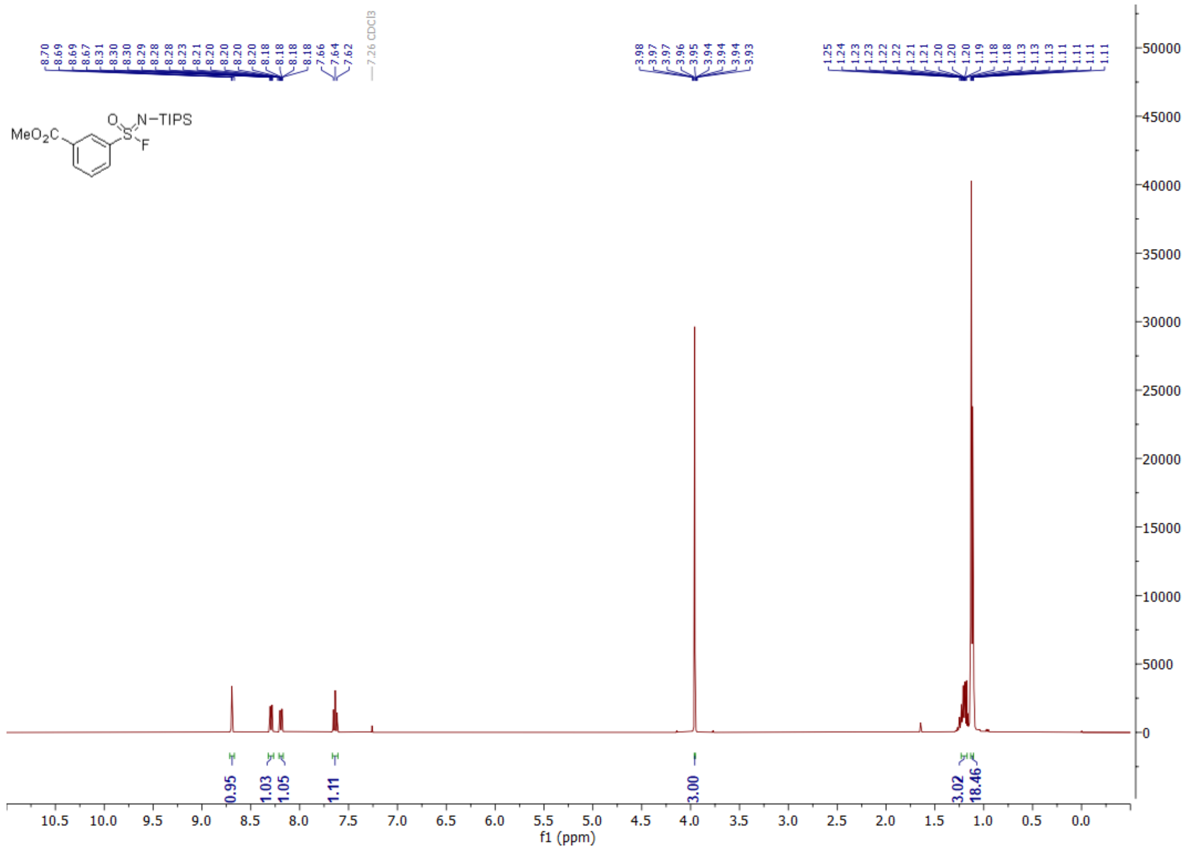

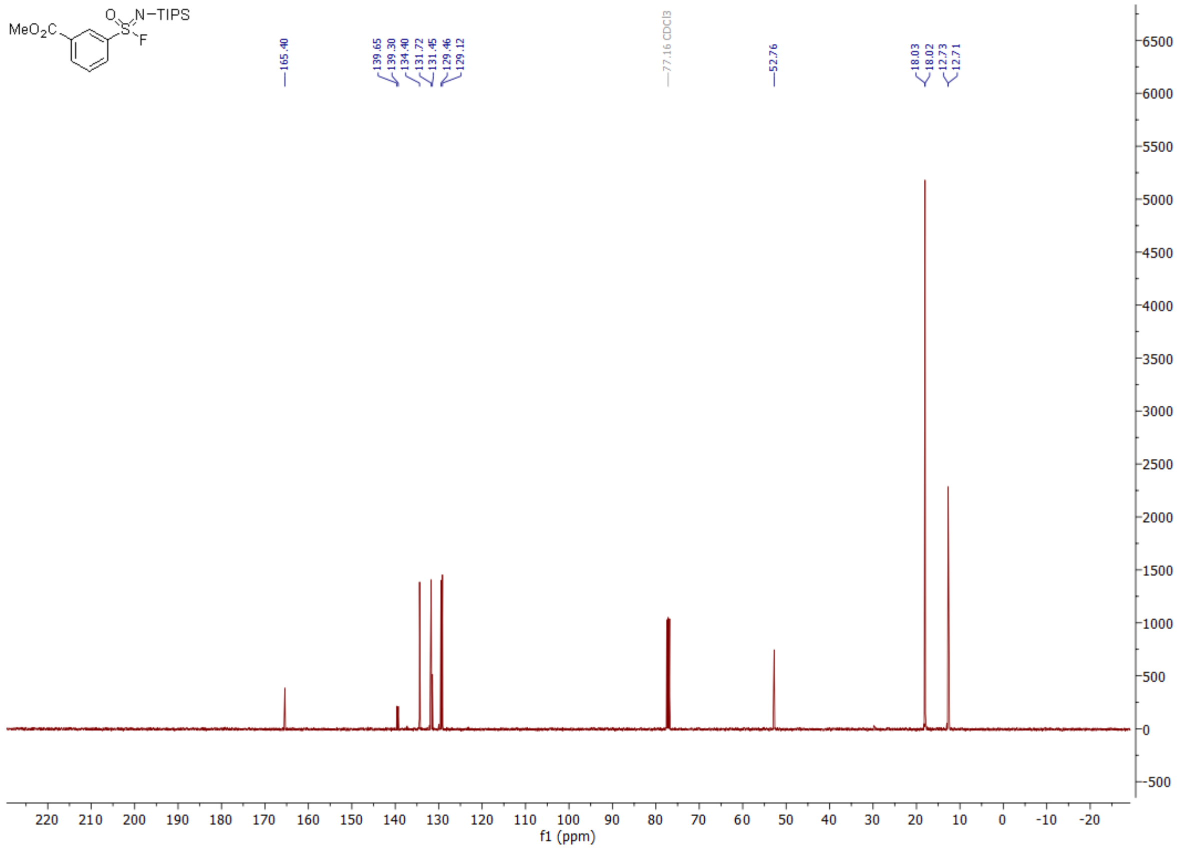
MeO2C
O
N−TIPS
S
F
165.40
139.65
139.30
134.40
131.72
131.45
129.46
129.12
77.16 CDCl3
52.76
18.03
18.02
12.73
12.71
f1 (ppm)

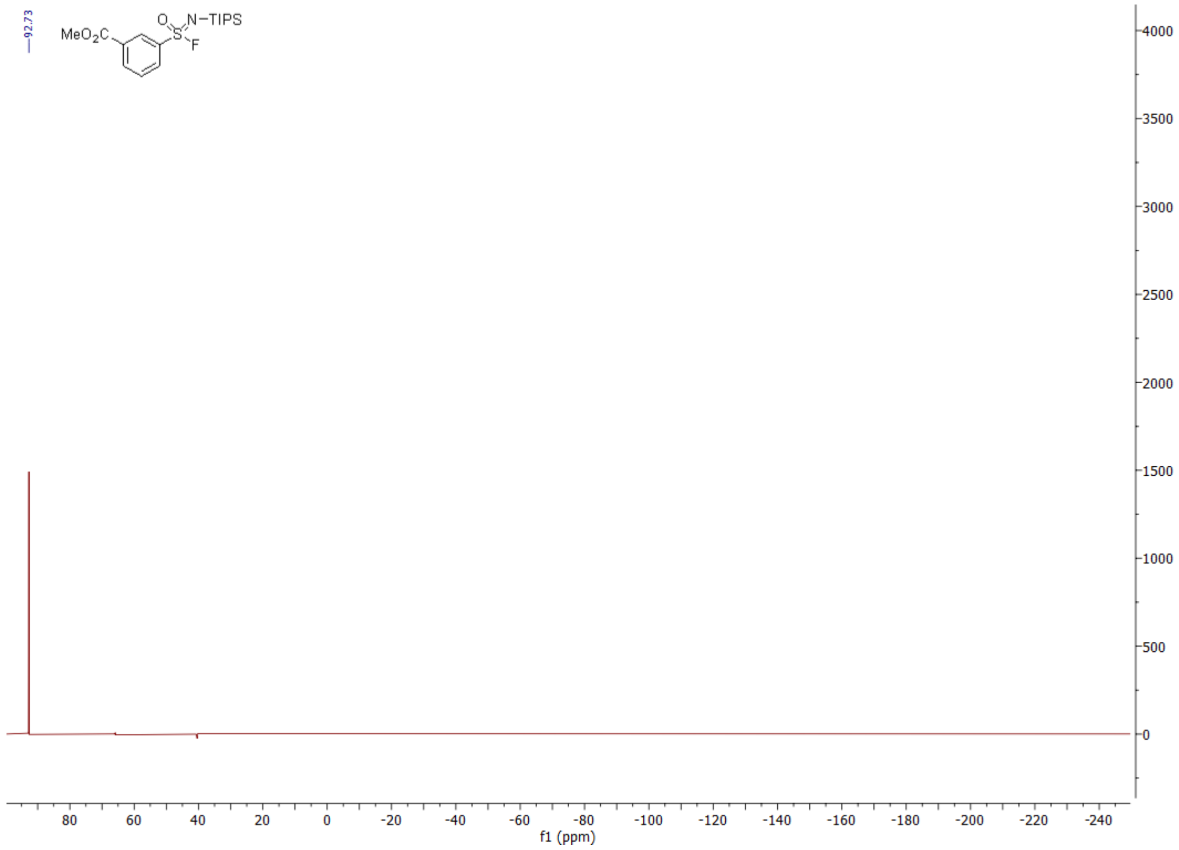
92.73
MeO2C
O
N−TIPS
S
F
f1 (ppm)

Methyl 3-(*N*-(triisopropylsilyl)sulfamoyl)benzoate (4f’)

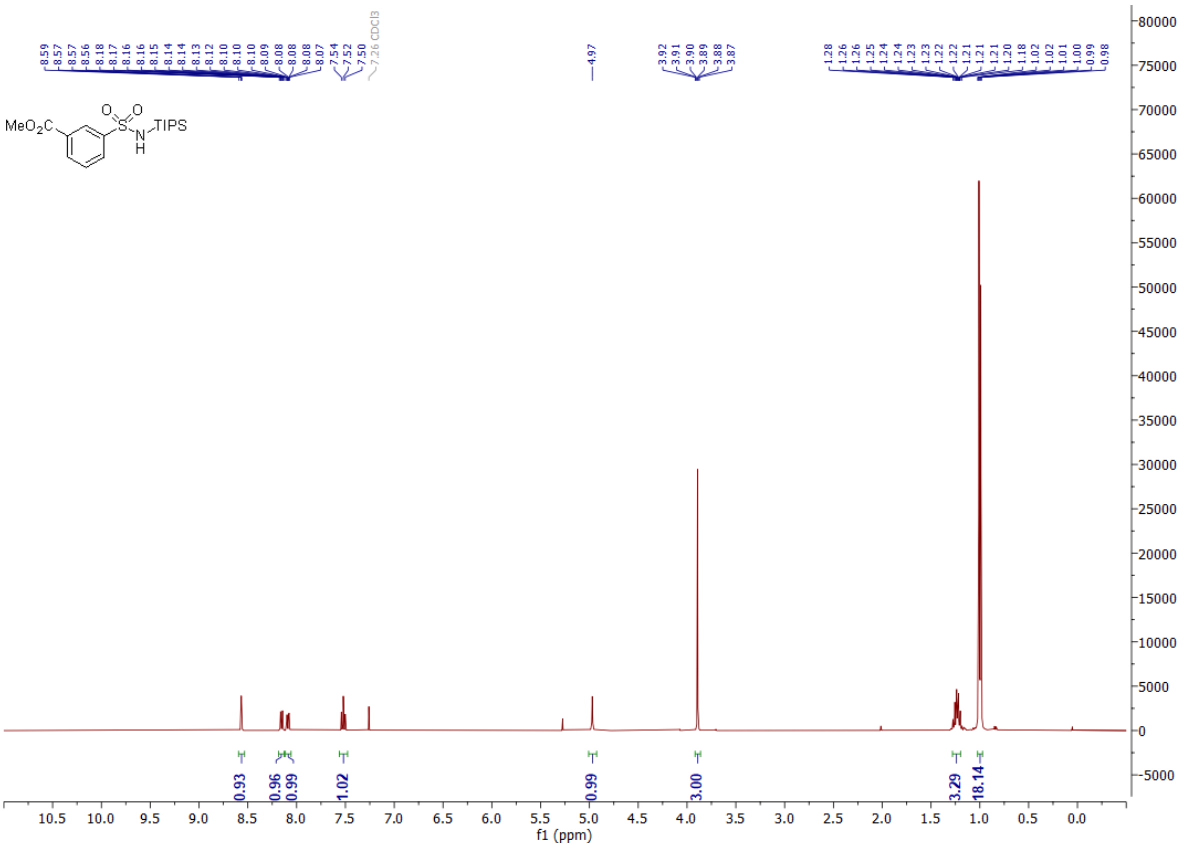

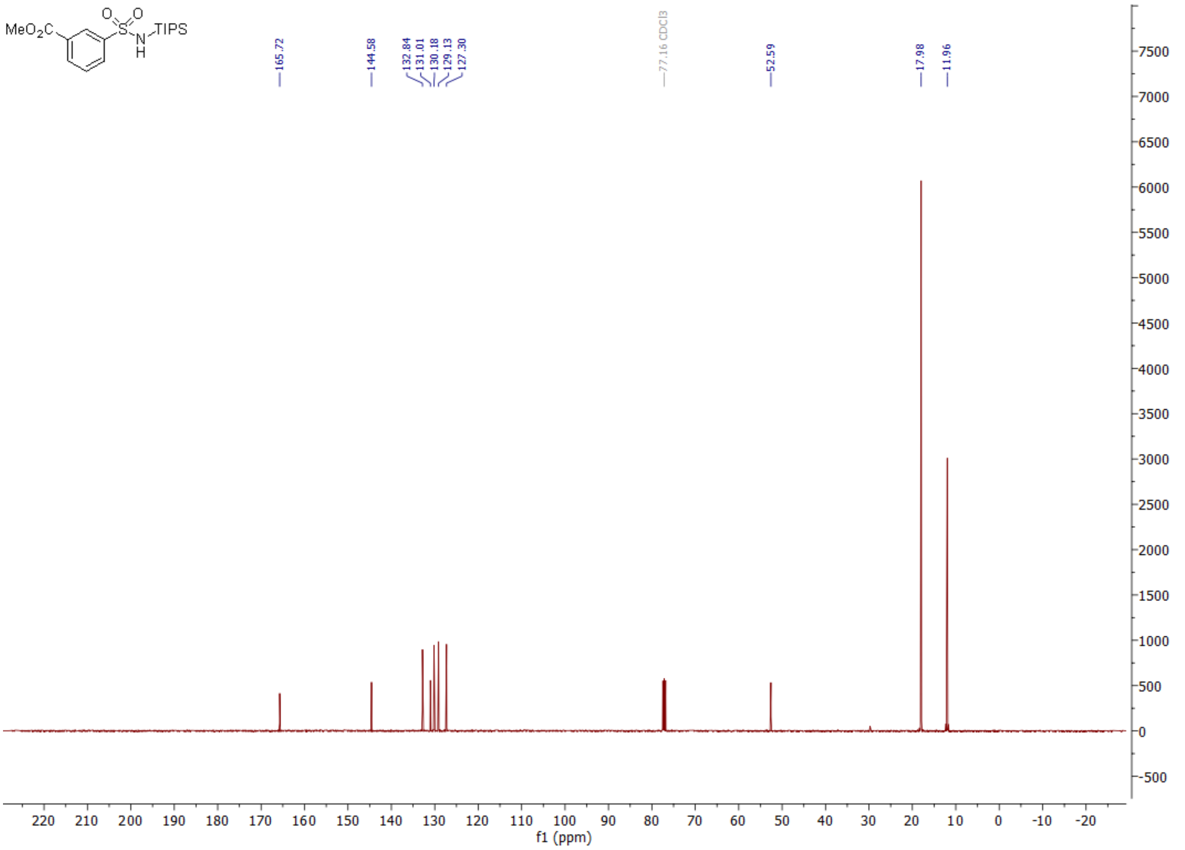

Methyl 3-sulfamoylbenzoate (4f)

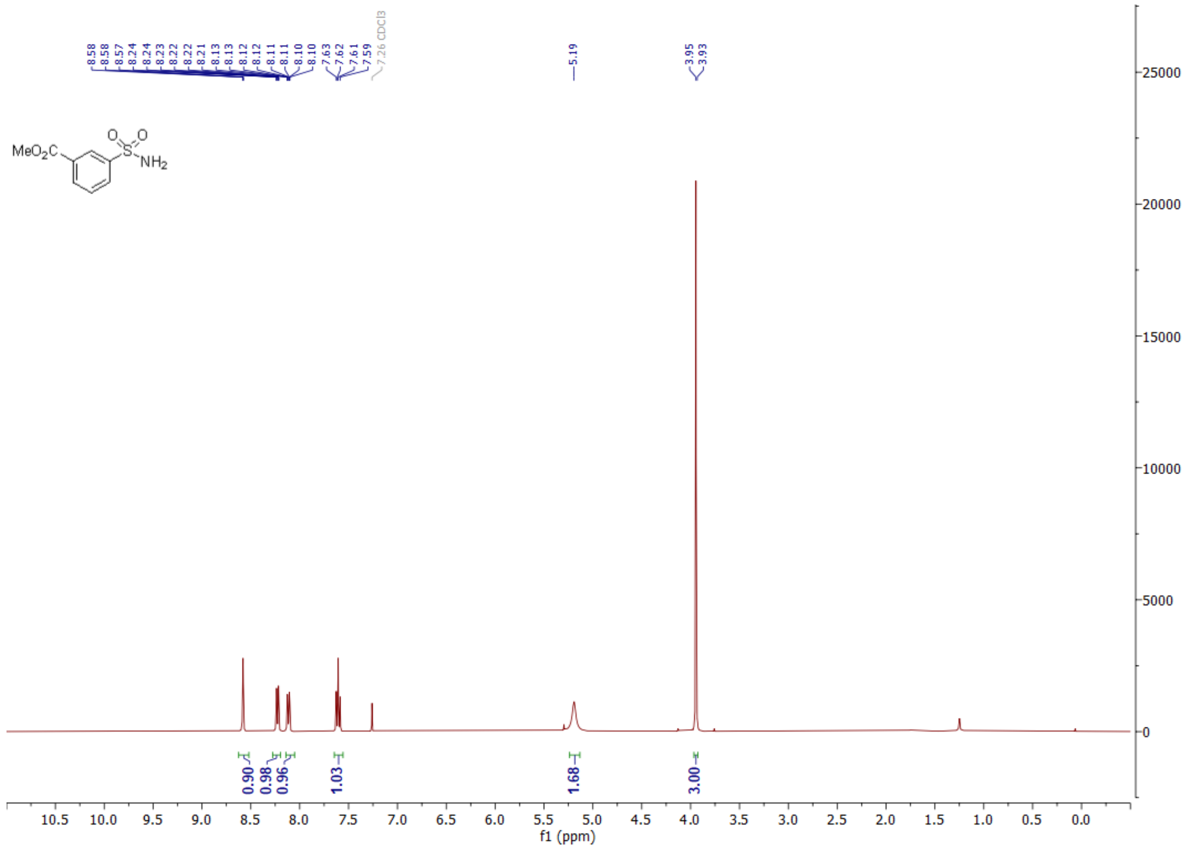

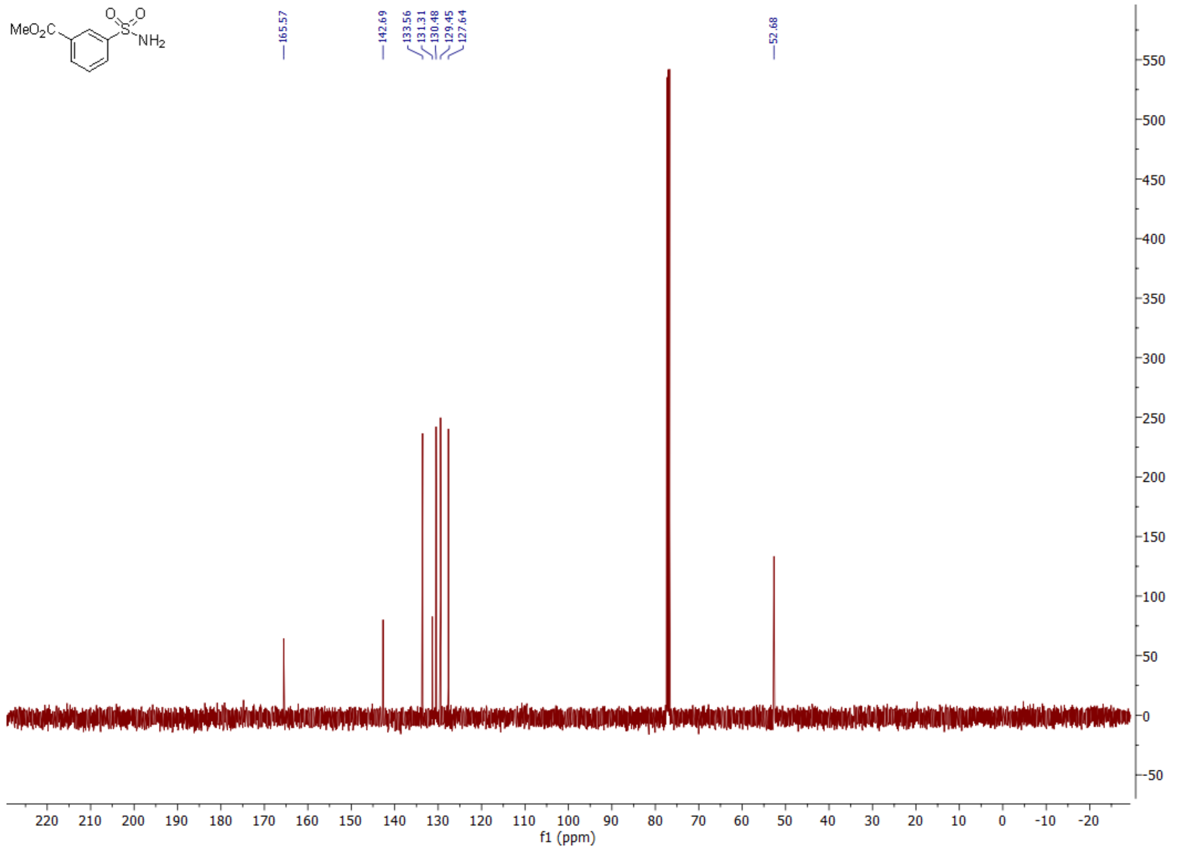

1-Tosyl-1,2,3,6-tetrahydropyridine-4-sulfinamide (5a)

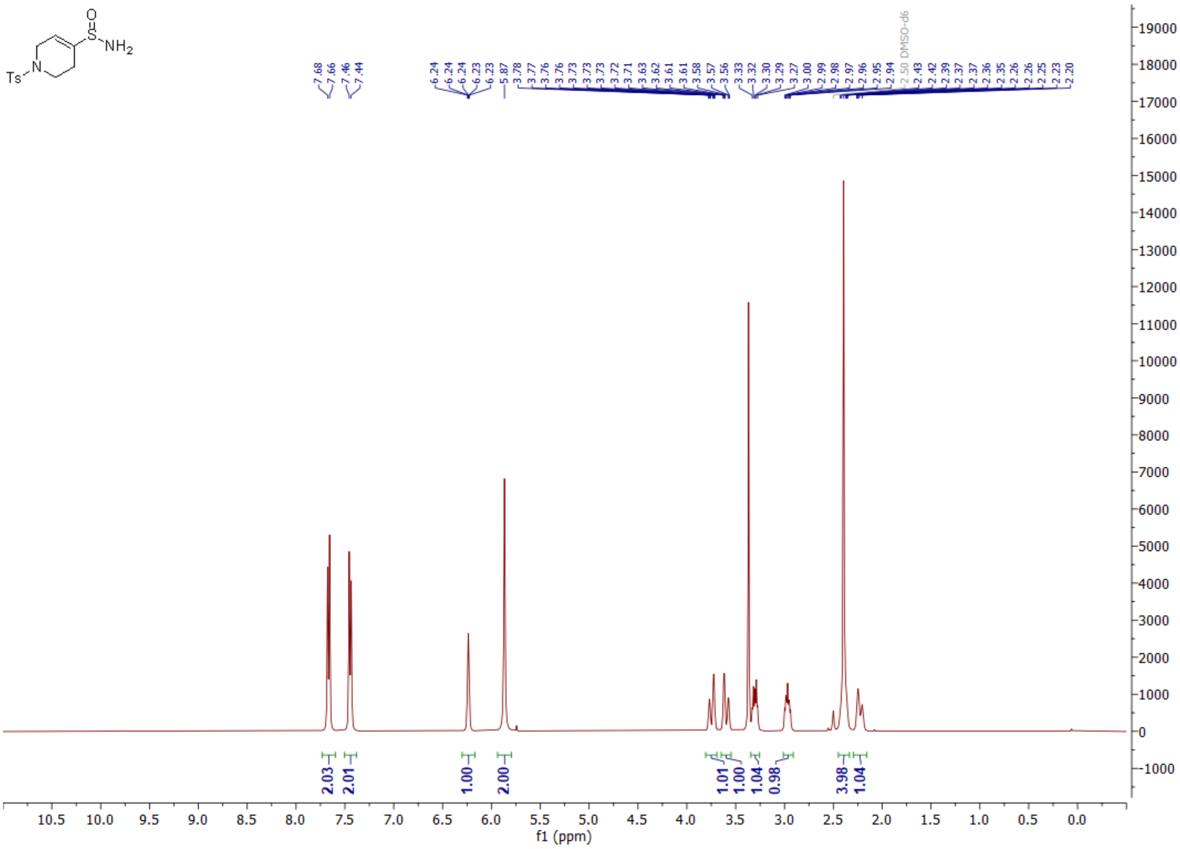

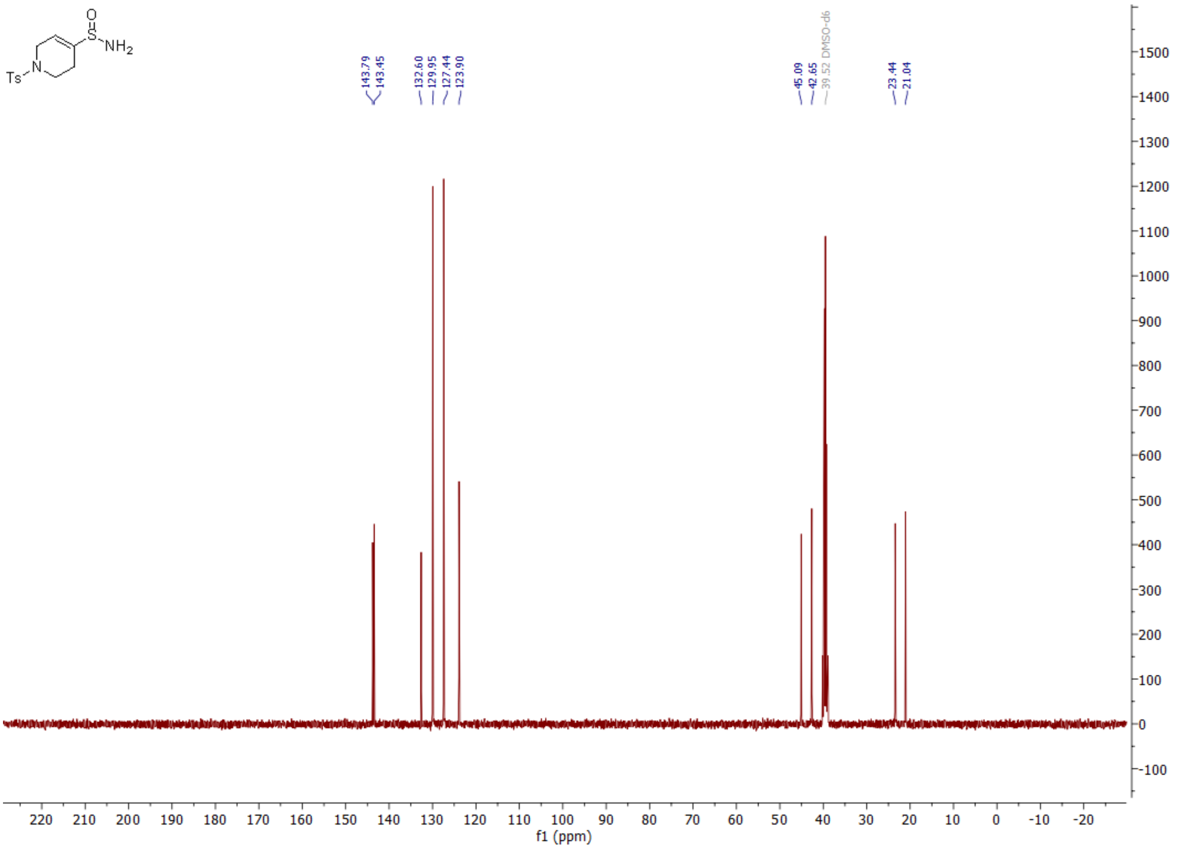

***tert*-Butyl 4-(1-tosyl-1,2,3,6-tetrahydropyridine-4-sulfonimidoyl)piperazine-1-carboxylate (5b)**

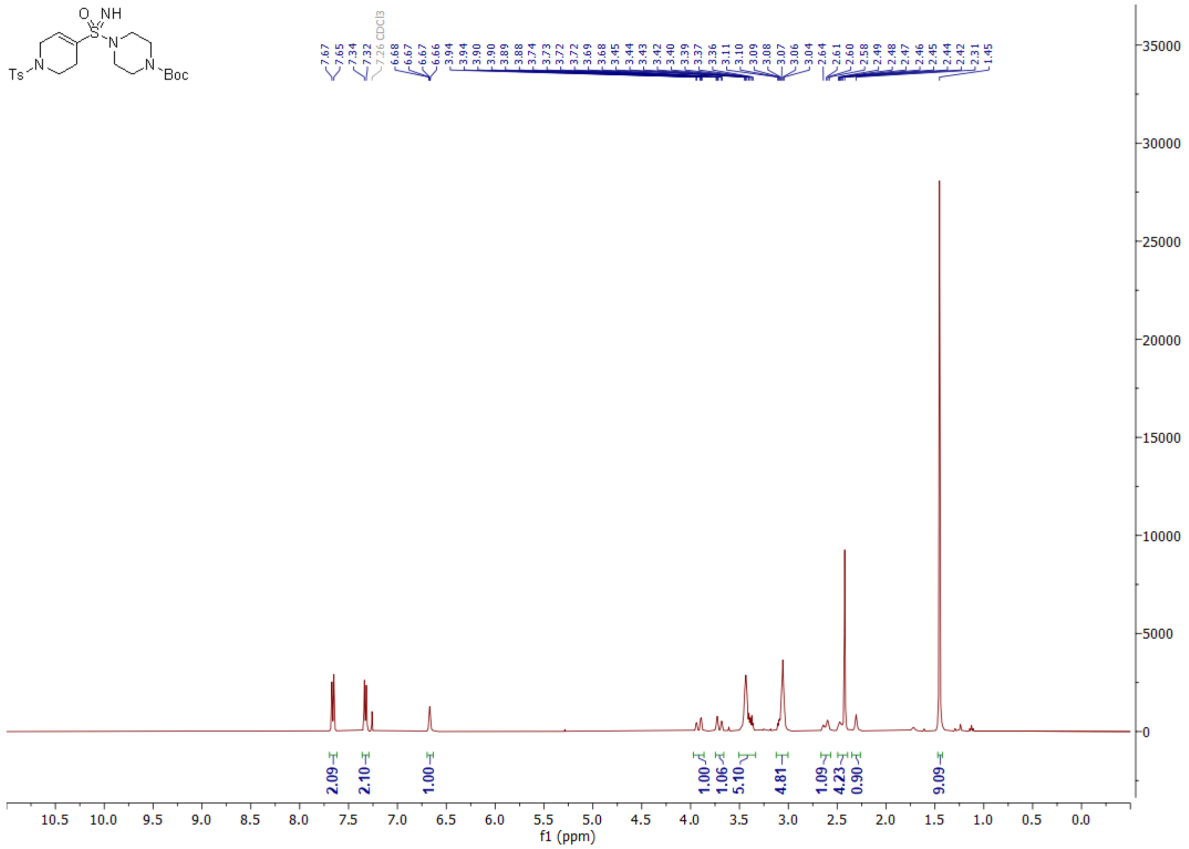

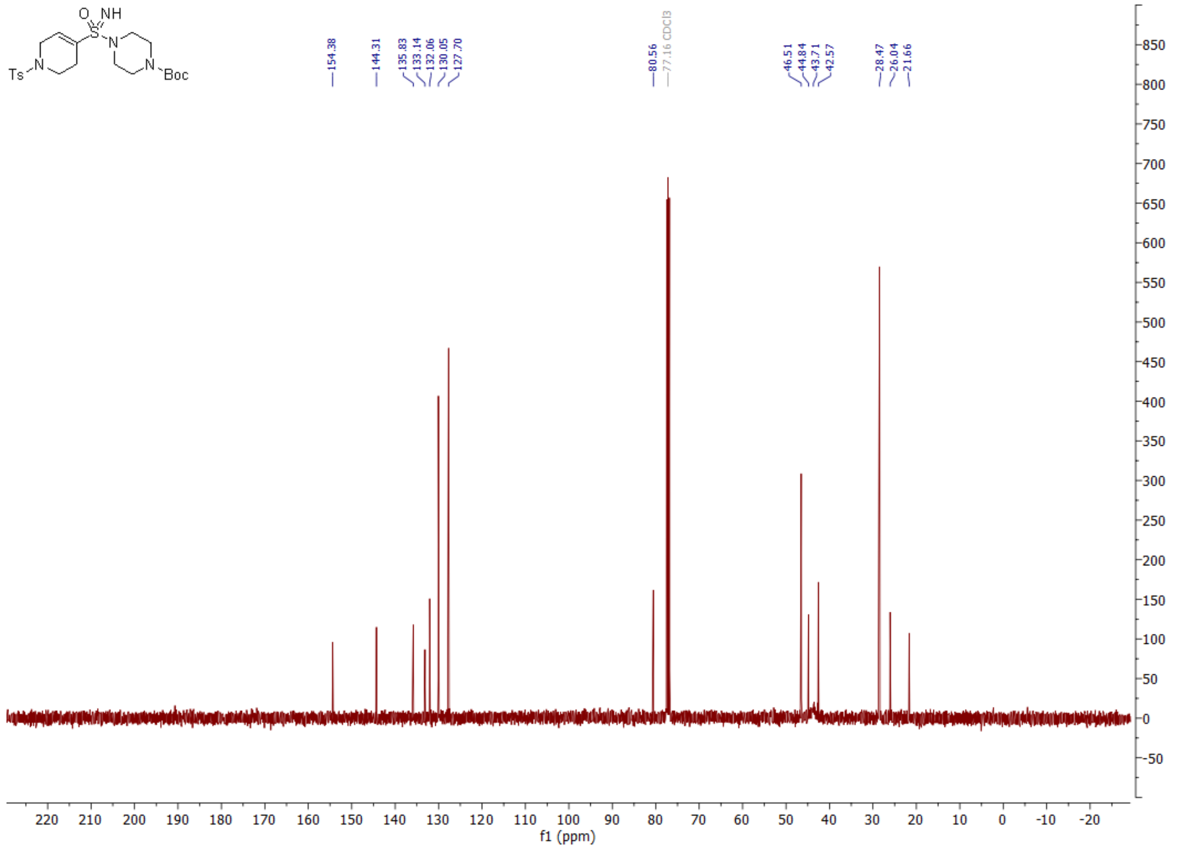

1-Tosyl-*N*-(triisopropylsilyl)-1,2,3,6-tetrahydropyridine-4-sulfonimidoyl fluoride (5c)

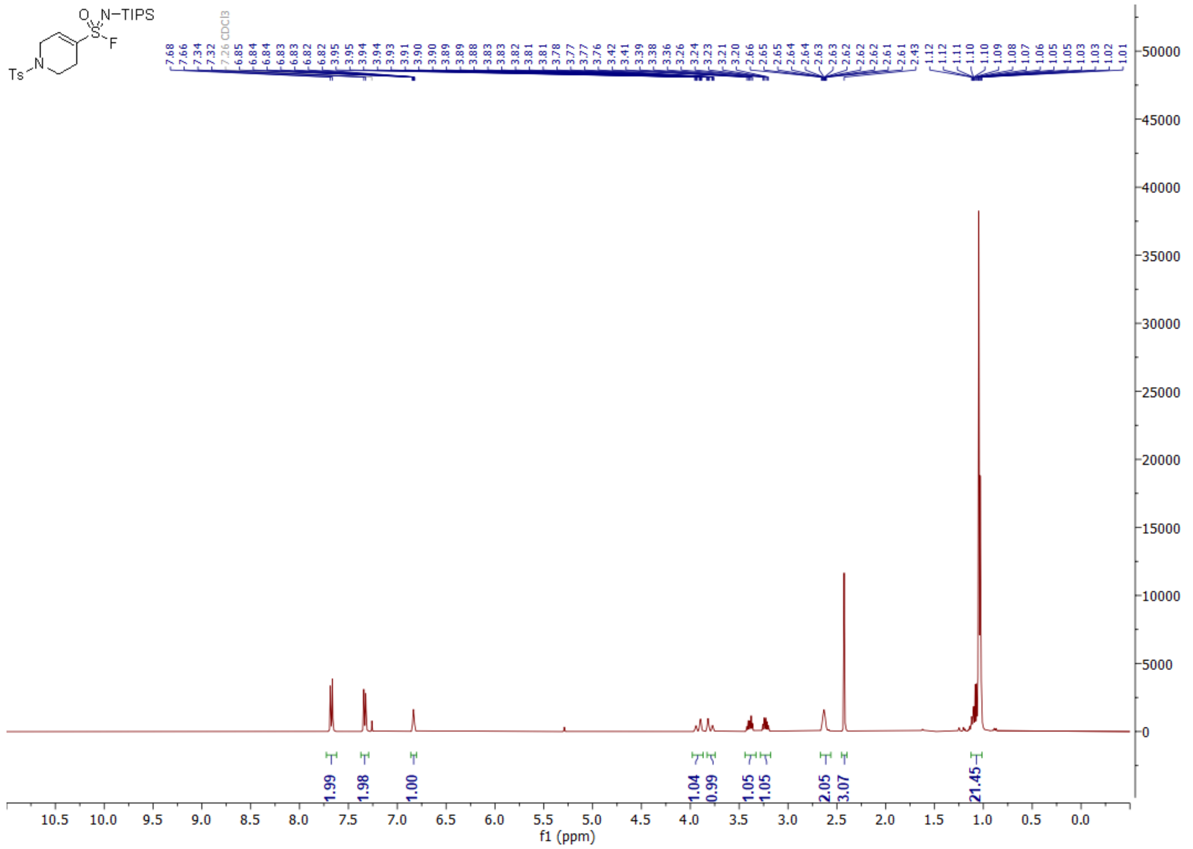

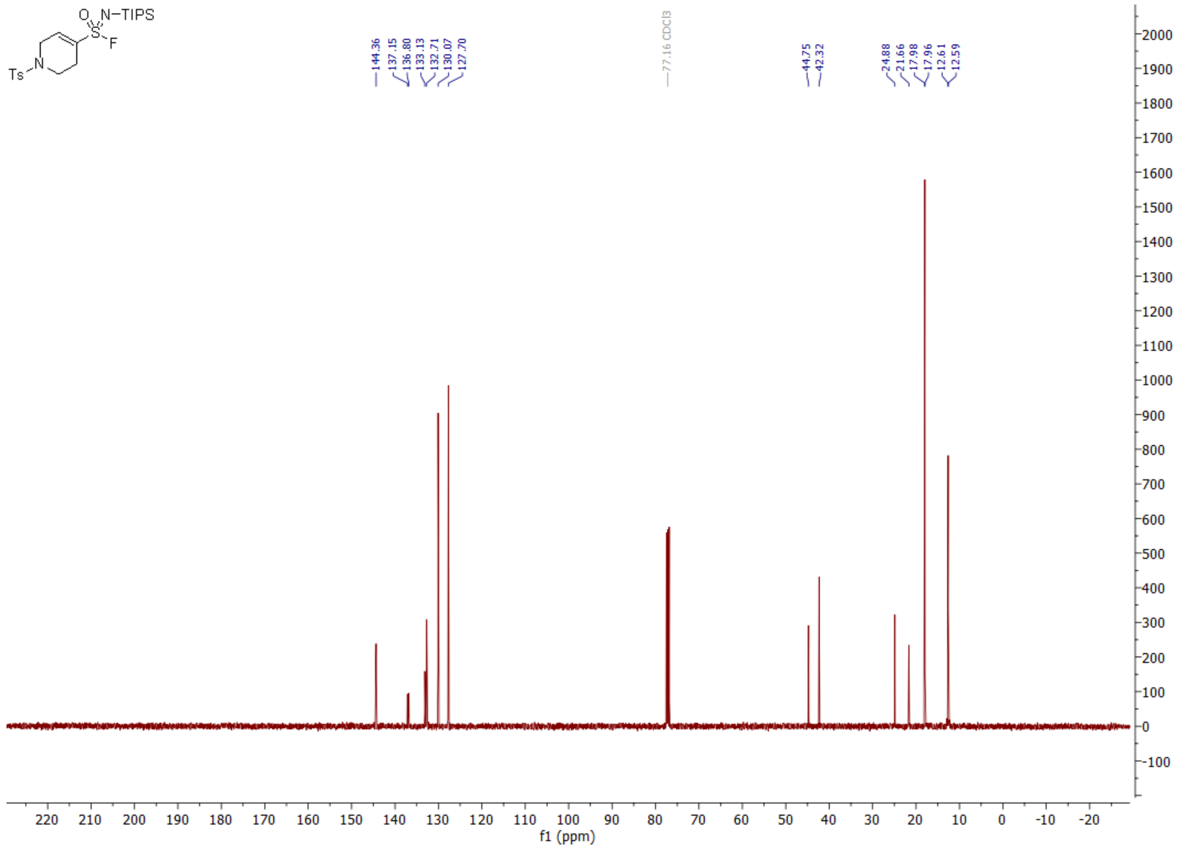

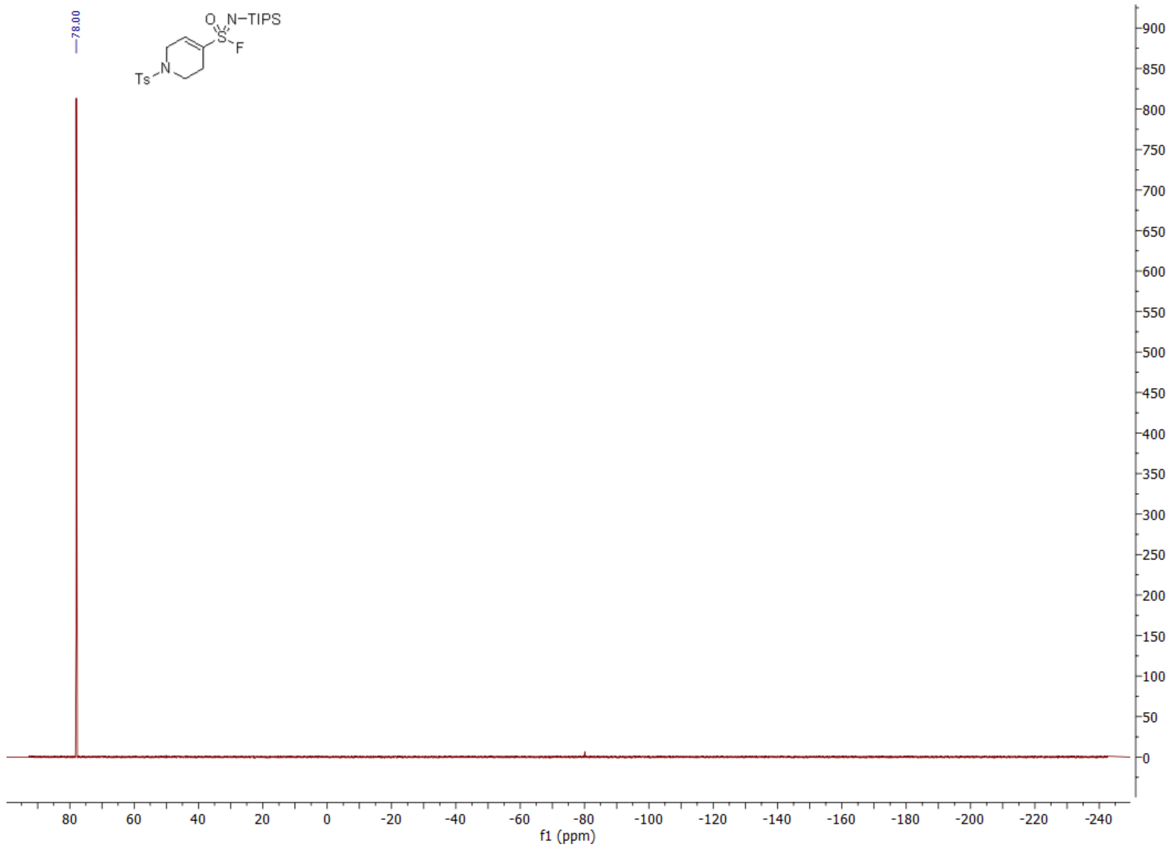

—78.00
O N−TIPS
S
F
Ts N
f1 (ppm)
